# Asymmetric Total Synthesis and Structural Revision of DAT_2_, an Antigenic Glycolipid from *Mycobacterium tuberculosis*

**DOI:** 10.1002/anie.202318582

**Published:** 2024-04-03

**Authors:** Zonghao Lin, Jeya Prathap Kaniraj, Mira Holzheimer, Jérôme Nigou, Martine Gilleron, Johan Hekelaar, Adriaan J. Minnaard

**Affiliations:** Stratingh Institute for Chemistry, University of Groningen Nijenborgh 7, 9747 AG, Groningen (The Netherlands); Stratingh Institute for Chemistry, University of Groningen Nijenborgh 7, 9747 AG, Groningen (The Netherlands); Stratingh Institute for Chemistry, University of Groningen Nijenborgh 7, 9747 AG, Groningen (The Netherlands); Institut de Pharmacologie et de Biologie Structurale, Université de Toulouse, CNRS, UPS, 205 route de Narbonne, F-31077 Toulouse (France); Institut de Pharmacologie et de Biologie Structurale, Université de Toulouse, CNRS, UPS, 205 route de Narbonne, F-31077 Toulouse (France); Stratingh Institute for Chemistry, University of Groningen Nijenborgh 7, 9747 AG, Groningen (The Netherlands); Stratingh Institute for Chemistry, University of Groningen Nijenborgh 7, 9747 AG, Groningen (The Netherlands)

**Keywords:** di*-O*-acyl trehalose, organic synthesis, stereochemistry, structural revision, antigenicity

## Abstract

DAT_2_ is a member of the diacyl trehalose family (DAT) of antigenic glycolipids located in the mycomembrane of *Mycobacterium tuberculosis* (*Mtb*). Recently it was shown that the molecular structure of DAT_2_ had been incorrectly assigned, but the correct structure remained elusive. Herein, the correct molecular structure of DAT_2_ and its methyl-branched acyl substituent mycolipanolic acid is determined. For this, four different stereoisomers of mycolipanolic acid were prepared in a stereoselective and unified manner, and incorporated into DAT_2_. A rigorous comparison of the four isomers to the DAT isolated from *Mtb H37Rv* by NMR, HPLC, GC, and mass spectrometry allowed a structural revision of mycolipanolic acid and DAT_2_. Activation of the macrophage inducible Ca^2+^-dependent lectin receptor (Mincle) with all four stereoisomers shows that the natural stereochemistry of mycolipanolic acid / DAT_2_ provides the strongest activation, which indicates its high antigenicity and potential application in serodiagnostics and vaccine adjuvants.

## Introduction

**Figure 1.**
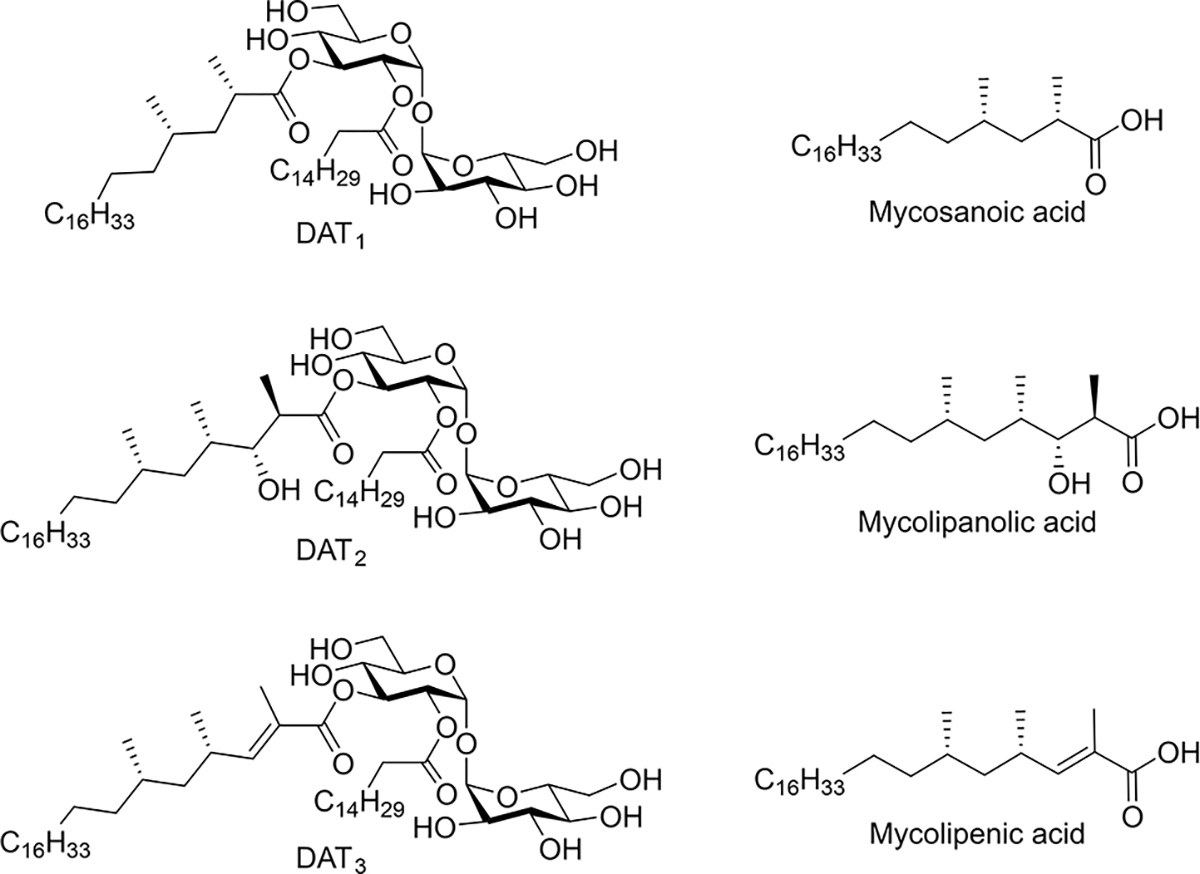
Chemical structures of the mycobacterial diacyl trehaloses DAT_1_, DAT_2_, DAT_3,_ and the corresponding fatty acids.

*Mycobacterium tuberculosis* (*Mtb*) is the causative agent of the disease tuberculosis (TB). In 2021, around 10.6 million people were diagnosed and 1.6 million people died from TB, the second leading infectious killer after COVID-19 (more than HIV/AIDS).^[1]^ The mycobacterial cell envelope is a contributing factor to the resilience of *Mtb* in host cells.^[2]^ About 40% of the dry weight of its cell wall is composed of lipids^[3]^ and a large percentage of the coding capacity of the bacterial genome is used for lipid biosynthesis and degradation.^[4]^ The lipid-rich cell wall of *Mtb* is involved in the regulation of the transport of nutrients, toxic host-cell effector molecules, and anti-tuberculosis drugs. In the last decades, it has been shown that many of these cell wall components have antigenic properties and/or possess immunomodulatory functions.^[5]^ Long-chain multi-methyl-branched fatty esters containing trehalose make up a significant part of the membrane, are unique to *Mycobacteria*, and are involved in pathogenesis.^[6]^ Di-*O*-acyl trehaloses include a family of glycolipids (Figure 1), DAT_1_, DAT_2_, DAT_3_ and DAT_4_.^[7]^ DAT_3_ and DAT_4_ were isolated as minor components and DAT_4_ remains unidentified.^[8]^ “DAT” often refers to the family of the four compounds because it is impossible to get the pure DAT compounds from the natural extract.^[9]^

The mycobacterial lipids component DAT, which is specifically found in *Mtb* and *M. fortuitum*, is crucial to the pathogenesis and structure of the cell envelope, and promotes bacterial intracellular survival.^[10]^ DAT is capable of modulating host immune responses.^[11,12]^ Macrophage-inducible C-type lectin (Mincle) is an innate immune receptor, and its activation is a central part of the innate immune response to *Mtb.*^[12b]^ DAT can act both as an antigen for T cells and an activator for the innate Mincle receptor, and small structural differences determine recognition by different parts of the immune system.^[13]^ The use of DAT in serodiagnosis has been postulated.^[14]^ In addition, there is a series of studies on the detection of antibodies against DAT and developing novel vaccine adjuvants from Mincle agonists.^[15]^

Whereas the structures of DAT_1_ and DAT_3_ are undisputed, the history of DAT_2_ and its acyl substituent mycolipanolic acid is long and winding. Mycolipanolic acid was first described by Coles and Polgar in 1968 after its isolation from a massive culture of *Mtb.*^[16]^ The compound, present as several homologues (e.g. length of the chain), was coined mycolipanolic acid. The C4 and C6 methyl substituents of the deoxypropionate chain were established to have the *S* (at that time “L”) configuration. Later it was shown that all methyl-branched fatty acids produced by the polyketide synthase PKS2, e.g. mycolipanolic acid, mycosanoic acid, mycolipenic acid, phthioceranic acid and hydroxy phthioceranic acid, indeed have an all-*S* configuration in the deoxypropionate chain.^[8]^ Based on IR-spectroscopy, 100 MHz ^1^H NMR, and base-induced epimerization of the methyl group at C2 of the corresponding methyl ester, it was postulated that the C2 methyl substituent and the C3 hydroxy group in mycolipanolic acid had an *erythro* relationship. It was also explicitly mentioned that the absolute configuration at C2 and C3 could not be established.

**Figure 2.**
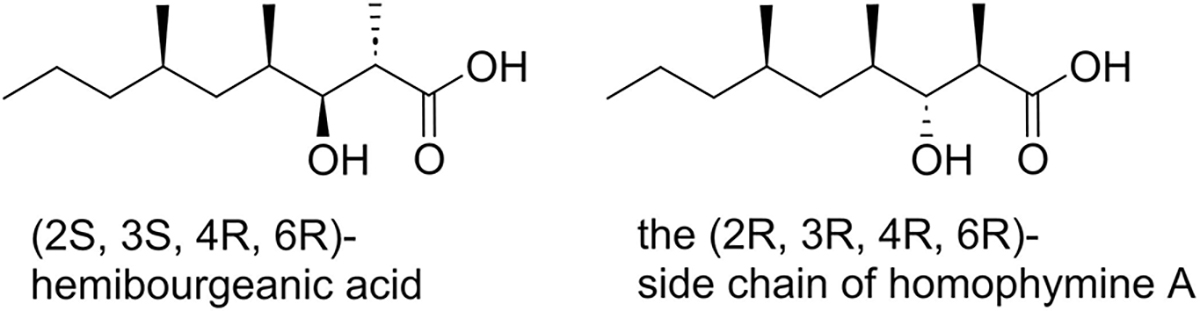
Hemibourgeanic acid and the side chain of homophymine A.

Several years later, in an unrelated study the dimer of a compound closely related to mycolipanolic acid, albeit with a much shorter alkyl chain,^[17]^ was found in a lichen. The compound was called bourgeanic acid (the monomer hemibourgeanic acid, Figure 2). The absolute stereochemistry of a derivative of hemibourgeanic was obtained by crystal structure determination.^[18]^ Hemibourgeanic acid has the 2*S*, 3*S*, 4*R*, 6*R* stereochemistry and is therefore enantiomeric with mycolipanolic acid at the C4 and C6 methyl branches. Over the years, several syntheses of hemibourgeanic acid were disclosed^[19]^ but no attempt was made to relate the spectral data to those of mycolipanolic acid.

In 1996, Wallace and Minnikin prepared mycolipanolic acid as a stereorandom mixture,^[20]^ and wrote, referring to the papers of Coles and Polgar “*The absolute configuration of this mycolipanolic acid has been established as (2S, 3R, 4S, 6S)-3-hydroxy-2,4,6-trimethyltetracosanoic acid. A definite syn relationship has been proven between the C2 methyl and the C3 hydroxy group.*” This clearly was a too bold statement and in addition, the earlier proposed *erythro* relationship would translate into an *anti*-relationship. As the synthesis was stereorandom, the authors could not contribute further to a confirmation of the structure.

In 2010, our group developed a stereoselective synthesis of mycolipanolic acid, with 2*S*, 3*R*, 4*S*, 6*S* configuration, and its related fatty acids.^[21]^ We compared the NMR data with the numerical data available in literature and the optical rotation with the one reported by Coles and Polgar while the NMR spectra were not available. These seemed in agreement, although the magnitude of the optical rotation of mycolipanolic acid is small (7°) and allows no conclusion in terms of stereochemistry. At that time, we did not have access to natural mycolipanolic acid. In parallel, the group of Zampella disclosed a stereoselective synthesis of (2*R*, 3*R*, 4*R*, 6*R*)-3-hydroxy-trimethyloctanoic acid (Figure 2) and showed it to be part of the depsipeptide homophymine A.^[22]^ Hemibourgeanic acid and the side chain of homophymine A are therefore epimers at C2 and C3, but comparison of the NMR data showed that these are very similar. In retrospect, the stereochemistry of mycolipanolic acid had not been entirely solved, and comparison of numerical ^1^H NMR data present in literature was insufficient. This was underscored by the structure elucidation of (−)-SCH 64874 and hirsutellomycin which possess a 3-hydroxy-2,4-dimethyl hexanoate side chain (so without a methyl substituent at C6). In order to establish the stereochemistry, the authors had to prepare all four diastereomers which showed small but distinct differences in the ^1^H- and ^13^C NMR spectra.^[23]^

In 1989, the mycolipanolic ester DAT_2_ was isolated by Daffé et al. The compound was initially named sulfolipid-IV because it was described as a 2,3-diacyl-trehalose-2*’*-sulfate.^[16[AM1],24]^ The structure of this family of acyl trehaloses was corrected to be a group of 2,3-di-*O*-acyltrehaloses.^[7,25]^ DAT_2_ was shown to contain a mycolipanolic acid residue at the C3 position, but the stereochemistry of mycolipanolic acid was not further studied.^[7]^

Recently, we reported the first total synthesis of DAT_1_, DAT_2_, and DAT_3_, in order to study the ability of the individual DAT family members to activate Mincle. Upon analysis by HPLC-MS, the retention time and mass spectra of DAT_1_ and DAT_3_ coincided with those of the natural material. The retention time of synthetic DAT_2_, however, showed more than 1 min difference with that of the natural product (available as a mixture), although the mass spectra were virtually indistinguishable.^[9]^ Thus, we suspected that the structure of DAT_2_ in literature was incorrect.

The results forced us to re-evaluate the stereochemistry of mycolipanolic acid, because other errors in the assigned structure of DAT_2_ would supposedly have led to differences in the mass spectra. In the biosynthesis of mycolipanolic acid, being a fatty acid-polyketide hybrid, the stereochemistry of methyl-branched C4 and C6 is determined by the enoyl reductase functional domain of PKS2, but the stereochemistry at the C3 and the C2 position is determined by the ketoreductase and the ketoacyl synthase domain, respectively.^[26]^ So, there is no obvious connection between the stereochemistry of C6/C4 and of C2/C3, and there is insufficient information of the structure of PKS2 to predict the stereochemistry of its products.

To clarify this situation, we decided to embark on the synthesis of all four diastereomers of mycolipanolic acid, keeping the stereochemistry at C4 and C6 fixed in the *S* configuration. These stereoisomers would be used to prepare the corresponding stereoisomers of DAT_2_ in order to explain the difference in HPLC retention time. Finally, we were interested to determine to what extent the stereochemistry of the mycolipanolic acid residue influences the potency of DAT_2_ to activate Mincle. We have shown previously that the structure of the residue, mycosanoic acid, mycolipanolic acid or mycolipenic acid, significantly influences activation of Mincle.^[9]^ Determination of the influence of stereochemistry would bring this fine-specificity one step further.

It is important to establish the correct structure of DAT_2_, not just as an intellectual challenge. There is a need for better, in particular faster and low-cost, diagnostics for tuberculosis infection. The diacyl trehaloses could play a role in ELISA-type assays, and in order to achieve this, synthetic antigens of well-defined structure are essential. These lipids might also play a role in the development of novel adjuvants, given their activation of the Mincle receptor.

## Results and Discussion

### Synthesis of four stereoisomeric mycolipanolic acids

**Scheme 1.**
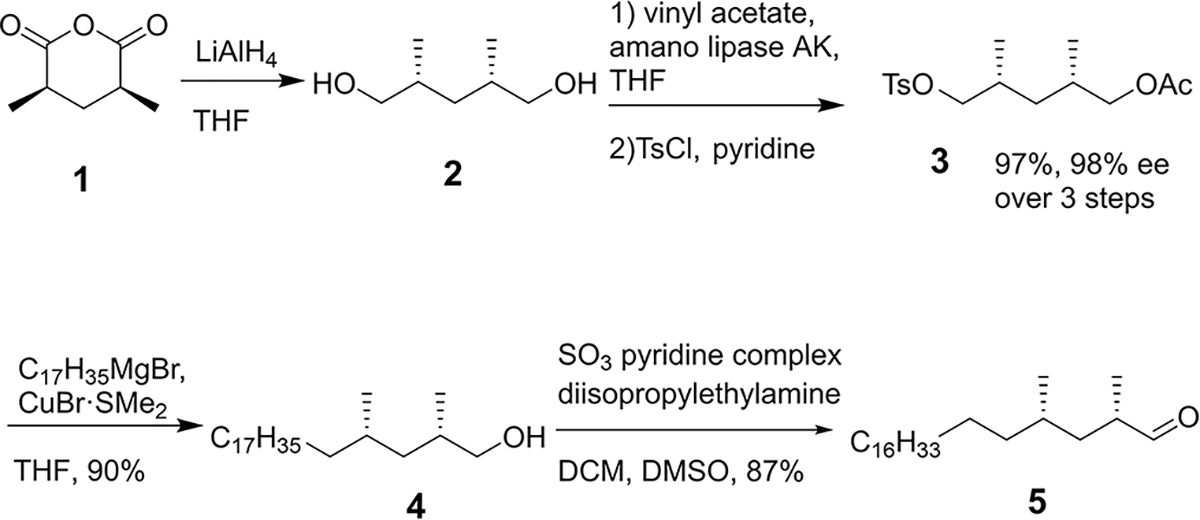
Synthesis of the common intermediate **5** (THF = tetrahydrofuran, TsCl = p-tolylsulfonyl chloride, DCM = dichloromethane, DMSO = dimethylsulfoxide).

The synthesis of the literature-proposed structure of mycolipanolic acid involved copper-catalyzed asymmetric conjugate addition (Cu-cat. ACA) and an Evans’ aldol reaction to introduce the stereocenters.^[9,21]^ For the current study, we developed a considerably more efficient synthesis based on the work of Breit et al.,^[27]^ (Scheme 1) to prepare aldehyde **5** by starting from *meso*-anhydride **1**. Reduction of **1** with LiAlH_4_ provided the desired *meso-*diol **2**, and a subsequent enzymatic desymmetrisation with amano lipase AK provided the mono-acetate in excellent selectivity (98% *ee*) and near quantitative yield. The heptadecanyl chain was installed by a modified copper catalysed cross-coupling reaction^[28]^ with tosylate **3**, with concomitant removal of the acetate group. This was followed by a Parikh–Doering oxidation, to give aldehyde **5** in high yield. In this way, the synthesis of **5** was shortened and provided a considerably higher yield.

**Scheme 2.**
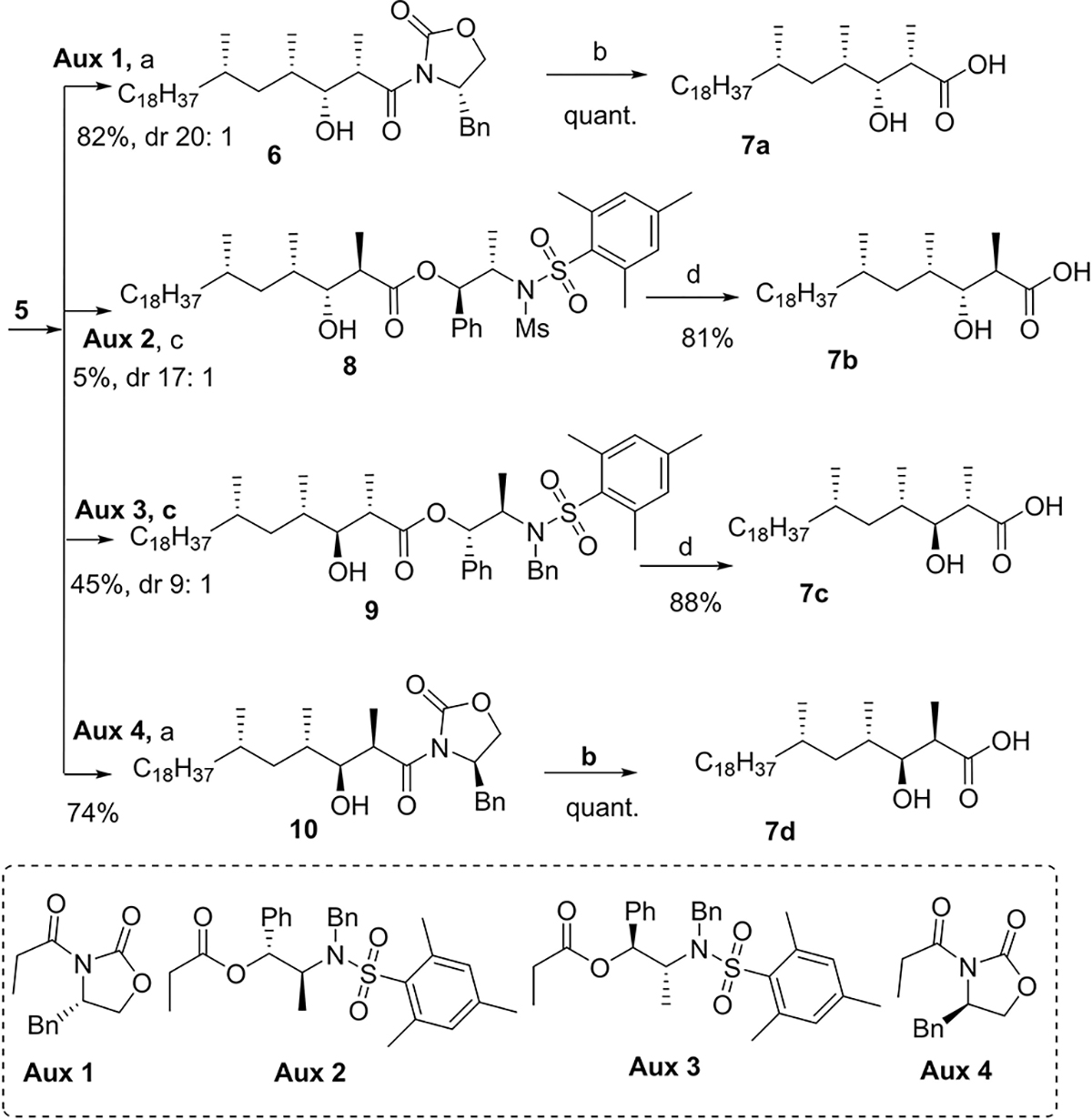
Asymmetric synthesis of four stereoisomeric mycolipanolic acids. Stereocenters of the hydroxymethyl units were stereoselectively installed through Evans and Abiko-Masamune aldol reactions. Reagents and conditions: a) Bu_2_BOTf, Et_3_N, DCM, −78°C to rt; b) LiOH, H_2_O_2_, THF, H_2_O; c) Cy_2_BOTf, Et_3_N, DCM, −78°C to −10°C; d) TBAOH, THF.

From **5**, all four stereoisomeric mycolipanolic acids could be synthesized in a limited number of steps (Scheme 2). To install the two remaining stereocenters, Evans aldol reactions^[29]^ were performed for the preparation of the mycolipanolic acids with a *syn*-hydroxymethyl unit. Abiko-Masamune aldol reactions^[30]^ were applied to prepare the *anti*-hydroxymethyl units. The different stereochemistry of the chiral auxiliaries led to the different relative configuration of the β-OH and α-methyl groups, and provided the aldol products **8**–**10** in an excellent diastereomeric ratio (dr). As the solubility of the substrates significantly decreased at lower temperature, the procedure of the aldol reactions leading to **9** and **10** had to be carefully optimized but finally were significantly improved to provide 45% and 74% yield, respectively. The 5% yield of **8** couldn’t be improved, however. It was attempted to prepare **7b** through a magnesium chloride mediated Evans-type anti-aldol reaction^[31]^ and alternatively a Mukaiyama aldol reaction,^[32]^ but neither of these worked. The aldol products **8**, **9** and **10** were finally hydrolyzed by lithium hydroxide (LiOH) or tetrabutylammonium hydroxide (TBAOH) to give diastereomers of mycolipanolic acid (**7b**–**d**).

### Preparation of suitably protected mycolipanolic acids

**Scheme 3.**
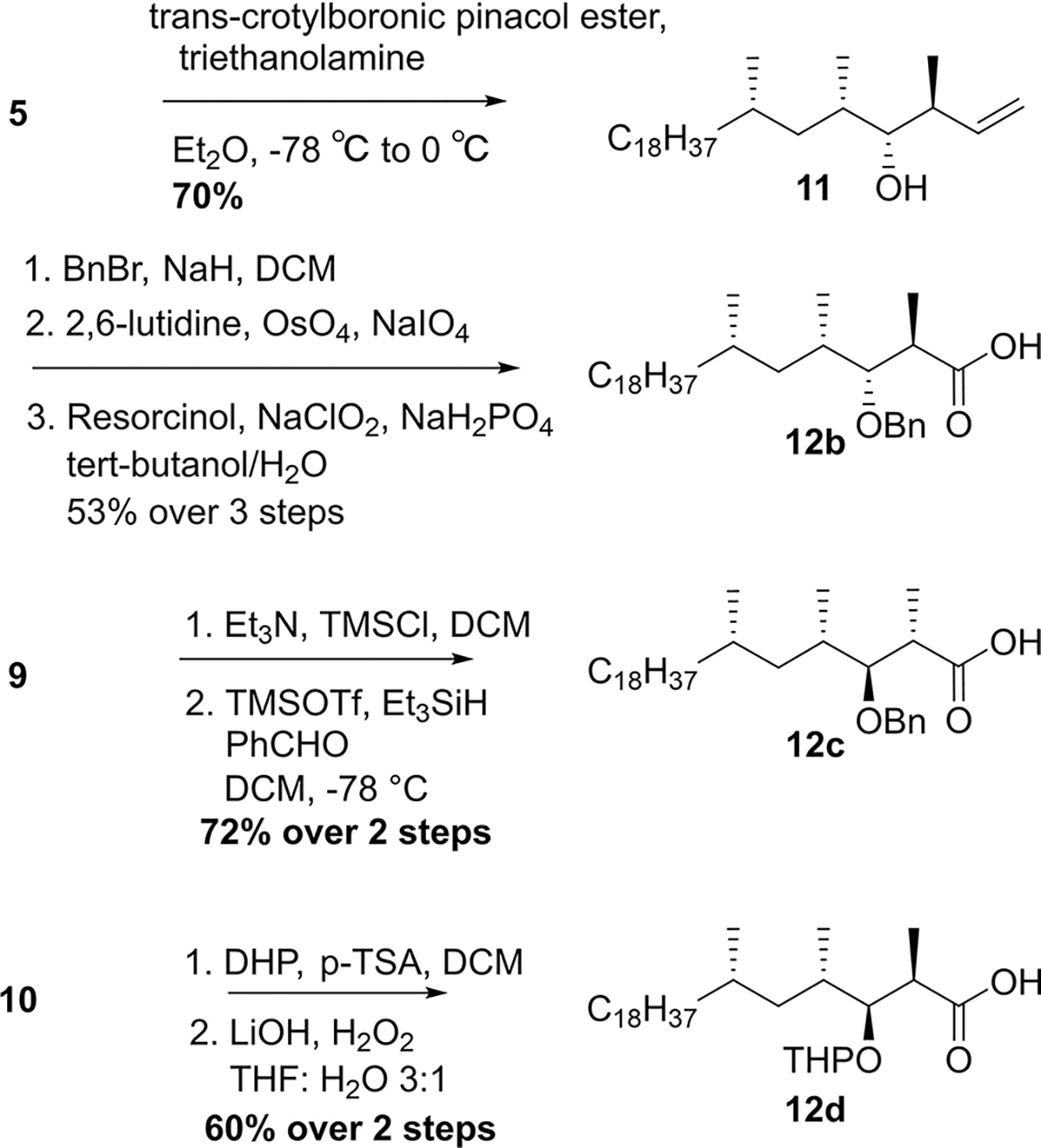
Preparation of β-OH protected mycolipanolic acids **12b**–**12d** (Et_2_O = diethylether, BnBr = benzyl bromide, TMSCl = trimethylsilyl chloride, TMSOTf = trimethylsilyl trifluoromethanesulfonate, DHP = di-hydropyran, p-TSA = p-tolylsulfonic acid).

In the initial total synthesis of DAT_2_, now called DAT_2_-a, we reported a direct esterification of mycolipanolic acid **7a** and suitably protected 2-palmitoyl trehalose **13**.^[9]^ The hydroxy group in **7a** remained unprotected. However, this approach did not work for the esterification of the other stereo-isomers of mycolipanolic acid. Neither Yamaguchi esterification nor Shiina esterification of the other three acids **7b**–**d** with protected 2-palmitoyl trehalose **13** or **16** worked well. One of the problems turned out to be that the β-OH group in **7b**–**d** took part in intra- and intermolecular esterification reactions, aggravated by the slow esterification reaction of the 3-OH of the trehalose core. This hydroxy group is difficult to access, in particular for the voluminous fatty acids. So, we decided to prepare the mycolipanolic acids with the β-OH group protected as a tetrahydropyran (THP) acetal or a benzyl (Bn) ether, which are not too bulky (Scheme 3). The protection procedures were carefully optimized to avoid epimerization of the α methyl group or elimination of the β-OH group.

Abiko-Masamune aldol reaction gave a very low yield of **8** (5%), and THP protection^[23]^ and benzylation^[33]^ of **8** either gave very low yield or didn’t proceed at all. Therefore we changed synthesis strategy for this particular isomer, and applied a Brown crotylation reaction with an E-crotylboronic ester to produce 2,3-anti-3,4-syn diastereomer **11**.^[34]^ This provided a much higher yield and good dr. After benzylation, the double bond was cleaved successfully through Lemieux–Johnson oxidation with OsO_4_/NaIO_4_ and was further oxidized to the corresponding acid **12b** by Pinnick oxidation.

Silylation of **9** was followed by benzylation and removal of the auxiliary^[35]^ to give the acid **12c**. The β-OH group in **10** was protected as a THP acetal and gave enantiopure acid **12d** after hydrolysis.

### Esterification of mycolipanolic acid isomers with trehalose

Suitably protected trehalose **13** was obtained starting from α,α-trehalose through a desymmetrization strategy previously applied in the synthesis of trehalose-based sulfoglycolipids,^[36]^ whereas trehalose **16** has been used to prepare sulfolipid SL-1 and Ac_2_SGL analogues.^[37]^

**Scheme 4.**
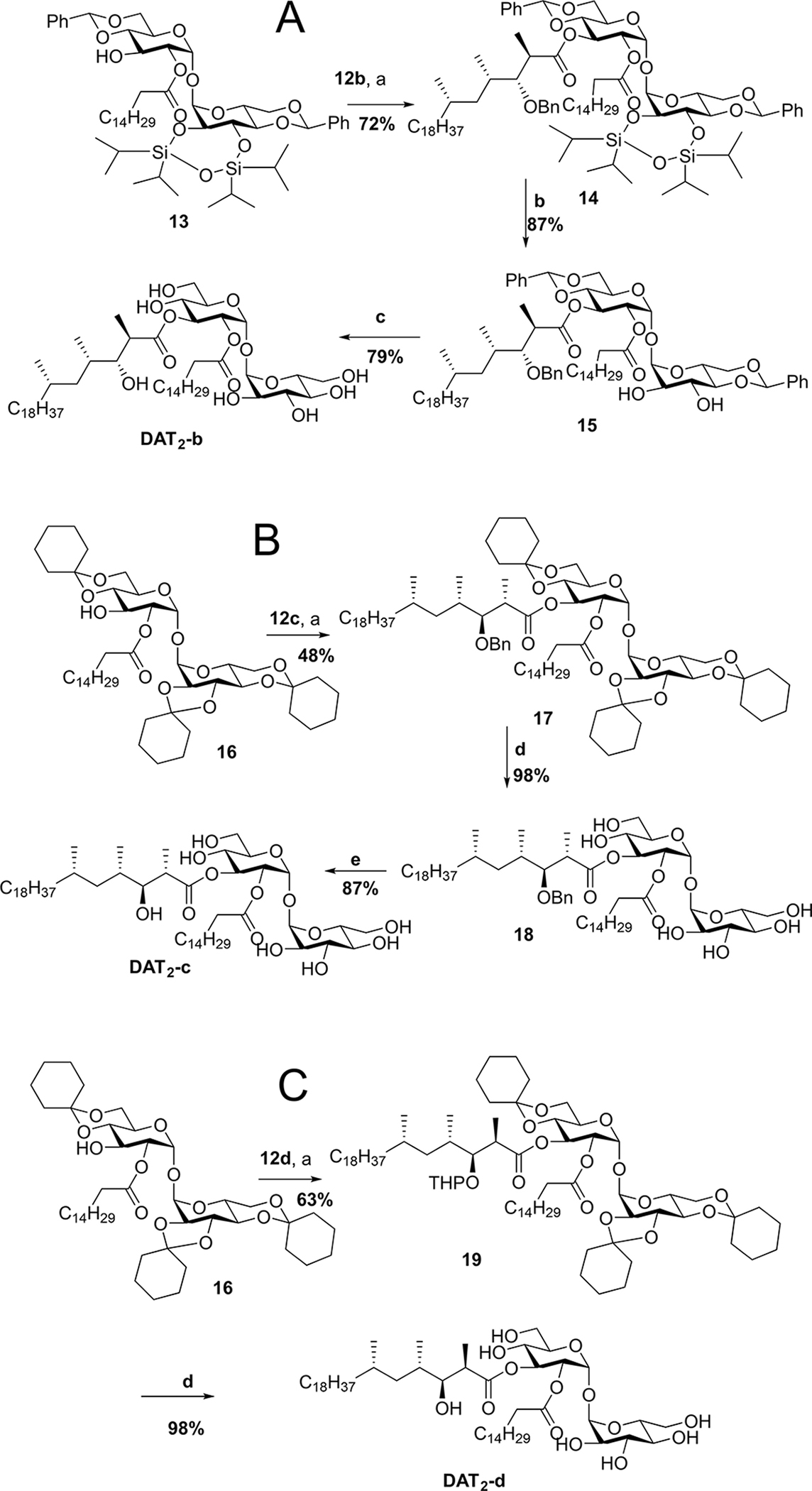
Completion of the synthesis of the diastereomers of DAT_2_. Suitably protected isomeric mycolipanolic acids were incorporated into protected palmitoyl trehaloses by Shiina esterification and gave the stereoisomers of DAT_2_ after deprotection. Reagents and conditions: a) Et_3_N, dimethylamino pyridine (DMAP), MNBA, DCM; b) Tetrabutylammonium fluoride (TBAF), AcOH; c) Pd/C, Pd(OH)_2_, H_2_, THF; d) AcOH, DCM, 50°C; e) H_2_, Pd/C, EtOAc, EtOH.

With the stereochemically pure acids **12b**–**d** in hand, the esterification of the palmitoylated trehaloses **13** and **16** was accomplished by following the Shiina procedure with the reagent 2-methyl-6-nitrobenzoic anhydride (MNBA). The reactions had to be carefully optimized by limiting the amount of base and the time for acid activation, and applying a concentration of the acid above 0.1 M. With the optimized conditions, esterification of **12b** with **13** produced **14** in 72% yield (Scheme 4A). Removal of the silyl protecting group followed by palladium-catalyzed hydrogenolysis^[36a]^ provided DAT_2_-b in good yield.

The esterification of **12c** and **12d** with **13** did not proceed, presumably because of the different conformations of these mycolipanolic acid stereoisomers. Better results, although for unknown reasons, were obtained with **16** to prepare **17** (Scheme 4B) and **19** (Scheme 4C) via Shiina esterification in 48% and 63% yield, respectively. The cyclohexylidene acetals of **17** were removed in aqueous AcOH (80%) at reflux and followed by hydrogenolysis to afford DAT_2_-c. Removal of the cyclohexylidene and the THP acetals in **19** was carried out in one step to give DAT_2_-d in 98% yield. This completed the synthesis of four stereoisomers of mycolipanolic acid and the corresponding DAT_2_ isomers. The compounds were characterized by ^1^H NMR, ^13^C NMR and HRMS.

### NMR analysis of synthetic and natural DAT_2_

**Figure 3.**
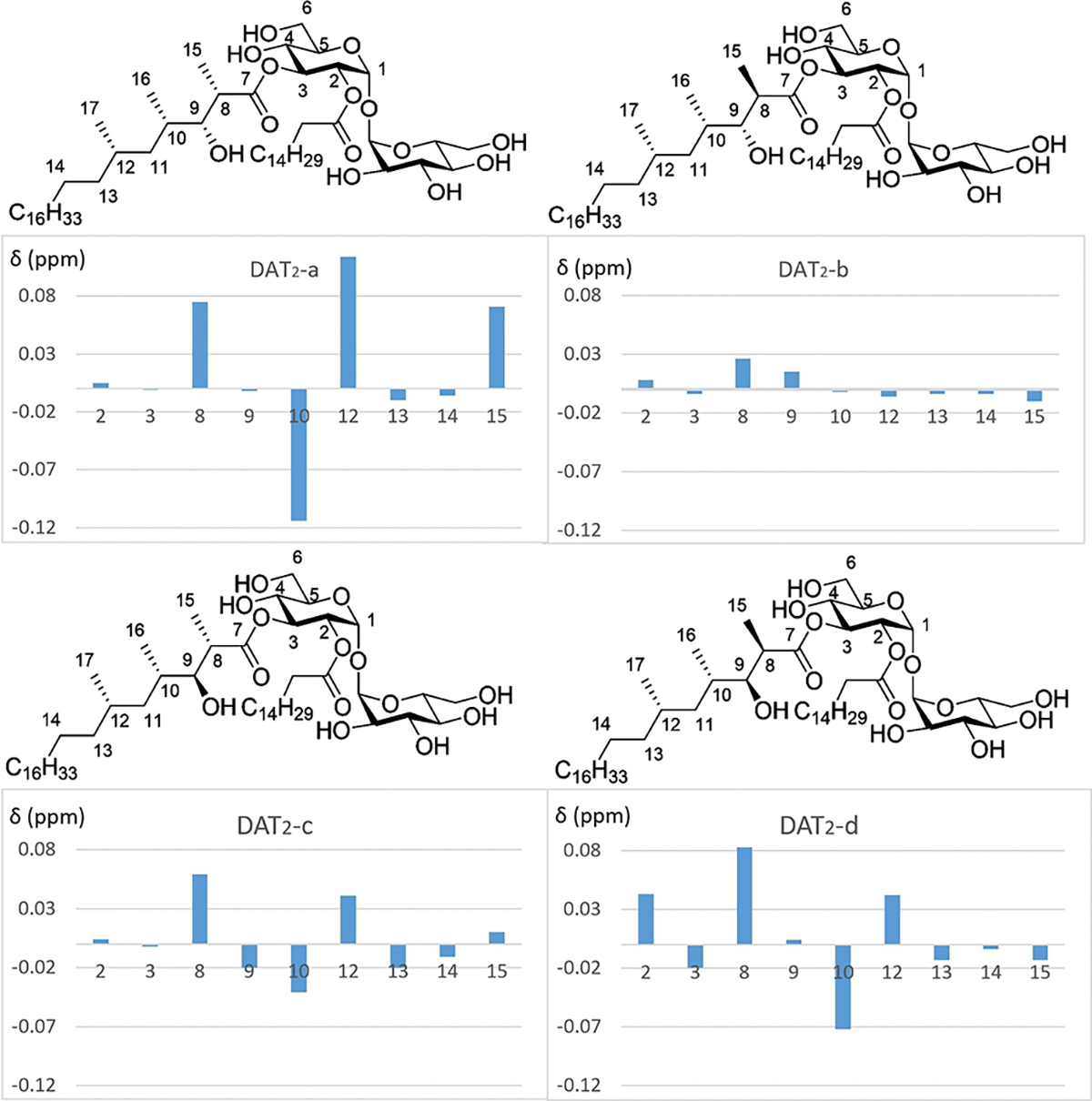
Comparison of the ^1^H NMR (600 MHz, CDCl_3_/CD_3_OD 4/1) spectra according to Hoye. The columns represent differences between each of the synthetic isomers and the natural sample. DAT_2_-b matches best with the signals from the natural sample (DAT_n_).

With four stereoisomers of DAT_2_ in hand, we sought to determine whether one of the synthesized glycolipids would match the structure of natural DAT_2_ (as a constituent of the diacyl trehalose family of compounds) present in pathogenic *Mtb.* To meticulously compare the ^1^H NMR spectra of the four synthetic diastereomers with that of natural DAT (here called DAT_n_), a purification of “DAT_n_” was undertaken using the reference laboratory strain H37Rv. As expected, the ^1^H NMR signals of the four stereoisomeric DAT_2_ were mutually very similar and also similar to those of the authentic natural product. Small deviations, however, were observed in the chemical shifts of the hydrogens attached to C8–C15 and to C2, and C3 of the trehalose core. Hoye’s differential chemical shift analysis^[23,38]^ was used to depict the differences between each of the synthetic isomers and natural DAT_n_ and to determine which synthetic isomer would match closest to the natural sample (Figure 3, see also the ESI†). With respect to these signals, the ^1^H NMR chemical shifts of DAT_2_-b rather than the other 3 DAT_2_ isomers were in excellent agreement with those present in the natural sample. Although the differences were small, these results indicated that the stereochemistry of the 3-hydroxy-2-methyl unit of DAT_2_ corresponds to that of DAT_2_-b. We were however not fully convinced, if only because it was surprising that such a small difference in stereochemistry between the originally prepared DAT_2_-a and DAT_2_-b/natural DAT_2_ would lead to such a significant difference in retention time on HPLC, observed earlier.

### Determination of natural DAT_2_ by HPLC-MS/MS analysis

**Figure 4.**
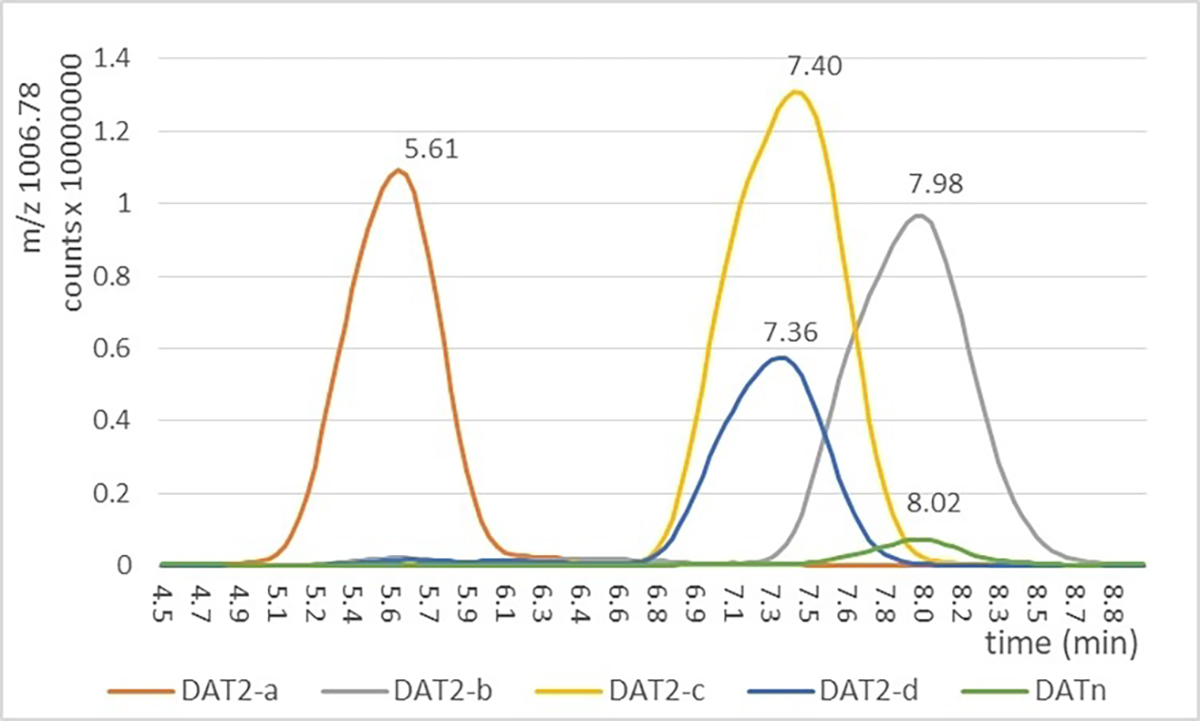
Comparison of the four synthetic isomers of DAT_2_ with the natural isolate DAT_n_ by HPLC.

The next comparison of the synthetic DAT_2_ isomers to natural DAT_n_ was by means of HPLC-MS/MS. Careful optimization of the solvent system used for reverse-phase chromatography on a C-18 column revealed that large differences in retention time could be obtained with a gradient of a 28:12:51:9 methanol:water:1-propanol:cyyclohexane mixture as the polar component and a 85:15 1-propanol:cyclohexane mixture as the nonpolar component. Both solvents contained 2.0 mM ammonium formate. The extracted-ion chromatograms (Figure 4) show a chromatographic match for DAT_2_-b (the grey line) and the natural isomer (the green line) while the other three synthetic DAT_2_ isomers show deviating retention times. In particular DAT_2_-a, possessing the structure proposed in literature, and initially prepared, showed a huge retention time difference with the natural isomer.

The mass of the natural isomer matched to those of the four DAT_2_ isomers, as expected. Collision-Induced Dissociation fragmentation of the natural and synthetic DAT_2_ isomers yielded interpretable fragmentations (see the ESI†) that supported the general structure and connectivity. Thus, we concluded that synthetic DAT_2_-b is identical to the natural isomer produced by the laboratory H37Rv strain, which matches with the result of the NMR comparison.

### Comparison of the mycolipanolic acids by GC-MS

**Figure 5.**
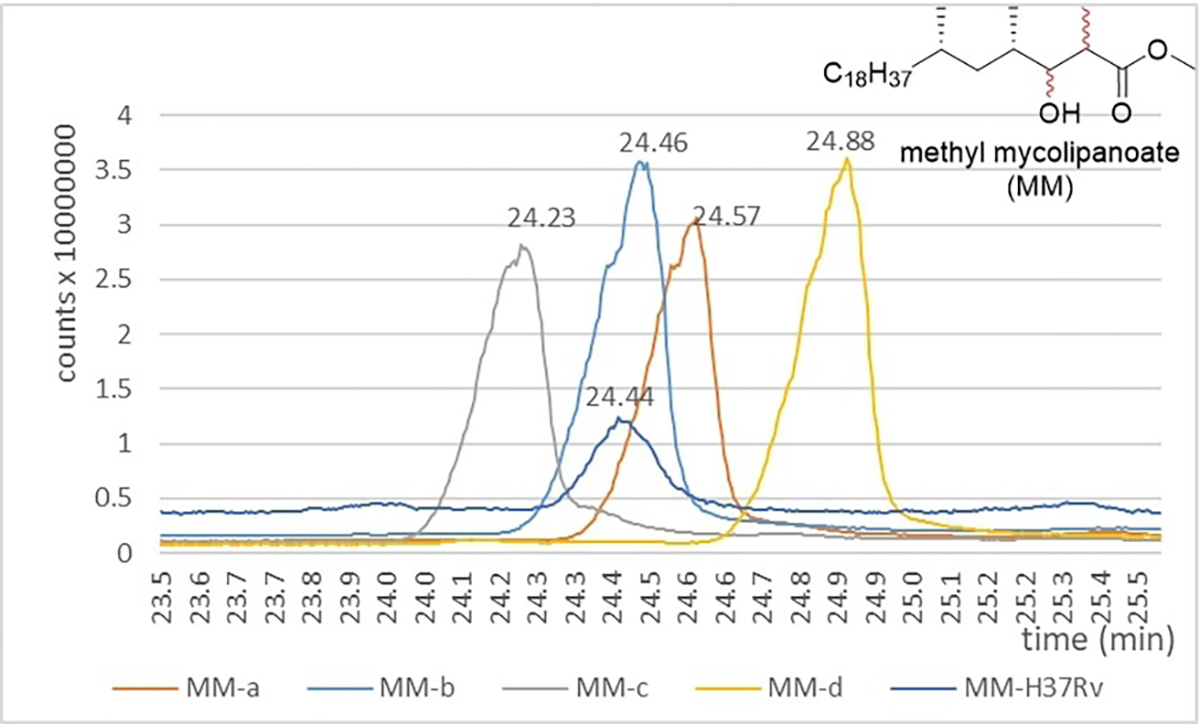
Comparison of retention times of the four isomeric methyl mycolipanoates with that of methyl mycolipanoate obtained from the natural isolate (*Mtb*-H37Rv) by GC chromatography on a polymethyl siloxane column.

To unequivocally determine the stereochemistry of natural mycolipanolic acid, a GC-MS analysis was carried out to confirm that acid **7b** from DAT_2_-b had the stereochemistry of natural mycolipanolic acid. After basic hydrolysis of a sample of the natural DAT_n_ extract, the four synthetic isomers of mycolipanolic acid, and the natural mycolipanolic acid were each converted into their corresponding methyl esters by treatment with trimethylsilyl diazomethane. GC-MS analysis (Figure 5) showed a chromatographic match of methyl mycolipanoate-b with the methyl mycolipanoate of H37Rv, which is fully consistent with the results of the NMR comparison and the HPLC-CID analysis. The retention time of the other isomers was sufficiently different. Peaks were identified by comparing the mass spectra ion fragments (see the ESI†).

Taken together, we established that the configuration of natural DAT_2_ is represented by the structure **DAT**_**2**_**-b** and that of mycolipanolic acid by **7b**. The stereochemistry of the α-methyl group was incorrectly assigned in the literature.^[20]^ This means that a number of studies provides an incorrect structure of mycolipanolic acid and DAT_2_.^[9,20–21,39]^

### Mincle activation by synthetic stereoisomers of DAT_2_

**Figure 6.**
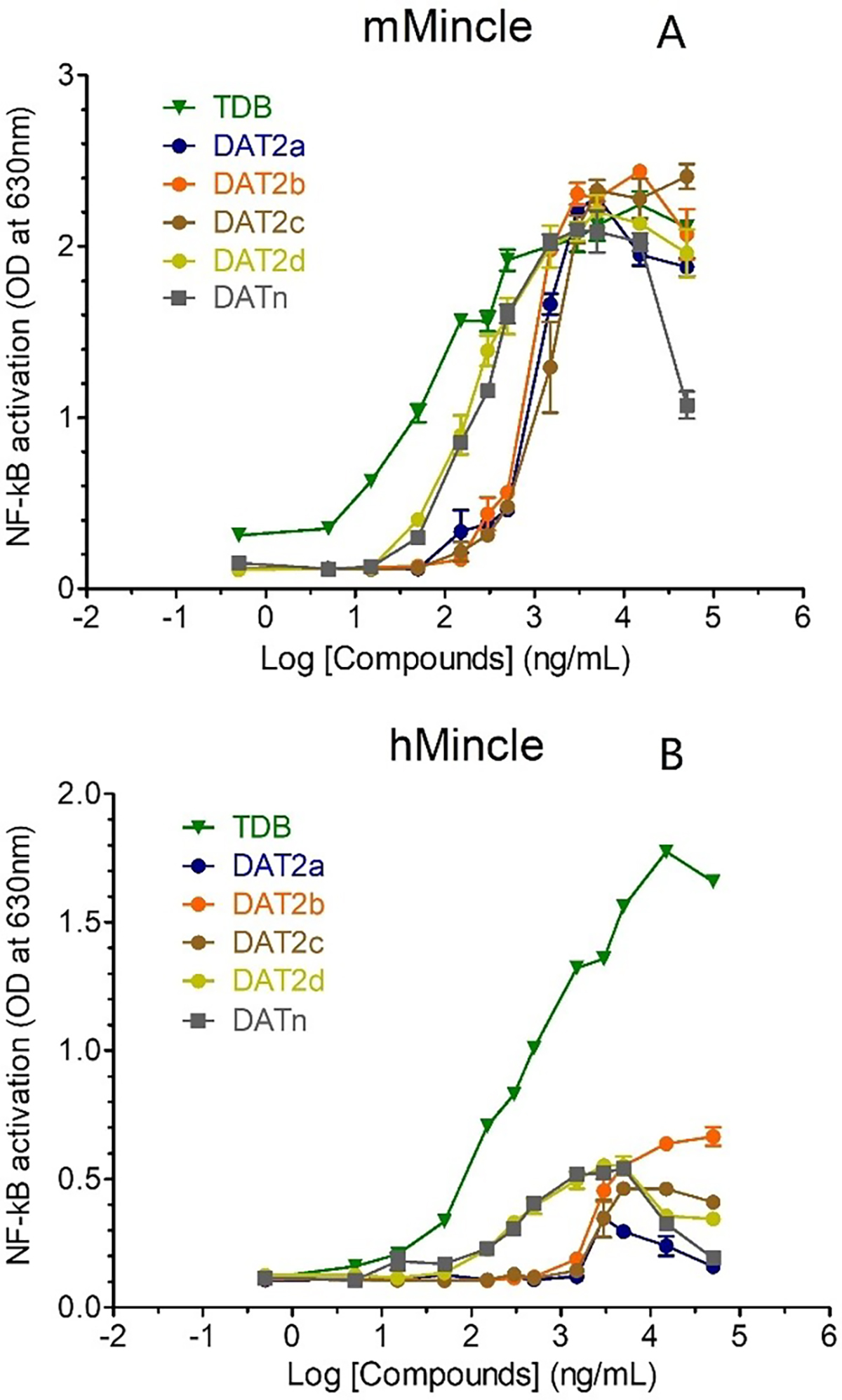
Mincle activation by synthetic DAT_2_ stereoisomers. HEK cells expressing murine (A) or human (B) Mincle and a NF-κB-inducible reporter system were stimulated with the indicated amount of DAT_2_ diastereomers, Mtb H37Rv natural DAT (DATn), or TDB. After 24 h, NF-κB activation was determined by reading OD at 630 nm.

We next investigated the capacity of the different stereoisomers, in comparison to the natural DAT family (DATn) from *M. tuberculosis* H37Rv, to induce Mincle activation using HEK cells expressing human Mincle (HEK-hMincle) or murine Mincle (HEK-mMincle), and an NF-κB-inducible reporter system (secreted alkaline phosphatase) (Figure 6). DATn induced NF-κB activation in a dose-dependent manner in both reporter cell lines, although as previously reported it was less potent (i.e. had a higher EC50) than trehalose-6,6′-dibehenate (TDB), a synthetic analog of TDM in which simpler fatty acids replace the complex mycolic acids.^[9]^ DATn showed an efficacy (i.e. a maximum response) similar to TDB in HEK-mMincle cells, but lower than TDB in HEK-hMincle cells. Of note, at high concentrations (>5 μg/ml), DATn showed toxicity.

Regarding the DAT_2_ isomers, DAT_2_-d showed the same potency as DATn in both reporter cell lines, whereas the other three DAT_2_ isomers were less potent. All the DAT_2_ isomers exhibited a similar efficacy, with the exception of DAT_2_-b, which was systematically more effective in HEK-hMincle cells.

## Conclusion

The asymmetric synthesis of four stereoisomers of mycolipanolic acid was achieved with full stereocontrol. It turned out that, with already two stereogenic methyl-substituents present in the substrate, different established methods for the preparation of aldol-type motives were required to prepare the various stereoisomers. A one-fits-all approach for the different syn- and anti-isomers could not be identified. For the syn-aldol products, the use of Evans chiral auxiliaries gave superior results. For the anti-aldol products, one was conveniently prepared using Abiko-Masamune’s chiral auxiliary, the other one using Brown’s crotylation reaction.

In this study, we accomplished the total synthesis of three diastereomers of mycobacterial DAT_2_ and mycolipanolic acid. Together with the already prepared isomer, four stereoisomers of DAT_2_ were compared with a natural sample from *Mtb* H37Rv, by NMR comparison and liquid chromatography-collision induced dissociation analysis. The corresponding synthetic and natural methyl mycolipanoates were analyzed through gas chromatography mass spectroscopy. Accordingly, the correct structure of natural DAT_2_ and the absolute configuration of natural mycolipanolic acid were determined to be DAT_2_-b and **7b** (2*R*, 3*R*, 4*S*, 6*S*). This means that the structure of mycolipanolic acid and of DAT_2_ has been re-assigned.

Immunological activity tests on mouse Mincle and human Mincle show that all four stereoisomers are potent activators, and natural DAT_2_/DAT_2_-b has the highest efficacy in HEK-hMincle cells. The previously proposed diastereomer DAT_2_-a is the least potent. DAT_2_ is highly antigenic and can potentially be applied in the development of serodiagnostic tests and vaccine adjuvants.

We found that small changes in the structure of the branched acyl chain in DAT result in large differences in recognition by Mincle. Considering the varying ratios of DAT_1_, DAT_2_, and DAT_3_ in natural “DAT”, and the possibility of contamination with minute amounts of oligosaccharides or peptides, means that the use of natural extracts of DAT to study immune response is not reliable. The availability of the DAT constituents by chemical synthesis enables the development of a serodiagnostic test and the use of these compounds as vaccine adjuvants.

## Supplementary Material

Supporting Information

## Data Availability

The data that support the findings of this study are available in the supplementary material of this article.
