# Supplementary material for: Asymmetric Total Synthesis and Structural Revision of DAT2, an Antigenic Glycolipid from Mycobacterium tuberculosis
Source: Angew Chem Int Ed Engl. Author manuscript; Available in PMC 2024 Oct 16. (PMC11482735; doi:10.1002/anie.202318582)
Supplement: Supporting Information [file NIHMS2025320-supplement-Supporting_Information.pdf]

Angewandte Chemie
Eine Zeitschrift der Gesellschaft Deutscher Chemiker

Supporting Information

Asymmetric Total Synthesis and Structural Revision of DAT_2, an Antigenic Glycolipid from *Mycobacterium tuberculosis*

*Z. Lin, J. P. Kaniraj, M. Holzheimer, J. Nigou, M. Gilleron, J. Hekelaar, A. J. Minnaard**

Asymmetric Total Synthesis and Structural Revision of DAT_2, an Antigenic Glycolipid from *Mycobacterium tuberculosis*

Zonghao Lin[a], Jeya Prathap Kaniraj[a], Mira Holzheimer[a], Jérôme Nigou[b], Martine Gilleron[b], Johan Hekelaar[a], and Adriaan J. Minnaard*,[a]

[a] M. Sc. Z. Lin, Dr. J. P. Kaniraj, Dr. M. Holzheimer, J. Hekelaar, Prof. Dr. A. J. Minnaard

Stratingh Institute for Chemistry, University of Groningen
Nijenborgh7, 9747 AG, Groningen (The Netherlands)
E-mail: a.j.minnaard@rug.nl

[b] Dr. M. Gilleron and Dr. J. Nigou
Institut de Pharmacologie et de Biologie Structurale, Université de Toulouse,
CNRS, UPS, 205 route de Narbonne, F-31077Toulouse (France)

Table of Contents

General methods and materials 2
Experimental Procedures and Characterization Data 3
Determination of natural DAT_2 by HPLC-CID analysis 19
Comparison of the mycolipanolic acids by GC-MS 23
Comparison of ^{1}H-NMR signals of the synthetic DAT_2 diastereomers with natural DAT_2 24
Mincle activation and binding assays 25
References 27
NMR Spectra 28
HRMS spectra 66

General methods and materials

All reactions were carried out under a nitrogen atmosphere using oven-dried glassware and using standard Schlenk techniques and anhydrous solvents unless otherwise mentioned. Reaction temperatures refer to the temperature of the heating mantle or cooling bath.
Anhydrous solvents (MTBE, DCM, THF, toluene) were taken from an MBraun solvent purification system (SPS-800). Other anhydrous solvents were purchased from Sigma-Aldrich, Acros and TCI Europe and used without further purification. Other reagents were purchased and used without further purification.
TLC analysis was performed on silica gel 60/Kieselguhr F254, 0.25 mm (Merck). Compounds were visualized using 254 nm UV light followed by p-anisaldehyde stain.
Flash chromatography was performed using silica gel type SiliaFlash P60 (230 – 400 mesh). The eluent composition is stated as v/v.
^{1}H and ^{13}C NMR spectra were recorded on an Agilent 400 NMR spectrometer at 400 and 100.59 MHz, respectively, using $CDCl_3$ or CD_3OD as the solvent. Chemical shifts are reported in ppm with the solvent resonance as the internal standard (for $CDCl_3$: δ 7.26 ppm for ^{1}H, δ 77.16 ppm for ^{13}C, CD_3OD: δ 3.31 ppm for ^{1}H, δ 49.00 ppm for ^{13}C). Data are reported as follows: chemical shifts (δ), multiplicity (s = singlet, d = doublet, dd = double doublet, ddd = double double doublet, ddp = double double pentet, td = triple doublet, t = triplet, q = quartet, b = broad, m = multiplet), coupling constant *J* (Hz), and integration value.
Enantiomeric excesses were determined by chiral HPLC analysis on a Shimadzu UFLC equipped with a diode-array detector. Integration at three different wavelengths (254, 220, 190 nm) was performed and the reported enantiomeric excess is an average of the three integrations. Retention times are reported in minutes.
High resolution mass spectra (HRMS) were recorded on a Thermo Scientific LTQ Orbitrap XL mass spectrometer with electrospray ionization (ESI) in positive or negative mode using DCM or methanol as solvent unless stated otherwise.

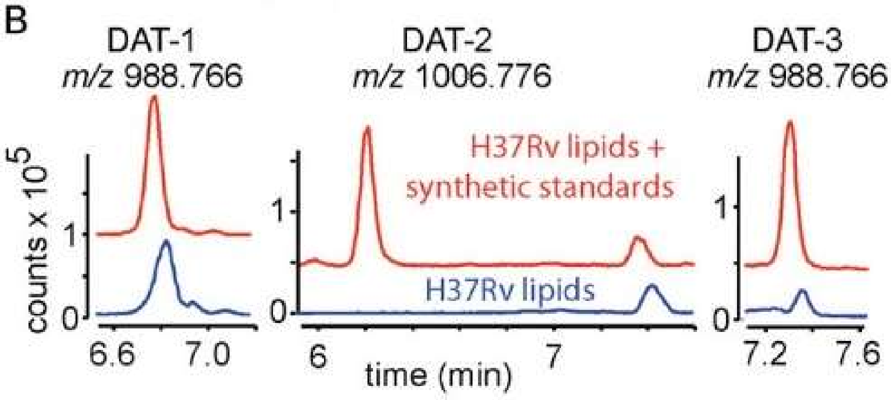

Figure S1. Figure reproduced from ref.[1]: comparison of synthetic standards of DAT_1, DAT_2, and DAT_3 with an extract of the reference *Mtb* strain H37Rv shows chromatographic coelution for DAT_1 and DAT_3 but not for DAT_2, indicating that synthetic DAT_2 is not identical to natural DAT_2

Experimental Procedures and Characterization Data

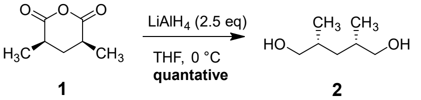

1 **2**

A round-bottom flask was loaded with **1**[2] (5.01 g, 35.2 mmol), followed by 100 mL of THF. At 0 °C, $LiAlH_4$ (3.34 g, 88.11 mmol) was added in four portions over five min. The reaction was allowed to warm to rt and stirred for 24 h. The reaction was cooled to 0 °C and quenched by slow addition of 400 mL of saturated Rochelle's Salt in 30 min. 300 mL of Et_2O was added and the mixture was left stirring overnight. 150 mL of water and 60 mL of dilute hydrochloric acid were added. The aqueous layer was extracted with ethyl acetate (3 × 100 mL), the combined organic layers were dried over anhydrous $MgSO_4$ and concentrated *in vacuo*. The *meso*-diol **2** was sufficiently pure to be used in the next step.

^{1}H NMR (400 MHz, $CDCl_3$) δ 3.45 (qd, J = 10.6, 5.7 Hz, 4H), 2.56(s, 2H)1.71 (dtd, J = 12.4, 6.8, 5.7 Hz, 2H), 1.55 (dt, J = 13.7, 6.8 Hz, 1H), 0.92 (d, J = 6.7 Hz, 6H), 0.90 – 0.85 (m, 1H).

^{13}C NMR (101 MHz, $CDCl_3$) δ 67.8, 37.0, 33.2, 17.8.

$[\alpha]_D^{23}$ = 0° (c = 0.2, $CHCl_3$).

The analytical data is in agreement with previous reports[2].

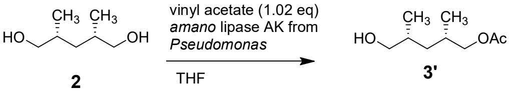

2 **3'**

A round-bottom flask was charged with 10.02 g (75.79 mmol) of *meso*-diol **2**, followed by 215 mL of dry THF, 7.2 mL vinyl acetate (77 mmol) and 0.50 g of amano lipase AK from *Pseudomonas fluorescence*. The reaction was left stirring at rt for 24 h, after which the reaction was filtered over celite, the filtrate was concentrated *in vacuo*, yielding the product **3'** (98% *ee* determined by compound **3''**), which was used for the next step without further purification.

^{1}H NMR (400 MHz, $CDCl_3$) δ 3.95 (dd, J = 10.8, 5.3 Hz, 1H), 3.83 (dd, J = 10.8, 6.8 Hz, 1H), 3.48 (dd, J = 10.6, 5.5 Hz, 1H), 3.39 (ddd, J = 10.5, 6.4, 0.8 Hz, 1H), 2.04 (s, 3H), 1.87 (dtd, J = 14.0, 6.9, 1.6 Hz, 1H), 1.76 – 1.65 (m, 1H), 1.42 (dt, J = 13.7, 6.8 Hz, 1H), 0.94 (ddd, J = 6.7, 3.6, 0.8 Hz, 6H).

^{13}C NMR (101 MHz, $CDCl_3$) δ 171.5, 69.3, 68.2, 37.4, 33.2, 30.2, 21.1, 18.0, 17.4.

HRMS calcd. for $[M + Na]^+$ 197.1148, found 197.1146. $[\alpha]_D^{23}$ = +7.6° (c = 0.21, $CHCl_3$).

The analytical data is in agreement with previous reports[2].

Determination of the enantiomeric excess:

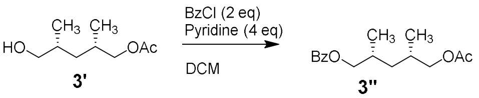

100 mg (0.57 mmol) of acetate **3'** was placed in a round-bottom flask. To this, 1 mL of dry DCM was added, followed by pyridine (0.37 mL, 2.3 mmol) and benzoyl chloride (133 µL, 1.15 mmol) at 0 °C. The ice bath was removed and the reaction was kept stirring. After 30 min, 108 µL (0.86 mmol) of 3-(dimethylamino)-1-propylamine was added to quench the reaction. After 30 min the mixture was transferred to a separatory funnel, diluted with 10 mL of DCM and washed with 5 mL of 1 M HCl, 5 mL of saturated $NaHCO_3$ and finally 5 mL of brine. The resulting DCM layer was dried over anhydrous $MgSO_4$, filtered and concentrated *in vacuo*, yielding the product quantitatively.

^{1}H NMR (400 MHz, $CDCl_3$) δ 8.11 – 7.99 (m, 2H), 7.62 – 7.52 (m, 1H), 7.49 – 7.40 (m, 2H), 4.20 (dd, J = 10.8, 5.5 Hz, 1H), 4.11 (dd, J = 10.8, 6.4 Hz, 1H), 3.96 (dd, J = 10.8, 5.7 Hz, 1H), 3.88 (dd, J = 10.8, 6.4 Hz, 1H), 2.12 – 2.04 (m, 1H), 2.02 (s, 3H), 1.99 – 1.89 (m, 1H), 1.55 (dt, J = 13.8, 6.8 Hz, 1H), 1.17 – 1.09 (m, 1H), 1.06 (d, J = 6.7 Hz, 3H), 0.99 (d, J = 6.7 Hz, 3H).

^{13}C NMR (101 MHz, $CDCl_3$) δ 171.4, 166.8, 133.0, 130.5, 129.7, 128.5, 69.5, 69.2, 37.8, 30.3, 30.2, 21.0, 18.0, 17.7.

HRMS Calcd. for $[M + H]^+$ 279.1591, found 279.1591. $[\alpha]_D^{23} = -6.0°$ (c = 0.2, $CHCl_3$).

The enantiomeric excess of 3'', and thereby of 3', was determined by chiral HPLC analysis using a Shimadzu LC- 10ADVP HPLC instrument equipped with a Shimadzu SPD-M10AVP diodearray detector. Chiralpak OD-H column, with heptane/i-PrOH: 98 : 2. Flow rate: 0.5 mL/min. UV detection at 254 nm.

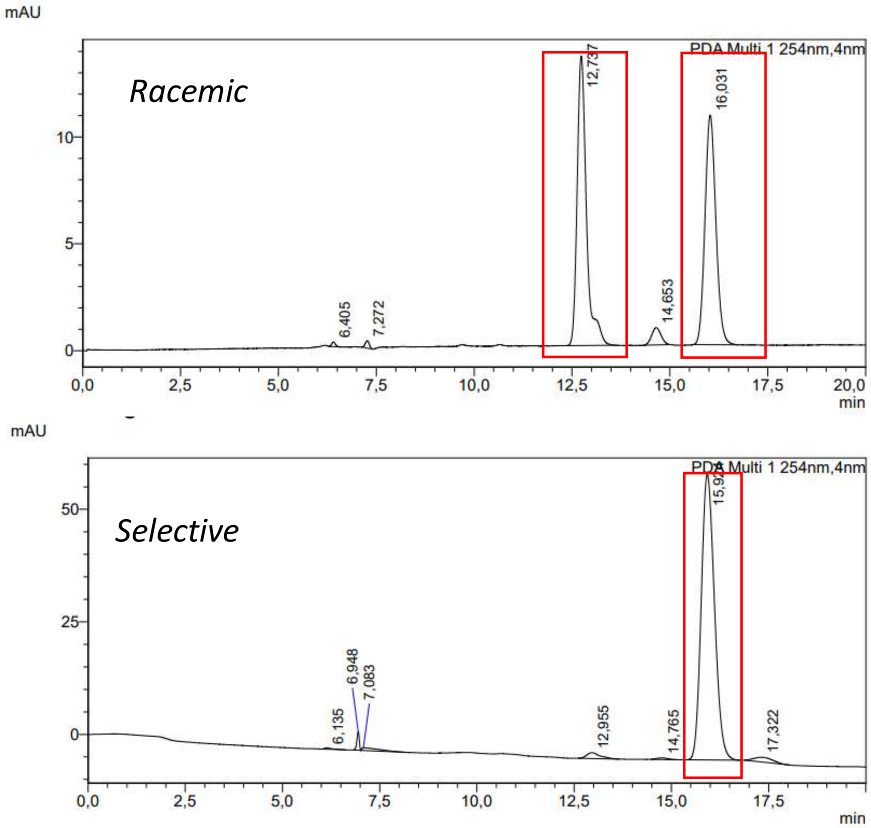

Fig. S2 Enantiomeric excess determination of 3".

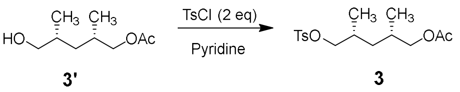

A dried round-bottom flask was loaded with 25 mL of dry pyridine, followed by 1.70 g (9.76 mmol) of **3’**. The reaction was placed in an ice bath and tosyl chloride (2.79 g, 14.6 mmol) was added in small portions. The ice bath was removed and the reaction was left stirring for 2 h. The reaction mixture was concentrated *in vacuo*, before transferred to a separatory funnel, 100 mL of ethyl acetate was added. The organic layer was washed five times with 50 mL saturated copper(II) sulfate solution. The organic layer was washed with sodium bicarbonate and with brine. The organic layer was dried over anhydrous $MgSO_4$ and concentrated *in vacuo*, yielding **3** (3.13 g, 97% over 3 steps).
^{1}H NMR (400 MHz, $CDCl_3$) δ 7.78 (d, J = 8.3 Hz, 2H), 7.35 (d, J = 7.8 Hz, 2H), 3.90 – 3.83 (m, 2H), 3.79 (ddd, J = 10.8, 6.4, 2.8 Hz, 2H), 2.45 (s, 3H), 2.04 (s, 3H), 1.95 – 1.83 (m, 1H), 1.82 – 1.72 (m, 1H), 1.39 (dt, J = 13.8, 6.9 Hz, 1H), 0.91 (dd, J = 8.0, 6.7 Hz, 6H).
^{13}C NMR (101 MHz, $CDCl_3$) δ 171.3, 144.9, 133.3, 130.04, 129.97, 128.0, 74.9, 69.0, 37.0, 30.5, 29.9, 21.8, 21.1, 17.7, 17.3.
HRMS (ESI+) Calcd. for $[M + Na]^+$ 351.1237, found 351.1230. $[\alpha]_D^{23}$ = -3.0° (c = 0.33, $CHCl_3$).
The analytical data is in agreement with previous reports[2].

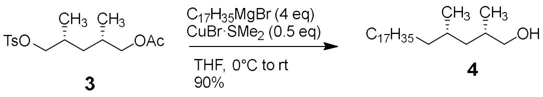

An oven-dried Schlenk flask was equipped with magnetic stirring and purged through three cycles of heating under vacuum and N_2 backfilling. Magnesium turnings (1.51 g, 62.1 mmol) were ground until polished and added to the flask under a flow of N_2, together with a crystal of iodine. The flask was heated gently until the purple iodine vapour filled the vessel. To the flask was then added dry THF (50 mL) which resulted in a green/brown suspension. In a separate (purged) flask, a solution of 1-bromoheptadecane (16.01 g, 50.13 mmol) in dry THF (40 mL + 10 mL over two washings) was prepared. This solution was slowly transferred to the reaction vessel over 8 min. The resulting reaction was stirred for 90 min to allow the Grignard reagent to form completely. Finally, copper(I) bromide dimethyl sulfide complex (1.24 g, 6.03 mmol) was added as a solid under a flow of N_2 causing the reaction to transition to a red-tinged black.

In a separate (purged) flask a solution of **3** (4.00 g, 12.2 mmol) in dry THF (30 mL + 10 mL over two washings) was prepared. The reaction vessel with the Grignard reagent was cooled to 0 °C and the solution of **3** was added *via* syringe over 13 min. Over this period, the reaction mixture changed to a very dark blue color. The reaction was stirred for 16 h, after which it had taken on a black colour with a faint blue/purple colour. This was taken as an indication that the reaction had completed. The mixture was quenched by the slow addition of cold saturated aqueous ammonium chloride (100 mL) which resulted in the formation of large amounts of a grey precipitate. The bi-layered system was separated. The aqueous phase was diluted with saturated aqueous Rochelle’s salt solution to redissolve the precipitate and was then extracted twice with diethyl ether. The combined organic phases were washed with a small amount of saturated aqueous Rochelle’s salt, dried over $MgSO_4$, and concentrated *in vacuo* to yield 13.68 g crude. The crude was loaded onto celite and purified using flash column chromatography (pentane : diethyl ether 99 : 1 to 95 : 5) to obtain the product as a light grey wax in 90% yield.

^{1}H NMR (400 MHz, $CDCl_3$) δ 3.52 (dd, J = 10.4, 5.1 Hz, 1H), 3.37 (dd, J = 10.4, 6.8 Hz, 1H), 1.72 (ddd, J = 7.4, 6.4, 5.0 Hz, 1H), 1.51 – 1.38 (m, 2H), 1.26 (s, 34H), 0.92 (d, J = 6.7 Hz, 3H), 0.88 (t, J = 6.5 Hz, 6H).

^{13}C NMR (101 MHz, $CDCl_3$) δ 68.6, 41.2, 36.8, 33.3, 32.1, 30.21, 30.18, 30.0, 29.86, 29.81, 29.5, 27.0, 22.8, 20.5, 17.5, 14.3.
HRMS calcd. for $[M + H]^+-H_2O$ 337.3829, found 337.3827. $[\alpha]_D^{23}$ = -3.3° (c = 0.02, $CHCl_3$).

The analytical data is in agreement with previous reports[3].

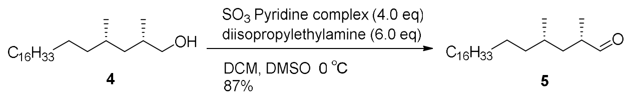

To a stirred solution of the alcohol **4** (0.87 g, 2.45 mmol) in dichloromethane (43 mL) and dimethylsulfoxide (23 mL), diisopropylethylamine (2.6 mL, 14.72 mmol) was added at 0 °C. SO_3·pyridine (1.56 g, 9.81 mmol) was dissolved in 20 ml DMSO and stirred at room temperature for 15 min and then added to the reaction mixture. The resulting solution was stirred for 45 min at 0 °C before saturated aqueous NH_4Cl (43 mL) was added. The separated aqueous layer was extracted with diethyl ether (3 × 120 mL). The combined organic layer was washed with phosphate buffer (pH 7), and brine, then dried over $MgSO_4$.Then it was filtered and concentrated in vacuo. The residue was purified by filtration through 20 mL silica (pentane/ether 98/2) to provide **5** (0.75 g, 87%) as a colorless wax.

^{1}H NMR (400 MHz, $CDCl_3$) δ 9.58 (d, *J* = 2.5 Hz, 1H), 2.43 (m, 1H), 1.71 (ddd, *J* = 13.9, 7.8, 6.1 Hz, 1H), 1.54 – 1.42 (m, 1H), 1.26 (s, 34H), 1.15 – 1.10 (m, 1H), 1.08 (d, *J* = 7.0 Hz, 3H), 0.88 (t, *J* = 7.1 Hz, 6H).

^{13}C NMR (101 MHz, $CDCl_3$) δ 205.5, 44.2, 38.3, 36.8, 31.9, 30.4, 29.9, 29.69, 29.68, 29.66, 29.4, 26.8, 22.7, 19.8, 14.2, 14.1.

The analytical data is in agreement with previous reports[3].

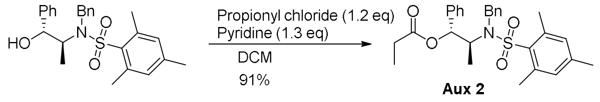

Propionyl chloride (0.58 g, 6.23 mmol) was added dropwise at 0 °C to a solution of the substrate (2.20 g, 5.19 mmol) and pyridine (550 µL, 6.75 mmol) in 30 mL dry DCM. The reaction was stirred at room temperature for 14 h and diluted with diethyl ether. The mixture was washed successively with water, 1 M HCl, water, saturated $NaHCO_3$ solution, and brine, and dried with anhydrous $MgSO_4$. After filtration, the solution was concentrated to give a crystalline residue **Aux 2** 2.27 g (91% yield).

^{1}H NMR (400 MHz, $CDCl_3$) δ 7.35 – 7.15 (m, 9H), 6.94 – 6.89 (m, 2H), 6.87 (s, 2H), 5.84 (d, *J* = 3.9 Hz, 1H), 4.75 – 4.53 (m, 2H), 4.04 (qd, *J* = 7.0, 4.0 Hz, 1H), 2.51 (s, 6H), 2.27 (s, 3H), 2.23 – 2.05 (m, 2H), 1.12 (d, *J* = 6.9 Hz, 3H), 1.01 (t, *J* = 7.5 Hz, 3H).

^{13}C NMR (101 MHz, $CDCl_3$) δ 172.6, 142.5, 140.2, 138.7, 138.6, 133.4, 132.1, 128.4, 128.4, 127.8, 127.3, 127.1, 125.9, 77.9, 56.7, 48.1, 27.5, 23.0, 20.9, 12.7, 8.8.

The analytical data is in agreement with previous reports[4].

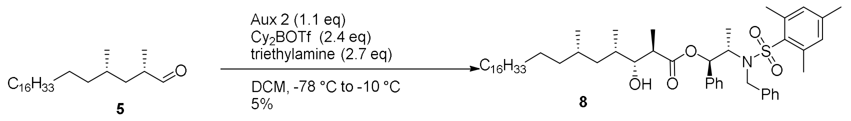

To a solution of **aux 2** (248.0 mg, 0.52 mmol) in DCM (5 mL) at -78 °C was added triethylamine (273 µL, 1.97 mmol), followed by a solution of dicyclohexylboron triflate (573 mg, 1.76 mmol) in DCM (1.5 mL) via syringe pump over 2 h. After addition, enol formation was allowed to take place by stirring for an additional 5 h at this temperature. Then, a solution of aldehyde **5** (200.6 mg, 0.57 mmol) in DCM (2 mL) was added slowly via syringe pump at a rate of 2 mL/h. After stirring for 1 hour at -78℃, the mixture was left stirring overnight while gradually warming to -10 °C. The reaction was quenched at -78 °C by addition of phosphate buffer (pH 7, 2 mL), MeOH (5 mL) and aqueous H_2O_2 (30%, 0.7 mL), allowed to reach room temperature, and stirred for another 24 h. Brine (10 ml) was added and the mixture was extracted with ether (4 × 4 mL). The combined organic layer was washed with brine (7 mL), dried over $MgSO_4$ and concentrated in vacuo yielding the crude product as a yellowish thick oil. The crude was dissolved in THF (2 mL), 2 M HCl aqueous solution (0.5 mL) was added and the mixture was stirred at room temperature for 1 h. Then the solvent was dried under nitrogen, 4 mL THF was added and neutralized by the addition of saturated aqueous $NaHCO_3$ (5 mL) and extracted with ether (3 × 4 mL). The combined organic layer was dried over $MgSO_4$ and concentrated in vacuo. The crude product was purified by flash column chromatography (10% EtOAc in pentane) to get the product **8** 22.0 mg (5% yield).

^{1}H NMR (400 MHz, $CDCl_3$) δ 7.37 – 7.29 (m, 2H), 7.25 – 7.14 (m, 6H), 6.89 (s, 2H), 6.86 – 6.80 (m, 2H), 5.81 (d, J = 4.0 Hz, 1H), 4.80 (d, 16.6 Hz, 1H), 4.57 (d, 16.6 Hz, 1H), 4.08 (qd, J = 7.0, 4.1 Hz, 1H), 3.63 (dd, J = 9.2, 2.5 Hz, 1H), 2.58 (m, 1H), 2.50 (s, 6H), 2.29 (s, 3H), 1.69 (qd, J = 7.0, 2.5 Hz, 1H), 1.39 (m, 1H), 1.25 (s, 35H), 1.15 (d, J = 7.0 Hz, 3H), 1.09 – 1.00 (m, 3H), 0.92 – 0.79 (m, 9H).

^{13}C NMR (101 MHz, $CDCl_3$) δ 175.3, 142.7, 140.4, 138.9, 138.4, 132.3, 128.54, 128.47, 128.0, 127.8, 127.3, 126.0, 78.3, 74.7, 56.9, 48.4, 43.7, 41.6, 37.2, 32.1, 31.2, 30.2, 29.89, 29.85, 29.81, 29.5, 27.1, 23.1, 22.8, 21.1, 20.1, 14.3, 14.0, 13.6, 12.9.

HRMS (ESI+) m/z calcd for $[M+NH_4]^+$ 849.6174; found 849.6176.

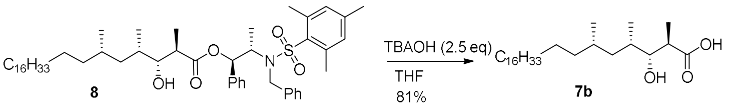

To a stirred solution of the ester **8** (240 mg, 0.289 mmol) in THF (3 mL, 0.1 M) a solution of aqueous NBu_4OH (1.5 M, 0.48 mL, 0.72 mmol) was added at 0 °C. The reaction was allowed to reach to room temperature and was left overnight. The reaction mixture was poured in an aqueous $KHSO_4$ solution (5%, 100 mL) and extracted with ether. The combined organic layer was washed with brine, dried over $MgSO_4$ and concentrated in vacuo. The crude residue was dissolved in acetonitrile and extracted with pentane and concentrated in vacuo, yielding the product **7b,** 100 mg (81%) in sufficient purity as a white solid.

^{1}H NMR (600 MHz, $CDCl_3$) δ 3.64 (dd, J = 8.6, 3.1 Hz, 1H), 2.70 – 2.59 (m, 1H), 1.74 (qt, J = 10.1, 5.0 Hz, 1H), 1.49 (tt, J = 9.6, 5.3 Hz, 1H), 1.45 – 1.38 (m, 1H), 1.25 (s, 35H), 1.19 (d, J = 7.1 Hz, 3H), 1.05 (m, 2H), 0.92 – 0.81 (m, 9H).

^{13}C NMR (151 MHz, $CDCl_3$) δ 181.0, 75.3, 43.3, 41.5, 37.0, 32.1, 31.8, 30.2, 30.0, 29.9, 29.5, 27.1, 22.9, 20.2, 14.3, 14.2, 13.1.

HRMS (ESI-) m/z calcd for $[M-H^+]$ 425.4000; found 425.3981. $[\alpha]_D^{23}$ = −5.0° (c = 0.010, $CHCl_3$).

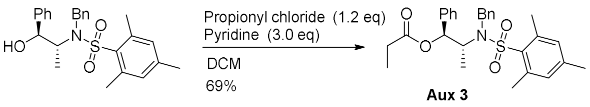

The substrate (7.20 g, 17.0 mmol) was dissolved in dry DCM (48 mL). Pyridine (4.03 g, 4.12 mL, 51.0 mmol) was added and the mixture was cooled to 0 °C. Propionyl chloride (1.89 g, 20.4 mmol) was added and left overnight while warming to room temperature. The mixture was diluted with DCM (50 mL) and washed with sat. aq. $NaHCO_3$ (50 mL). The aqueous layer was extracted with DCM and the combined organic layers were washed with 1 M HCl and, dried over $MgSO_4$, filtered and concentrated under reduced pressure. The crude yellow sticky oil was dissolved in a small amount of DCM while heating in a water bath. Upon cooling down, small crystals were observed, so cold heptane was added and the compound precipitated as small crystals. Filtering through a glass filter and rinsing with cold heptane yielded **Aux 3** 5.63 g, 69% of product as a white powder and 2.5 g of the mother liquid. This was not further crystallized.

^{1}H NMR (400 MHz, $CDCl_3$) δ 7.37 – 7.14 (m, 9H), 6.95 – 6.90 (m, 2H), 6.88 (s, 2H), 5.85 (d, J = 3.9 Hz, 1H), 4.77 – 4.57 (m, 2H), 4.05 (qd, J = 7.0, 3.9 Hz, 1H), 2.52 (s, 6H), 2.28 (s, 3H), 2.23 – 2.05 (m, 2H), 1.13 (d, J = 7.0 Hz, 3H), 1.02 (t, J = 7.5 Hz, 3H).

^{13}C NMR (101 MHz, $CDCl_3$) δ 172.7, 142.6, 140.3, 138.80, 138.75, 133.5, 132.3, 128.52, 128.50, 127.9, 127.5, 127.2, 126.0, 78.1, 56.9, 48.3, 27.6, 23.1, 21.0, 12.8, 8.9.

The analytical data is in agreement with previous reports[4].

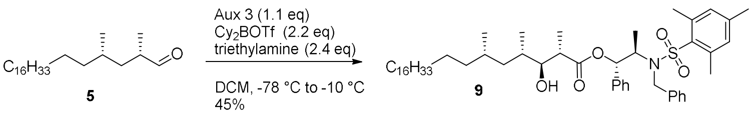

Auxiliary 3 (600 mg, 1.25 mmol) was dissolved in anhydrous DCM (15 mL) and Et_3N (304 mg, 418 µL, 3.00 mmol) was added. The reaction was allowed to cool down to -78 °C. A solution of dicyclohexyl (((trifluoromethyl)sulfonyl)oxy)borane (89 mg, 2.75 mmol) in 5 mL anhydrous DCM was added over 2 h. The reaction was left stirring for 2 h and a solution of aldehyde **5** (485 mg, 1.38 mmol) in anhydrous DCM (5 mL) was added over 1 h. After 20 min at -78 °C, the reaction was slowly warmed to -10 °C and left stirred overnight. The reaction was quenched with 7 mL of phosphate buffer solution, 18 mL of MeOH and 2.2 mL of H_2O_2 (35% in water) and stirred at room temperature for 1 h, after which the layers were separated. The aqueous layer was diluted with brine (50 mL) and extracted with EtOAc (3 × 20 mL). The combined organic layers were washed with brine, dried over Na_2SO_4, filtered and concentrated under reduced pressure. Column chromatography (0-5% EtOAc/heptane) gave product **9** 470 mg as a clear oil (45% yield).

^{1}H NMR (400 MHz, $CDCl_3$) δ 7.38 – 7.15 (m, 8H), 6.88 (s, 2H), 6.86 – 6.82 (m, 2H), 5.81 (d, *J* = 4.3 Hz, 1H), 4.69 (dd, *J* = 101.1, 16.5 Hz, 2H), 4.15 – 4.06 (m, 1H), 3.46 (dd, *J* = 7.4, 4.3 Hz, 1H), 2.68 (p, *J* = 7.2 Hz, 1H), 2.50 (s, 6H), 2.29 (s, 3H), 1.67 (ddd, *J* = 10.5, 6.8, 3.5 Hz, 1H), 1.47(s, 1H), 1.26 (s, 35H), 1.18 (d, *J* = 7.0 Hz, 3H), 1.11 (d, *J* = 7.1 Hz, 3H), 0.97 – 0.87 (m, 9H).

^{13}C NMR (101 MHz, $CDCl_3$) δ 175.2, 142.7, 140.4, 138.7, 138.3, 133.5,132.3, 128.5, 128.4, 128.1, 127.8, 127.3, 126.0, 78.3(C×2), 56.9, 48.4, 42.7, 37.9, 35.6, 32.9, 32.1, 30.22, 30.17, 29.91, 29.84, 29.79, 29.5, 26.9, 23.1, 22.8, 21.2, 21.0, 17.4, 14.6, 14.3, 13.7.

HRMS (ESI+) m/z calcd for $[M+Na]^+$ 854.5728; found 854.5723.

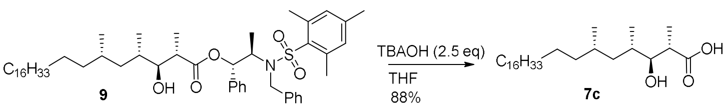

To a stirred solution of ester **9** (137.2 mg, 0.17 mmol) in THF (1.7 mL, 0.1 M) a solution of aqueous NBu_4OH (1.5 M, 275 µL, 0.41 mmol) was added at 0 °C. The reaction was allowed to reach room temperature and stirred overnight and then poured into an aqueous $KHSO_4$ solution (5%, 100 mL) and extracted with Et_2O. The combined organic layer was washed with brine, dried over $MgSO_4$ and concentrated in vacuo. The crude residue was dissolved in acetonitrile and extracted with pentane, yielding the product **7c** in sufficient purity as a white solid (61.6 mg, 88% yield).

^{1}H NMR (600 MHz, $CDCl_3$) δ 3.49 (dd, *J* = 6.9, 4.7 Hz, 1H), 2.71 (p, *J* = 7.1 Hz, 1H), 1.79 – 1.69 (m, 1H), 1.49 (dd, *J* = 12.1, 4.6 Hz, 1H), 1.25 (s, 39H),1.04(m, 1H), 0.96 (d, *J* = 6.7 Hz, 3H), 0.91 – 0.86 (m, 6H).

^{13}C NMR (151 MHz, $CDCl_3$) δ 180.7, 78.3, 42.3, 38.1, 35.5, 33.3, 31.9, 30.11, 30.07, 29.77, 29.72, 29.67, 29.4, 26.8, 22.7, 21.0, 17.0, 14.8, 14.1.

HRMS (ESI-) m/z calcd for $[M-H^+]$ 425.4000; found 425.3981. $[\alpha]_D^{23}$ = −28.6° (c = 0.010, $CHCl_3$).

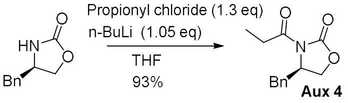

(R)-4-benzyloxazolidin-2-one (5.00 g, 28.22 mmol) was loaded to a vacuum-dried three-neck flask. This was dissolved in THF (74 mL) and cooled down to -78 °C. n-BuLi (1.90 g, 29.63 mmol) was added over 30 min using a syringe pump. The reaction mixture turned bright orange and was stirred for 30 min. Propionyl chloride (3.39 g, 3.2 mL, 36.68 mmol) was added dropwise using a syringe pump over 30 min and was stirred for 20 min. The bath was removed and the reaction mixture was stirred for 1.5 h. The mixture was slowly quenched with NH_4Cl and diluted with ethyl acetate. The organic layer was washed with $NaHCO_3$, H_2O, brine and dried over $MgSO_4$, filtered and concentrated under reduced pressure. A column-like filtration was performed by flushing first with heptane, then eluting with 5% ethyl acetate in heptane and finally with 20% ethyl acetate in heptane. The product **aux 4** (6.12 g, 26.2 mmol, 93%) was obtained as a transparent viscous oil.

^{1}H NMR (400 MHz, $CDCl_3$) δ 7.38 – 7.17 (m, 5H), 4.74 – 4.61 (m, 1H), 4.26 – 4.11 (m, 2H), 3.30 (dd, *J* = 13.4, 3.3 Hz, 1H), 3.08 – 2.86 (m, 2H), 2.77 (dd, *J* = 13.4, 9.6 Hz, 1H), 1.20 (td, *J* = 7.4, 0.9 Hz, 3H).

^{13}C NMR (101 MHz, $CDCl_3$) δ 174.2, 153.6, 135.5, 129.5, 129.1, 127.4, 66.3, 55.3, 38.0, 29.3, 8.4.

The analytical data is in agreement with previous reports[5].

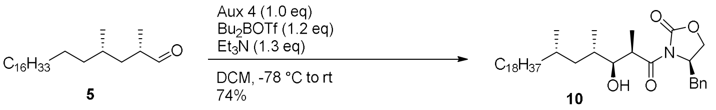

Aux 4 (590.0 mg, 2.53 mmol) was dissolved in anhydrous DCM (10 mL). The solution was cooled to 0 °C and dibutylboron trifluoride (818.1 mg, 3.0 mL, 1 M, 2.99 mmol) was added dropwise over 3 min. After stirring for 5 min, anhydrous Et_3N (332.7 mg, 458 µL, 3.29 mmol) was added. The mixture was stirred for another 45 min at 0 °C and then cooled down to -78 °C. Aldehyde **5** (981.1 mg, 2.78 mmol) was dissolved in anhydrous DCM (8 mL) (first in 4 mL, and then rinsed with 2 × 2 mL) and was added to the mixture dropwise. Upon addition of the aldehyde, a yellow suspension formed. The reaction was stirred for 1 h at -78 °C, then the cryostat was switched off and the mixture was allowed to slowly reach room temperature overnight. The reaction was quenched by addition of 38 mL aqueous 1 M KH_2PO_4 solution, followed by addition of 24 mL MeOH and 24 mL 3/2 MeOH/35% H_2O_2 mixture. The resulting mixture was stirred for 1 h at room temperature and then diluted with water (120 mL). The layers were separated and the aqueous layer was extracted with Et_2O. The combined organic layers were washed with saturated aqueous $NaHCO_3$ and brine, dried over Na_2SO_4, filtered and concentrated under reduced pressure. Column chromatography (0-14% Et_2O/heptane) yielded 74% of the product **10**.

^{1}H NMR (400 MHz, $CDCl_3$) δ 7.37 – 7.26 (m, 3H), 7.22 – 7.19 (m, 2H), 4.69 (ddt, *J* = 9.4, 6.9, 3.4 Hz, 1H), 4.25 – 4.14 (m, 2H), 3.98 (qd, *J* = 7.0, 2.9 Hz, 1H), 3.59 (dd, *J* = 7.9, 2.9 Hz, 1H), 3.26 (dd, *J* = 13.4, 3.4 Hz, 1H), 2.79 (dd, *J* = 13.4, 9.5 Hz, 1H), 1.64 (ddd, *J* = 15.3, 9.7, 3.1 Hz, 2H), 1.52 (dd, *J* = 6.5, 3.3 Hz, 1H), 1.26(s, 37H), 0.92 – 0.84 (m, 12H).

^{13}C NMR (101 MHz, $CDCl_3$) δ 177.9, 153.0, 135.2, 129.6, 129.1, 127.5, 76.2, 66.2, 55.3, 41.0, 40.0, 38.9, 35.8, 33.6, 32.1, 30.4, 30.2, 29.89, 29.83, 29.78, 29.5, 27.0, 22.8, 21.2, 16.1, 14.3, 10.4.

HRMS (ESI+) m/z calcd for $[M+Na]^+$ 608.4649; found 608.4642.

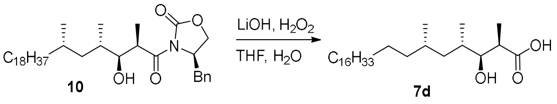

Hydrolysis of **10** was performed according to that of reported **6**.[1]

^{1}H NMR (600 MHz, $CDCl_3$) δ 3.67 (dd, *J* = 7.9, 3.5 Hz, 1H), 2.73 (qd, *J* = 7.1, 3.5 Hz, 1H), 1.69 – 1.56 (m, 1H), 1.56 – 1.46 (m, 1H), 1.25 (s, 37H), 1.04 – 0.92 (m, 2H), 0.91 – 0.83 (m, 9H).

^{13}C NMR (151 MHz, $CDCl_3$) δ 181.5, 76.4, 41.8, 40.7, 35.7, 33.6, 32.1, 30.4, 30.2, 29.90, 29.85, 29.81, 29.5, 27.0, 22.8, 21.2, 16.3, 14.3, 10.0.

HRMS (ESI-) m/z calcd for $[M-H^+]$ 425.4000; found 425.3982. $[\alpha]_D^{23} = -20.6°$ (c = 0.010, $CHCl_3$).

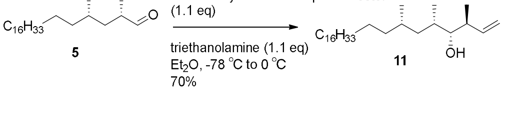

trans-Crotylboronic acid pinacol ester (96.2 mg, 0.53 mmol) in 0.3 mL of ether was cooled to -78 °C. The aldehyde **5** (169.0 mg, 0.48 mmol) in 0.3 mL ether was added and the mixture was allowed to - 30 °C for 3 h, the ice/acetone bath was removed and the reaction was left overnight. Triethanolamine (78.6 mg, 0.527 mmol) was added and the suspension was stirred for 2 h. The white suspension was filtered and the residue was rinsed with

ether, the filtrate was concentrated and purified by column chromatography (pentane/ether 99/1) to give **11** in 70% yield.

^{1}H NMR (400 MHz, $CDCl_3$) δ 5.74 (ddd, *J* = 17.0, 10.5, 8.6 Hz, 1H), 5.18 – 5.08 (m, 2H), 3.17 (dd, *J* = 8.2, 3.2 Hz, 1H), 2.28 (m, 1H), 1.74 (m, 1H), 1.48 – 1.36 (m, 1H), 1.36 – 1.27 (m, 2H), 1.26 (s, 32H), 1.05 (m, 2H), 0.98 (d, *J* = 6.8 Hz, 3H), 0.87 (dd, *J* = 6.9, 4.6 Hz, 9H).

^{13}C NMR (101 MHz, $CDCl_3$) δ 141.8, 116.4, 76.6, 42.3, 42.0, 37.0, 32.1, 31.6, 30.2, 30.0, 29.89, 29.87, 29.82, 29.5, 27.1, 22.8, 20.3, 16.8, 14.3, 13.3.

Ionization of this compound in ESI-mass failed, a GC-MS spectrum was successfully obtained (added at the last part of this document).

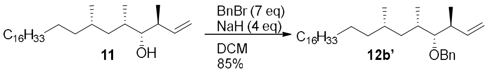

NaH (60% dispersion in oil, 15.7 mg, 0.39 mmol) was added into a stirred solution of the crotylation product **11** (40.0 mg, 0.10 mmol) in dry DCM (1.6 mL) under N_2 at 0 °C and stirred for 30 min. BnBr (81 µL, 0.69 mmol) was added dropwise and stirred for 1 h before warming to room temperature. The reaction was left overnight. The reaction mixture was quenched with H_2O (1.6 mL) and extracted with Et_2O, washed with brine and evaporated with toluene (2.5 mL). The crude material was purified by flash column chromatography (100% pentane) and gave 41.0 mg product **12b'** (85% yield).

^{1}H NMR (400 MHz, $CDCl_3$) δ 7.43 – 7.19 (m, 5H), 5.96 (ddd, *J* = 17.7, 10.3, 7.9 Hz, 1H), 5.14 – 4.97 (m, 2H), 4.62 (d, *J* = 11.1 Hz, 1H), 4.52 (d, *J* = 11.1 Hz, 1H), 3.06 (dd, *J* = 6.4, 4.2 Hz, 1H), 2.51 (h, *J* = 7.0 Hz, 1H), 1.81 (dtd, *J* = 7.7, 6.4, 4.1 Hz, 1H), 1.53 – 1.46 (m, 1H), 1.45 – 1.36 (m, 1H), 1.26 (d, *J* = 3.5 Hz, 32H), 1.05 (d, *J* = 6.9 Hz, 3H), 1.03 – 0.96 (m, 2H), 0.94 (d, *J* = 6.7 Hz, 3H), 0.90 – 0.84 (m, 6H).

^{13}C NMR (101 MHz, $CDCl_3$) δ 142.1, 139.4, 128.3, 127.7, 127.4, 114.2, 87.0, 74.8, 42.2, 41.2, 36.6, 33.2, 32.1, 30.21, 30.17, 29.89, 29.86, 29.82, 29.5, 27.0, 22.9, 20.6, 17.7, 15.1, 14.3.

HRMS (ESI+) m/z calcd for $[M+H]^+$ 499.4873; found 499.4854.

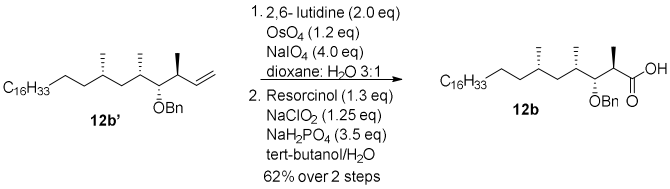

To a solution of benzylated crotylation product **12b'** (30.0 mg, 0.06 mmol) in dioxane-water (3:1, 0.6 mL, 0.1 M) were added 2,6-lutidine (14 µL, 0.12 mmol), OsO_4 (2.5% in 2-methyl-2-propanol, 0.07 mmol) and the mixture was left for 10 min. $NaIO_4$ (51.4 mg, 0.24 mmol) was added and the mixture was stirred at room temperature for 5 h. Water (1 mL) and CH_2Cl_2 (2 mL) were added. The organic layer was separated, and the water layer was extracted by CH_2Cl_2 (2 mL) three times. The combined organic layers were washed with brine and dried over Na_2SO_4. The solvent was removed, and the product was purified with silica gel column chromatography (pentane/ether 99/1-98/2) to afford corresponding aldehyde **12b''**.

^{1}H NMR (400 MHz, $CDCl_3$) δ 9.81 (d, *J* = 2.4 Hz, 1H), 7.38 – 7.27 (m, 5H), 4.59 (d, *J* = 11.1 Hz, 1H), 4.52 (d, *J* = 11.1 Hz, 1H), 3.53 (dd, *J* = 7.0, 3.6 Hz, 1H), 2.72 (pd, *J* = 7.0, 2.5 Hz, 1H), 1.87 (dtd, *J* = 8.0, 6.4, 3.5 Hz, 1H), 1.53 (d, *J* = 8.0 Hz, 1H), 1.42 (ddd, *J* = 13.4, 7.4, 6.0 Hz, 1H), 1.34 – 1.17 (m, 35H), 1.08 (d, *J* = 7.1 Hz, 3H), 0.97 (d, *J* = 6.8 Hz, 3H), 0.87 (d, *J* = 6.6 Hz, 6H).

^{13}C NMR (101 MHz, $CDCl_3$) δ 205.2, 138.5, 128.5, 127.8, 127.7, 84.2, 74.3, 49.3, 41.6, 36.5, 33.0, 32.1, 30.2, 29.87, 29.85, 29.81, 29.5, 27.0, 22.8, 20.5, 15.0, 14.3, 11.9.

The aldehyde **12b''** was dissolved in 0.1 mL of tert-butyl alcohol and resorcinol (1.4 mg, 0.05 mmol). A solution of $NaClO_2$ (1.1 mg, 0.05 mmol) and NaH_2PO_4 (27.4 mg, 0.18 mmol) in 0.1 mL tert-butyl alcohol/H_2O was added dropwise. The mixture was stirred at room temperature for 1.5 h. Volatile components were subsequently removed in vacuum, the residue was dissolved in 1 mL of water, the aqueous layer was acidified to pH 3 with

HCl and extracted with three 1 mL portions of Et_2O. The combined ether layers were washed with 1 mL of cold water, dried and concentrated to give an orange solid, after purification (DCM/MeOH 99/1-97/3), 19.2 mg acid **12b** was obtained (62% yield over 2 steps).

^{1}H NMR (400 MHz, $CDCl_3$) δ 7.37 – 7.27 (m, 5H), 4.61 (s, 2H), 3.55 (dd, *J* = 7.3, 3.2 Hz, 1H), 2.79 (p, *J* = 7.2 Hz, 1H), 1.85 (qd, *J* = 6.8, 3.0 Hz, 1H), 1.59 – 1.49 (m, 1H), 1.44 (dt, *J* = 13.5, 6.7 Hz, 1H), 1.25 (s, 33H), 1.21 (d, *J* = 7.2 Hz, 3H), 1.12 – 1.02 (m, 2H), 0.93 (d, *J* = 6.8 Hz, 3H), 0.88 (dd, *J* = 8.0, 6.3 Hz, 6H).

^{13}C NMR (101 MHz, $CDCl_3$) δ 178.8, 138.2, 128.5, 127.9, 127.8, 84.6, 75.1, 42.9, 41.5, 36.6, 32.7, 32.1, 30.2, 30.1, 29.88, 29.86, 29.8, 29.5, 27.0, 22.8, 20.4, 15.3, 14.6, 14.3.

HRMS (ESI+) m/z calcd for $[M+H]^+$ 517.4615; found 517.4614.

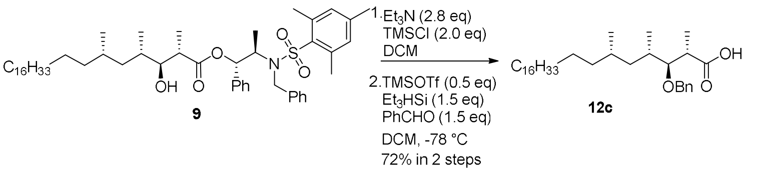

Compound **9** (177.1 mg, 0.21 mmol) was dissolved in dry DCM (2 mL). Triethylamine (60.3 mg, 83 µL, 0.60 mmol) and TMSCl (46.2 mg, 54 µL, 0.43 mmol) were added dropwise and the reaction was stirred overnight. The reaction was quenched with water, saturated aqueous $NaHCO_3$ was added and the aqueous layer was extracted with DCM. The combined organic layers were washed with saturated aqueous $NaHCO_3$, dried over Na_2SO_4, filtered and concentrated under reduced pressure. The crude NMR showed the desired product **12c'** and the orange oil (186 mg, 206 µmol, 97%) was used as such for the next step.

^{1}H NMR (400 MHz, $CDCl_3$) δ 7.43 – 7.35 (m, 2H), 7.33 – 7.21 (m, 5H), 7.19 – 7.05 (m, 3H), 6.86 (s, 2H), 6.78 (dt, *J* = 6.9, 1.3 Hz, 2H), 5.67 (d, *J* = 5.7 Hz, 1H), 4.87 (d, *J* = 16.2 Hz, 1H), 4.42 (d, *J* = 16.2 Hz, 1H), 4.11 – 4.00 (m, 1H), 3.64 (dd, *J* = 7.5, 3.9 Hz, 1H), 2.68 (p, *J* = 7.2 Hz, 1H), 2.42 (s, 6H), 2.30 (s, 3H), 1.60 (tt, *J* = 7.0, 3.5 Hz, 1H), 1.48 – 1.38 (m, 1H), 1.26 (s, 36H), 1.18 (d, *J* = 6.9 Hz, 3H), 0.95 (d, *J* = 7.1 Hz, 3H), 0.87 (dt, *J* = 13.7, 6.6 Hz, 9H), 0.09 (s, 9H).

^{13}C NMR (101 MHz, $CDCl_3$) δ 173.7, 142.5, 140.5, 138.7, 138.4, 133.2, 132.2, 128.5, 128.4, 128.3, 127.9, 127.4, 126.6, 79.3, 77.9, 56.9, 48.4, 44.4, 38.8, 35.6, 33.3, 32.1, 30.2, 30.1, 29.87, 29.83, 29.79, 29.5, 27.0, 23.0, 22.8, 21.2, 21.0, 17.2, 14.9, 14.3, 14.2, 1.0.

HRMS (ESI+) m/z calcd for $[M+Na]^+$ 926.6123; found 926.6122.

The compound **12c'** (185.0 mg, 0.21 mmol) was co-evaporated with toluene. Then it was dissolved in dry DCM (2.5 mL) and cooled to -78 °C. Benzaldehyde (32.6 mg, 31 µL, 30.1 mmol), triethylsilane (35.7 mg, 49 µL, 0.31 mmol) and TMSOTf (22.8 mg, 19 µL) were added and the reaction was stirred for 15 min. The bath was removed and stirring was continued for 2 h. The reaction was quenched with saturated aqueous $NaHCO_3$ and the aqueous layer was extracted with DCM. The combined organic layers were washed with $NaHCO_3$, dried over Na_2SO_4, filtered and concentrated under reduced pressure. The crude oil was purified by column chromatography (pentane/EtOAc 20/1) and obtained the product **12c** in 72% yield over 2 steps.

^{1}H NMR (400 MHz, $CDCl_3$) δ 7.46 – 7.27 (m, 5H), 4.67 (d, *J* = 11.1 Hz, 1H), 4.59 (d, *J* = 11.2 Hz, 1H), 3.48 (t, *J* = 5.0 Hz, 1H), 2.79 (qd, *J* = 7.2, 5.6 Hz, 1H), 2.00 – 1.83 (m, 1H), 1.50 – 1.38 (m, 1H), 1.26 (s, 38H), 1.15 – 1.04 (m, 2H), 0.97 (d, *J* = 6.9 Hz, 3H), 0.88 (t, *J* = 6.8 Hz, 6H).

^{13}C NMR (101 MHz, $CDCl_3$) δ 178.5, 137.8, 128.6, 128.05, 127.97, 86.0, 74.5, 42.0, 39.8, 36.0, 32.9, 32.1, 30.3, 30.2, 29.89, 29.86, 29.81, 29.5, 26.9, 22.8, 20.9, 16.4, 15.6, 14.3.

HRMS (ESI+) m/z calcd for $[M+Na]^+$ 539.4435; found 539.4436.

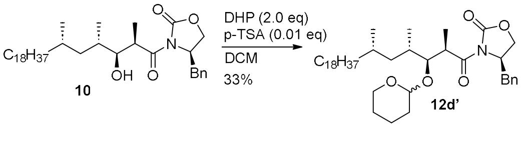

Compound **10** (116.0 mg, 0.20 mmol) was dissolved in dry DCM (1 mL) and DHP (33.3 mg, 36 µL, 0.40 mmol) and p-TSA (0.4 mg, 2 µmol) were added. The reaction was stirred for 4 h after which it was diluted with water. The aqueous layers were extracted with DCM and the combined organic layers were washed with brine, dried over Na_2SO_4, filtered and concentrated under reduced pressure. Column chromatography 0-8% EtOAc/heptane yielded the product **12d'** 76 mg (76%) as a mixture of diastereomers because of the THP protecting group .

^{1}H NMR (400 MHz, $CDCl_3$) δ 7.38 – 7.17 (m, 5H), 4.65-4.43 (m, 2H), 4.20 – 4.08 (m, 2H), 3.95 (qd, *J* = 7.0, 4.6 Hz, 1H), 3.89 – 3.71 (m, 2H), 3.51 – 3.22 (m, 2H), 2.76 (m, 1H), 1.75 (m, 3H), 1.59 – 1.40 (m, 5H), 1.27 (s, 37H), 1.00 – 0.79 (m, 12H).

^{13}C NMR (101 MHz, $CDCl_3$) δ 176.7, 175.4, 153.5, 153.0, 135.9, 135.4, 129.6, 129.6, 129.6, 129.1, 129.0, 127.5, 127.3, 101.7, 100.6, 83.6, 82.7, 66.3, 64.3, 56.4, 41.0, 40.5, 37.9, 37.9, 35.7, 34.4, 32.1, 31.1, 31.0, 30.36, 30.28, 30.2, 29.89, 29.87, 29.83, 29.79, 29.5, 27.0, 26.9, 25.6, 25.5, 22.8, 21.3, 20.8, 20.8, 20.2, 16.8, 16.2, 14.2, 13.8, 11.3.

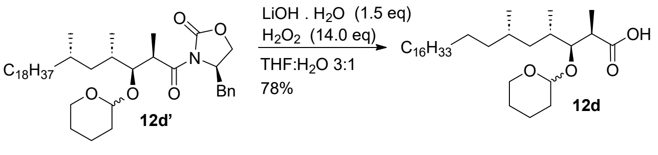

THP protect aldol product **12d'** (76.0 mg, 0.113 mmol) was dissolved in a mixture of THF and water (3:1) and cooled to 0 °C. Lithium hydroxide hydrate (7.1 mg, 0.17 mmol) and hydrogen peroxide (154.3 mg, 129 µL, 35% wt, 1.59 mmol) were added and the reaction was stirred overnight. The reaction was quenched with 10% $Na_2S_2O_3$ (20 mL) and the aqueous layer was extracted with EtOAc. The combined organic layers were dried over Na_2SO_4, filtered and concentrated under reduced pressure. The crude product was dissolved in acetonitrile and extracted with heptane to give compound **12d** (45 mg, 78%) as a clear oil.

^{1}H NMR (400 MHz, $CDCl_3$) δ 4.60 (m, 1H), 4.00 – 3.88 (m, 1H), 3.80 (m, 1H), 3.55 – 3.40 (m, 1H), 2.77 (m, 1H), 1.92 – 1.74 (m, 2H), 1.63 – 1.37 (m, 5H), 1.25 (s, 34H), 1.05 – 0.78 (m, 11H).
As the compound is a mixture of diastereomers due to the presence of the THP group, NMR analysis was thwarted and only the main signals of the H-NMR are provided.

Preparation of protected trehalose strategy 1

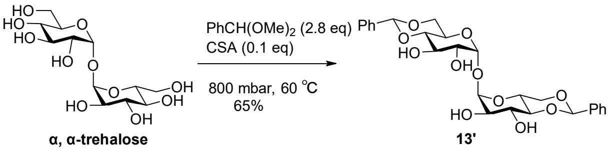

A flask was charged with α, α-trehalose (10.01 g, 0.03 mol) in 70 mL dry DMF. Camphorsulfonic acid (0.68 g, 2.93 mmol) was added as a solid followed by addition of $PhCH(OMe)_2$ (12.3 mL, 81.96 mmol). The flask was placed on a rotary evaporator at 60 ºC under reduced pressure (800 mbar). After 7 h, the remaining slurry was suspended in 150 mL 5% aq. $NaHCO_3$ and stirred overnight. The suspension was filtered and the residue was washed with water. The crude product was dissolved in EtOAc (500 mL) and washed with water (2 × 200 mL). The organic layer was dried over $MgSO_4$ and concentrated to give the product **13'** (9.89 g, 65% yield) as colorless solid.

^{1}H NMR (400 MHz, CD_3OD) δ 7.51 – 7.43 (m, 4H), 7.31 (dd, *J* = 5.0, 2.0 Hz, 6H), 5.55 (s, 2H), 5.10 (d, *J* = 3.9 Hz, 2H), 4.20 (dd, *J* = 10.0, 5.0 Hz, 2H), 4.09 (td, *J* = 9.9, 5.0 Hz, 2H), 4.01 (t, *J* = 9.4 Hz, 2H), 3.70 (t, *J* = 10.1 Hz, 2H), 3.60 (dd, *J* = 9.4, 3.9 Hz, 2H), 3.46 (t, *J* = 9.5 Hz, 2H).

^{13}C NMR (101 MHz, CD_3OD) δ 139.3, 130.0, 129.1, 127.7, 103.1, 96.5, 83.1, 73.8, 71.6, 70.0, 64.3.

The analytical data is in agreement with previous reports[6].

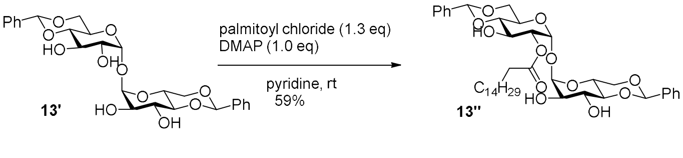

A flask was placed under N_2 atmosphere and charged with dibenzylidene trehalose **13'** (3.01 g, 5.81 mmol) and DMAP (0.71 g, 5.81 mmol) under nitrogen flow. The solids were dissolved in 10 mL dry pyridine. Palmitoyl chloride (2.3 mL, 7.55 mmol) was added dropwise at room temperature and the resulting mixture was stirred for 24 h. The reaction was quenched by addition of 40 mL water and the aqueous phase was extracted with EtOAc. The combined organic extracts were washed with brine (40 mL), dried over $MgSO_4$ and concentrated. The crude product was purified by flash chromatography (pentane/EtOAc 3/2 to 1/1) to give the product **13''** (2.57 g, 59% yield) as colorless wax.

^{1}H NMR (400 MHz, $CDCl_3$) δ 7.50 (dd, *J* = 6.8, 3.0 Hz, 2H), 7.45 (dd, *J* = 6.8, 3.0 Hz, 2H), 7.36 (dq, *J* = 6.6, 3.7 Hz, 6H), 5.54 (s, 1H), 5.46 (s, 1H), 5.35 (d, *J* = 3.8 Hz, 1H), 5.09 (d, *J* = 3.7 Hz, 1H), 4.88 (dd, *J* = 9.7, 3.8 Hz, 1H), 4.31 (dd, *J* = 10.3, 5.0 Hz, 1H), 4.25 (t, *J* = 9.6 Hz, 1H), 4.11 (tq, *J* = 9.3, 4.9, 4.2 Hz, 2H), 3.99 (t, *J* = 9.3 Hz, 1H), 3.85 – 3.55 (m, 5H), 3.49 – 3.42 (m, 1H), 2.42 (hept, *J* = 8.1, 7.7 Hz, 2H), 1.63 (q, *J* = 7.5 Hz, 3H), 1.25 (s, 24H), 0.88 (t, *J* = 6.8 Hz, 4H).

^{13}C NMR (101 MHz, $CDCl_3$) δ 173.4, 136.9, 136.8, 129.3, 129.3, 128.3, 128.3, 126.3, 126.2, 101.9, 101.8, 94.8, 92.4, 81.2, 80.7, 77.3, 77.0, 76.7, 72.9, 72.1, 71.1, 68.7, 68.6, 68.4, 63.2, 62.8, 34.0, 31.9, 29.68, 29.66, 29.64, 29.58, 29.56, 29.51, 29.4, 29.2, 29.1, 24.7, 22.7, 14.1.

The analytical data is in agreement with previous reports[7].

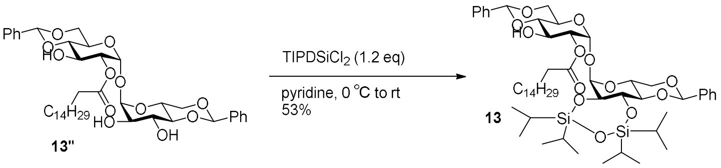

A flask was charged with palmitoyl trehalose **13''** (1.78 g, 2.35 mmol) and placed under N_2 atmosphere. The starting material was dissolved in 23 mL dry pyridine and cooled to 0 ºC. $TIPSCl_2$ (0.89 g, 2.82 mmol) was added at 0 ºC, then the reaction was stirred at room temparature for 3 d. The reaction mixture was poured onto ice water and the aqueous phase was extracted with EtOAc. The combined organic extracts were washed with brine. dried over $MgSO_4$ and concentrated. The crude product was purified by flash chromatography (pentane/EtOAc 10/1) and got 1.26 g product **13** (53% yield).

^{1}H NMR (400 MHz, $CDCl_3$) δ 7.50 – 7.41 (m, 4H), 7.35 (m, 6H), 5.53 (s, 2H), 5.37 (d, *J* = 3.8 Hz, 1H), 5.11 (d, *J* = 4.0 Hz, 1H), 4.87 (dd, *J* = 9.7, 3.8 Hz, 1H), 4.28 – 4.18 (m, 3H), 4.17 – 4.10 (m, 2H), 3.89 (dd, *J* = 8.5, 4.0 Hz, 1H), 3.82 (dd, *J* = 10.0, 4.7 Hz, 1H), 3.78 – 3.66 (m, 2H), 3.58 (t, *J* = 9.2 Hz, 1H), 3.52 (t, *J* = 9.3 Hz, 1H), 2.42 (m, 12H), 1.65 – 1.55 (m, 2H), 1.26 (s, 10H), 1.05 (s, 44H), 0.97 (m, 4H).

^{13}C NMR (101 MHz, $CDCl_3$) δ 173.6, 137.0, 129.3, 128.6, 128.3, 127.9, 126.4, 125.9, 102.3, 101.0, 94.4, 91.8, 81.4, 81.0, 75.1, 73.4, 73.0, 68.8, 68.7, 68.6, 62.8, 62.3, 60.4, 34.0, 31.9, 29.69, 29.67, 29.65, 29.54, 29.47, 29.36, 29.2, 29.0, 24.67, 22.68, 21.1, 17.42, 17.37, 17.31, 17.28, 17.15, 17.11, 17.05, 17.03, 14.17, 14.12, 13.0, 12.79, 12.71, 12.2, 11.7.

The analytical data is in agreement with previous reports[7].

Preparation of protected trehalose strategy 2

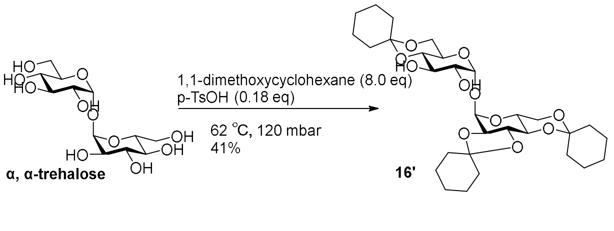

To a stirred suspension of anhydrous α, α-trehalose (4.02 g, 11.74 µmol) in anhydrous DMF (28 mL) under N_2, p-TSA (0.40 g, 2.10 mmol) and 1,1-dimethoxycyclohexane (13.50 g, 93.95 mmol) were added sequentially. The reaction mixture was heated to 65 °C at 120 mbar pressure on a rotary evaporator. After 2 h, the reaction mixture was cooled in an ice bath and poured into an ice-cold solution of 2 M NaOH (30 mL). The product was extracted in ethyl acetate, dried on $MgSO_4$, and concentrated under reduced pressure. Silica gel column chromatography of the crude product (pentane/EtOAc 3/2) furnished the product **16'** as white solid 2.82 g (41% yield).

^{1}H NMR (400 MHz, $CDCl_3$) δ 5.38 (d, *J* = 1.7 Hz, 1H), 5.12 (s, 1H), 4.14 – 4.04 (m, 2H), 3.97 – 3.82 (m, 4H), 3.82 – 3.60 (m, 6H), 3.56 – 3.45 (m, 2H), 3.20 (s, 1H), 2.86 (s, 1H), 2.35 – 2.20 (m, 1H), 2.19 – 2.09 (m, 1H), 1.99 – 1.70 (m, 5H), 1.70 – 1.19 (m, 30H).

^{13}C NMR (101 MHz, $CDCl_3$) δ 171.4, 112.5, 100.0, 99.8, 96.1, 94.0, 76.2, 73.3, 73.1, 72.7, 72.6, 71.8, 66.3, 64.0, 61.62, 61.58, 60.6, 37.9, 37.8, 36.3, 36.1, 27.9, 27.8, 25.7, 25.6, 25.1, 23.9, 23.6, 22.9, 22.7, 22.59, 22.55, 21.2, 14.3.

The analytical data is in agreement with previous reports[8].

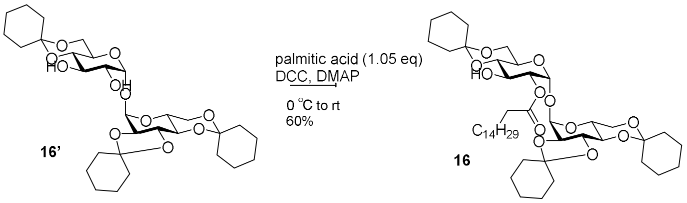

To the protected trehalose **16'** (1.00 g, 1.72 mmol) in 70 mL dry DCM containing 4 Å molecular sieves, was added palmitic acid (416.0 mg, 1.72 mmol) and DMAP (209.7 mg, 1.72 mmol). The solution was cooled to 0 ºC, and DCC (424.9 mg, 2.06 mmol) was added. After stirring for 15 min, the solution was allowed to warm to rt. After an additional 4.5 h, the white precipitate was removed by filtration and rinsed with DCM. The filtrate was concentrated and the resulting off-white solid was purified using column chromatography (pentane/EtOAc 9/1) to afford the product **16** as a white solid (832.0 mg, 60% yield).

^{1}H NMR (400 MHz, $CDCl_3$) δ 5.34 (d, *J* = 2.3 Hz, 1H), 5.29 (d, *J* = 3.9 Hz, 1H), 4.82 (dt, *J* = 9.6, 2.4 Hz, 1H), 4.11 (t, *J* = 9.3 Hz, 1H), 4.02 (dd, *J* = 10.5, 8.3 Hz, 1H), 3.99 – 3.91 (m, 2H), 3.85 (dd, *J* = 10.1, 5.5 Hz, 1H), 3.81 – 3.68 (m, 3H), 3.60 (dq, *J* = 10.4, 5.5, 5.0 Hz, 2H), 3.49 (dq, *J* = 8.4, 3.6, 2.9 Hz, 1H), 2.50 – 2.37 (m, 2H), 2.17 (d, *J* = 1.6 Hz, 1H), 1.93 (q, *J* = 5.8 Hz, 2H), 1.77 (m, 2H), 1.72 – 1.39 (m, 28H), 1.25 (s, 27H), 0.92 – 0.83 (m, 3H).

^{13}C NMR (101 MHz, $CDCl_3$) δ 173.6, 112.6, 100.05, 99.97, 94.0, 93.5, 76.2, 73.5, 73.11, 73.09, 69.3, 66.4, 63.9, 61.6, 38.1, 38.0, 36.4, 36.2, 34.2, 32.1, 31.1, 29.85, 29.83, 29.81, 29.80, 29.73, 29.67, 29.5, 29.4, 29.3, 27.98, 27.94, 25.7, 25.2, 25.0, 24.0, 23.7, 23.0, 22.8, 22.7, 22.6, 14.3.

The analytical data is in agreement with previous reports[8].

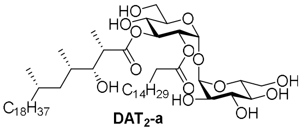

We reported the synthesis of **DAT_2-a** in 2020[1]. $[\alpha]_D^{23}$ = +55.6° (c = 0.005, $CHCl_3$).

^{1}H NMR (600 MHz, $CDCl_3/CD_3OD$) δ 5.30 (dd, *J* = 10.2, 9.2 Hz, 1H), 5.14 (d, *J* = 3.6 Hz, 1H), 4.96 (d, *J* = 3.7 Hz, 1H), 4.76 (dd, *J* = 10.3, 3.6 Hz, 1H), 3.82 (ddd, *J* = 10.0, 5.5, 2.6 Hz, 1H), 3.70 (dt, *J* = 12.0, 2.5 Hz, 1H), 3.65 (t, *J* = 9.4 Hz, 1H), 3.61 – 3.52 (m, 3H), 3.50 – 3.43 (m, 2H), 3.43 – 3.38 (m, 2H), 3.29 (t, *J* = 9.4 Hz, 1H), 2.55 (qd, *J* = 7.0, 5.3 Hz, 1H), 2.19 – 2.10 (m, 2H), 1.49 (dq, *J* = 8.8, 6.5 Hz, 1H), 1.40 (q, *J* = 7.4 Hz, 2H), 1.11 (s, 62H), 1.03 (d, *J* = 7.0 Hz, 3H), 0.84 (m, 1H), 0.77 (d, *J* = 6.6 Hz, 3H), 0.73 (td, *J* = 6.7, 2.1 Hz, 9H).

^{13}C NMR (151 MHz, $CDCl_3/CD_3OD$ 4/1) δ 176.2, 173.7, 94.6, 91.8, 75.3, 73.4, 72.74, 72.68, 72.5, 71.8, 70.7, 70.2, 69.3, 61.6, 61.4, 43.2, 41.2, 36.4, 34.0, 33.2, 32.1, 30.2, 29.94, 29.92, 29.88, 29.86, 29.85, 29.82, 29.80, 29.7, 29.5, 29.3, 27.0, 24.8, 22.8, 20.4, 15.1, 14.1, 11.3.

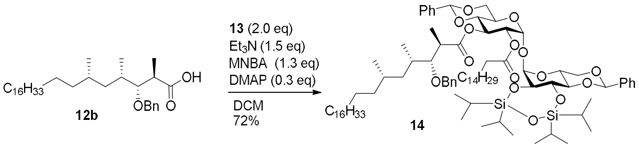

To a solution of the benzyl protected acid **12b** (20.0 mg, 0.04 mmol) in dry DCM (0.4 mL) was added Et_3N (8 μL, 0.06 mmol), DMAP (1.4 mg, 0.01 mmol) and 2-methyl-6-nitrobenzoic anhydride (17.3 mg, 0.05 mmol). This was stirred at room temperature for 10 min, after which the protected trehalose **13** (77.3 mg, 0.08 mmol) in dry DCM (0.4 mL) was added and the reaction was stirred overnight. The reaction was quenched with saturated aqueous NH_4Cl, water was added and the aqueous layer was extracted with DCM. The combined organic layers were washed with water, dried over Na_2SO_4, filtered and concentrated under reduced pressure. The crude product was purified by column chromatography (2%-4% EtOAc/pentane) and provided 42 mg product **14** (72% yield).

^{1}H NMR (400 MHz, $CDCl_3$) δ 7.46 (m, 2H), 7.38 – 7.27 (m, 5H), 7.23 – 7.10 (m, 6H), 7.04 – 6.96 (m, 2H), 5.67 (d, *J* = 9.8 Hz, 1H), 5.53 (s, 1H), 5.40 (d, *J* = 3.8 Hz, 1H), 5.25 (s, 1H), 5.13 (d, *J* = 4.1 Hz, 1H), 5.03 (dd, *J* = 9.9, 3.8 Hz, 1H), 4.46 (d, *J* = 11.7 Hz, 1H), 4.29 (dd, *J* = 9.8, 4.9 Hz, 1H), 4.19 (t, *J* = 10.0 Hz, 3H), 4.15 – 4.11 (m, 1H), 3.92 (dd, *J* = 8.4, 4.1 Hz, 1H), 3.80 (td, *J* = 9.9, 4.5 Hz, 1H), 3.68 (td, *J* = 10.0, 5.3 Hz, 3H), 3.58 (dd, *J* = 8.7, 2.6 Hz, 1H), 3.52 (t, *J* = 9.2 Hz, 1H), 2.74 (p, *J* = 7.2 Hz, 1H), 2.29 (dt, *J* = 12.5, 8.0 Hz, 2H), 1.78 – 1.66 (m, 1H), 1.54 (d, *J* = 6.9 Hz, 3H), 1.26 (s, 52H), 1.16 – 1.10 (m, 15H), 1.09 – 1.02 (m, 18H), 1.03 – 0.98 (m, 3H), 0.88 (t, *J* = 6.6 Hz, 9H), 0.79 (d, *J* = 6.7 Hz, 3H), 0.73 (d, *J* = 6.4 Hz, 3H).

^{13}C NMR (101 MHz, $CDCl_3$) δ 175.5, 173.4, 139.2, 137.8, 137.0, 129.0, 128.7, 128.1, 128.1, 128.0, 127.6, 127.1, 126.4, 126.1, 101.9, 101.3, 94.5, 91.9, 82.7, 81.2, 79.4, 75.5, 73.8, 73.7, 71.5, 69.4, 68.9, 63.0, 62.7, 43.5, 41.5, 37.0, 34.1, 32.1, 31.7, 30.2, 29.87, 29.83, 29.82, 29.7, 29.6, 29.53, 29.52, 29.3, 29.2, 27.0, 24.8, 22.8, 20.0, 17.57, 17.56, 17.5, 17.35, 17.33, 17.31, 17.28, 17.15, 14.8, 14.3, 14.1, 13.1, 12.8, 12.4, 11.9.

HRMS (ESI+) m/z calcd for $[M+Na]^+$ 1519.9936; found 1519.9931.

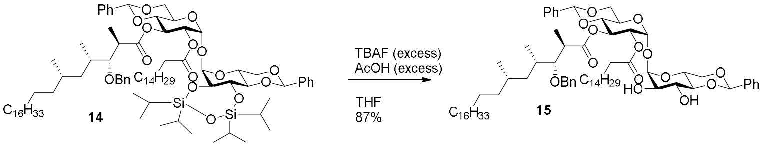

Protected diacyl trehalose **14** (13.0 mg, 0.01 mmol) was dissolved in 0.1 mL THF. To this, TBAF (1 M in THF, 0.34 mL, 0.34 mmol) and glacial AcOH (19 μL, 0.34 mmol) were added to the stirred solution at room temperature. After 2 h, TLC showed the reaction was complete. The reaction was diluted with EtOAc (4 mL) and then washed with water (1.5 mL) and brine (1.5 mL), dried over $MgSO_4$ and concentrated. The crude product was purified by flash column chromatography (pentane/EtOAc 3/1 to 3/2) giving the product **15** (9.5 mg, 87%) as colorless wax.

^{1}H NMR (400 MHz, $CDCl_3$) δ 7.46 (dt, *J* = 6.3, 3.7 Hz, 2H), 7.40 – 7.29 (m, 5H), 7.16 (qt, *J* = 9.0, 4.5 Hz, 6H), 7.06 (dt, *J* = 7.4, 3.7 Hz, 2H), 5.67 (t, *J* = 9.8 Hz, 1H), 5.51 (s, 1H), 5.41 (d, *J* = 3.9 Hz, 1H), 5.28 (s, 1H), 5.17 (d, *J* = 3.8 Hz, 1H), 5.06 (dd, *J* = 10.0, 3.9 Hz, 1H), 4.53 (d, *J* = 11.8 Hz, 1H), 4.28 (q, *J* = 4.9 Hz, 1H), 4.19 (q, *J* = 4.9 Hz, 1H), 4.13 (dd, *J* = 10.3, 4.8 Hz, 1H), 4.07 (d, *J* = 9.3 Hz, 1H), 3.80 (td, *J* = 9.9, 4.5 Hz, 1H), 3.70 (qd, *J* = 9.3, 3.1 Hz, 4H), 3.59 (dd, *J* = 8.6, 2.7 Hz, 1H), 3.52 (d, *J* = 9.3 Hz, 1H), 3.48 (s, 6H), 2.79 (p, *J* = 7.3 Hz, 1H), 2.41 – 2.24 (m, 2H), 1.75 (qd, *J* = 6.9, 2.5 Hz, 2H), 1.57 (q, *J* = 7.4 Hz, 3H), 1.44 – 1.36 (m, 2H), 1.33 – 1.16 (m, 52H), 1.09 (d, *J* = 7.1 Hz, 3H), 0.88 (t, *J* = 6.6 Hz, 6H), 0.82 (d, *J* = 6.7 Hz, 3H), 0.76 (d, *J* = 6.4 Hz, 3H).

^{13}C NMR (101 MHz, $CDCl_3$) δ 175.6, 173.2, 139.2, 137.1, 136.8, 129.4, 129.0, 128.4, 128.2, 128.1, 127.4, 127.1, 126.4, 126.2, 102.1, 101.7, 94.9, 92.5, 83.0, 81.0, 79.3, 74.0, 72.4, 71.4, 71.3, 69.3, 68.8, 63.5, 63.3, 51.0, 43.5, 41.6, 37.0, 34.1, 32.1, 31.9, 30.2, 30.0, 29.89, 29.86, 29.84, 29.82, 29.81, 29.7, 29.6, 29.52, 29.51, 29.4, 29.3, 27.1, 24.8, 22.8, 20.1, 14.9, 14.3, 14.1.

HRMS (ESI+) m/z calcd for $[M+NH_4]^+$ 1272.8860; found 1272.8886.

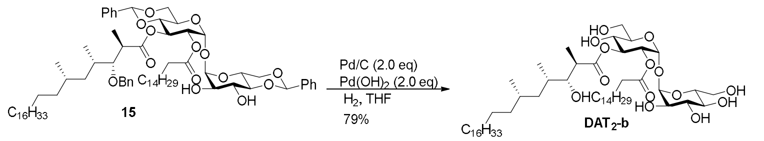

A flask was charged with protected diacyl trehalose **15** (23.0 mg, 0.02 mmol), Pd/C (10% Pd by weight, 39.0 mg, 0.04 mmol) and $Pd(OH)_2$ (20% Pd by weight, 25.7 mg, 0.04 mmol). Then 1 mL THF was added, the resulting suspension was placed under H_2 atmosphere and stirred at room temperature for 4 days. The reaction mixture was filtered over celite using MeOH to fully elute the product and then purified by flash column chromatography (CH_2Cl_2/acetone/MeOH 8/1/1). The product **DAT$_2$-b** was obtained as a colorless oil (14.3 mg, 79% yield).

^{1}H NMR (600 MHz, $CDCl_3/CD_3OD$ 4/1) δ 5.30 (dd, J = 10.2, 9.2 Hz, 1H), 5.14 (d, J = 3.6 Hz, 1H), 4.97 (d, J = 3.7 Hz, 1H), 4.76 (dd, J = 10.3, 3.6 Hz, 1H), 3.85 (dq, J = 8.1, 2.8 Hz, 1H), 3.72 (dd, J = 12.1, 2.6 Hz, 1H), 3.66 (t, J = 9.4 Hz, 1H), 3.61 – 3.54 (m, 3H), 3.51 – 3.44 (m, 3H), 3.40 (dd, J = 9.8, 3.7 Hz, 1H), 3.30 (t, J = 9.4 Hz, 1H), 2.50 (dq, J = 9.7, 7.0 Hz, 1H), 2.15 (q, J = 7.5 Hz, 2H), 1.60 (qd, J = 7.0, 2.2 Hz, 1H), 1.41 (t, J = 7.3 Hz, 2H), 1.36 (dd, J = 8.7, 5.1 Hz, 1H), 1.25 (dd, J = 13.7, 6.9 Hz, 1H), 1.11 (m, 60H), 0.91 (d, J = 7.0 Hz, 3H), 0.76 – 0.71 (m, 9H), 0.69 (d, J = 6.8 Hz, 3H).

^{13}C NMR (151 MHz, $CDCl_3/CD_3OD$ 4/1) δ 176.7, 173.6, 94.7, 91.9, 75.4, 73.4, 73.1, 72.7, 72.2, 71.8, 70.7, 70.2, 69.0, 61.6, 61.5, 44.2, 41.8, 37.1, 34.0, 32.1, 31.1, 29.95, 29.89, 29.85, 29.83, 29.79, 29.78, 29.63, 29.50, 29.46, 29.3, 27.1, 24.8, 22.8, 20.0, 14.2, 14.1, 12.4.

HRMS (ESI+) m/z calcd for $[M+NH_4]^+$ 1006.7764; found 1006.7780. $[\alpha]_D^{23}$ = +26.0° (c = 0.005, $CHCl_3$).

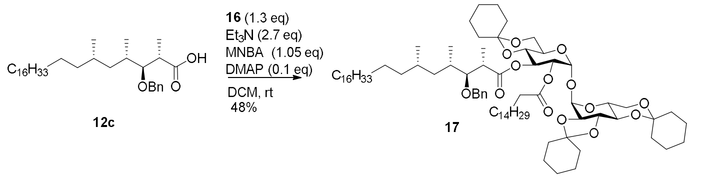

To a solution of compound **12c** (27.0 mg, 0.05 mmol) in dry DCM (0.5 mL) was added Et_3N (14.3 mg, 20 μL, 0.14 mmol), DMAP (0.7 mg, 5 μmol) and 2-methyl-6-nitrobenzoic anhydride (19.8 mg, 0.06 mmol). The reaction mixture was stirred for 10 min, after which cyclohexylidene trehalose **16** (54.8 mg, 0.07 mmol) in dry DCM (0.5 mL) was added. The reaction was stirred at room temperature overnight.

The reaction mixture was quenched with saturated aqueous NH_4Cl, water was added and the aqueous layer was extracted with DCM. The combined organic layers were washed with water, dried over Na_2SO_4, filtered and concentrated under reduced pressure. Column chromatography (10% EtOAc/pentane) gave the product **17** as a clear oil (33.0 mg, 48%).

^{1}H NMR (400 MHz, $CDCl_3$) δ 7.37 – 7.27 (m, 5H), 5.45 (dt, J = 14.8, 9.8 Hz, 1H), 5.36 (d, J = 3.0 Hz, 1H), 5.31 (dd, J = 5.4, 3.8 Hz, 1H), 4.95 (dd, 1H), 4.71 – 4.45 (m, 2H), 4.21 (t, J = 9.4 Hz, 1H), 4.07 (td, J = 10.2, 5.3 Hz, 1H), 3.96 (td, J = 8.8, 8.3, 4.1 Hz, 1H), 3.90 – 3.64 (m, 6H), 3.63 – 3.48 (m, 4H), 2.79 – 2.70 (m, 1H), 2.34 (t, J = 6.7 Hz, 1H), 2.15 (d, J = 13.2 Hz, 1H), 2.04 (ddd, J = 9.1, 4.6, 2.8 Hz, 1H), 1.91 – 1.84 (m, 3H), 1.80 (tt, J = 6.7, 3.8 Hz, 2H), 1.67 (t, J = 6.4 Hz, 7H), 1.54 – 1.45 (m, 8H), 1.25 (s, 65H), 1.14 (d, J = 7.2 Hz, 3H), 0.96 (dd, J = 6.9, 2.4 Hz, 3H), 0.88 (t, J = 6.7 Hz, 9H).

^{13}C NMR (101 MHz, $CDCl_3$) δ 174.6, 174.5, 173.6, 173.5, 139.2, 128.3, 127.9, 127.48, 127.46, 112.8, 99.9, 99.8, 94.8, 93.9, 93.5, 92.4, 85.7, 85.6, 76.2, 74.6, 74.6, 73.4, 73.1, 72.7, 72.6, 71.8, 71.7, 70.8, 70.6, 69.3, 69.2, 66.5, 64.4, 64.3, 64.1, 61.6, 61.5, 61.4, 43.0, 43.0, 42.1, 38.9, 38.8, 38.1, 38.0, 37.9, 36.4, 36.1, 35.8, 35.7, 33.6, 32.5, 32.1, 30.3, 30.2, 29.92, 29.85, 29.81, 29.80, 29.75, 29.66, 29.51, 29.50, 29.4, 29.1, 27.9, 27.8, 27.72, 27.66, 27.2, 27.0, 25.7, 25.6, 25.2, 25.1, 24.7, 24.6, 24.0, 23.7, 22.9, 22.8, 22.74, 22.65, 22.59, 21.1, 17.5, 17.4, 14.4, 14.3. The product most probably is present in two conformations.

HRMS (ESI+) m/z calcd for $[M+Na]^+$ 1341.9666; found 1341.9654.

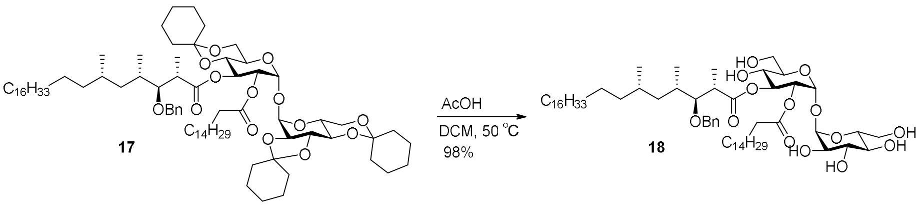

Compound **17** (26.0 mg, 0.02 mmol) was dissolved in DCM (2 mL) and then added to 80% aqueous AcOH (5 mL) and stirred at 50 °C for 7 h, after which the solvent was evaporated under reduced pressure and the resulting oil was co-evaporated with toluene. The crude colourless oil was purified by column chromatography (DCM/Acetone/MeOH 16/1/1) to provide 21.0 mg product **18** (98% yield).

^{1}H NMR (400 MHz, $CDCl_3$) δ 7.36 – 7.26 (m, 4H), 7.22 (td, *J* = 5.8, 4.9, 3.1 Hz, 1H), 5.40 (q, *J* = 9.4 Hz, 1H), 5.26 (d, *J* = 3.4 Hz, 1H), 5.19 (s, 1H), 4.85 (dd, *J* = 10.1, 3.4 Hz, 1H), 4.60 (d, *J* = 11.1 Hz, 1H), 4.49 (d, *J* = 11.3 Hz, 1H), 4.08 (s, 1H), 3.92 (d, *J* = 15.5 Hz, 2H), 3.85 – 3.71 (m, 2H), 3.69 – 3.61 (m, 2H), 3.62 – 3.54 (m, 2H), 3.54 – 3.47 (m, 1H), 3.32 (t, *J* = 9.5 Hz, 2H), 2.79 (t, *J* = 7.2 Hz, 1H), 1.95 (m, 1H), 1.80 (s, 1H), 1.45 (s, 2H), 1.25 (s, 53H), 1.11 (d, *J* = 6.9 Hz, 3H), 1.05 (d, *J* = 11.1 Hz, 2H), 0.93 (d, *J* = 6.5 Hz, 3H), 0.87 (q, *J* = 5.8, 5.0 Hz, 9H).

^{13}C NMR (101 MHz, $CDCl_3$) δ 176.5, 173.4, 138.8, 128.5, 127.9, 127.7, 95.3, 92.2, 85.9, 74.3, 73.5, 72.9, 72.6, 71.4, 71.0, 70.3, 69.2, 63.0, 60.8, 43.1, 39.3, 35.9, 33.5, 32.6, 32.1, 32.1, 30.34, 30.29, 30.0, 29.92, 29.89, 29.87, 29.82, 29.69, 29.58, 29.55, 29.52, 29.16, 27.11, 24.67, 22.9, 21.0, 17.22, 17.20, 17.12, 14.4, 14.3, 13.2.

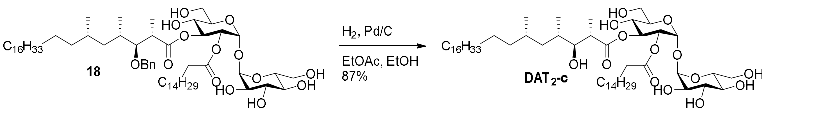

Compound **18** (22.0 mg, 0.02 mmol) was dissolved in EtOH (1.1 mL) and EtOAc (0.7 mL) and Pd/C (12.1 mg, 10% wt, 6 µmol) was added to the mixture. The flask was flushed with nitrogen, after which a hydrogen balloon was attached. The nitrogen was removed with vacuum (and backfilled 3 times-then removed) and the flask was purged with hydrogen (3 times). The reaction mixture was stirred at room temperature for 3 h, after which it was filtered through celite. The celite was washed with MeOH and the solvent was evaporated to yield the product. The crude product was purified by column chromatography (DCM/acetone/MeOH 8/1/1) to get the final product **DAT_2-c** 17.6 mg (87% yield).

^{1}H NMR (600 MHz, $CDCl_3/CD_3OD$ 4/1) δ 5.30 (t, *J* = 9.9 Hz, 1H), 5.12 (d, *J* = 3.6 Hz, 1H), 4.94 (d, *J* = 3.9 Hz, 1H), 4.76 (dd, *J* = 10.4, 3.6 Hz, 1H), 3.81 (ddd, *J* = 10.1, 5.3, 2.6 Hz, 1H), 3.69 (dd, *J* = 12.1, 2.7 Hz, 1H), 3.64 (t, *J* = 9.4 Hz, 1H), 3.60 – 3.52 (m, 3H), 3.49 – 3.43 (m, 2H), 3.38 (td, *J* = 9.7, 9.0, 3.6 Hz, 2H), 3.27 (t, *J* = 9.4 Hz, 1H), 2.53 (p, *J* = 7.4 Hz, 1H), 2.13 (t, *J* = 7.6 Hz, 2H), 1.56 (ddt, *J* = 10.2, 6.7, 3.6 Hz, 1H), 1.40 (q, *J* = 7.0 Hz, 2H), 1.35 – 1.27 (m, 1H), 1.10 (s, 49H), 0.96 (d, *J* = 6.8 Hz, 3H), 0.81 (d, *J* = 6.8 Hz, 3H), 0.72 (q, *J* = 6.7 Hz, 6H).

^{13}C NMR (151 MHz, $CDCl_3/CD_3OD$ 4/1) δ 175.3, 173.4, 94.4, 91.6, 78.0, 73.2, 72.5, 72.4, 72.0, 71.5, 70.2, 70.0, 69.0, 61.2, 44.2, 37.1, 35.4, 33.7, 32.1, 31.8, 30.0, 29.9, 29.62, 29.56, 29.54, 29.49, 29.3, 29.21, 29.17, 29.0, 24.5, 22.5, 20.7, 17.1, 14.1, 13.8.

HRMS (ESI+) m/z calcd for $[M+Na]^+$ 1011. 7318; found 1011.7310. $[\alpha]_D^{23}$ = +73.2° (c = 0.005, $CHCl_3$).

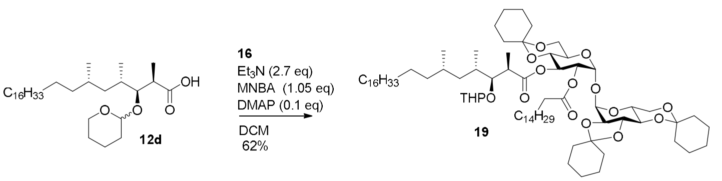

To a solution of compound **12d** (20.0 mg, 0.04 mmol) in dry DCM (1 mL) was added Et_3N (10.7 mg, 15.1 µL, 0.11 mmol), DMAP (0.48 mg, 4 µmol) and 2-methyl-6-nitrobenzoic anhydride (14.2 mg, 0.04 mmol) and the mixture was stirred for 10 min, after which cyclohexylidene trehalose **16** (28.4 mg, 0.04 mmol) in dry DCM (1 mL) was added. The reaction was stirred at room temperature overnight. The reaction was quenched with

saturated aqueous NH_4Cl, water was added and the aqueous layer was extracted with DCM. The combined organic layers were washed with water, dried over Na_2SO_4, filtered and concentrated under reduced pressure. Column chromatography 0-10% EtOAc/heptane gave pure product **19** 31.9 mg (63% yield).

^{1}H NMR (400 MHz, $CDCl_3$) δ 5.36 – 5.32 (m, 1H), 5.29 (d, J = 4.1 Hz, 1H), 4.93 (td, J = 10.2, 3.7 Hz, 1H), 4.57 (ddd, J = 14.1, 5.2, 2.7 Hz, 1H), 4.17 (td, J = 9.4, 5.7 Hz, 1H), 4.05 (td, J = 10.2, 5.2 Hz, 1H), 3.97 – 3.81 (m, 3H), 3.83 – 3.65 (m, 5H), 3.56 (dddd, J = 10.6, 9.0, 5.1, 1.5 Hz, 1H), 3.52 – 3.37 (m, 2H), 2.61 (dtt, J = 9.3, 4.8, 2.7 Hz, 1H), 2.41 – 2.29 (m, 2H), 2.15 (d, J = 15.5 Hz, 1H), 1.93 – 1.77 (m, 5H), 1.73 – 1.44 (m, 33H), 1.25 (d, J = 4.6 Hz, 62H), 1.13 (d, J = 7.0 Hz, 2H),0.93 (d, J = 6.8 Hz, 2H), 0.88 (m, 9H).

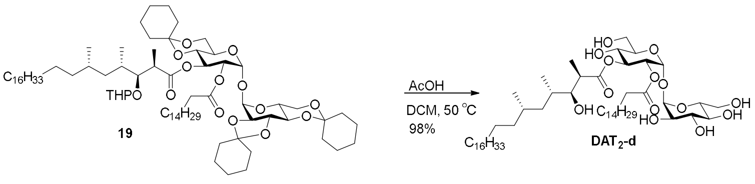

Compound **19** (32.0 mg, 25 µmol) in DCM was added into 80% aqueous AcOH (5 mL) and stirred at 50 °C for 4 h. The solvent was evaporated under reduced pressure and the resulting oil was co-evaporated with toluene. Column chromatography (DCM/MeOH/Acetone 8/1/1) gave the final product **DAT_2-d** (23.5 mg, 98% yield).

^{1}H NMR (600 MHz, $CDCl_3/CD_3OD$) δ 5.34 (t, J = 9.7 Hz, 1H), 5.14 (d, J = 3.6 Hz, 1H), 4.96 (d, J = 3.7 Hz, 1H), 4.74 (dd, J = 10.3, 3.6 Hz, 1H), 3.84 (ddd, J = 10.2, 5.5, 2.6 Hz, 1H), 3.71 (dd, J = 12.2, 2.6 Hz, 1H), 3.66 (t, J = 9.4 Hz, 1H), 3.62 – 3.52 (m, 5H), 3.49 – 3.42 (m, 2H), 3.40 (dd, J = 9.8, 3.8 Hz, 1H), 3.29 (t, J = 9.4 Hz, 1H), 2.55 (qd, J = 7.1, 3.0 Hz, 1H), 2.15 (t, J = 7.5 Hz, 2H), 1.53 (ddd, J = 13.2, 9.7, 3.1 Hz, 1H), 1.40 (ddt, J = 13.4, 6.5, 3.2 Hz, 3H), 1.11 (s, 56H), 0.96 (d, J = 7.0 Hz, 3H), 0.80 (dt, J = 13.4, 4.5 Hz, 1H), 0.74 (q, J = 7.0 Hz, 12H), 0.68 (d, J = 6.7 Hz, 3H).

^{13}C NMR (151 MHz, $CDCl_3/CD_3OD$ 4/1) δ 176.1, 173.7, 94.8, 91.9, 76.5, 73.4, 72.8, 72.7, 72.4, 71.8, 70.7, 70.2, 69.5, 61.6, 61.4, 42.5, 41.5, 35.7, 34.1, 33.6, 32.1, 30.4, 30.2, 29.93, 29.87, 29.84, 29.80, 29.7, 29.5, 29.3, 27.2, 25.0, 22.8, 21.2, 16.0, 14.1, 8.5.

HRMS (ESI+) m/z calcd for $[M+NH_4]^+$ 1006.7764; found 1006.7778. $[\alpha]_D^{23}$ = +37.2° (c = 0.005, $CHCl_3$).

Determination of natural DAT_2 by HPLC-CID analysis

Purification of the DAT from *M. tuberculosis* H37Rv.

Mtb cells were extracted three times in a chloroform/methanol (C/M) mixture (1:2, l:l, 2:1 v/v, successively) at room temperature. The whole extract was concentrated and resuspended in chloroform (100 ml), and washed twice with an equal volume of water. The organic phase was brought to dryness, and solubilized in acetone (25 mL) and allowed to precipitate overnight at 4°C. The suspension was centrifuged at 4 °C for 10 min (3000 rpm). The "acetone-soluble" fraction was then fractionated on a silica acid column (2 × 24 cm) eluted with increasing amounts of methanol in chloroform. DAT were eluted in C/M 9/1 (v/v) mixture. As this fraction was still contaminated by sulfoglycolipids, a QMA Sep-pak (Waters, France) was realized. DAT were eluted in C/M 9/1 (v/v) mixture while sulfoglycolipids required ammonium acetate 0.1 M in C/M to be eluted. As a dye was still present in the DAT-containing fraction, a reverse phase C18 Sep-pak (Waters, France) was realized. The dye was eluted using methanol/water (l:l, v/v) eluent while the DAT were eluted with pure methanol. Purification was checked by TLC on aluminium-backed silica gel plates (Alugram Sil G; Macherey-Nagel) using C/M 9:1, v/v, as migration solvent. A spray composed of 0.1% orcinol in 40% H_2SO_4 was used to detect glycolipids. DAT purity was also checked by 1D ^{1}H and 2D ^{1}H-^{13}C NMR.

MALDI-TOF and NMR analysis

Maldi-Tof analysis was performed on an AB Sciex TOF/TOF 5800 mass spectrometer using the reflectron mode. Ionization was achieved by irradiation with an Nd:YAG laser (349 nm) operating with a pulse rate of 400 Hz. The laser intensity was set at 2000 and the continuous stage motion was used with a velocity of 600 µm/sec. Acquisition of the data was done in the positive ion mode. Typically, spectra from 3000 to 5000 laser shots were summed to obtain the final spectrum. Typically, 0.5µL (1 µg) of DAT dissolved in chloroform was mixed directly on the target with 0.5µL of matrix solution, 2-(4-hydroxyphenylazo)benzoic acid (HABA, Sigma) dissolved at 10 mg/mL in ethanol/water 1:1 (by volume).

NMR spectra were recorded at 298K with a cryo-probed Bruker DRX600 spectrometer (Karlsruhe, Germany) using to 2D ^{1}H-^{13}C HMQC sequence. Native molecules were dissolved in $CDCl_3$-CD_3OD (D, 99.99% from Euriso-Top, Saint-Aubin, France) (9:l, v/v) and analyzed in 200 x 5 mm UL-5 NMR tubes (Euriso-Top, Saint-Aubin, France). Proton and carbon chemical shifts are expressed in part per million downfield from the signal of external acetone (δH 2.225 and δC 31.45).

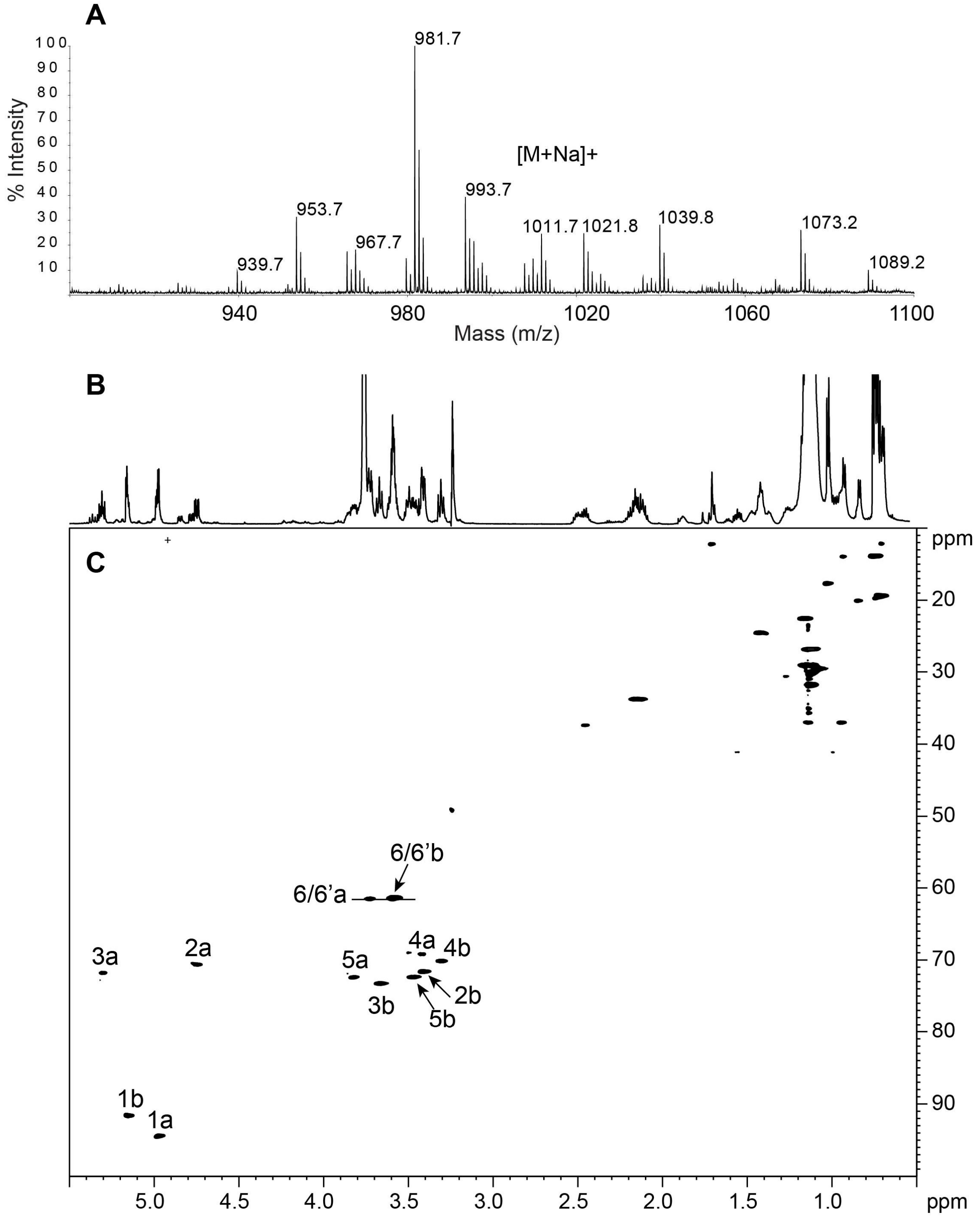

Fig. S3. Structural characterization of natural DAT (DATn) purified from Mtb H37Rv. Positive ion mode MALDI MS spectrum (A), 1D ^{1}H NMR (B) and 2D ^{1}H-^{13}C HSQC (C) spectra. The two glucose spin systems are annotated as "a" and "b". System "b" corresponds to the 2,3-diacyl glucose.

HPLC-MS Analysis of DAT_2 diastereomers

The synthetic DAT_2 and the natural DAT_n were analyzed on an UPLC (Waters: Acquity, Binary Solvent manager) coupled to a triple quadrupole mass detector (Waters) using a C-18 HPLC column (Acquity UPLC® BEH C18, 1.7 µm, 2.1 mm × 50 mm) and a gradient method with 28:12:51:9 methanol: water: 1-propanol: cyclohexane (solvent A) and 85:15 1-propanol: cyclohexane (solvent B). Both solvents contained 2.0 mM ammonium formate. 0.1% water was added to solvent B to aid dissolution. When the LC-MS method of our previous report[1] was used, which was performed on an Agilent Technologies 6530 Accurate-Mass Q-TOF LC/MS, but now implemented on the available Waters TQD system in our lab, we experienced overpressure. We ascribed this to solvent miscibility problems in the LC system. In order to solve the overpressure problem, the starting mobile phase was premixed as the new solvent A, which worked well when we changed the quadrupole solvent manager to a binary solvent manager. The solvent gradient used a 0.3 mL/min flowrate throughout and started at 40% solvent B, increased linearly starting at 1.0 min and ending at 50% solvent B at 10.0 min, holding at 50% B until 15.0 min. Every sample was prepared in the starting mobile phase at a concentration of 1 mg/mL. Runs were initiated with 5 µL sample injections. Extracted ion chromatograms were generated using MassLynx software (Waters) in order to compare the retention times of the DAT_2 compounds.

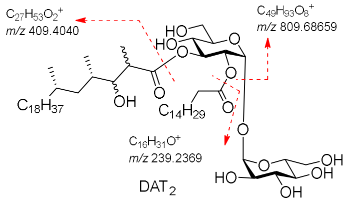

Main fragmentation reactions of DAT_2 by Collision-Induced Dissociation mass spectrometry. Extracted ion chromatograms of ions corresponding with the ammonium adduct (m/z = 1006.78) showed m/z values consistent with those expected from DAT_2.

CID (collision induced dissociation) was carried out in positive mode, 20 V, argon as collision gas. MassLynx software was used for data acquisition and for comparing the MS^2 spectra of the different DAT_2 isomers. CID results of the DAT_2 isomers and the natural DAT_n yielded the ions shown in the Figures below. Calculated masses for ions not shown in Figure 3B appear below.

Table S1. Mass data of DAT_2

DAT_2 isomer	$M+NH_4^+$	loss of ammonia	loss of H_2O	loss of hexose	loss of palmitoyl	loss of H_2O	hexose-palmitoly (H^+)	loss of water
Calculated mass	1006.78	989.75	971.74	809.69	571.46	553.45	401.29	383.28

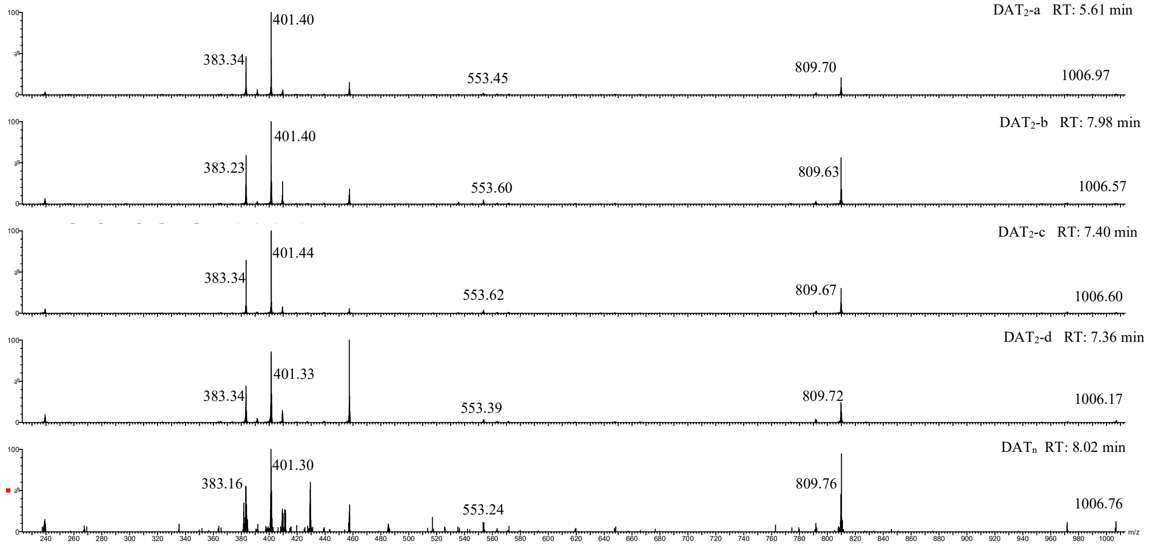

Collision-Induced Dissociation of DAT_2 stereoisomers. Extracted ion chromatograms of ions corresponding with the ammonium adduct of DAT_2 (calculated m/z =1006.78). Synthetic DAT_2 stereoisomers and bacterial DAT_n were collided at 20 V via HPLC-MS. The spectra of the bacterial DAT_n were compared to the CID spectra for the respective synthesized DAT_2 isomers to rule in structural matches based on more than one fragment match with better than acceptable mass accuracy. Synthetic DAT_2-b and natural sample have the same retention time and the same parent ion m/z window collided.

Comparison of the mycolipanolic acids by GC-MS

Hydrolysis of bacterial DAT_n and conversion of mycolipanolic acid into its methyl ester

To a stirred solution of 0.2 mg natural (bacterial) DAT_n in 0.2 mL THF, a solution of aqueous Bu_4NOH (1.5 M, 0.010 ml) was added at 0 °C. The mixture was stirred at room temperature for 5 h. The reaction mixture was poured in an aqueous $KHSO_4$ solution (5%, 10 ml) and extracted with ether. The combined organic layer was washed with brine, dried over $MgSO_4$ and concentrated in vacuo to get light yellow oil.

The oil obtained was carefully dried and dissolved in 0.2 mL dry toluene: MeOH (3:2) under N_2. 50 µL Trimethylsilyldiazomethane (0.6 M) was added and the mixture was stirred for 30 min. Then volatiles were evaporated to obtain the natural methyl mycolipanoate.

0.7 mg mycolipanolic acid isomers (**10a-d**) were separately dissolved in 0.2 mL dry toluene: MeOH (3:2) under N_2. 30 µL Trimethylsilyldiazomethane (0.6 M, 10 eq) was added and the mixtures were stirred for 30 min. Then the samples were evaporated to dryness to obtain methyl mycolipanoate-a, b, c, d.

GC-MS Analysis of methyl mycolipanoates

Methyl mycolipanoate stereoisomers were analysed by GC–MS on an SHIMADZU GCMS-QP2010 system (Shimadzu corporation, Japan). 1 µL of each sample in $CHCl_3$ was injected with a 1:10 split ratio at 112.7 kPa and an inlet temperature of 270 °C. An HP-5MS (5% phenyl, polymethyl siloxane column of 30 m × 250 mm i.d. × 0.25 µm film thickness) was used. The injected sample was split equally between the MS and EI detectors at the end of the GC column. The initial oven temperature was 230 °C, rising to 260 °C at a rate of 1 °C/min. Helium was used as carrier gas at a flow rate of 1.0 ml/min. MS data was acquired in the scan model from 30 to 500 m/z.

Desired methyl mycolipanoates peaks were identified according to the MS results, the fragments ionization fits with the structures. Due to high energy of the EI source, none of these compounds form molecular ions (expected at m/z = 440) that survive through the ion source. MS spectra gave characteristic ion fragment (m/z 88, 117) of the methyl esters.[9] All of these compounds have the same peaks at m/z =88, 177, 352, corresponding to the ions resulted from α-cleavage of the hydroxyl group.

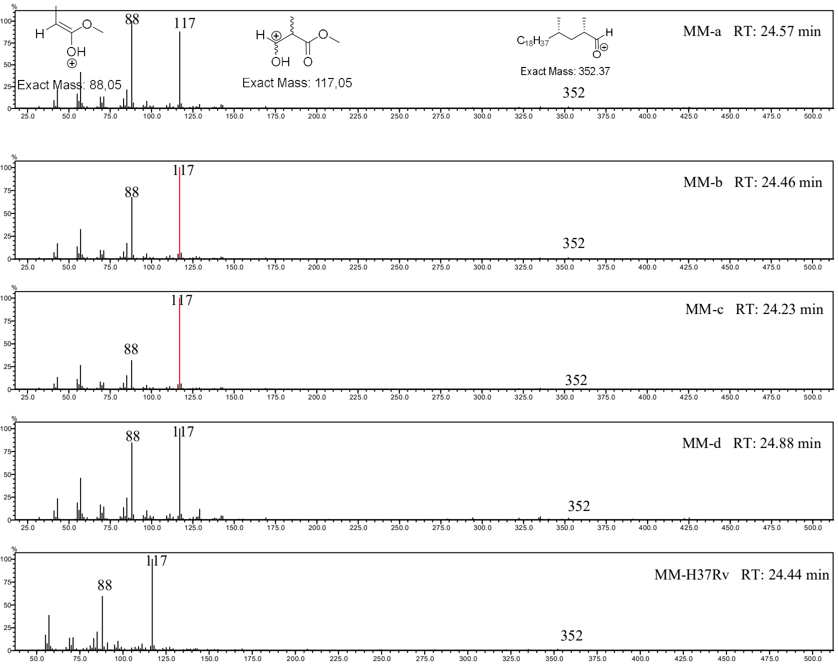

Fig. S4 MS spectra of methyl mycolipanoates from GC-MS. Synthetic methyl mycolipanoate-b and natural sample have the same rentention time and the same ionization fragments.

Comparison of ^{1}H-NMR signals of the synthetic DAT_2 diastereomers with the natural sample

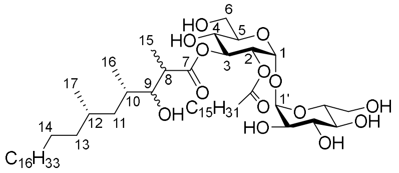

Table S2 Comparison of 1H-NMR signals of the synthetic DAT_2 diastereomers with the natural sample

Proton	chemical shift DAT_2-a [ppm]	chemical shift DAT_2-b [ppm]	chemical shift DAT_2-c [ppm]	chemical shift DAT_2-d [ppm]	chemical shift DAT_n [ppm]
H-3	4.764	4.761	4.763	4.745	4.765
H-2	5.298	5.301	5.297	5.336	5.293
H-8	2.545	2.496	2.529	2.553	2.470
H-9	3.706	3.723	3.688	3.712	3.708
H-10	1.488	1.600	1.561	1.530	1.602
H-11	1.403	1.409	1.393	1.400	1.413
H-13	1.034	0.914	0.961	0.962	0.920
H-14	0.734	0.736	0.729	0.736	0.740
H-15	0.767	0.686	0.706	0.683	0.696

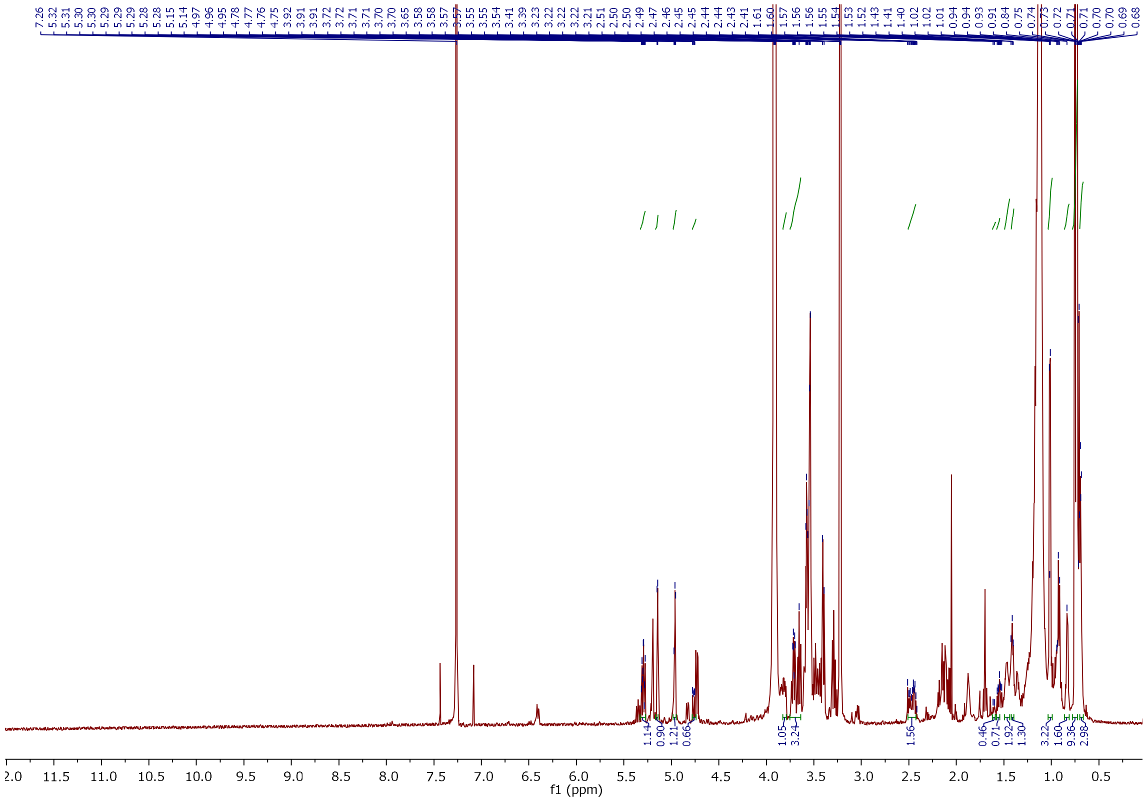

^{1}H NMR of natural DAT_n extracted from the laboratory H37Rv strain (600 MHz, $CDCl_3/CD_3OD$ 4/1)

Mincle Reporter Cell Line Experiments

Glycolipids (1 mg/mL in isopropanol) were added to 96-well plates (Nunc) in serial dilutions from 10 µg to 0.1 ng/well, followed by evaporation of the solvents (7). The HEK-Blue™ hMincle and HEK-Blue™ mMincle cell lines (InvivoGen), derivatives of HEK293 cells that stably express the human or murine Mincle gene along with a NF-κB-inducible reporter system (secreted alkaline phosphatase) were maintained in Dulbecco's modified Eagle's medium (DMEM, Gibco) containing 10% Fetal Bovine Serum (FBS, Gibco) 4.5 g/l glucose, 2 mM L-glutamine, 100 U/ml penicillin, 100 µg/ml streptomycin (Sigma) and 100 µg/ml zeocin, 200 µg/ml hygromycin, 10 µg/ml blasticidin, 1 µg/ml puromycin and 50 µg/ml mofetil (all from Invivogen). Reporter cells (5×10^4 /well) were stimulated for 24h, after which alkaline phosphatase activity was measured by mixing 20 µL of the culture supernatant and 180 µL of Quanti-Blue (InvivoGen) and reading OD at 630 nm. [10]

References

[1] M. Holzheimer, J. F. Reijneveld, A. K. Ramnarine, G. Misiakos, D. C. Young, E. Ishikawa, T. Y. Cheng, S. Yamasaki, D. B. Moody, I. Van Rhijn, *ACS Chem. Biol.* **2020**, *15*, 1835-1841.

[2] Y. Schmidt, K. Lehr, U. Breuninger, G. Brand, T. Reiss, B. Breit, *J. Org. Chem.* **2010**, *75*, 4424-4433.

[3] B. ter Horst, J. van Wermeskerken, B. L. Feringa, A. J. Minnaard, *Eur. J. Org. Chem.* **2010**, 38–41.

[4] T. Inoue, J. F. Liu, D. C. Buske, A. Abiko, *J. Org. Chem.* **2002**, *67*, 5250-5256.

[5] L. C. Dias, E. C. Polo, *J. Org. Chem.* **2017**, *82*, 4072-4112.

[6] M. Das, Y. Du, J. S. Mortensen, M. Ramos, L. Ghani, H. J. Lee, H. E. Bae, B. Byrne, L. Guan, C. J. Loland, *Org. Biomol. Chem.* **2019**, *17*, 3249-3257.

[7] J. Guiard, A. Collmann, M. Gilleron, L. Mori, G. De Libero, J. Prandi, G. Puzo, *Angew. Chem.* **2008**, *120*, 9880-9884.

[8] V. A. Sarpe, S. S. Kulkarni, *Org. Lett.* **2014**, *16*, 5732-5735.

[9] a) X. Xie, C. Khosla, D. E. Cane, *J. Am. Chem. Soc.* **2017**, *139*, 6102-6105; b) J. L. Vicario, D. Badia, E. Dominguez, M. Rodríguez, L. Carrillo, *J. Org. Chem.* **2000**, *65*, 3754-3760.

[10] A. Decout, S. Silva-Gomes, D. Drocourt, S. Barbe, I. André, F. J. Cueto, T. Lioux, D. Sancho, E. Pérouzel, A. Vercellone, *Proc. Natl. Acad. Sci. U.S.A.* **2017**, *114*, 2675-2680.

NMR Spectra

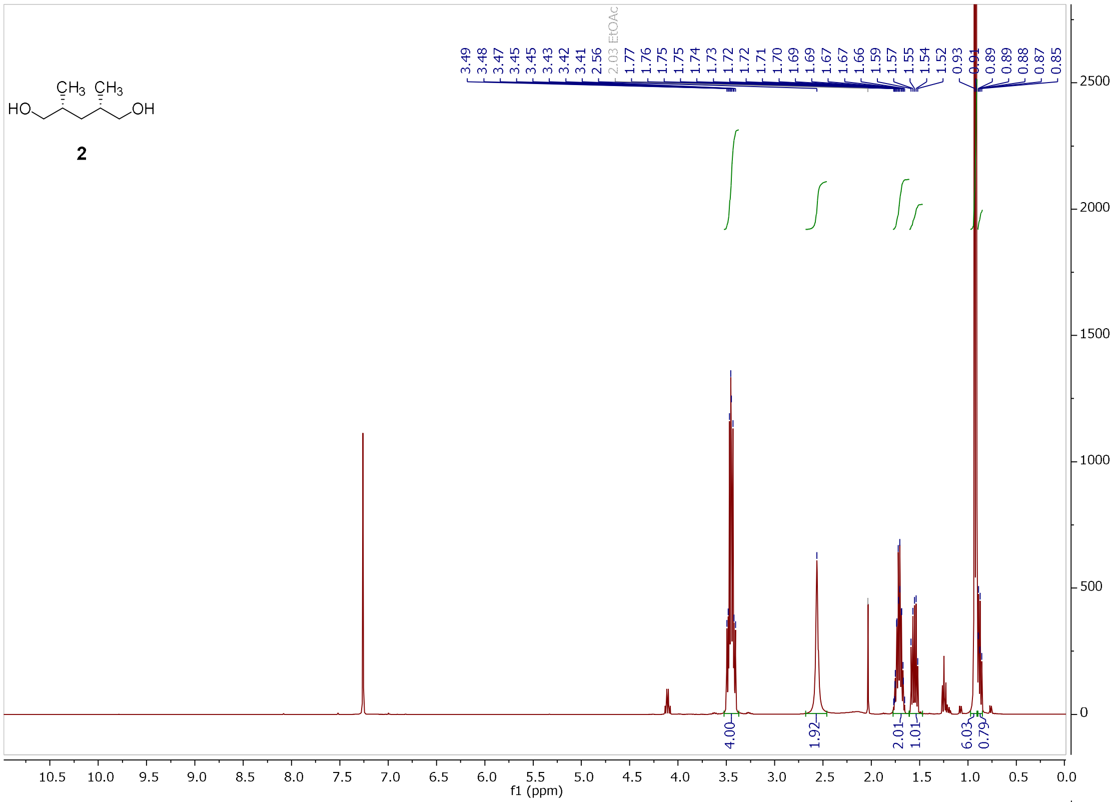

HO
OH
CH3
CH3
2
3.49
3.48
3.47
3.45
3.45
3.43
3.42
3.41
2.56
2.03 EtOAc
1.77
1.76
1.75
1.75
1.74
1.73
1.72
1.72
1.71
1.70
1.69
1.69
1.67
1.67
1.66
1.59
1.57
1.55
1.54
1.52
0.93
0.91
0.89
0.89
0.88
0.87
0.85
4.00
1.92
2.01
1.01
6.03
0.79
f1 (ppm)

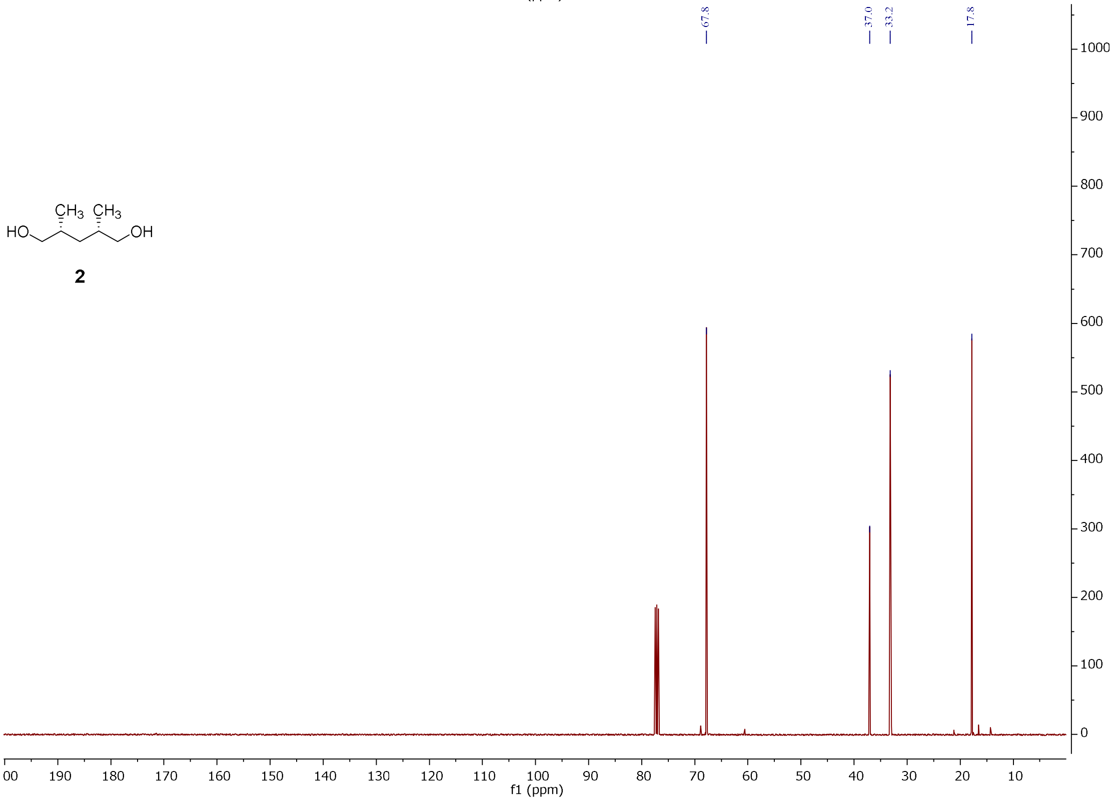

HO
OH
CH3
CH3
2
67.8
37.0
33.2
17.8
f1 (ppm)

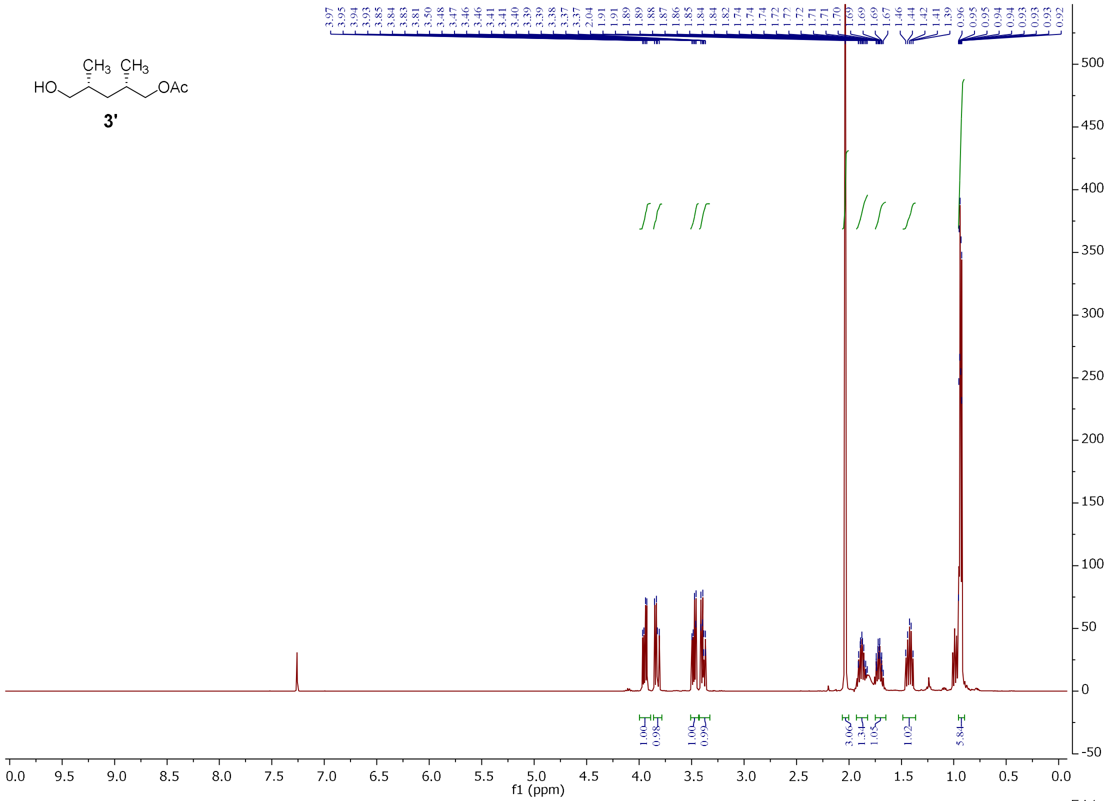
HO
CH3
CH3
OAc
3'
f1 (ppm)

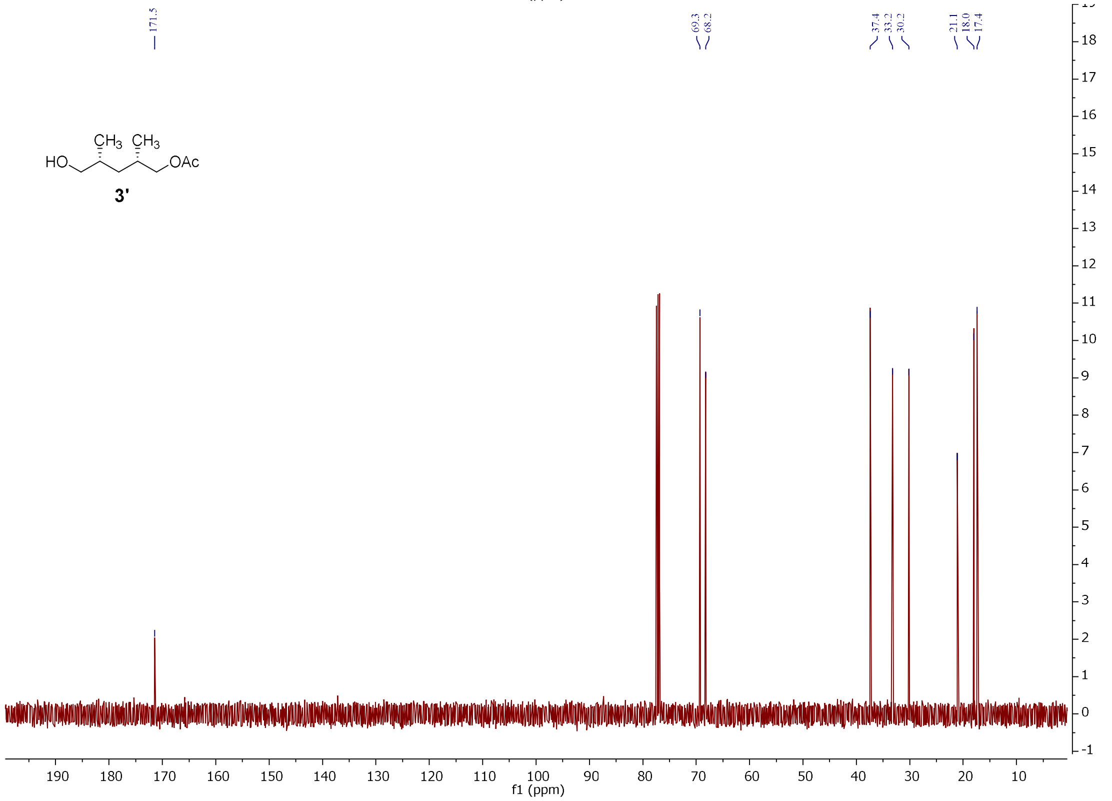
HO
CH3
CH3
OAc
3'
171.5
69.3
68.2
37.4
33.2
30.2
21.1
18.0
17.4
f1 (ppm)

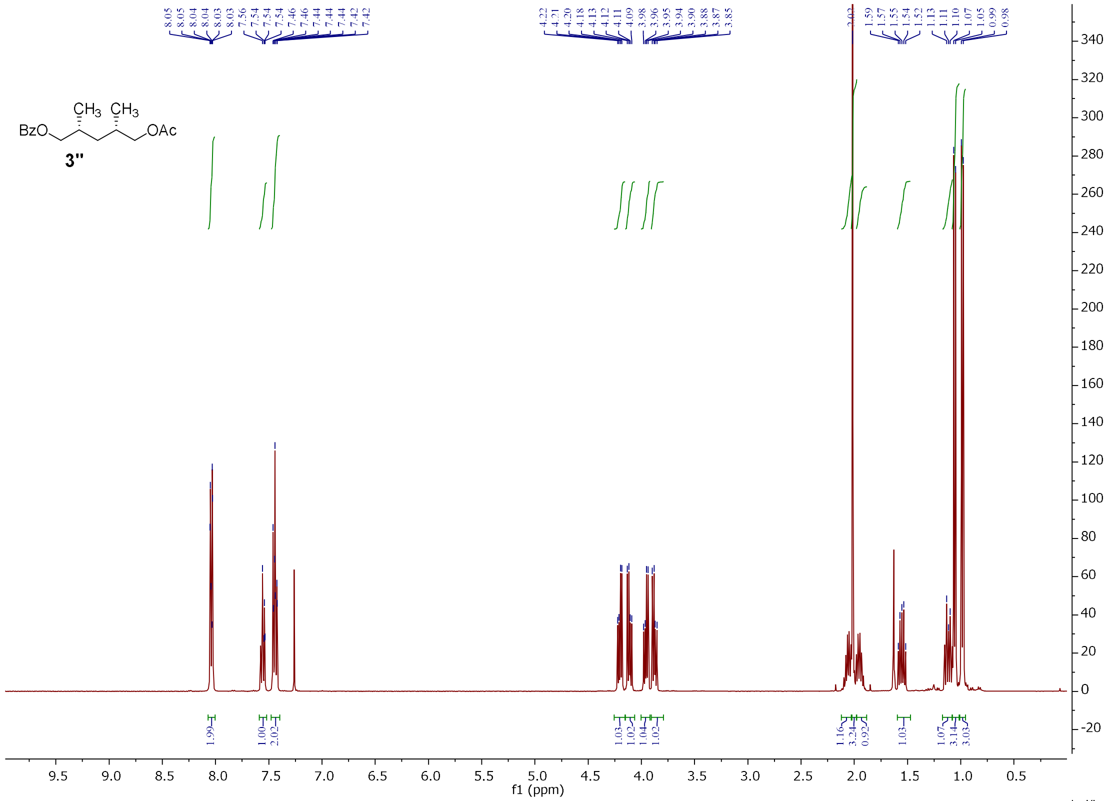

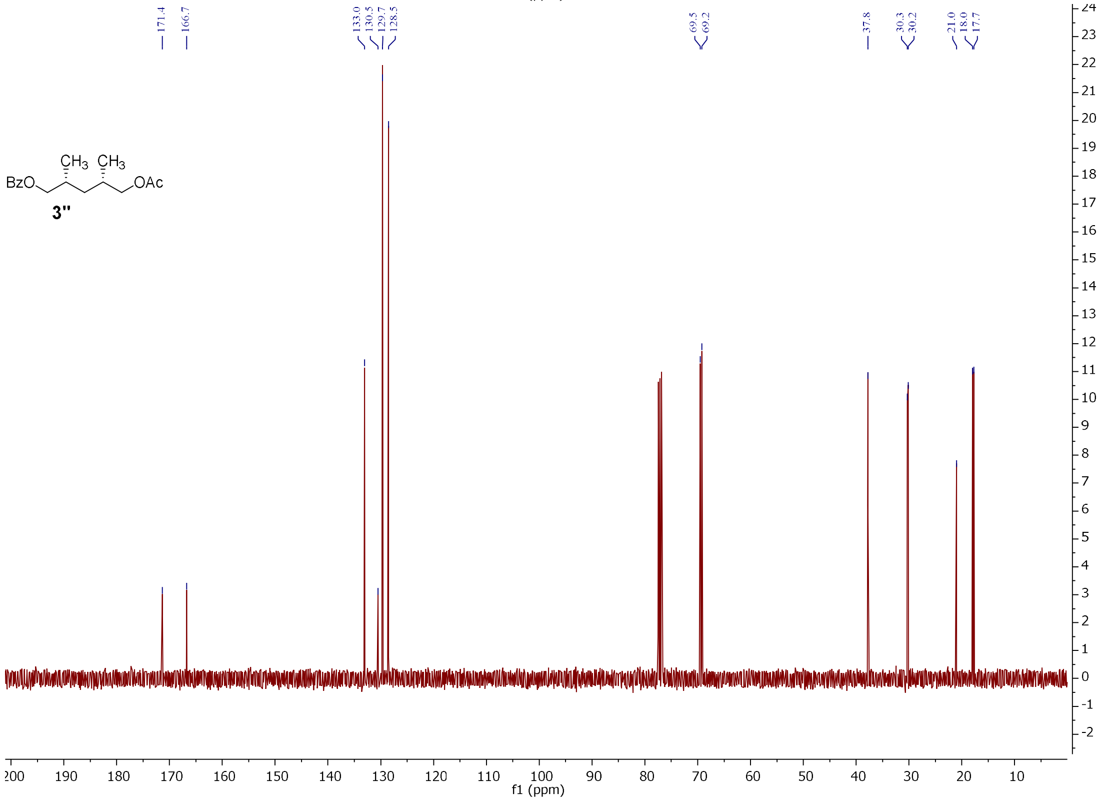

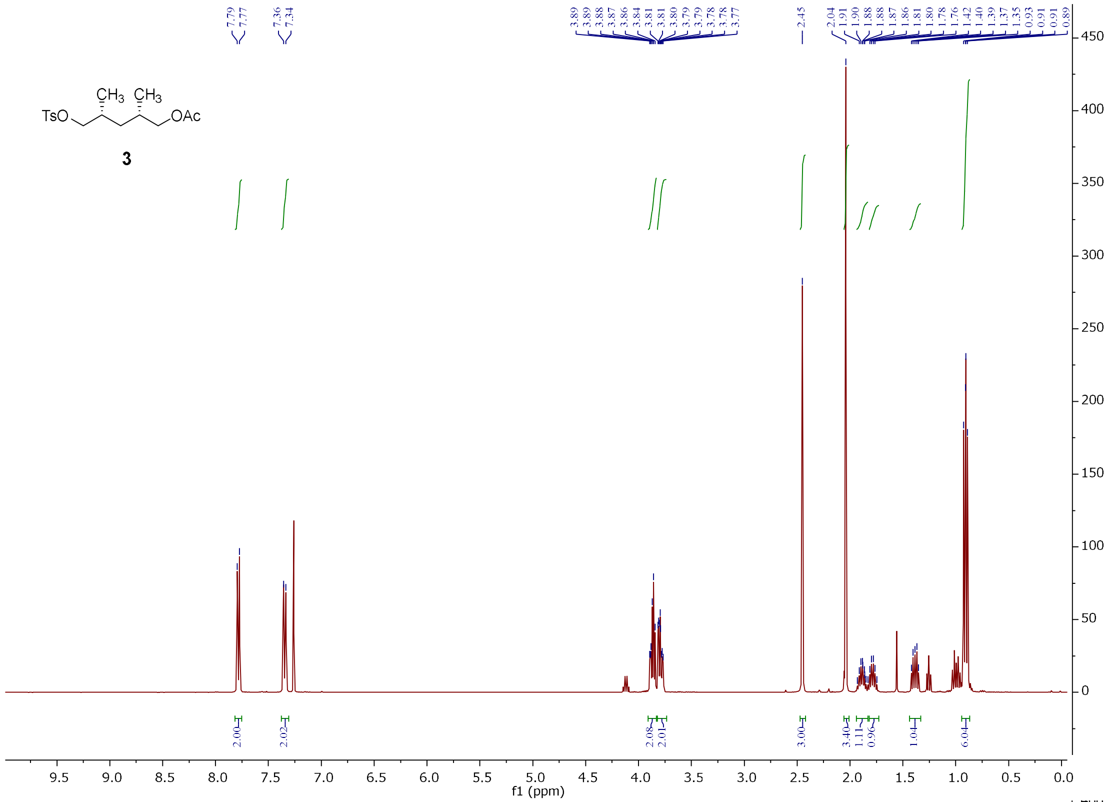
CH3 CH3
TsO OAc
3
7.79
7.77
7.36
7.34
3.89
3.89
3.88
3.87
3.86
3.84
3.81
3.81
3.80
3.79
3.79
3.78
3.78
3.77
2.45
2.04
1.91
1.90
1.88
1.88
1.87
1.86
1.81
1.80
1.78
1.76
1.42
1.40
1.39
1.37
1.35
0.93
0.91
0.91
0.89
2.00
2.02
2.08
2.01
3.00
3.40
1.11
0.96
1.04
6.04
f1 (ppm)

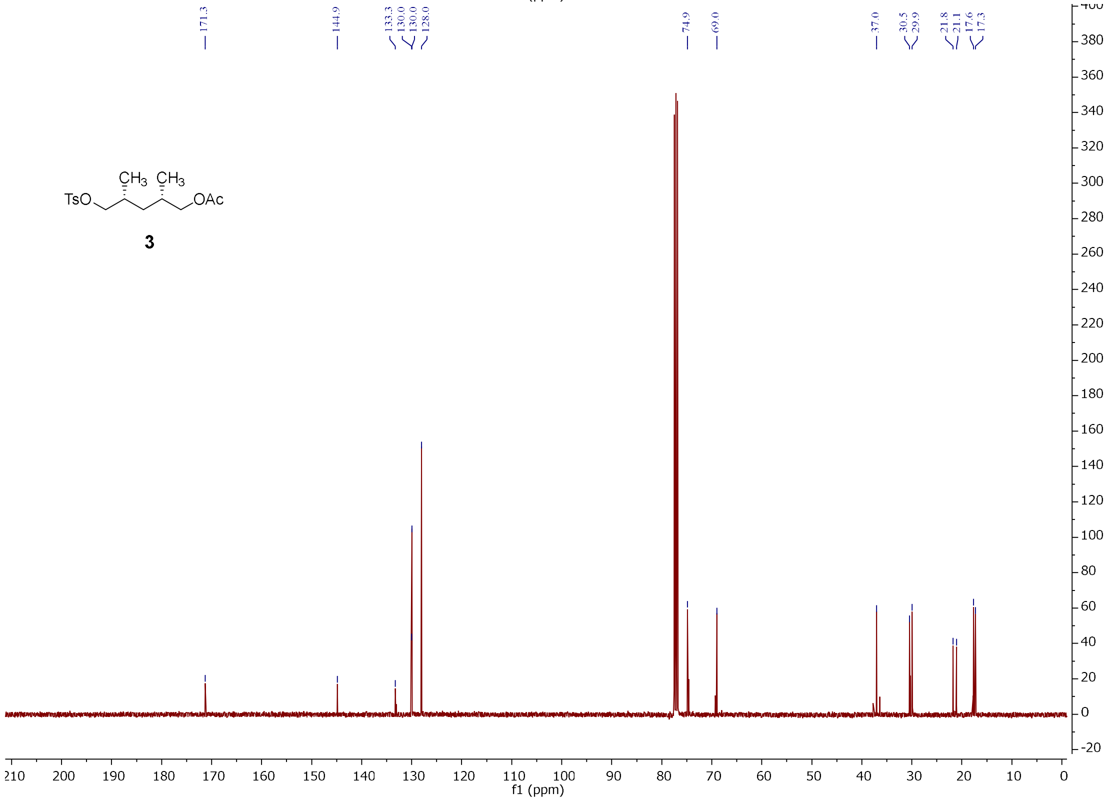
CH3 CH3
TsO OAc
3
171.3
144.9
133.3
130.0
130.0
128.0
74.9
69.0
37.0
30.5
29.9
21.8
21.1
17.6
17.3
f1 (ppm)

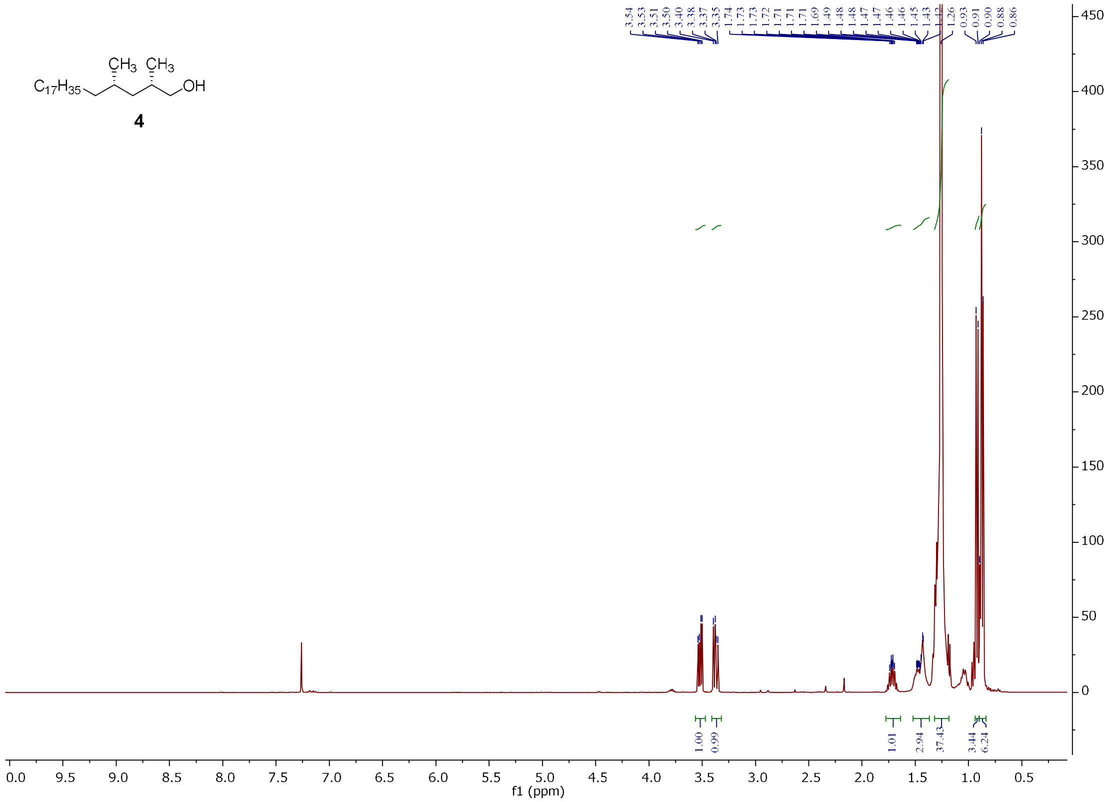

3.54 3.53 3.51 3.50 3.40 3.38 3.37 3.35 1.74 1.73 1.73 1.72 1.71 1.71 1.71 1.69 1.49 1.48 1.48 1.47 1.47 1.46 1.46 1.45 1.43 1.26 0.93 0.91 0.90 0.88 0.86
CH3 CH3
C17H35 OH
4
1.00 0.99 1.01 2.94 37.43 3.44 6.24
f1 (ppm)

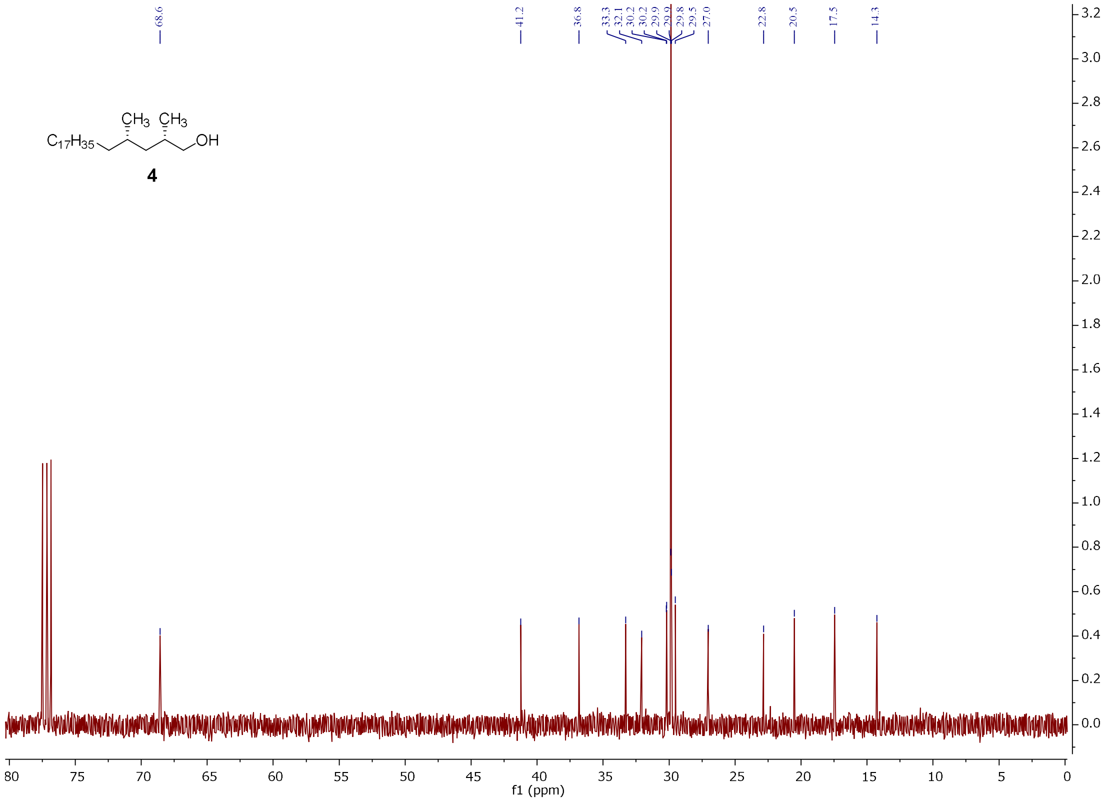

68.6 41.2 36.8 33.3 32.1 30.2 30.2 29.9 29.9 29.8 29.5 27.0 22.8 20.5 17.5 14.3
CH3 CH3
C17H35 OH
4
f1 (ppm)

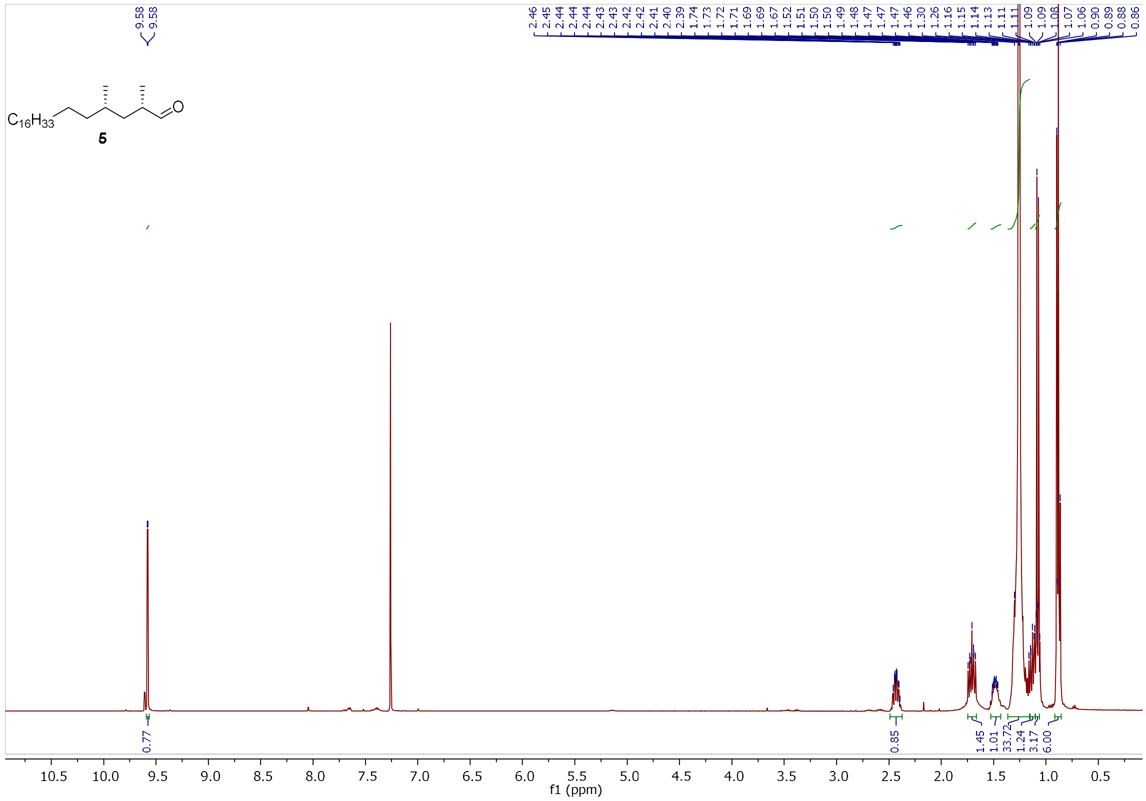
9.58
9.58
2.46
2.45
2.44
2.44
2.44
2.43
2.43
2.42
2.42
2.41
2.40
2.39
1.74
1.73
1.72
1.71
1.69
1.69
1.67
1.52
1.51
1.50
1.50
1.49
1.48
1.47
1.47
1.46
1.30
1.26
1.16
1.15
1.14
1.13
1.11
1.11
1.09
1.09
1.08
1.07
1.06
0.90
0.89
0.88
0.86
C16H33
5
0.77
0.85
1.45
1.01
33.72
1.24
3.17
6.00
10.5
10.0
9.5
9.0
8.5
8.0
7.5
7.0
6.5
6.0
5.5
5.0
4.5
4.0
3.5
3.0
2.5
2.0
1.5
1.0
0.5
f1 (ppm)

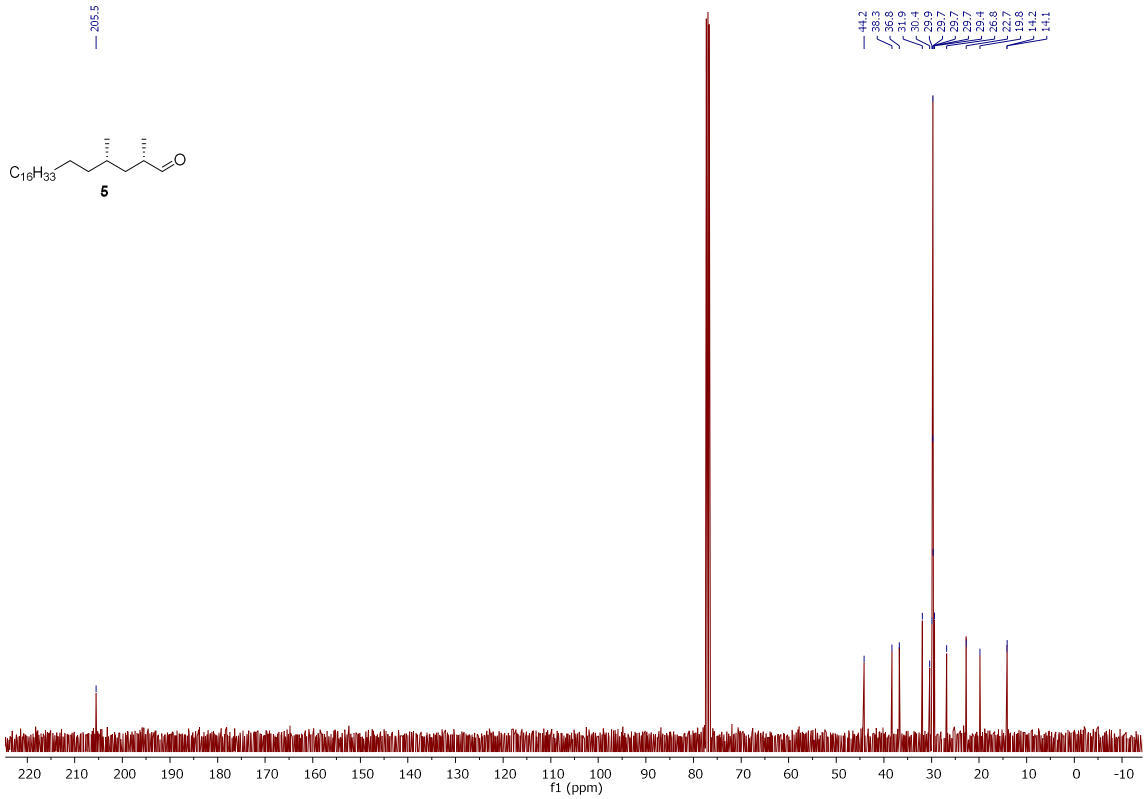
205.5
44.2
38.3
36.8
31.9
30.4
29.9
29.7
29.7
29.7
29.4
26.8
22.7
19.8
14.2
14.1
C16H33
5
220
210
200
190
180
170
160
150
140
130
120
110
100
90
80
70
60
50
40
30
20
10
0
-10
f1 (ppm)

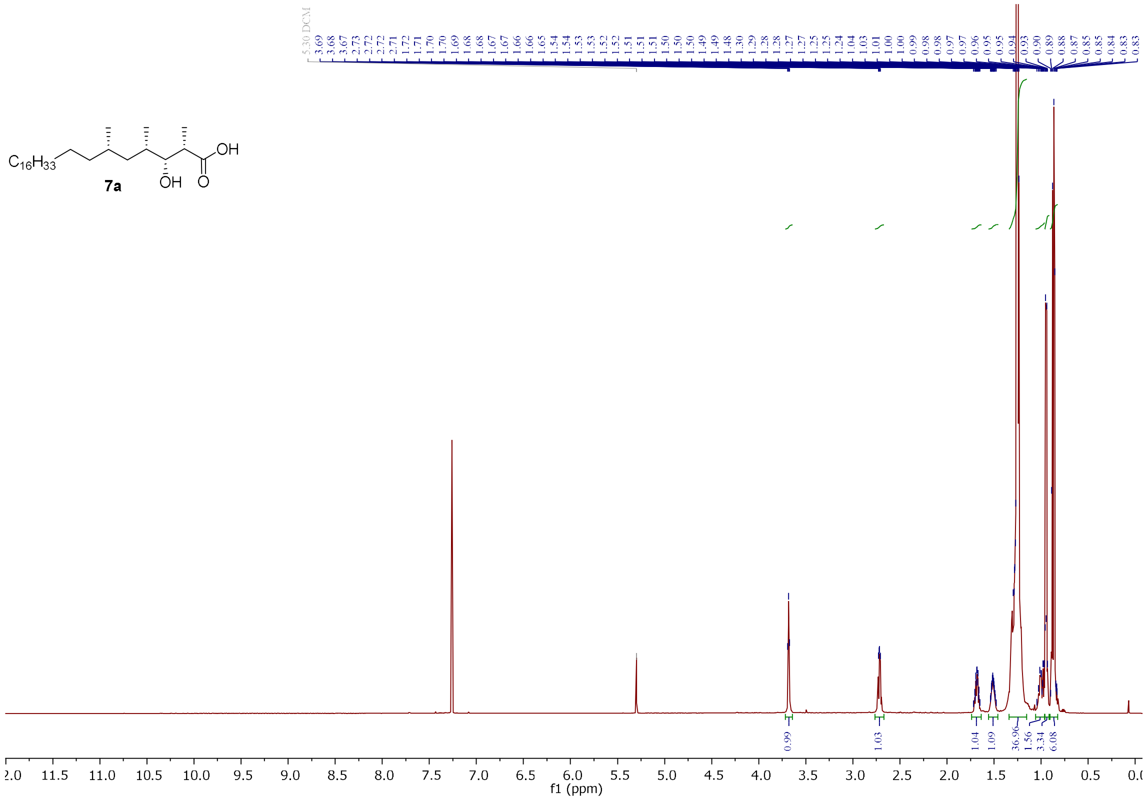
C16H33
7a
OH
OH
O
f1 (ppm)

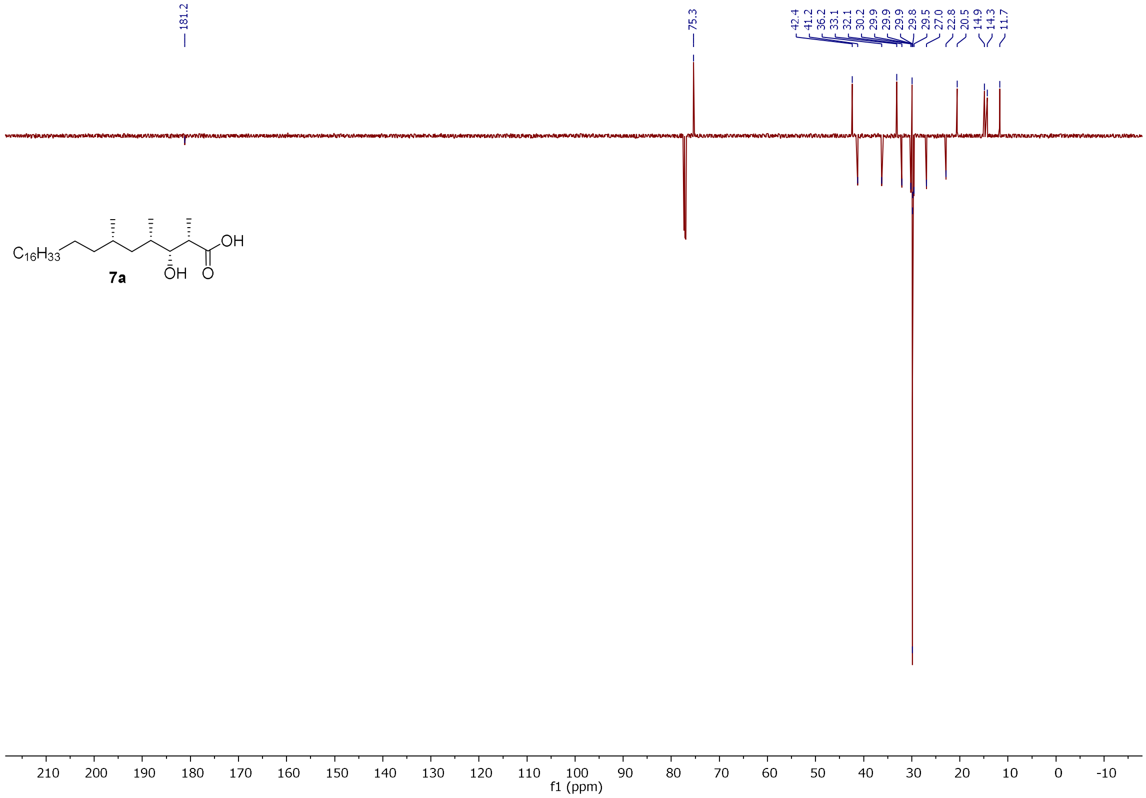
181.2
75.3
42.4
41.2
36.2
33.1
32.1
30.2
29.9
29.9
29.9
29.8
29.5
27.0
22.8
20.5
14.9
14.3
11.7
C16H33
7a
OH
OH
O
f1 (ppm)

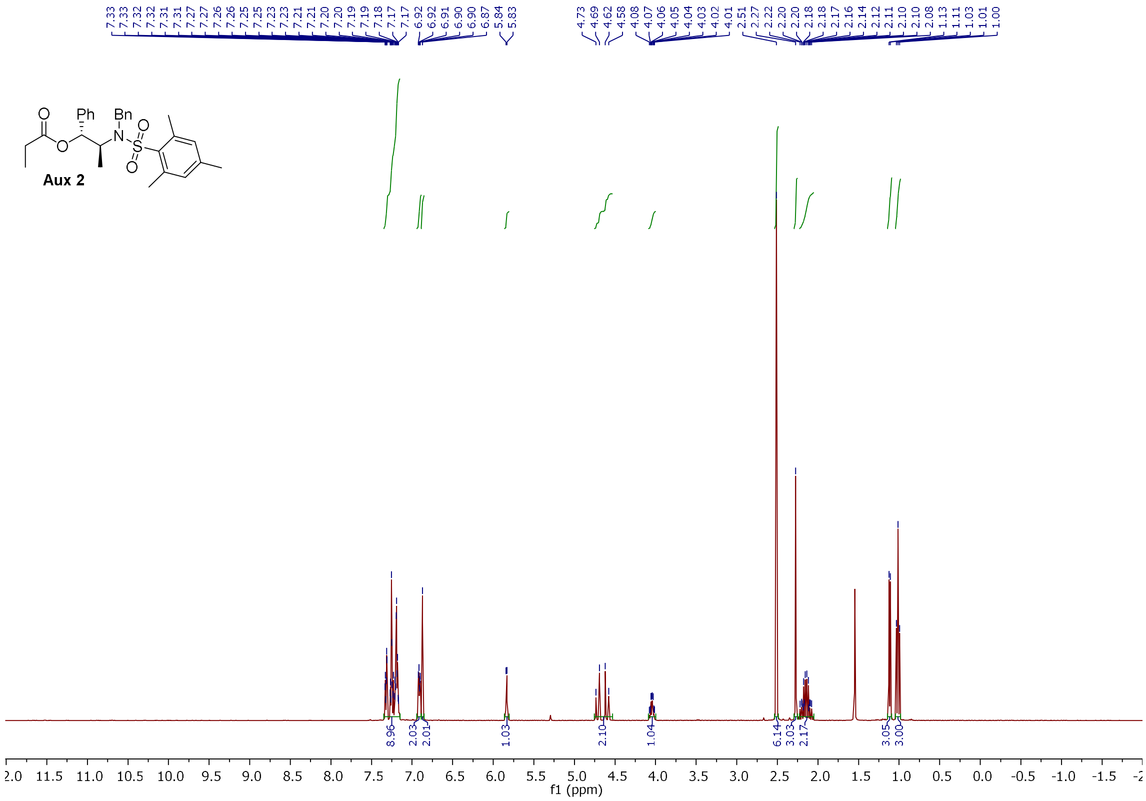
7.33 7.33 7.32 7.32 7.31 7.31 7.27 7.27 7.26 7.26 7.25 7.25 7.23 7.23 7.21 7.21 7.20 7.20 7.19 7.19 7.18 7.17 7.17 6.92 6.92 6.91 6.90 6.90 6.87 5.84 5.83
4.73 4.69 4.62 4.58 4.08 4.07 4.06 4.05 4.04 4.03 4.02 4.01 2.51 2.27 2.22 2.20 2.20 2.18 2.18 2.17 2.16 2.14 2.12 2.11 2.10 2.10 2.08 1.13 1.11 1.03 1.01 1.00
Ph
Bn
Aux 2
8.96 2.03 2.01 1.03 2.10 1.04 6.14 3.03 2.17 3.05 3.00
f1 (ppm)

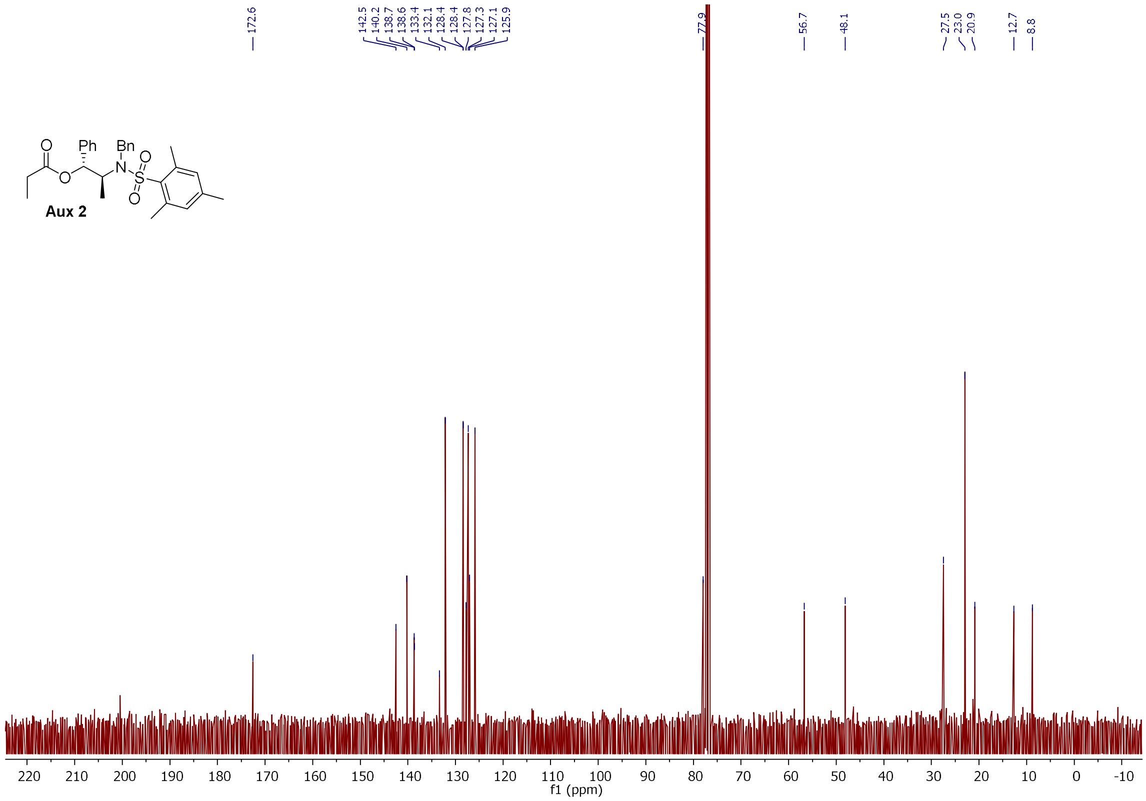
172.6
142.5 140.2 138.7 138.6 133.4 132.1 128.4 128.4 127.8 127.3 127.1 125.9
77.9
56.7
48.1
27.5 23.0 20.9
12.7
8.8
Ph
Bn
Aux 2
f1 (ppm)

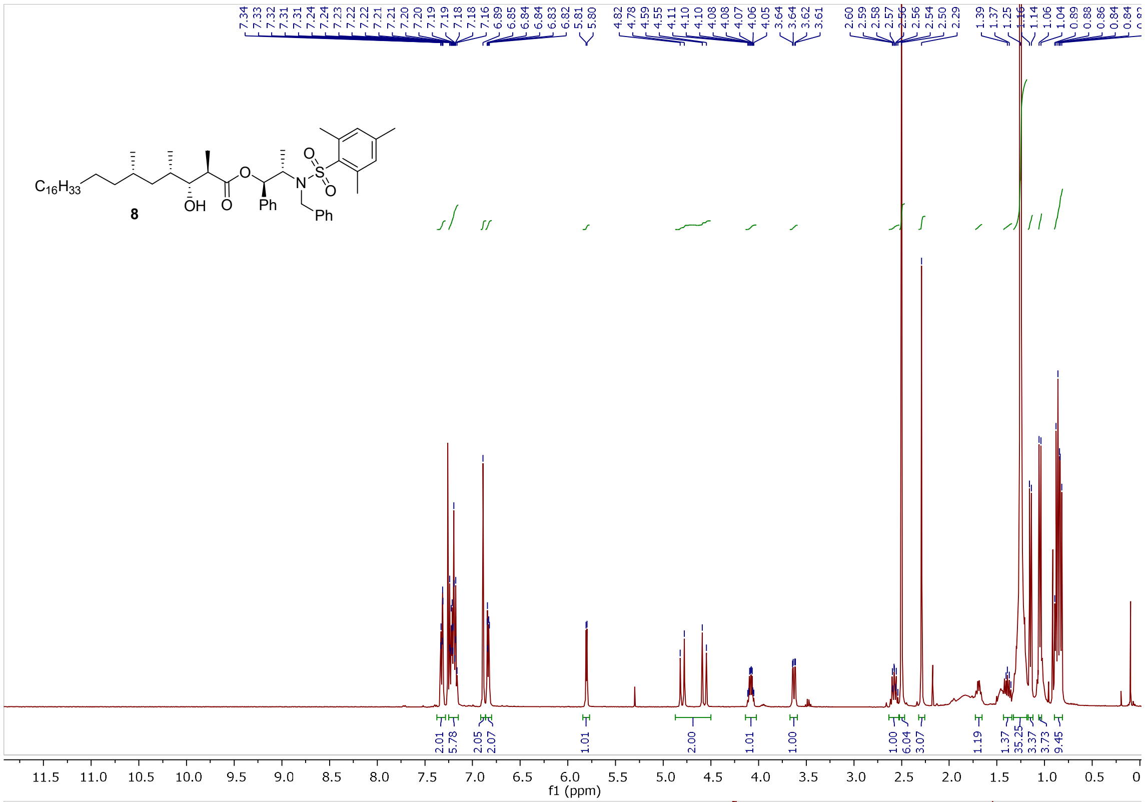

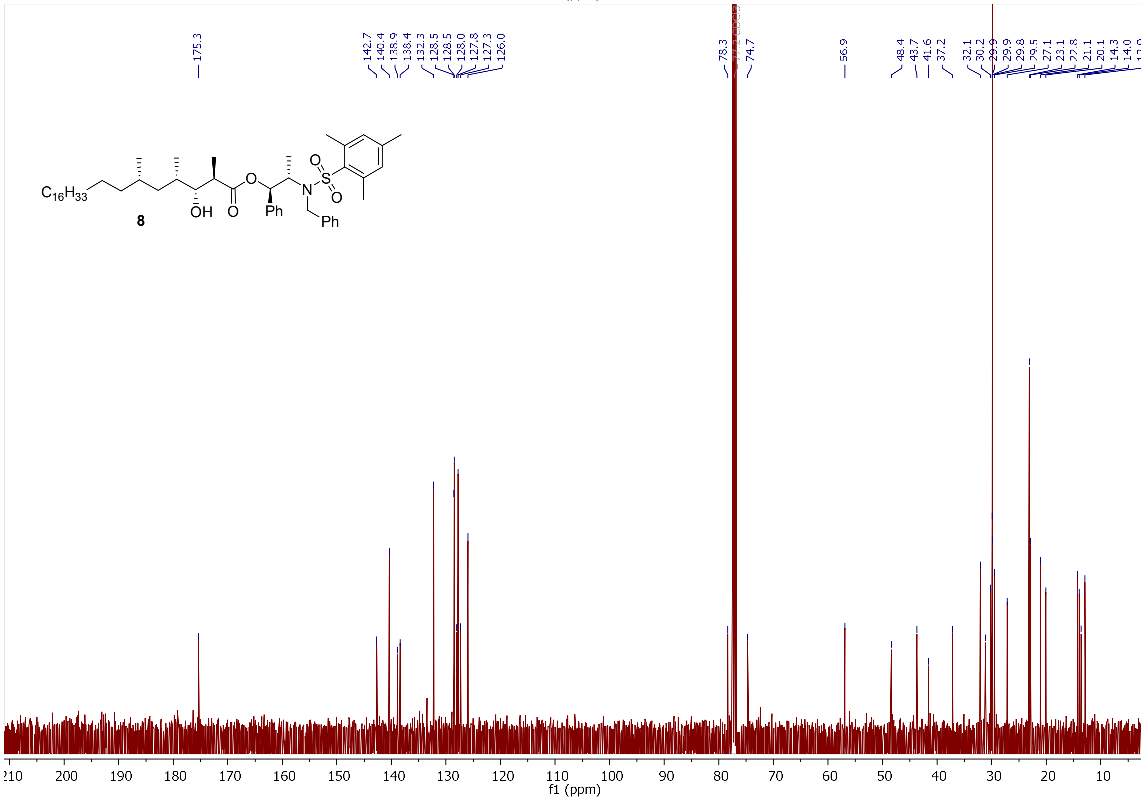

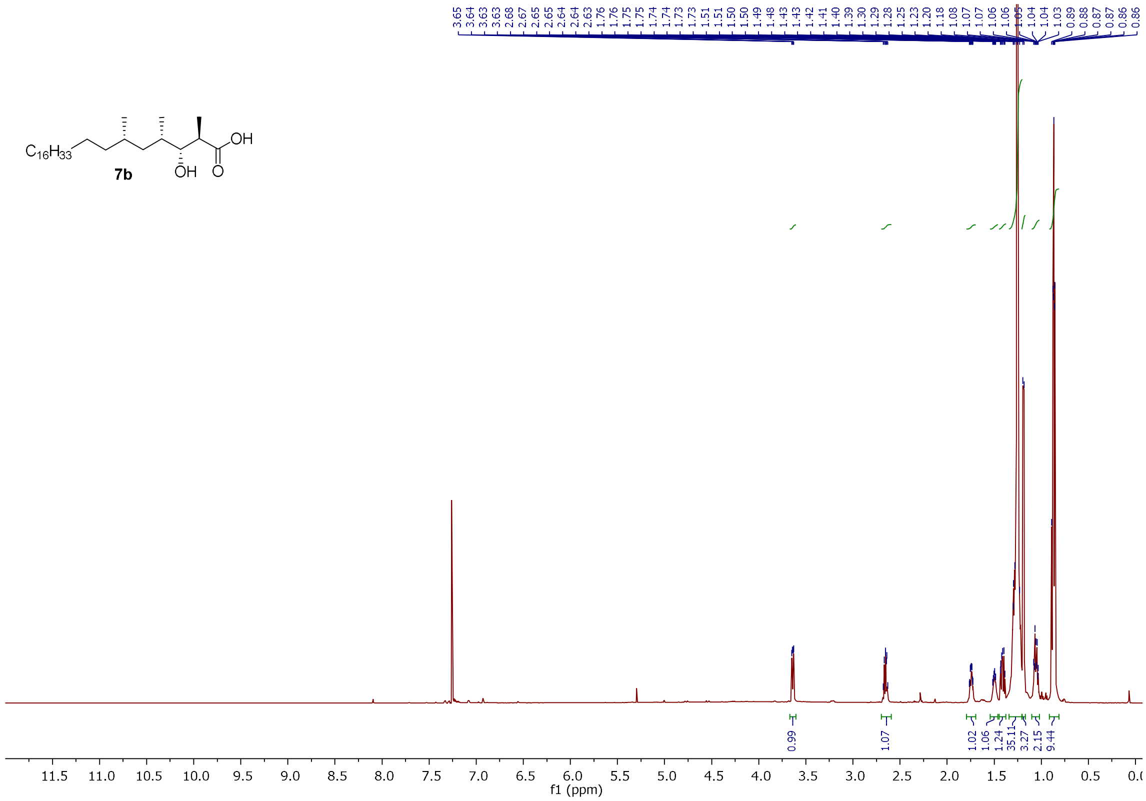
3.65 3.64 3.63 3.63 2.68 2.67 2.65 2.65 2.64 2.63 1.76 1.76 1.75 1.75 1.74 1.74 1.73 1.73 1.51 1.51 1.50 1.50 1.49 1.48 1.43 1.42 1.41 1.40 1.39 1.30 1.29 1.28 1.25 1.23 1.20 1.18 1.08 1.07 1.07 1.06 1.06 1.05 1.04 1.04 1.03 0.89 0.88 0.87 0.86 0.86
$C_{16}H_{33}$
OH
OH
O
7b
0.99
1.07
1.02
1.06
1.24
35.11
3.27
2.15
9.44
f1 (ppm)

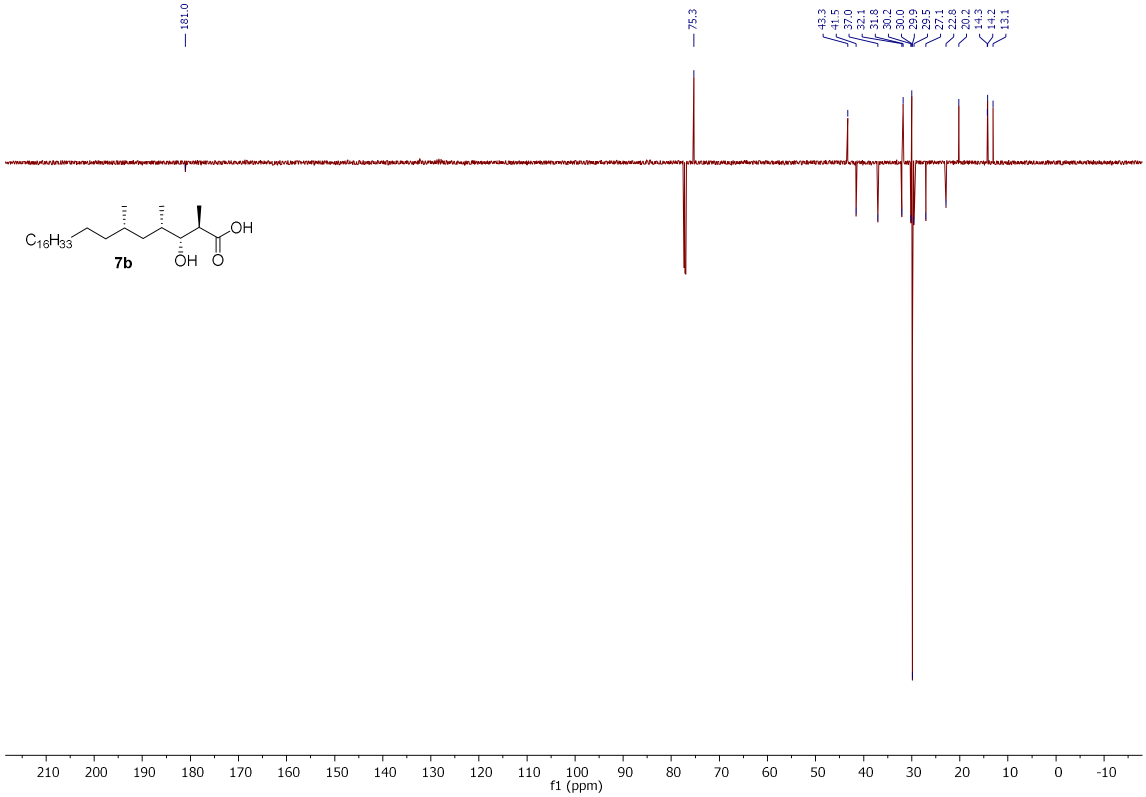
181.0
75.3
43.3 41.5 37.0 32.1 31.8 30.2 30.0 29.9 29.5 27.1 22.8 20.2 14.3 14.2 13.1
$C_{16}H_{33}$
OH
OH
O
7b
f1 (ppm)

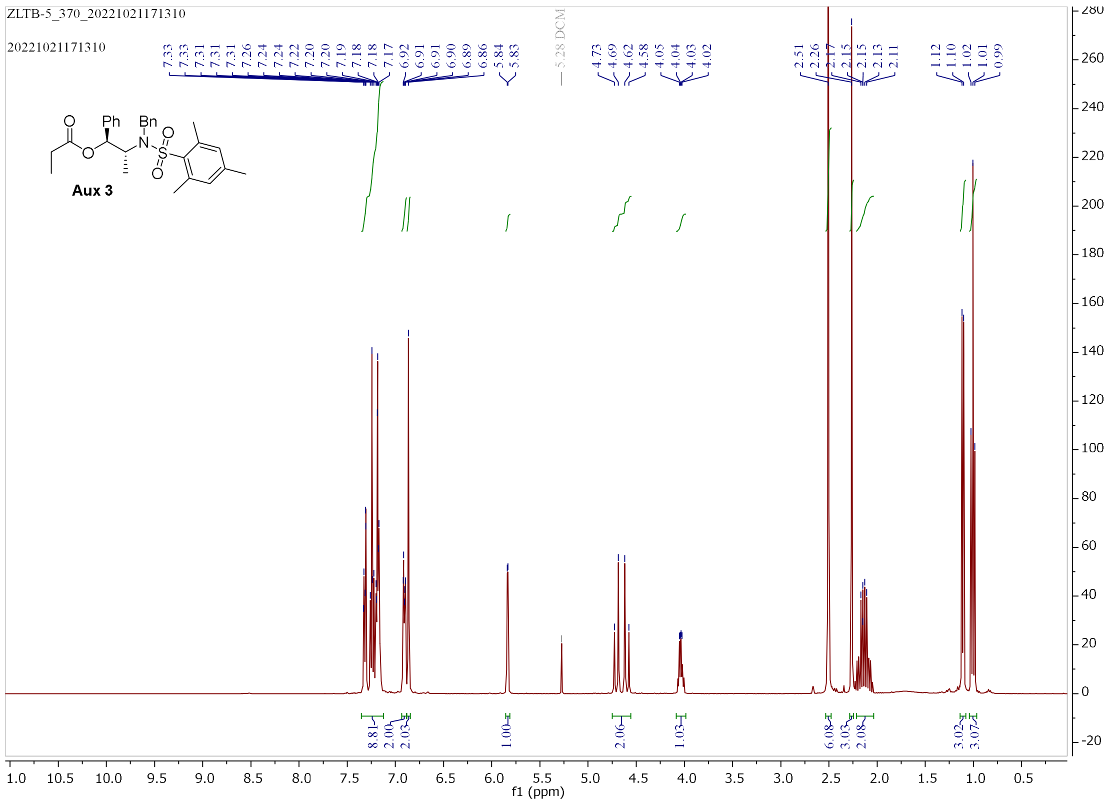
ZLTB-5_370_20221021171310
20221021171310
Ph
Bn
Aux 3
5.28 DCM
f1 (ppm)

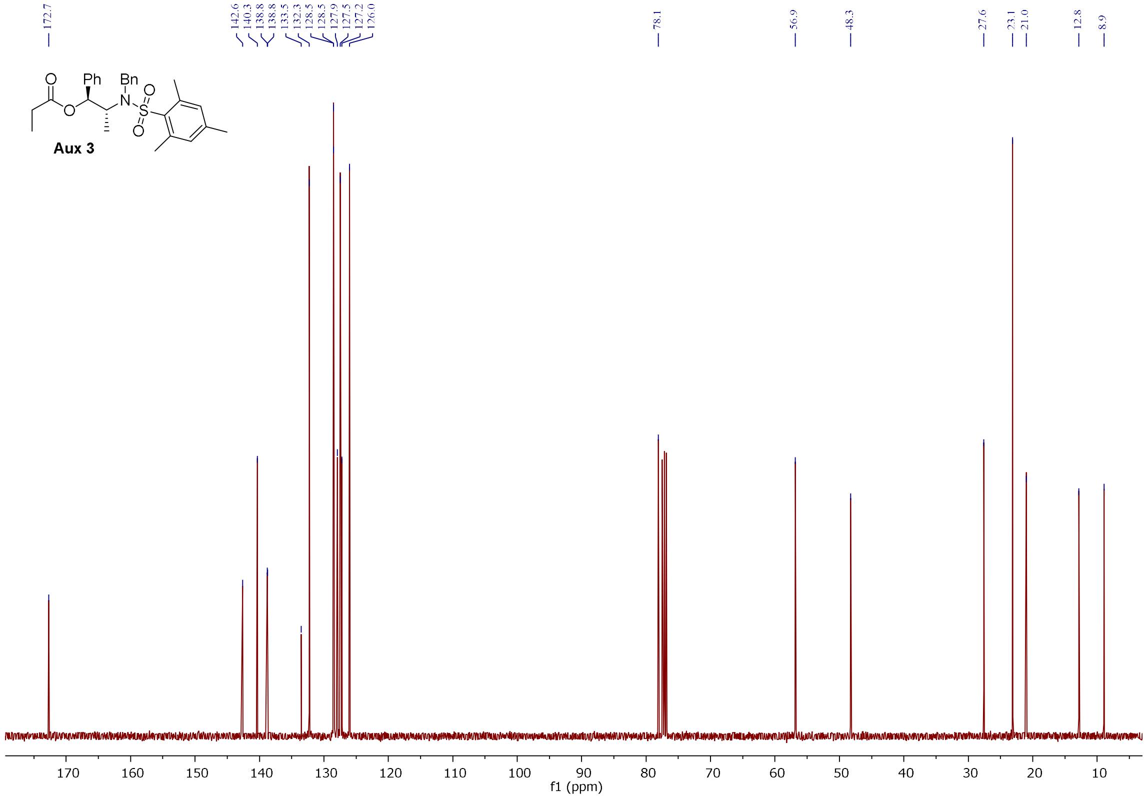
172.7
142.6
140.3
138.8
138.8
133.5
132.3
128.5
128.5
127.9
127.5
127.2
126.0
78.1
56.9
48.3
27.6
23.1
21.0
12.8
8.9
Ph
Bn
Aux 3
f1 (ppm)

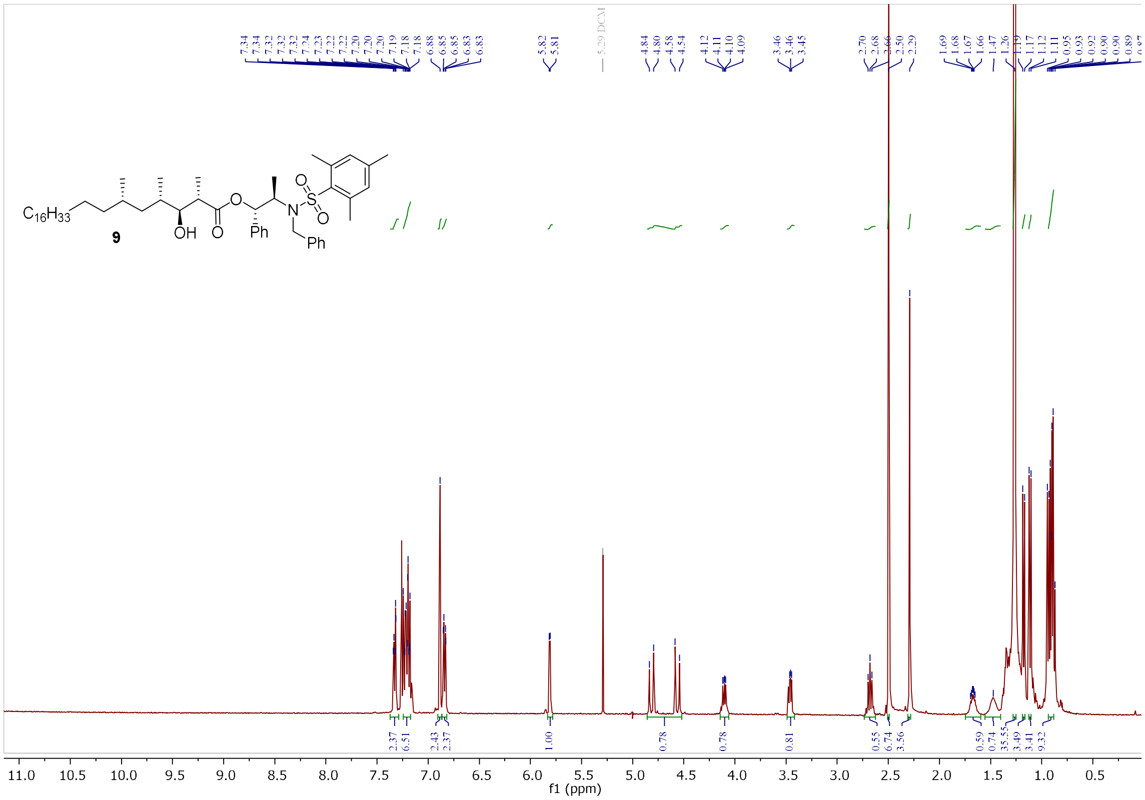

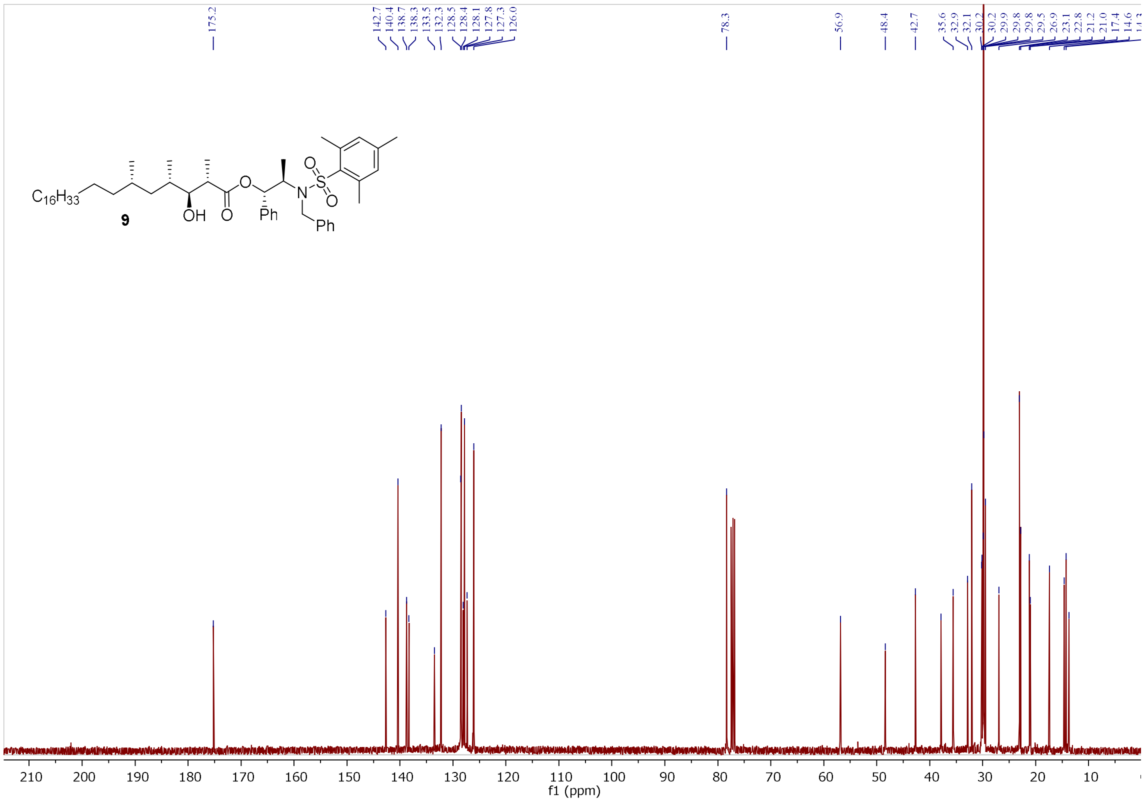

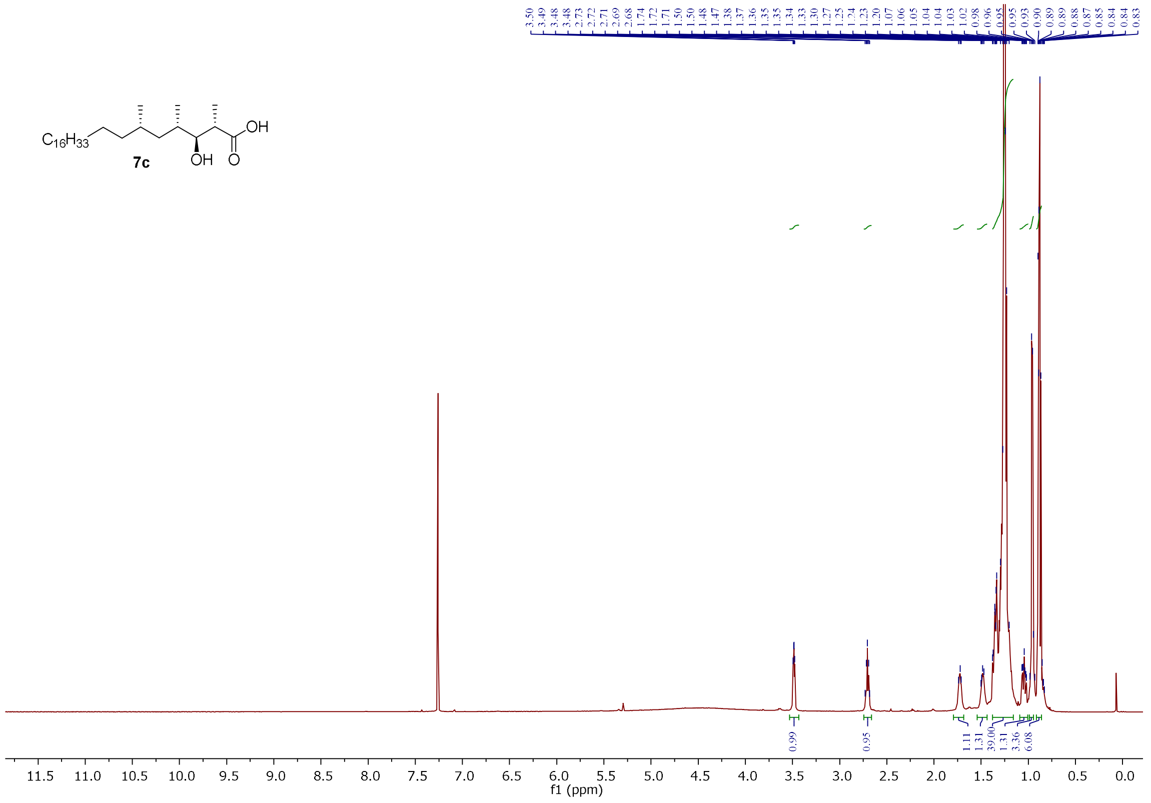

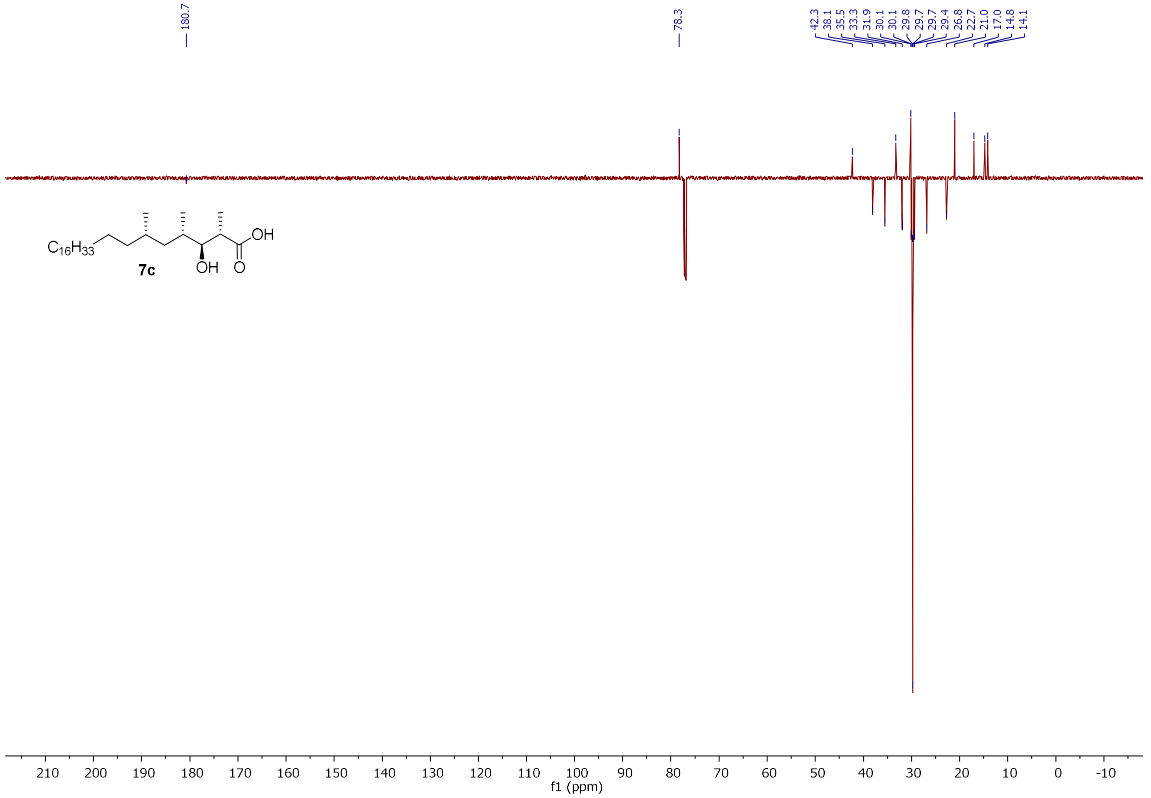

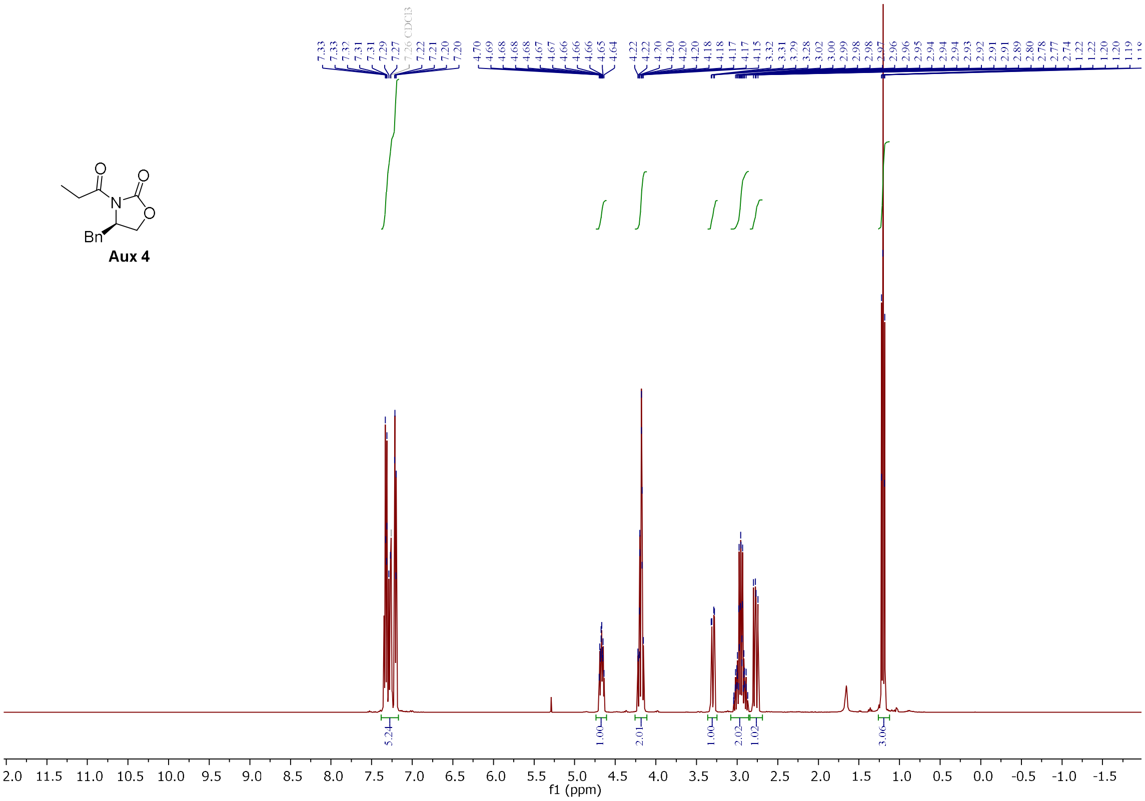
O
O
N
O
Bn
Aux 4
CDCl3
5.24
1.00
2.01
1.00
2.02
1.02
3.06
f1 (ppm)

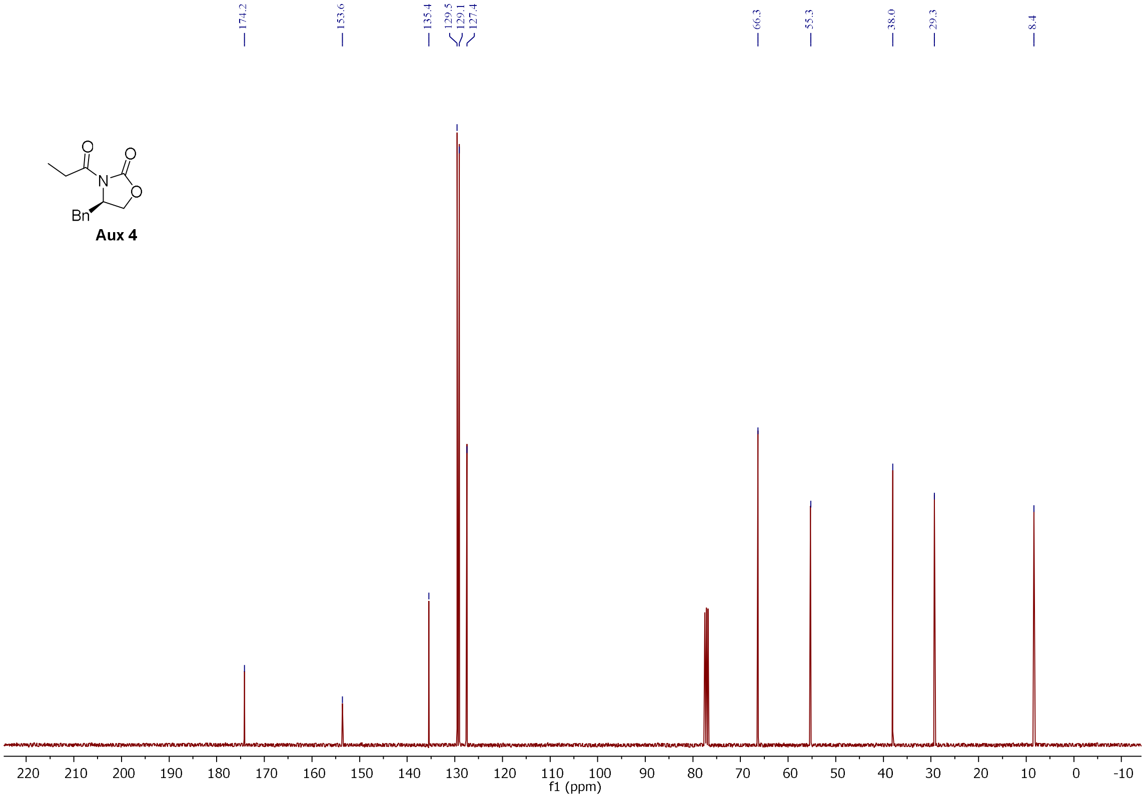
174.2
153.6
135.4
129.5
129.1
127.4
66.3
55.3
38.0
29.3
8.4
O
O
N
O
Bn
Aux 4
f1 (ppm)

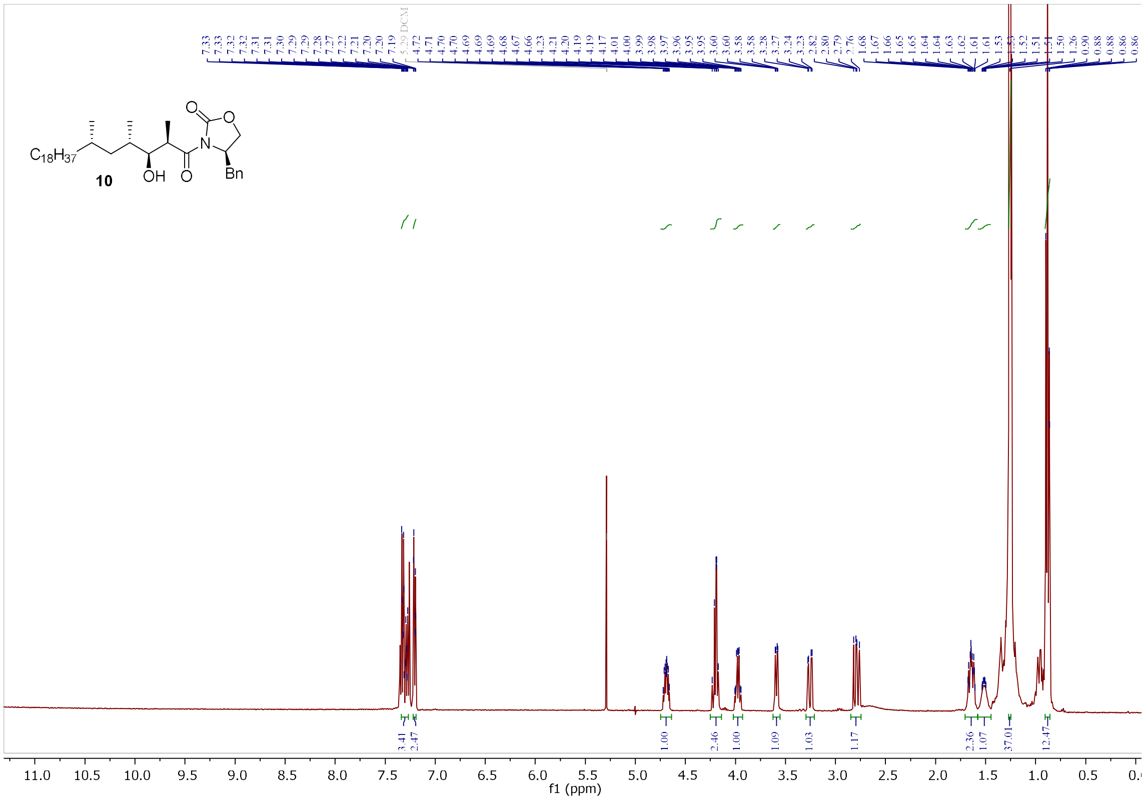

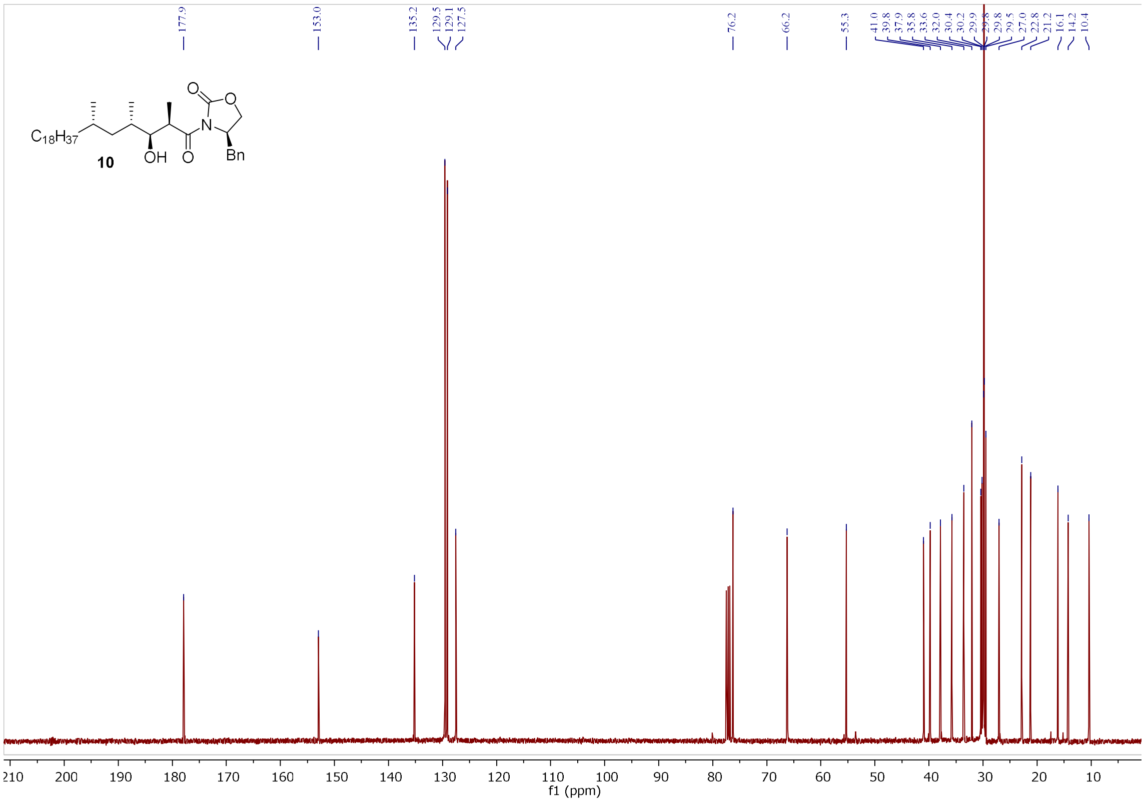

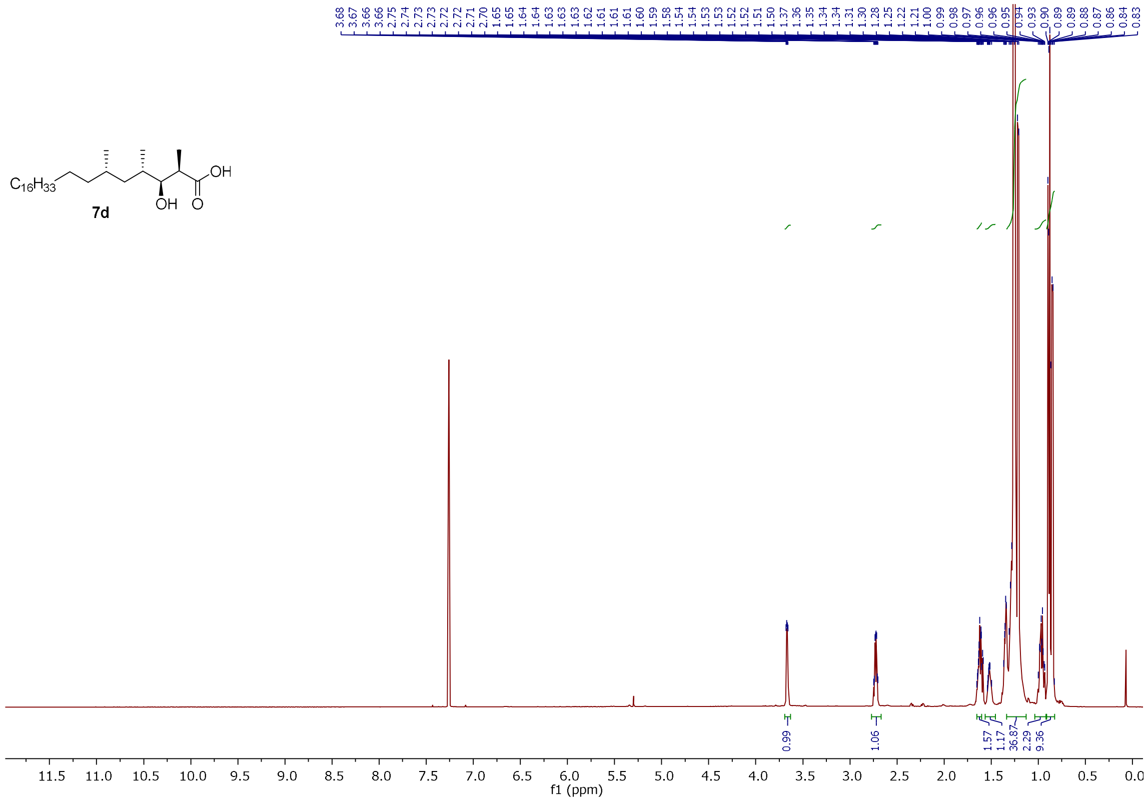
3.68 3.67 3.66 2.75 2.74 2.73 2.73 2.72 2.72 2.71 2.70 1.65 1.64 1.64 1.63 1.63 1.63 1.62 1.61 1.61 1.61 1.60 1.59 1.58 1.54 1.54 1.53 1.53 1.53 1.52 1.52 1.51 1.50 1.37 1.36 1.35 1.34 1.34 1.31 1.30 1.28 1.25 1.22 1.21 1.00 0.99 0.98 0.97 0.96 0.96 0.95 0.94 0.93 0.90 0.89 0.89 0.88 0.87 0.86 0.84 0.83
$C_{16}H_{33}$
OH
OH
O
7d
0.99
1.06
1.57
1.17
36.87
2.29
9.36
f1 (ppm)

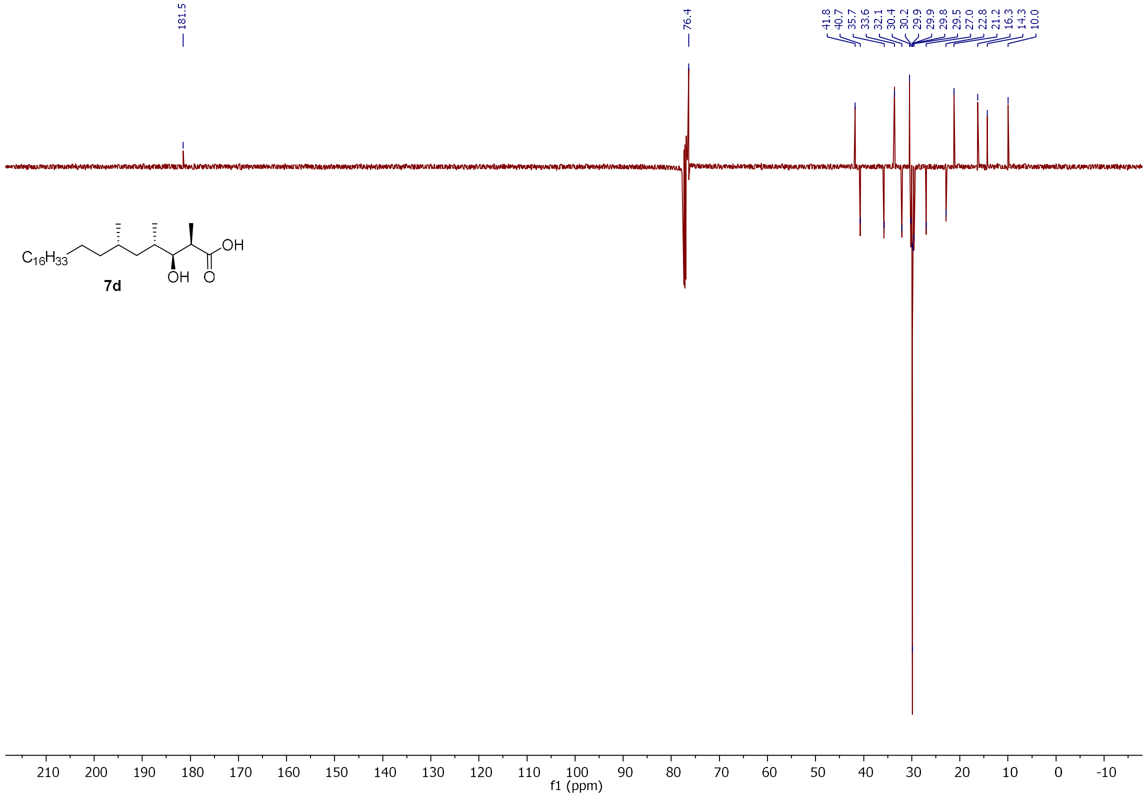
181.5
76.4
41.8 40.7 35.7 33.6 32.1 30.4 30.2 29.9 29.9 29.8 29.5 27.0 22.8 21.2 16.3 14.3 10.0
$C_{16}H_{33}$
OH
OH
O
7d
f1 (ppm)

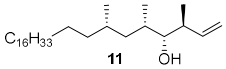

C16H33
OH

11

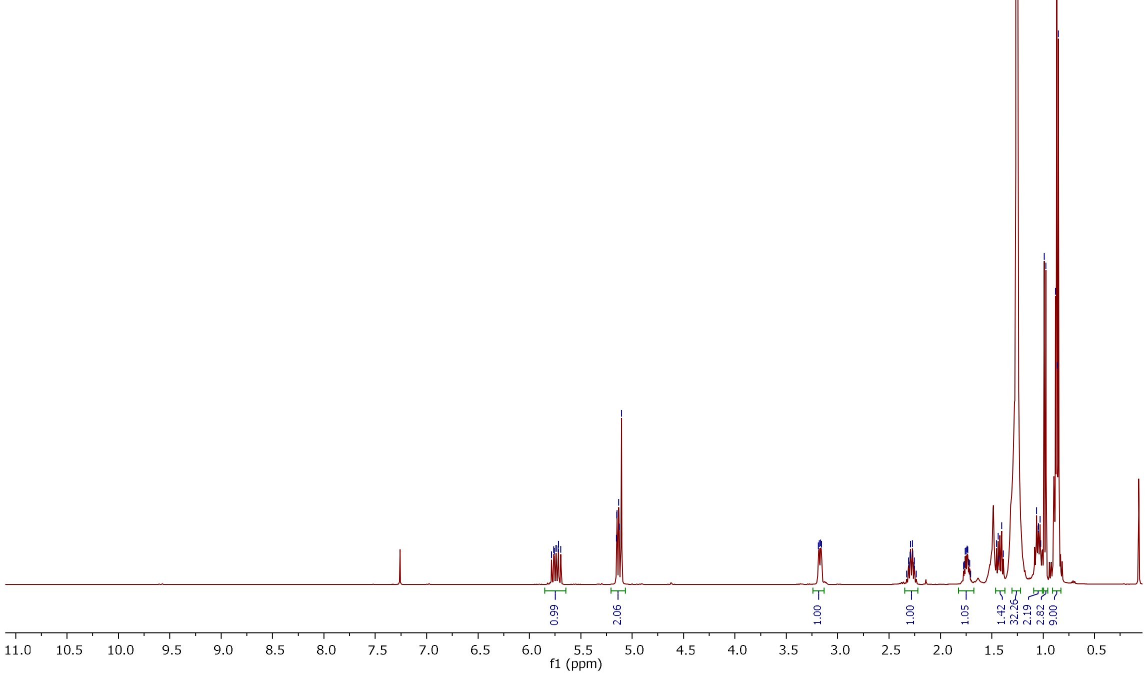

f1 (ppm)

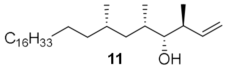

C16H33
OH

11

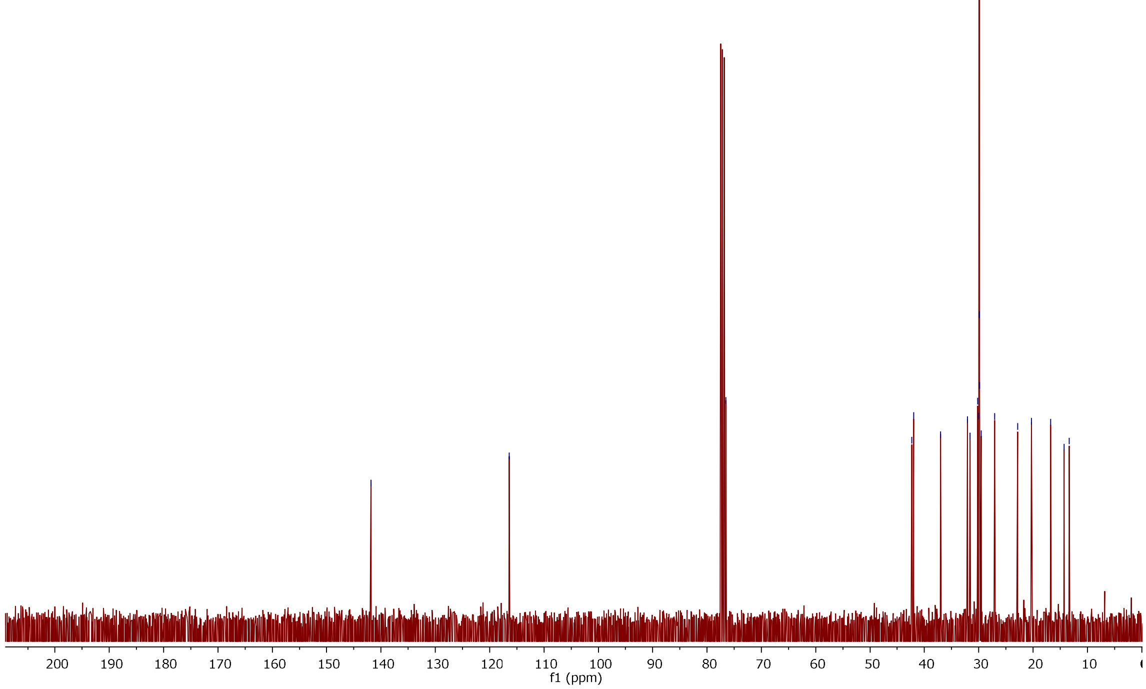

141.8
116.4
76.6
42.3
42.0
37.0
32.1
31.6
30.0
29.9
29.9
29.8
29.5
27.1
22.8
20.3
16.8
14.3
13.3
f1 (ppm)

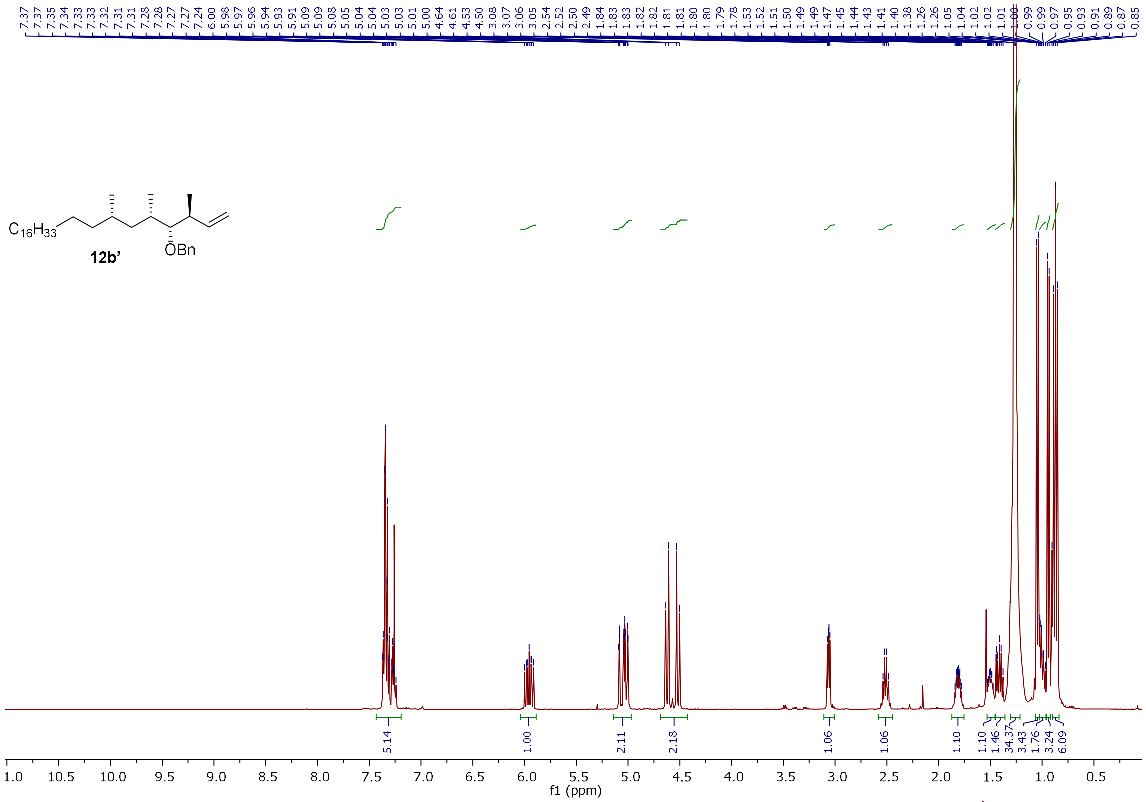

12b'

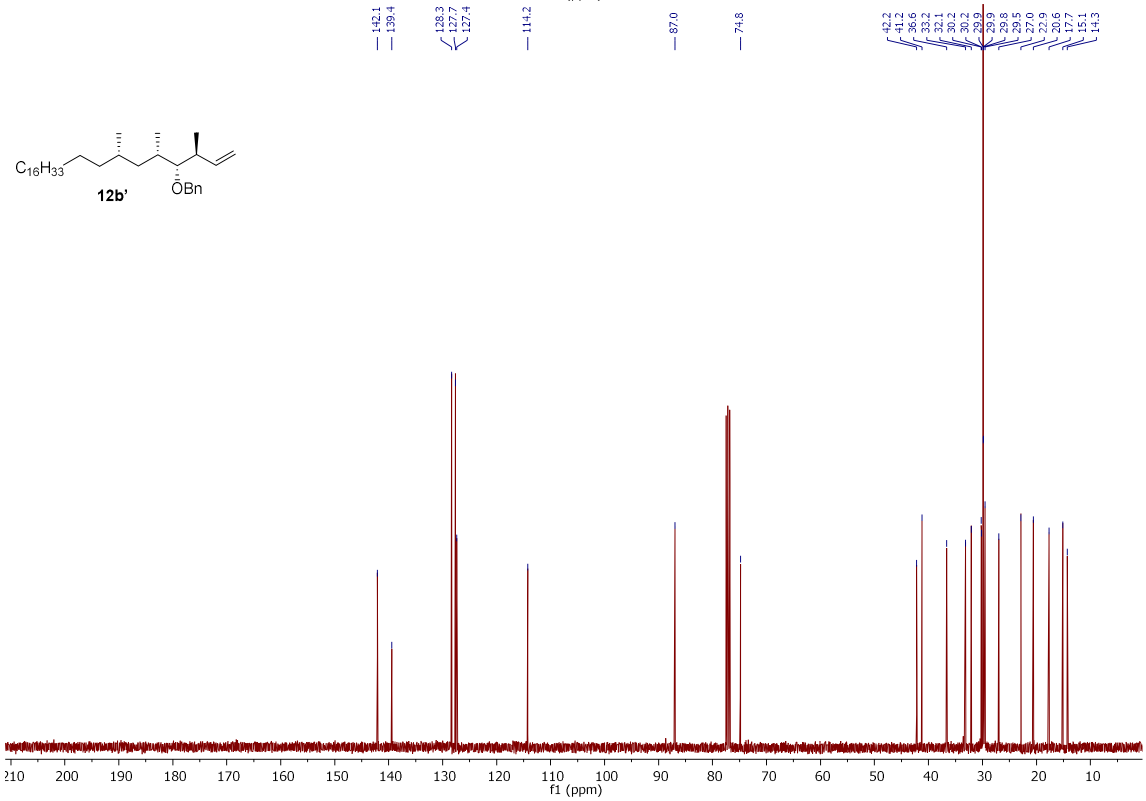

12b'

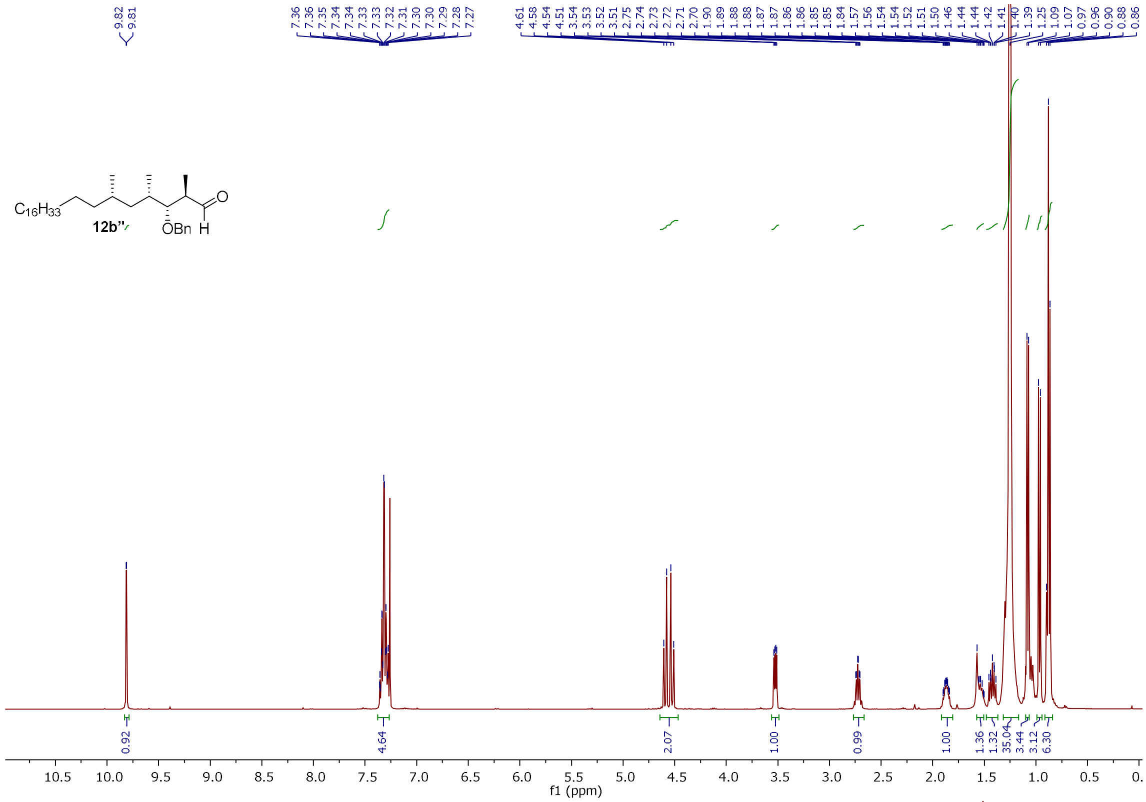

9.82
9.81
7.36
7.36
7.35
7.34
7.34
7.33
7.33
7.32
7.31
7.30
7.30
7.29
7.28
7.27
4.61
4.58
4.54
4.51
3.54
3.53
3.52
3.51
2.75
2.74
2.73
2.72
2.71
2.70
1.90
1.89
1.88
1.88
1.87
1.87
1.86
1.86
1.85
1.85
1.84
1.57
1.56
1.54
1.54
1.52
1.51
1.50
1.46
1.44
1.42
1.41
1.40
1.39
1.25
1.09
1.07
0.97
0.96
0.90
0.88
0.86
C16H33
12b''
OBn
H
O
0.92
4.64
2.07
1.00
0.99
1.00
1.36
1.32
35.04
3.44
3.12
6.30
f1 (ppm)

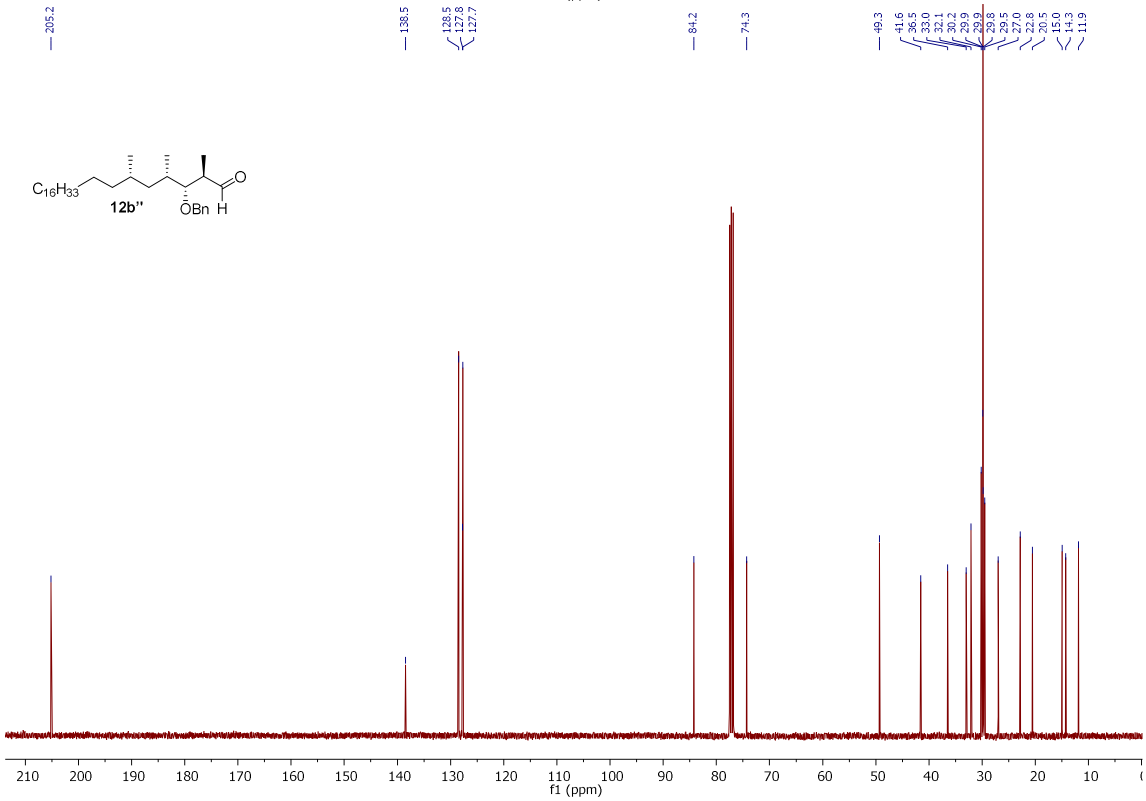

205.2
138.5
128.5
127.8
127.7
84.2
74.3
49.3
41.6
36.5
33.0
32.1
30.2
29.9
29.8
29.5
27.0
22.8
20.5
15.0
14.3
11.9
C16H33
12b''
OBn
H
O
f1 (ppm)

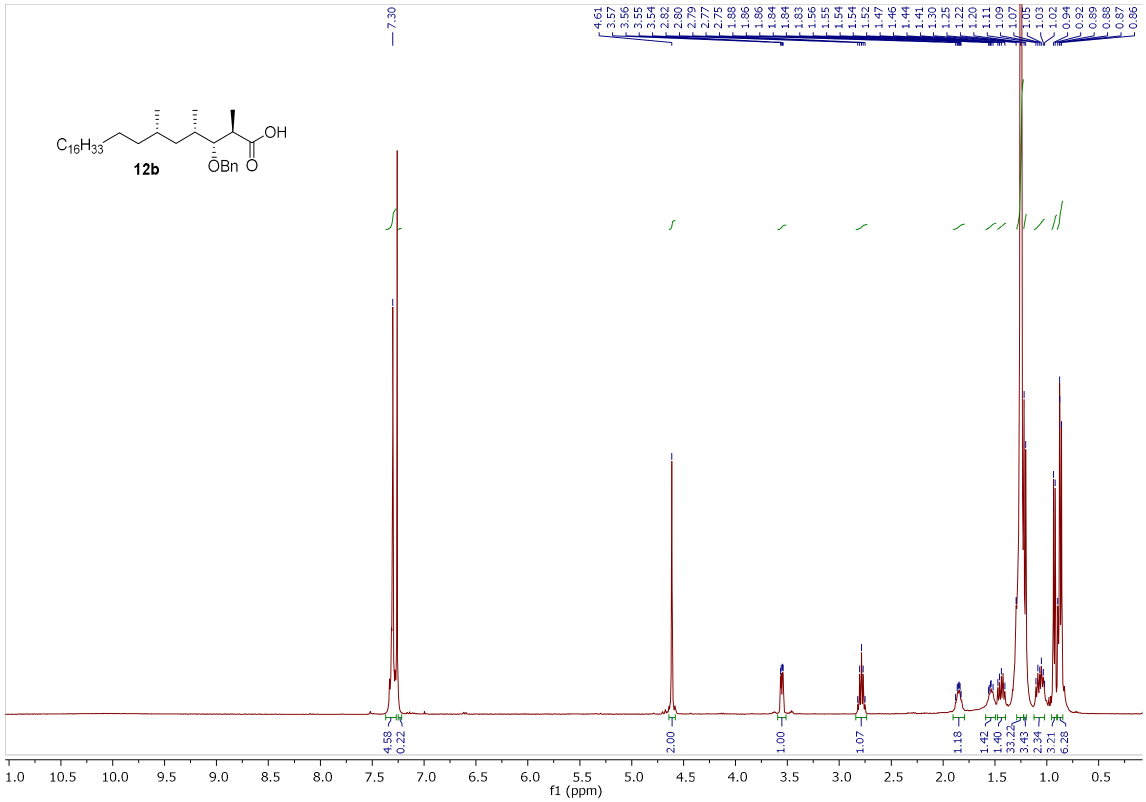

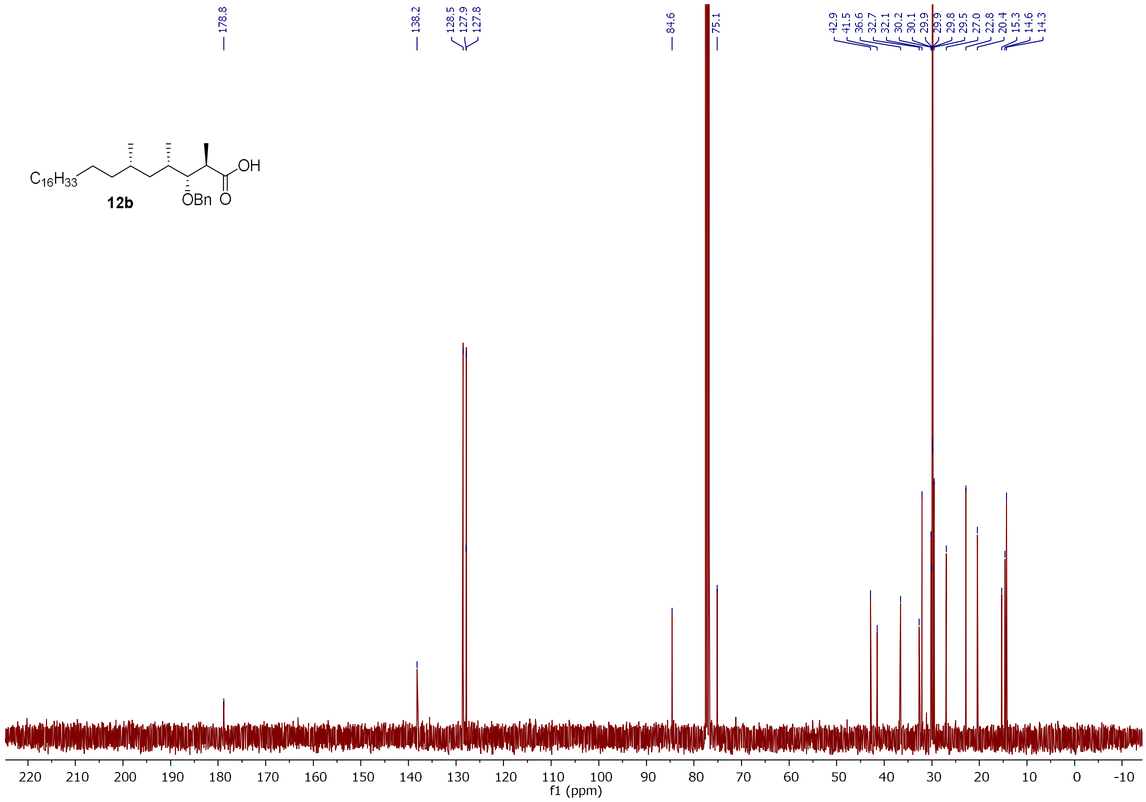

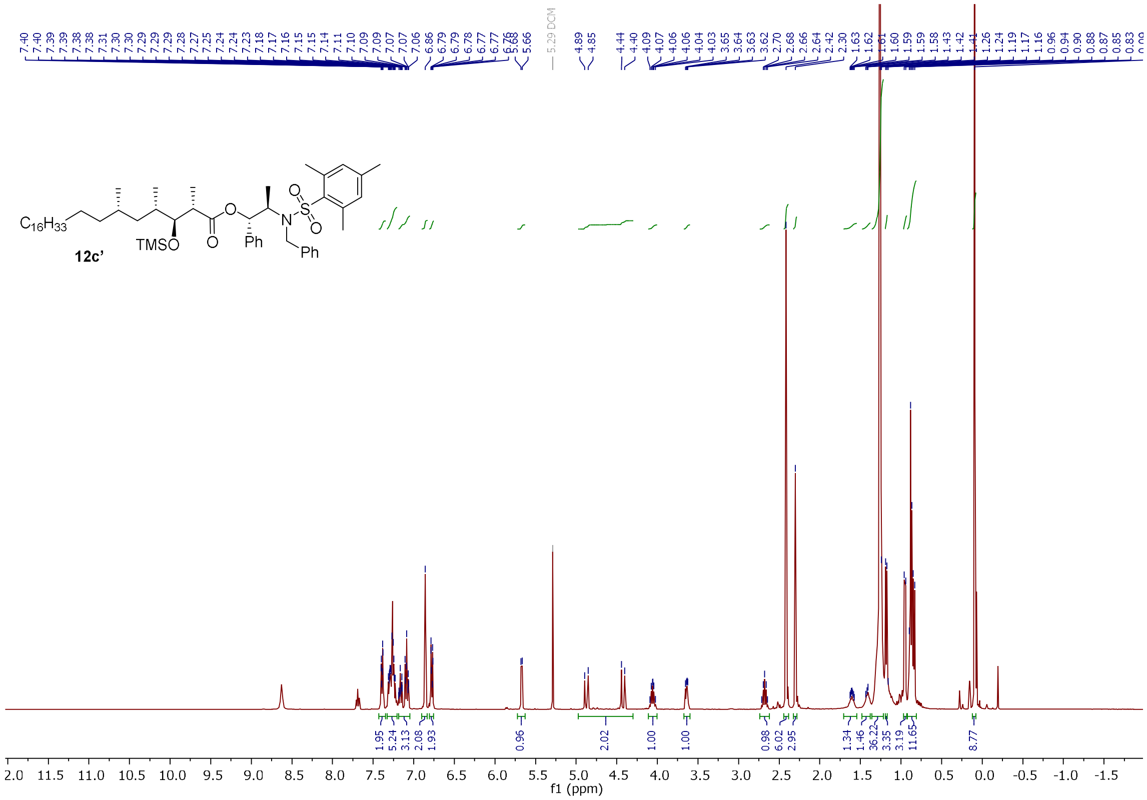

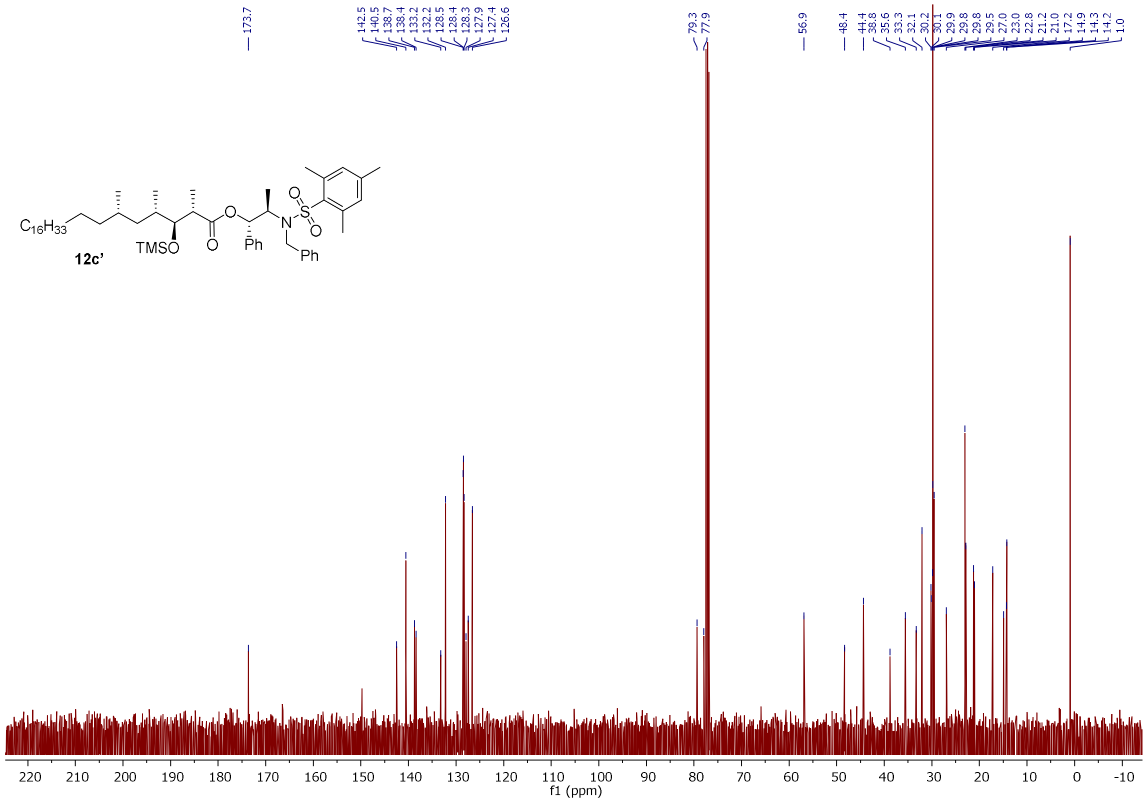

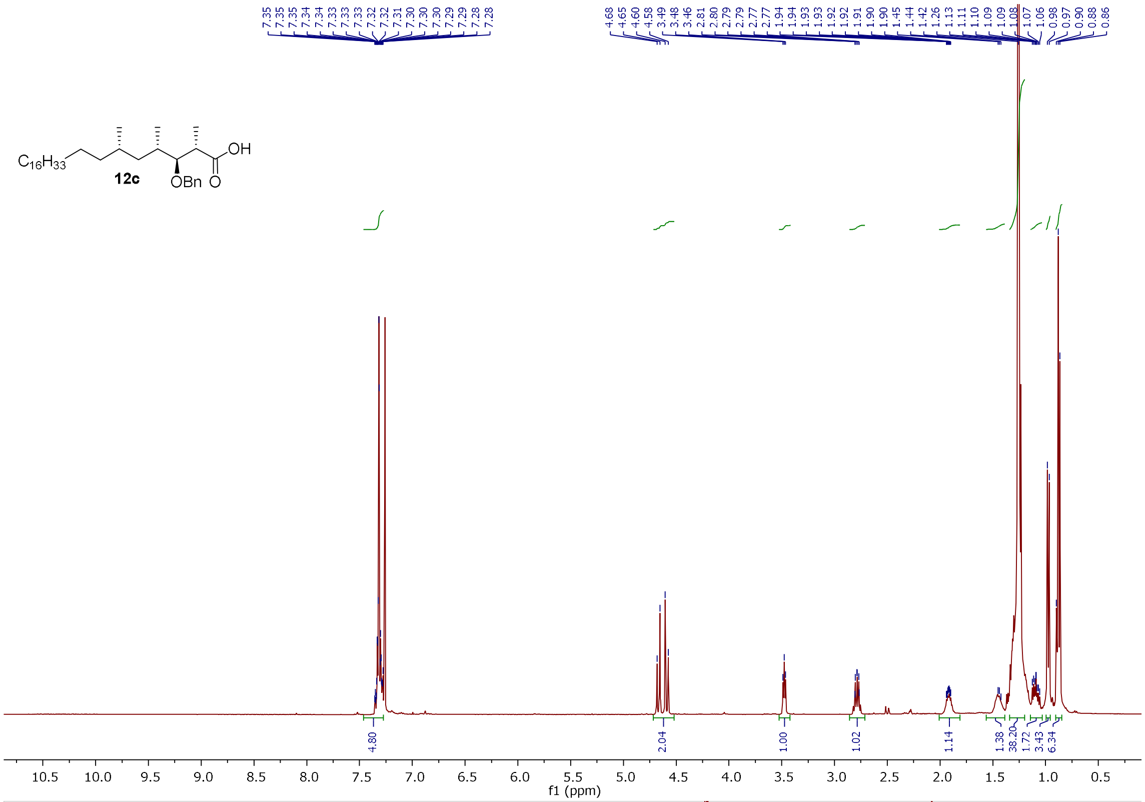

7.35 7.35 7.34 7.34 7.33 7.33 7.33 7.32 7.32 7.31 7.30 7.30 7.30 7.29 7.29 7.28 7.28
4.68 4.65 4.60 4.58 3.49 3.48 3.46 2.81 2.80 2.79 2.79 2.77 1.94 1.94 1.93 1.93 1.92 1.92 1.91 1.90 1.90 1.45 1.44 1.42 1.26 1.13 1.11 1.10 1.09 1.09 1.08 1.07 1.06 0.98 0.97 0.90 0.88 0.86
C16H33
12c
OBn
OH
O
4.80
2.04
1.00
1.02
1.14
1.38
38.20
1.72
3.43
6.34
f1 (ppm)

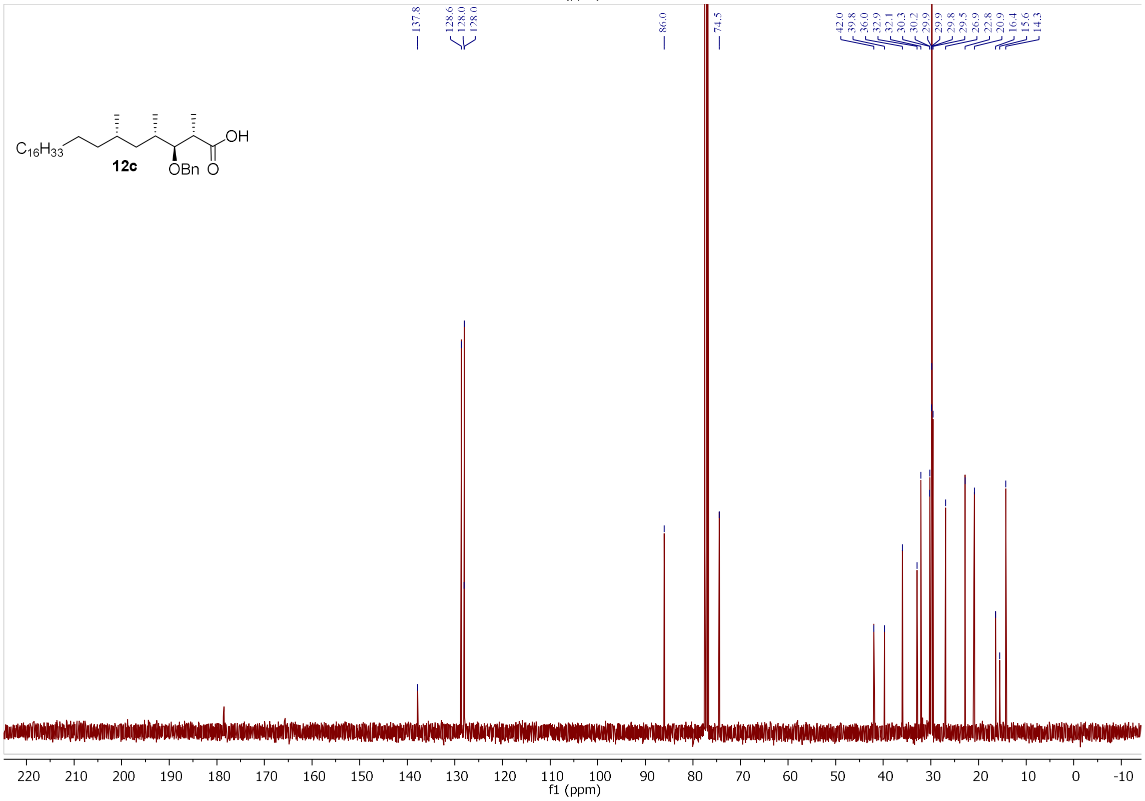

137.8
128.6 128.0 128.0
86.0
74.5
42.0 39.8 36.0 32.9 32.1 30.3 30.2 29.9 29.8 29.5 26.9 22.8 20.9 16.4 15.6 14.3
C16H33
12c
OBn
OH
O
f1 (ppm)

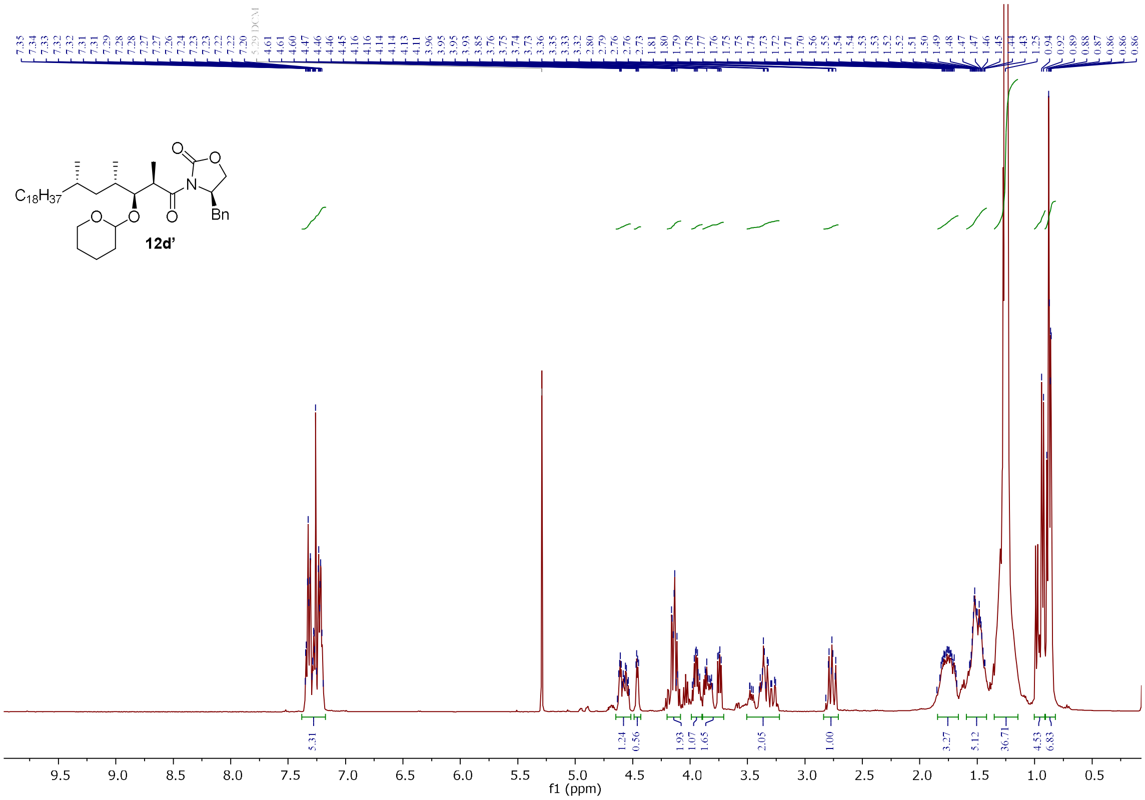

C18H37
Bn
12d'
f1 (ppm)

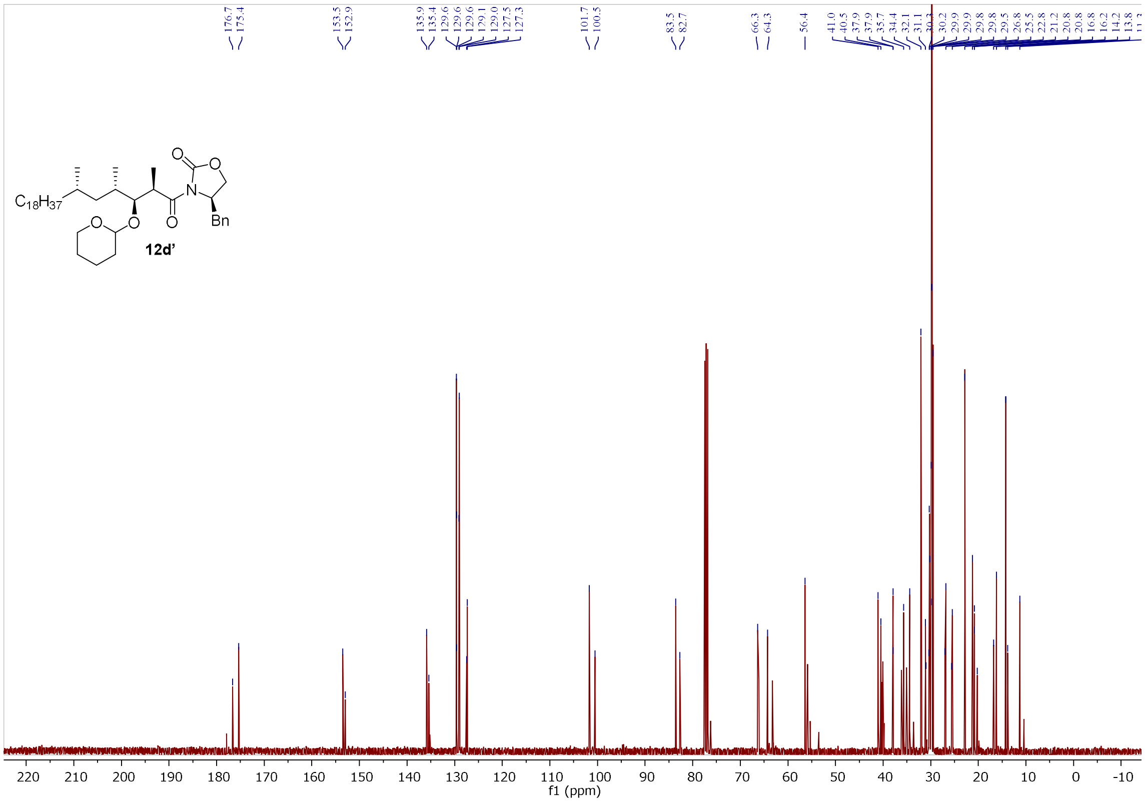

C18H37
Bn
12d'
f1 (ppm)

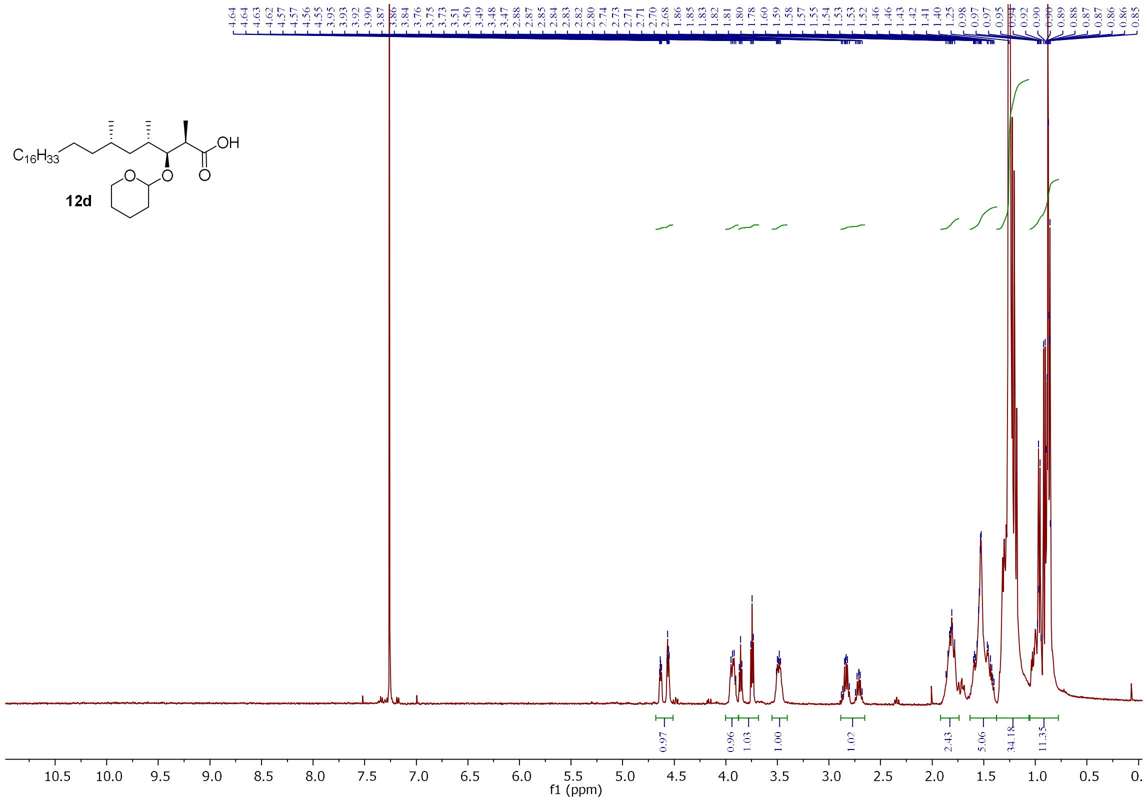

12d

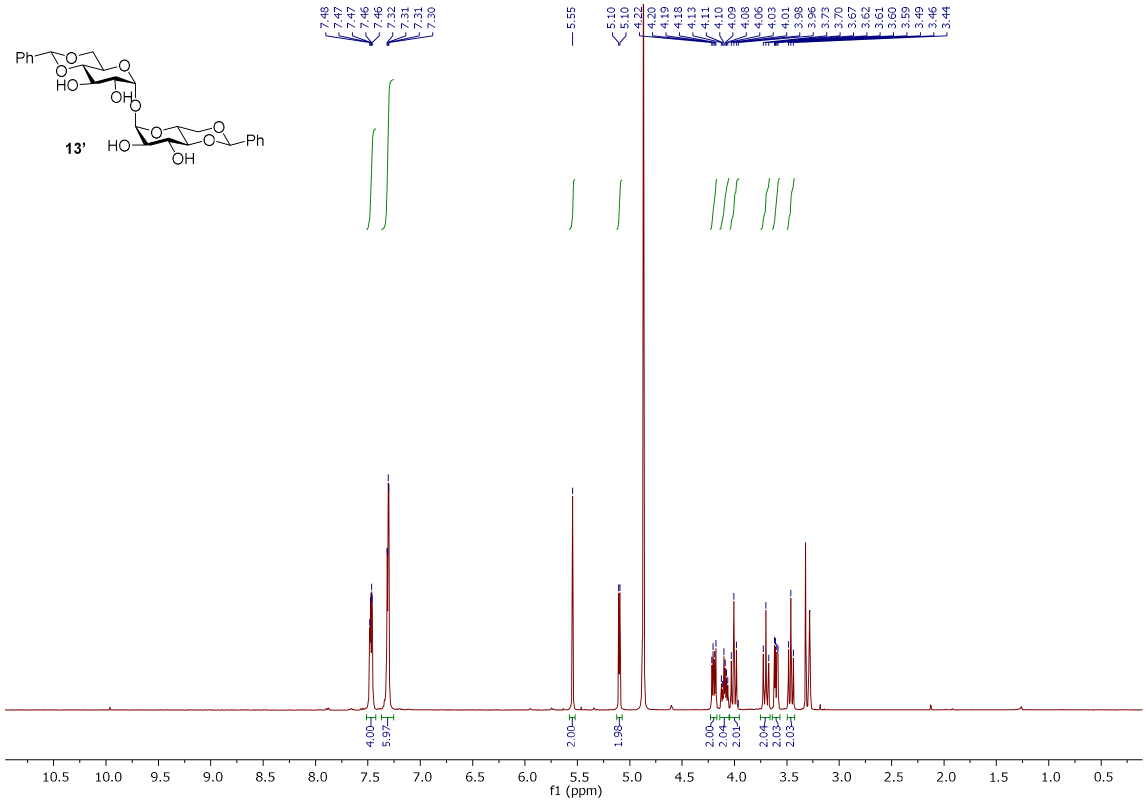

13'
Ph
HO
OH
HO
OH
Ph
7.48 7.47 7.47 7.46 7.46 7.32 7.31 7.31 7.30
5.55
5.10 5.10
4.22 4.20 4.19 4.18 4.13 4.11 4.11 4.10 4.09 4.08 4.06 4.03 4.01 3.98 3.96 3.73 3.70 3.67 3.62 3.61 3.60 3.59 3.49 3.46 3.44
4.00 5.97 2.00 1.98 2.00 2.04 2.01 2.04 2.03 2.03
f1 (ppm)

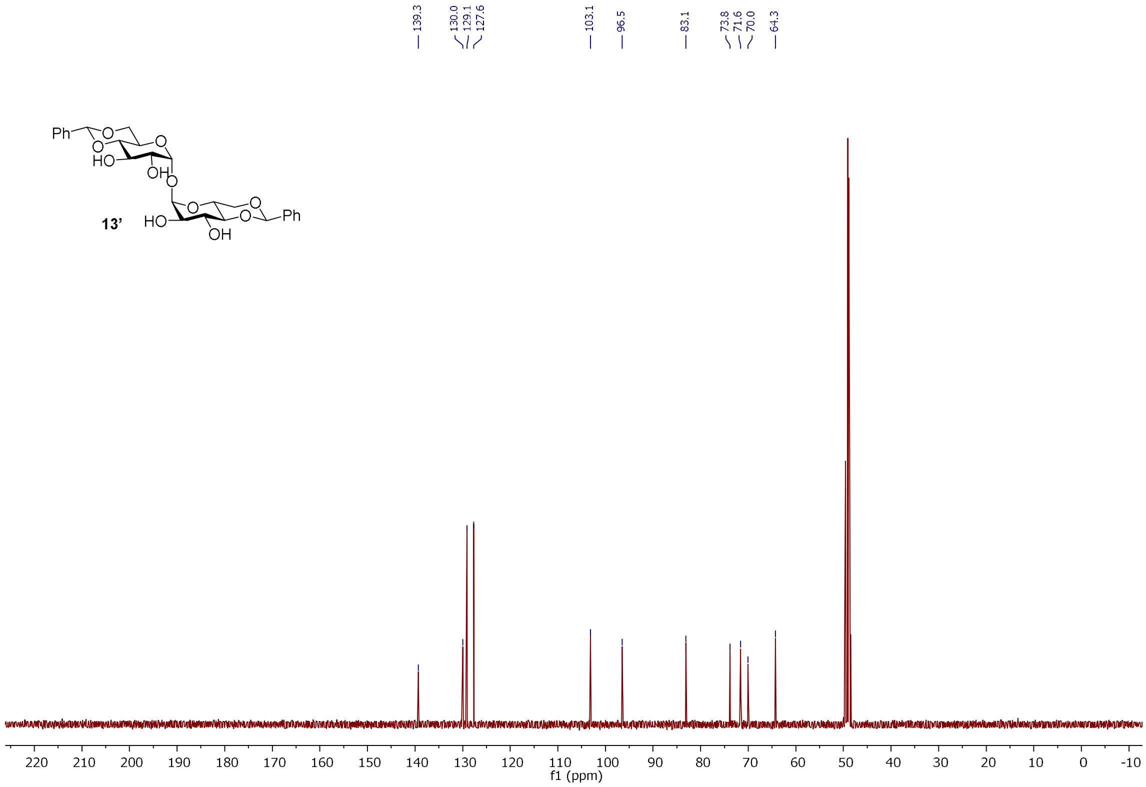

13'
Ph
HO
OH
HO
OH
Ph
139.3
130.0 129.1 127.6
103.1
96.5
83.1
73.8 71.6 70.0
64.3
f1 (ppm)

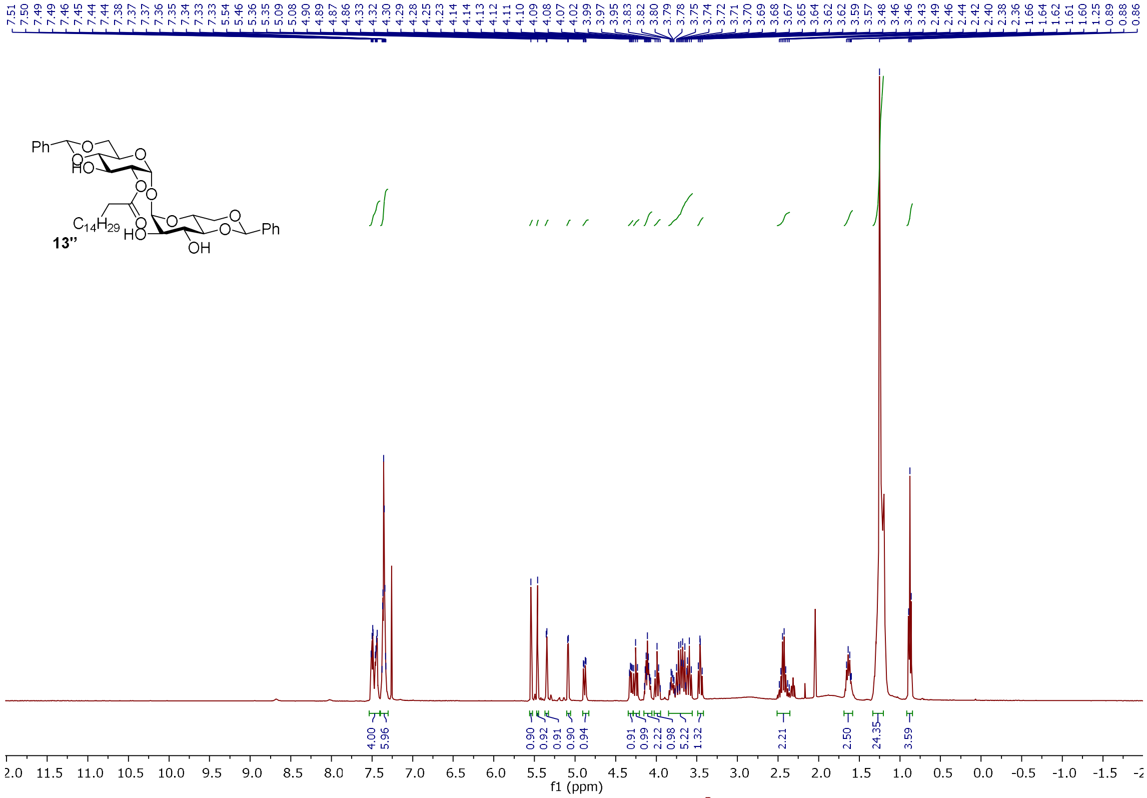
7.51 7.50 7.49 7.49 7.46 7.45 7.44 7.44 7.38 7.37 7.37 7.36 7.35 7.34 7.33 7.33 5.54 5.46 5.36 5.35 5.09 5.08 4.90 4.89 4.87 4.86 4.33 4.32 4.30 4.29 4.28 4.25 4.23 4.14 4.14 4.13 4.12 4.11 4.10 4.09 4.08 4.07 4.02 3.99 3.97 3.95 3.83 3.82 3.80 3.79 3.78 3.76 3.75 3.74 3.72 3.71 3.70 3.69 3.68 3.67 3.65 3.64 3.62 3.62 3.59 3.57 3.48 3.46 3.46 3.43 2.49 2.46 2.44 2.42 2.40 2.38 2.36 1.66 1.64 1.62 1.61 1.60 1.25 0.89 0.88 0.86
Ph
HO
O
$C_{14}H_{29}$
HO
OH
Ph
13''
4.00 5.96 0.90 0.92 0.91 0.90 0.94 0.91 0.99 2.22 0.98 5.22 1.32 2.21 2.50 24.35 3.59
f1 (ppm)

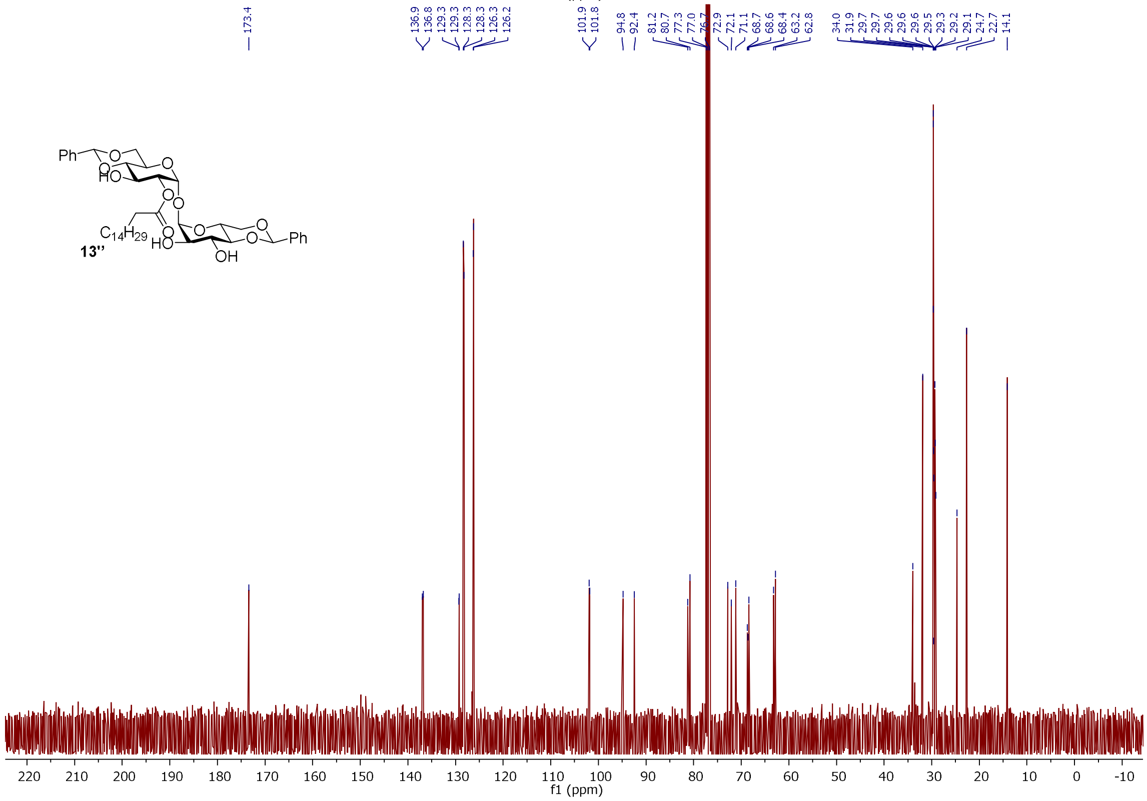
173.4 136.9 136.8 129.3 129.3 128.3 128.3 126.3 126.2 101.9 101.8 94.8 92.4 81.2 80.7 77.3 77.0 76.7 72.9 72.1 71.1 68.7 68.6 68.4 63.2 62.8 34.0 31.9 29.7 29.6 29.6 29.6 29.5 29.3 29.2 29.1 24.7 22.7 14.1
Ph
HO
O
$C_{14}H_{29}$
HO
OH
Ph
13''
f1 (ppm)

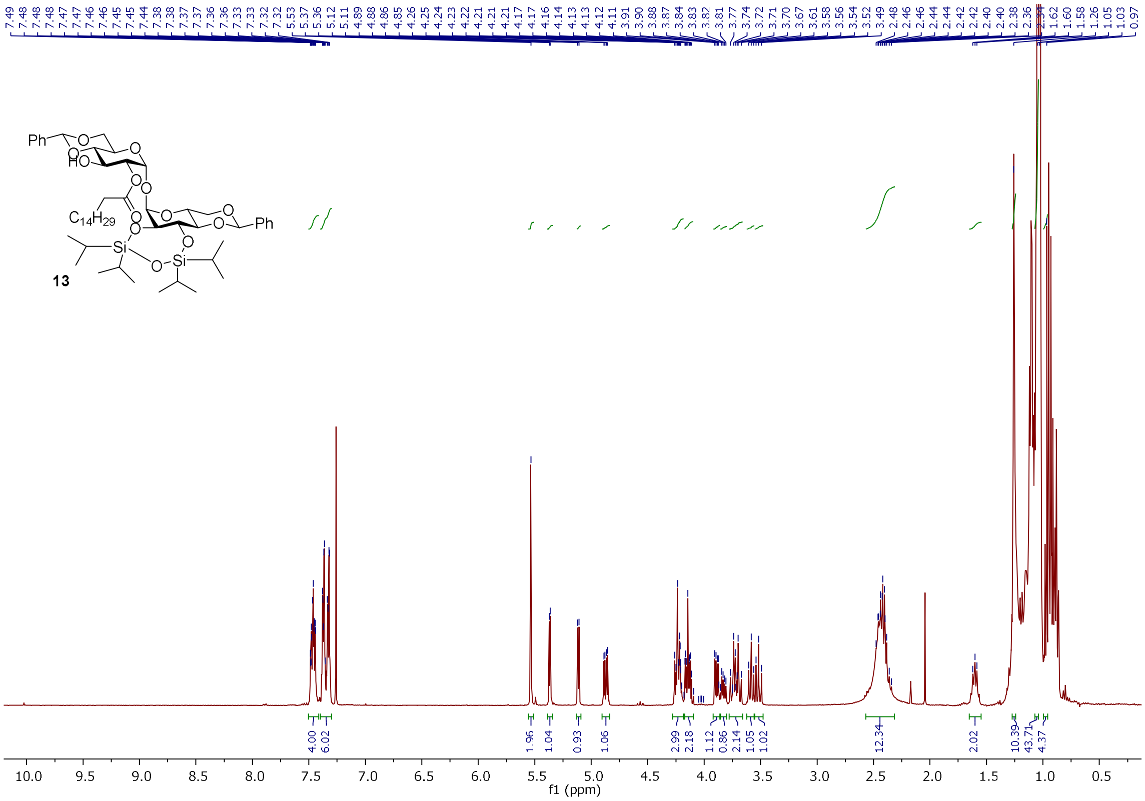
7.49 7.48 7.48 7.47 7.46 7.45 7.45 7.44 7.38 7.38 7.37 7.37 7.36 7.36 7.33 7.33 7.32 7.32 5.53 5.37 5.36 5.12 5.11 4.89 4.88 4.86 4.85 4.26 4.25 4.24 4.23 4.22 4.21 4.21 4.21 4.21 4.17 4.17 4.16 4.14 4.13 4.13 4.13 4.12 4.11 3.91 3.90 3.88 3.87 3.84 3.83 3.82 3.81 3.77 3.74 3.72 3.71 3.70 3.67 3.61 3.58 3.56 3.54 3.52 3.49 2.48 2.46 2.46 2.44 2.44 2.42 2.42 2.40 2.40 2.38 2.36 1.62 1.60 1.58 1.26 1.05 1.03 0.97
Ph
O
O
HO
O
O
C14H29
O
O
O
O
Ph
O
O
Si
O
Si
13
4.00 6.02 1.96 1.04 0.93 1.06 2.99 2.18 1.12 0.86 2.14 1.05 1.02 12.34 2.02 10.39 43.71 4.37
10.0 9.5 9.0 8.5 8.0 7.5 7.0 6.5 6.0 5.5 5.0 4.5 4.0 3.5 3.0 2.5 2.0 1.5 1.0 0.5
f1 (ppm)

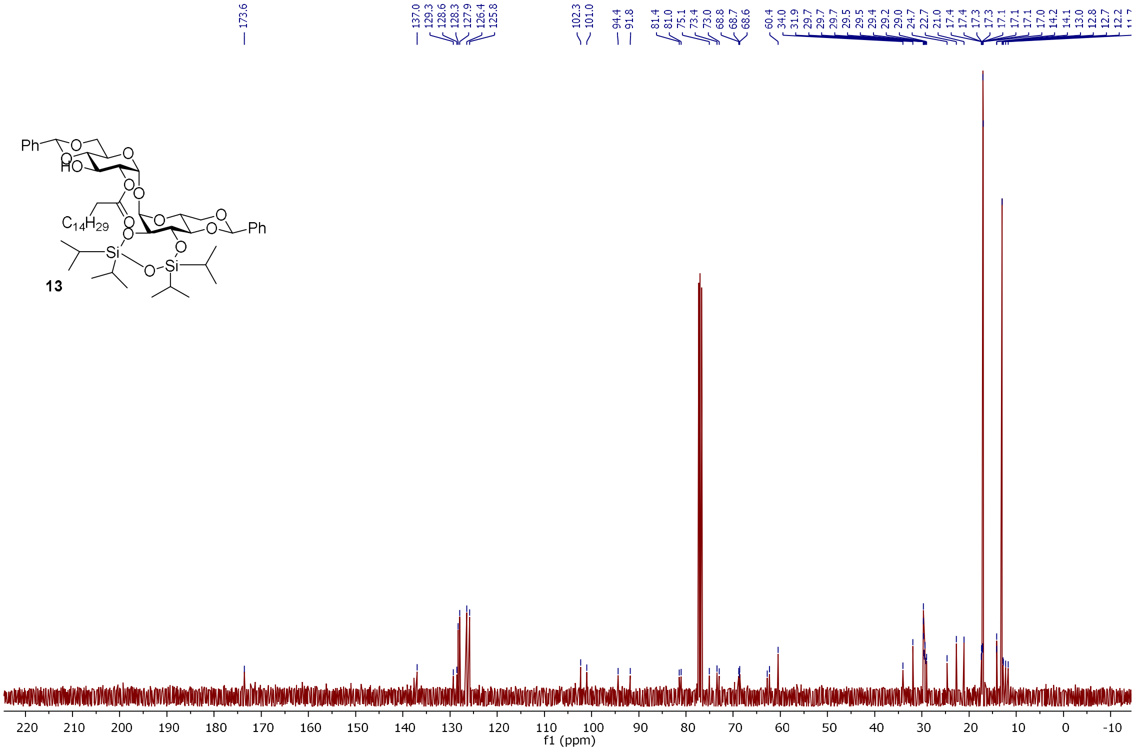
173.6
137.0 129.3 128.6 128.3 127.9 126.4 125.8
102.3 101.0
94.4 91.8
81.4 81.0 75.1 73.4 73.0 68.8 68.7 68.6
60.4 34.0 31.9 29.7 29.7 29.7 29.5 29.4 29.2 29.0 24.7 22.7 21.0 17.4 17.4 17.3 17.3 17.1 17.1 17.1 17.0 14.2 14.1 13.0 12.8 12.7 12.2
Ph
O
O
HO
O
O
C14H29
O
O
O
O
Ph
O
O
Si
O
Si
13
220 210 200 190 180 170 160 150 140 130 120 110 100 90 80 70 60 50 40 30 20 10 0 -10
f1 (ppm)

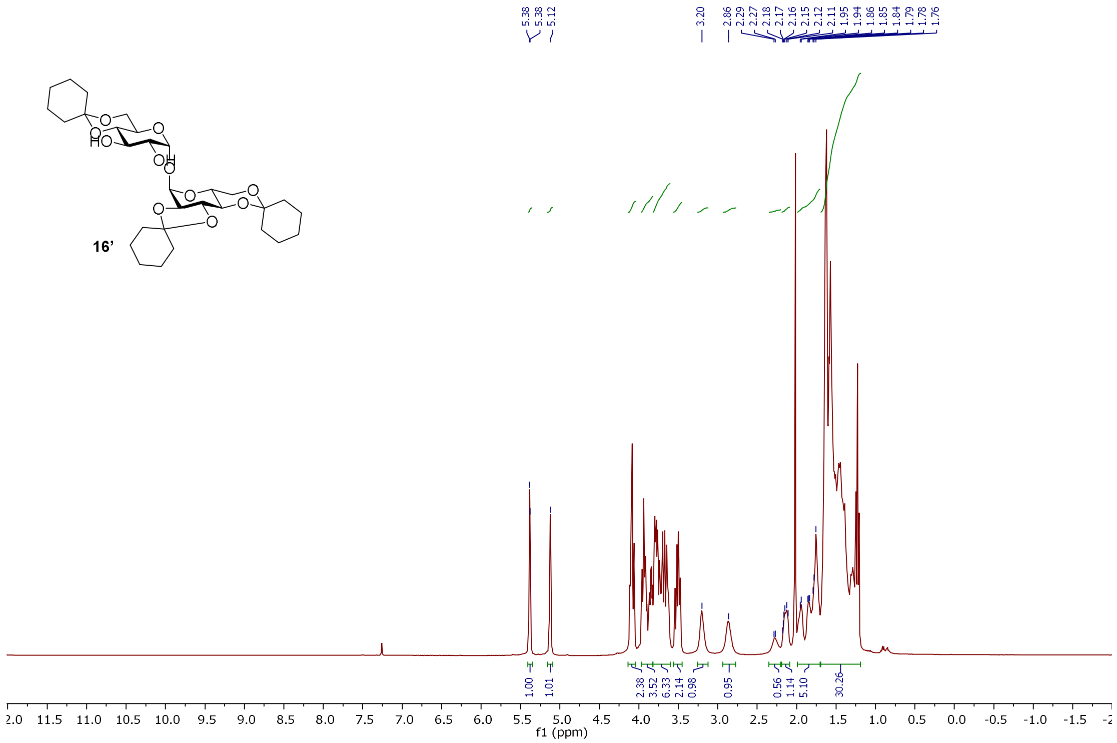
5.38
5.38
5.12
3.20
2.86
2.29
2.27
2.18
2.17
2.16
2.15
2.12
2.11
1.95
1.94
1.86
1.85
1.84
1.79
1.78
1.76
HO
OH
16'
1.00
1.01
2.38
3.52
6.33
2.14
0.98
0.95
0.56
1.14
5.10
30.26
f1 (ppm)

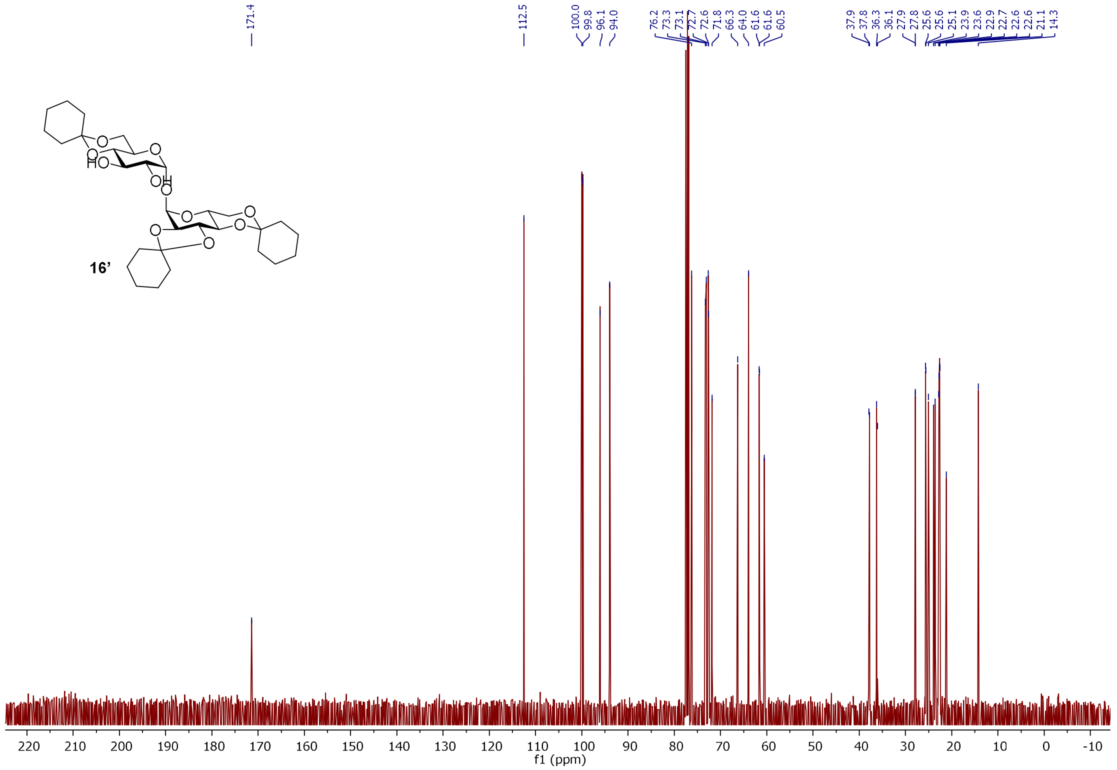
171.4
112.5
100.0
99.8
96.1
94.0
76.2
73.3
73.1
72.7
72.6
71.8
66.3
64.0
61.6
61.6
60.5
37.9
37.8
36.3
36.1
27.9
27.8
25.6
25.6
25.1
23.9
23.6
22.9
22.7
22.6
21.1
14.3
HO
OH
16'
f1 (ppm)

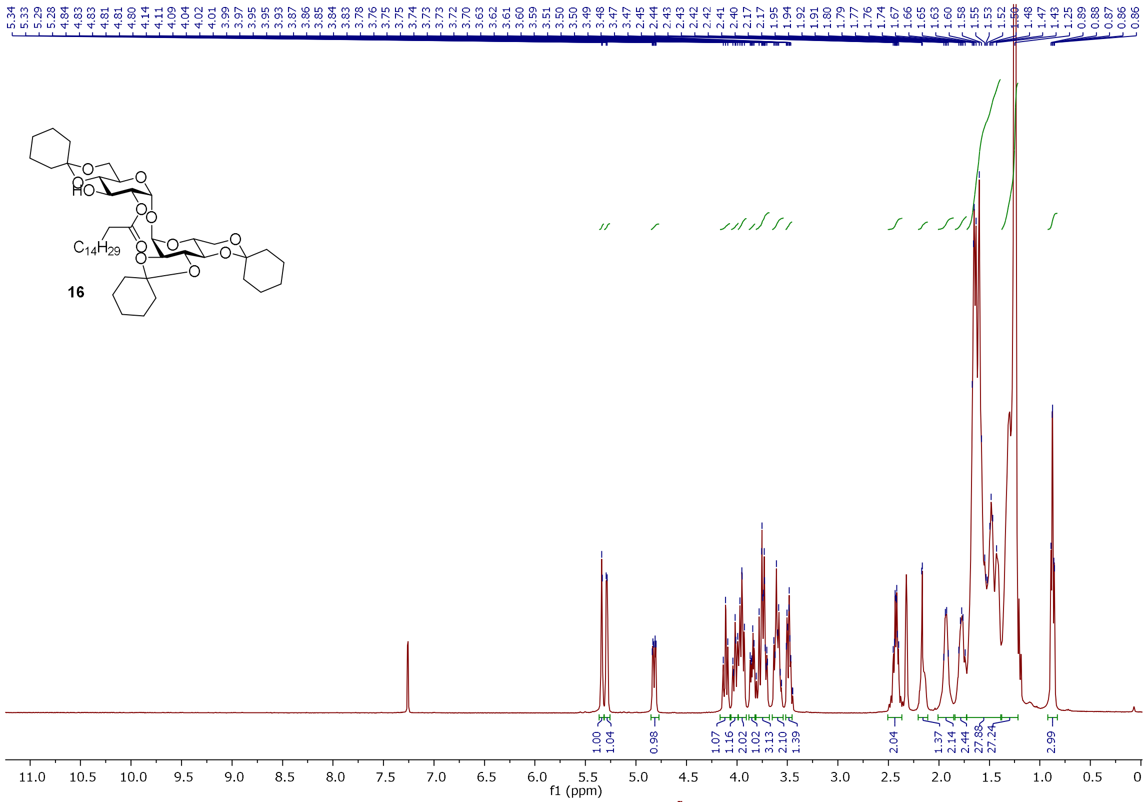

5.34 5.33 5.29 5.28 4.84 4.83 4.83 4.81 4.81 4.80 4.14 4.11 4.09 4.04 4.02 4.01 3.99 3.97 3.95 3.95 3.93 3.87 3.86 3.85 3.84 3.83 3.78 3.76 3.75 3.75 3.74 3.73 3.73 3.72 3.70 3.63 3.62 3.61 3.60 3.59 3.51 3.50 3.50 3.49 3.48 3.47 3.47 2.45 2.44 2.43 2.43 2.42 2.41 2.40 2.17 2.17 2.17 1.95 1.94 1.92 1.91 1.80 1.79 1.77 1.76 1.74 1.67 1.66 1.65 1.63 1.60 1.58 1.55 1.53 1.52 1.50 1.48 1.47 1.43 1.25 0.89 0.88 0.87 0.86 0.86
HO
$C_{14}H_{29}$
16
1.00 1.04 0.98 1.07 1.16 2.02 1.02 3.13 2.10 1.39 2.04 1.37 2.14 2.44 27.88 27.24 2.99
11.0 10.5 10.0 9.5 9.0 8.5 8.0 7.5 7.0 6.5 6.0 5.5 5.0 4.5 4.0 3.5 3.0 2.5 2.0 1.5 1.0 0.5 0
f1 (ppm)

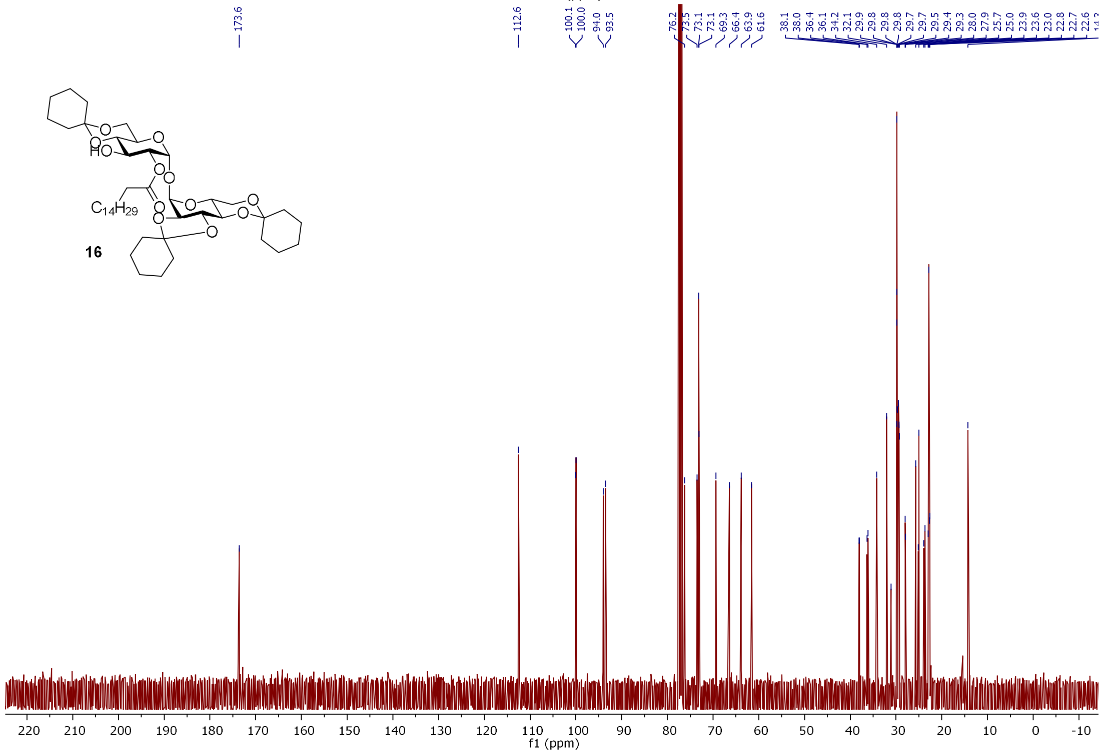

173.6 112.6 100.1 100.0 94.0 93.5 76.2 73.5 73.1 73.1 69.3 66.4 63.9 61.6 38.1 38.0 36.4 36.1 34.2 32.1 29.9 29.8 29.8 29.7 29.7 29.5 29.4 29.3 28.0 27.9 25.7 25.0 23.9 23.6 23.0 22.8 22.7 22.6
HO
$C_{14}H_{29}$
16
220 210 200 190 180 170 160 150 140 130 120 110 100 90 80 70 60 50 40 30 20 10 0 -10
f1 (ppm)

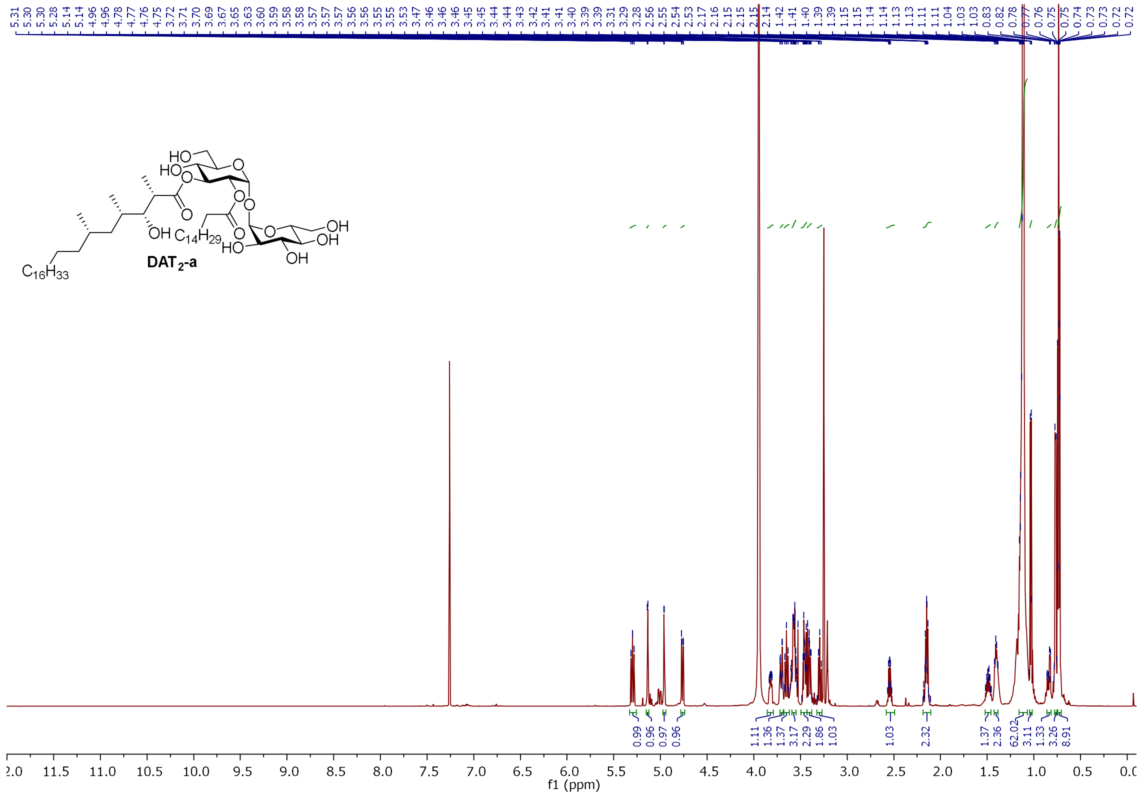

5.31 5.30 5.30 5.28 5.14 5.14 4.96 4.96 4.78 4.77 4.76 4.75 3.72 3.71 3.70 3.69 3.67 3.65 3.63 3.60 3.59 3.58 3.58 3.57 3.57 3.57 3.56 3.56 3.55 3.55 3.53 3.47 3.46 3.46 3.45 3.45 3.44 3.44 3.43 3.42 3.41 3.41 3.40 3.39 3.39 3.31 3.29 3.28 2.56 2.55 2.54 2.53 2.17 2.16 2.15 2.15 2.15 2.14 1.42 1.41 1.40 1.39 1.39 1.15 1.15 1.14 1.13 1.13 1.11 1.11 1.04 1.03 1.03 0.83 0.82 0.78 0.77 0.76 0.75 0.75 0.74 0.73 0.73 0.72 0.72
HO
HO
OH
C14H29
HO
OH
OH
OH
C16H33
DAT2-a
0.99 0.96 0.97 0.96 1.11 1.36 1.37 3.17 2.29 1.86 1.03 1.03 2.32 1.37 2.36 62.02 3.11 1.33 3.26 8.91
f1 (ppm)

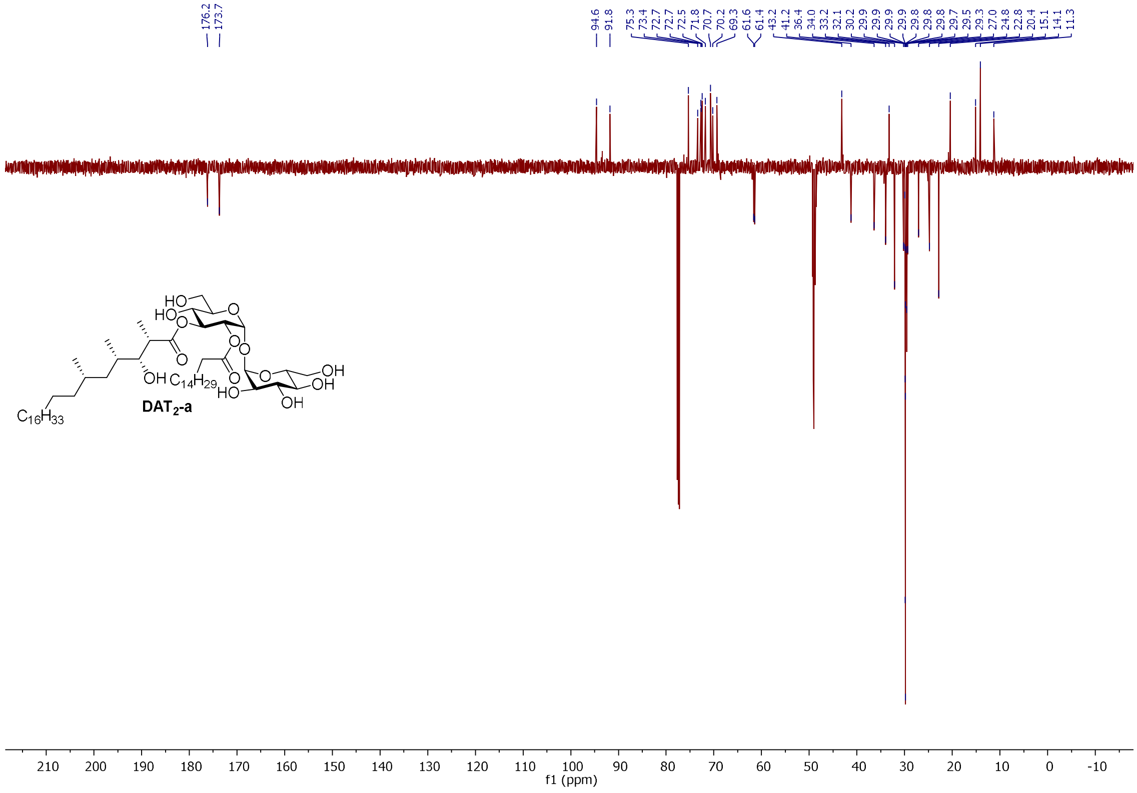

176.2 173.7 94.6 91.8 75.3 73.4 72.7 72.7 72.5 71.8 70.7 70.2 69.3 61.6 61.4 43.2 41.2 36.4 34.0 33.2 32.1 30.2 29.9 29.9 29.9 29.8 29.8 29.7 29.5 29.3 27.0 24.8 22.8 20.4 15.1 14.1 11.3
HO
HO
OH
C14H29
HO
OH
OH
OH
C16H33
DAT2-a
f1 (ppm)

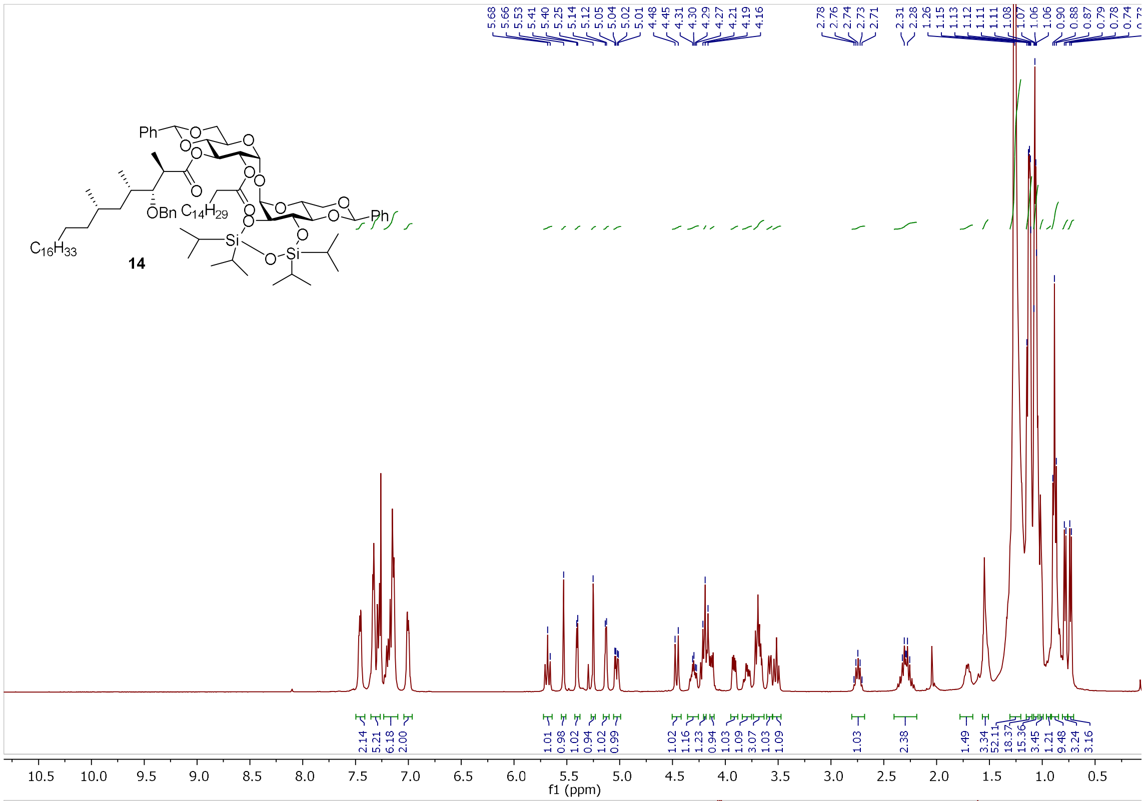

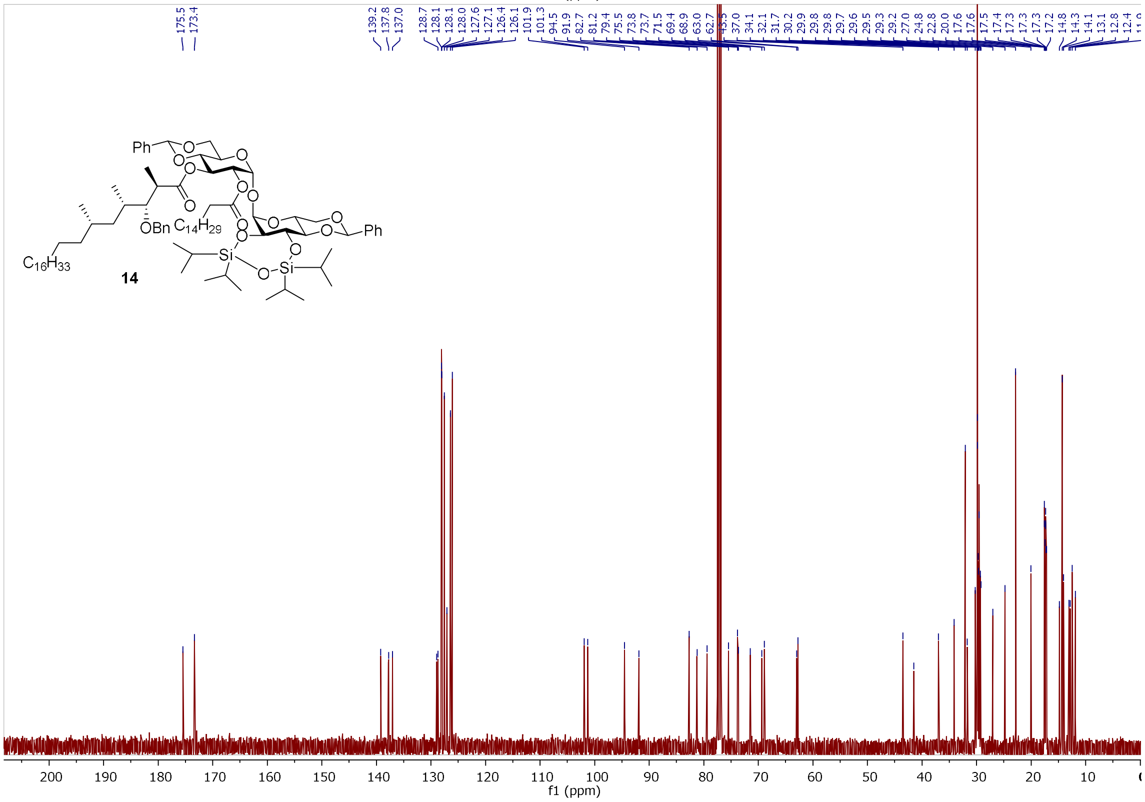

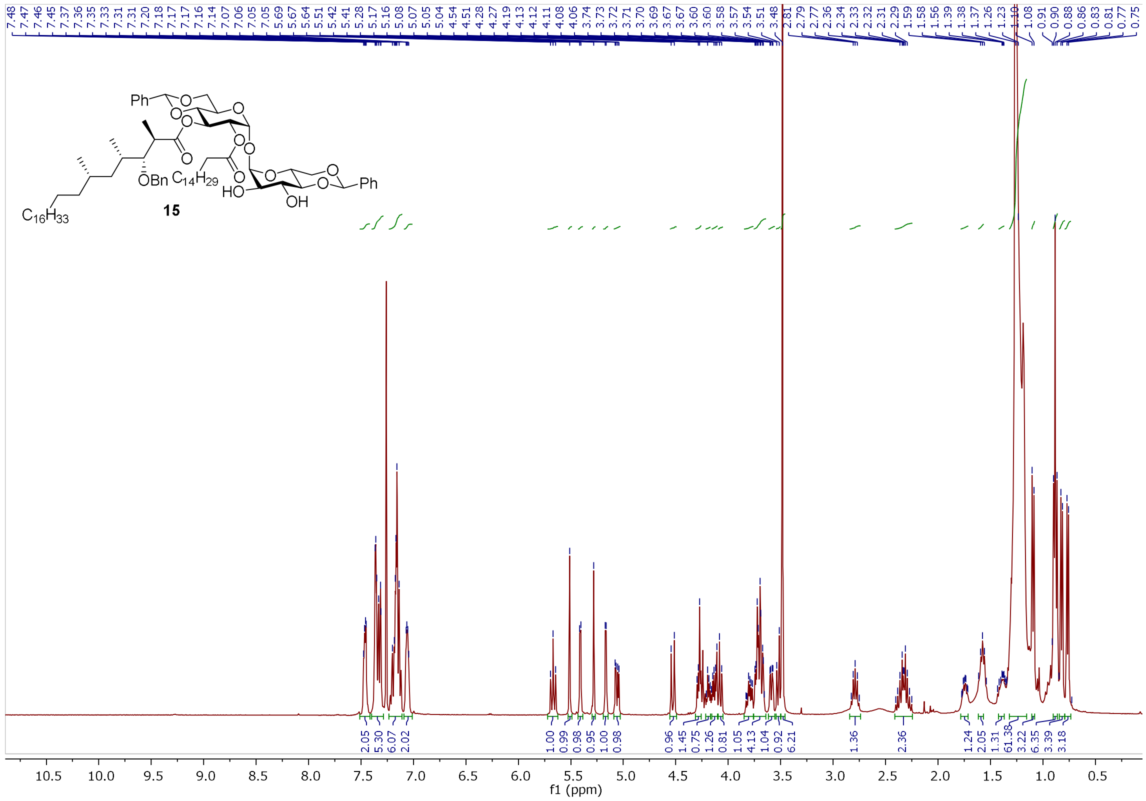
7.48 7.46 7.45 7.37 7.36 7.35 7.33 7.31 7.31 7.20 7.18 7.17 7.17 7.16 7.14 7.07 7.06 7.05 7.05 5.69 5.67 5.64 5.51 5.42 5.41 5.28 5.17 5.16 5.08 5.07 5.05 5.04 4.54 4.51 4.28 4.27 4.19 4.13 4.12 4.11 4.08 4.06 3.74 3.73 3.72 3.71 3.70 3.69 3.67 3.67 3.60 3.60 3.58 3.57 3.54 3.51 3.48 2.81 2.79 2.77 2.36 2.34 2.33 2.32 2.31 2.29 1.59 1.58 1.56 1.39 1.38 1.37 1.26 1.23 1.10 1.08 0.91 0.90 0.88 0.86 0.83 0.81 0.77 0.75
Ph
OBn
C14H29
HO
OH
Ph
C16H33
15
2.05 5.30 6.07 2.02 1.00 0.99 0.98 0.95 1.00 0.98 0.96 1.45 0.75 1.26 0.81 1.05 4.13 1.04 0.92 6.21 1.36 2.36 1.24 2.05 1.31 61.38 3.22 6.35 3.39 3.18
10.5 10.0 9.5 9.0 8.5 8.0 7.5 7.0 6.5 6.0 5.5 5.0 4.5 4.0 3.5 3.0 2.5 2.0 1.5 1.0 0.5
f1 (ppm)

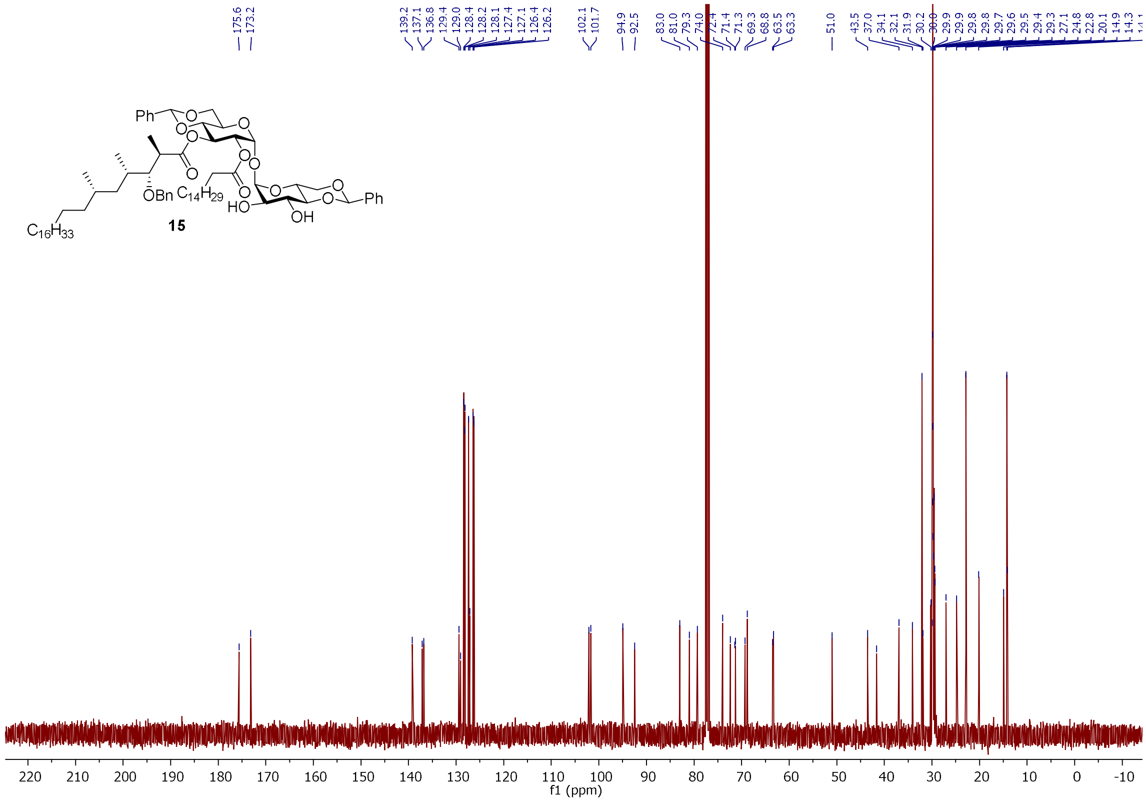
175.6 173.2 139.2 137.1 136.8 129.4 129.0 128.4 128.2 128.1 127.4 127.1 126.4 126.2 102.1 101.7 94.9 92.5 83.0 81.0 79.3 74.0 72.4 71.4 71.3 69.3 68.8 63.5 63.3 51.0 43.5 37.0 34.1 32.1 31.9 30.2 30.0 29.9 29.9 29.8 29.8 29.7 29.6 29.5 29.4 29.3 27.1 24.8 22.8 20.1 14.9 14.3
Ph
OBn
C14H29
HO
OH
Ph
C16H33
15
220 210 200 190 180 170 160 150 140 130 120 110 100 90 80 70 60 50 40 30 20 10 0 -10
f1 (ppm)

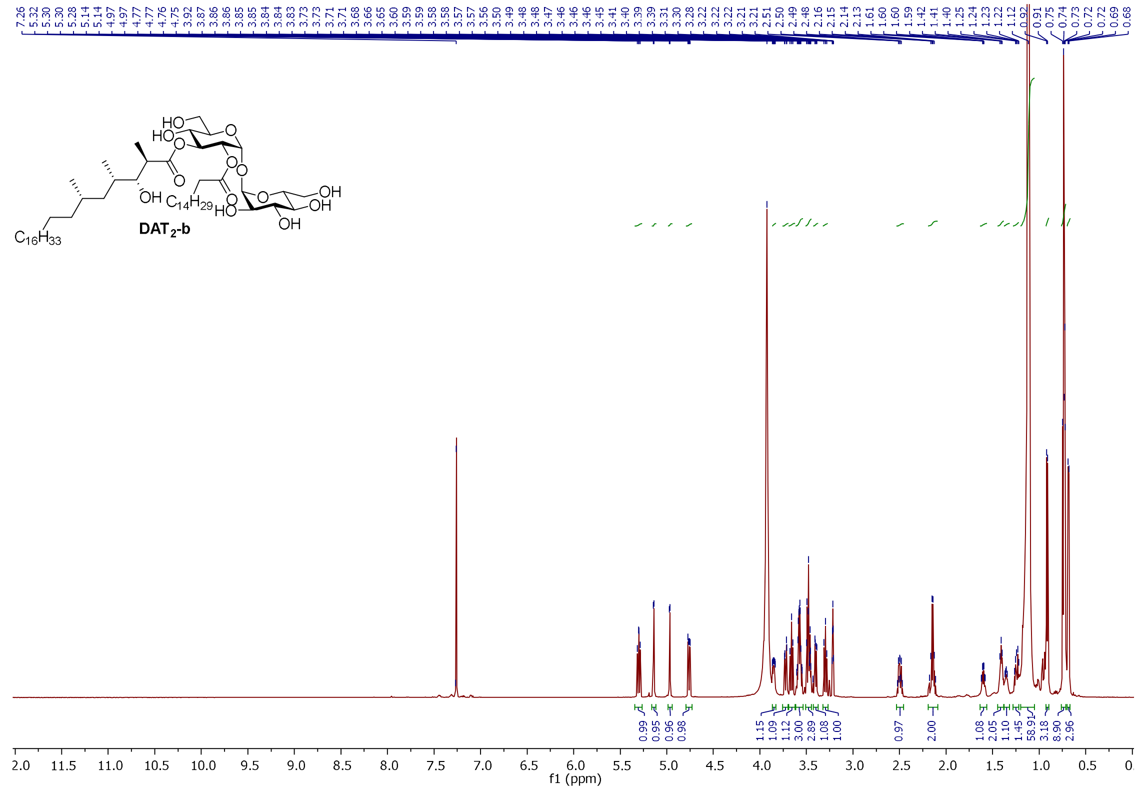

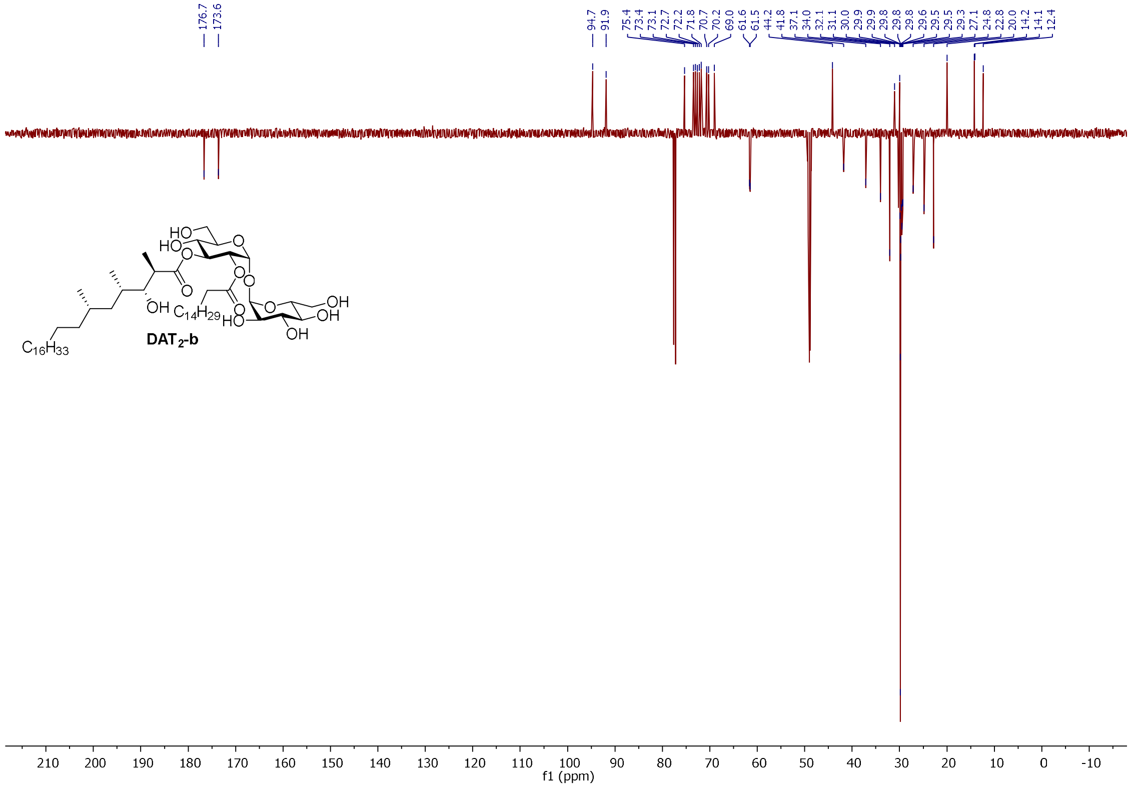

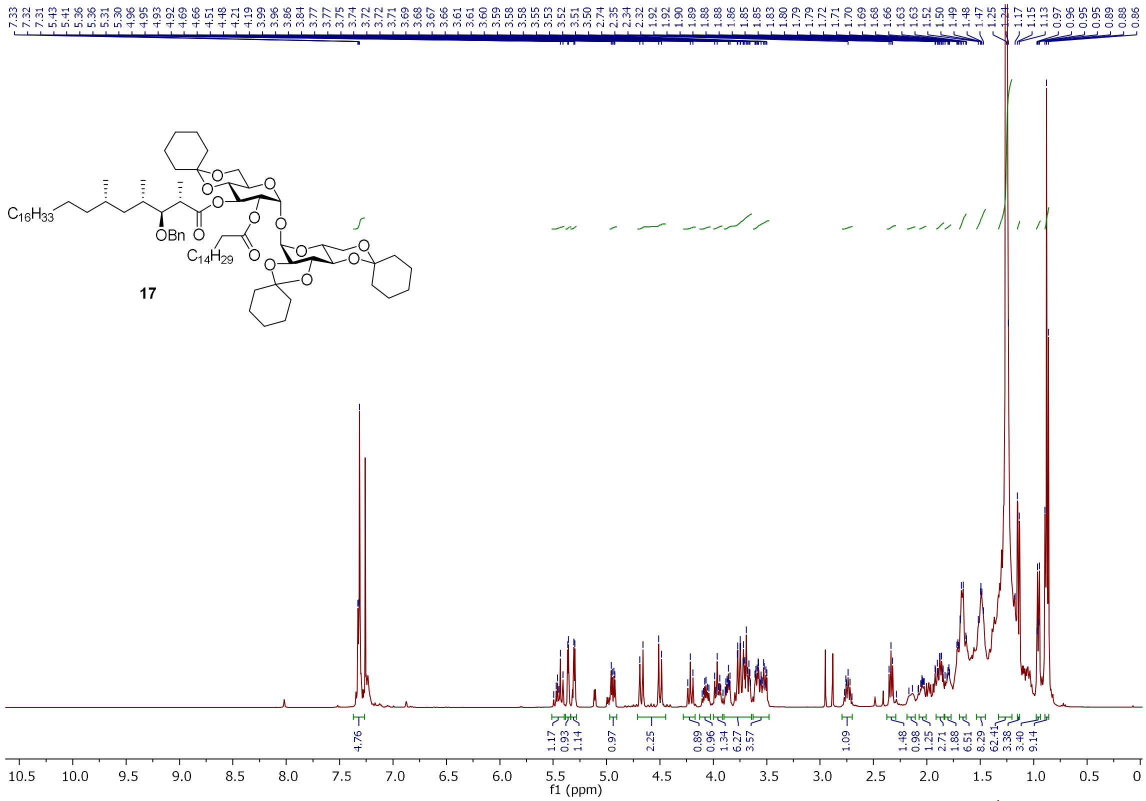
C16H33
OBn
C14H29
17
f1 (ppm)

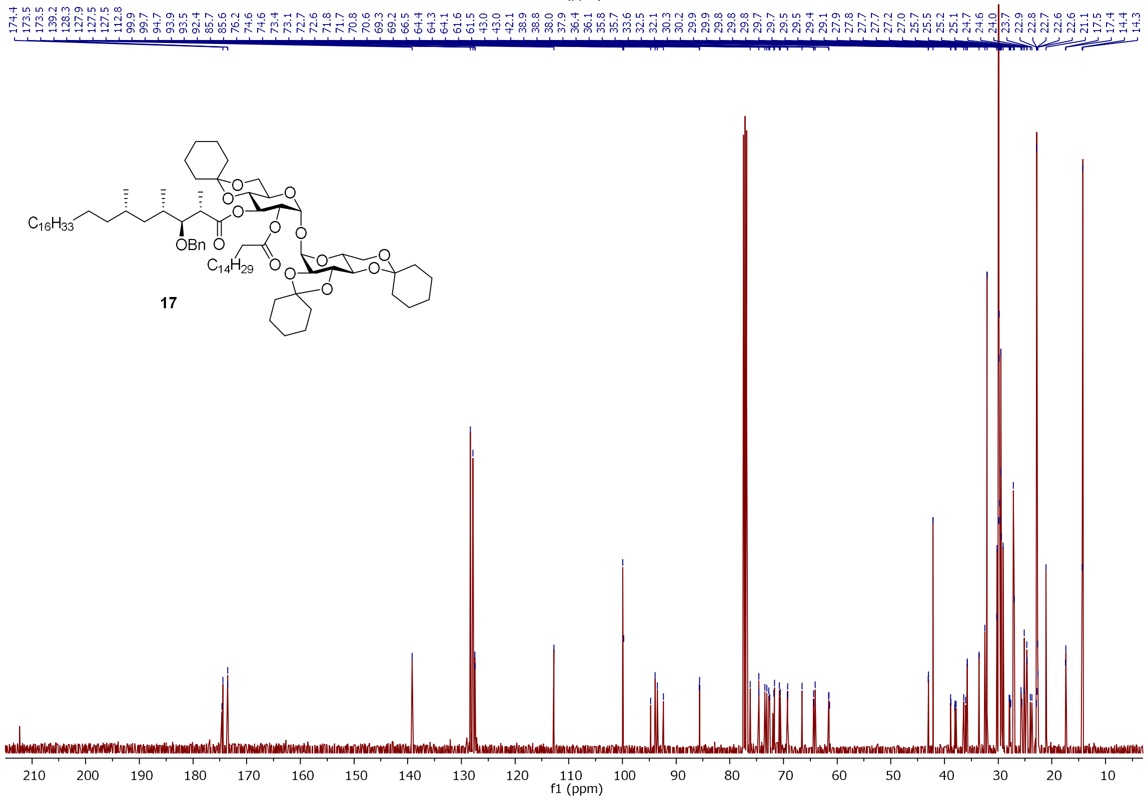
C16H33
OBn
C14H29
17
f1 (ppm)

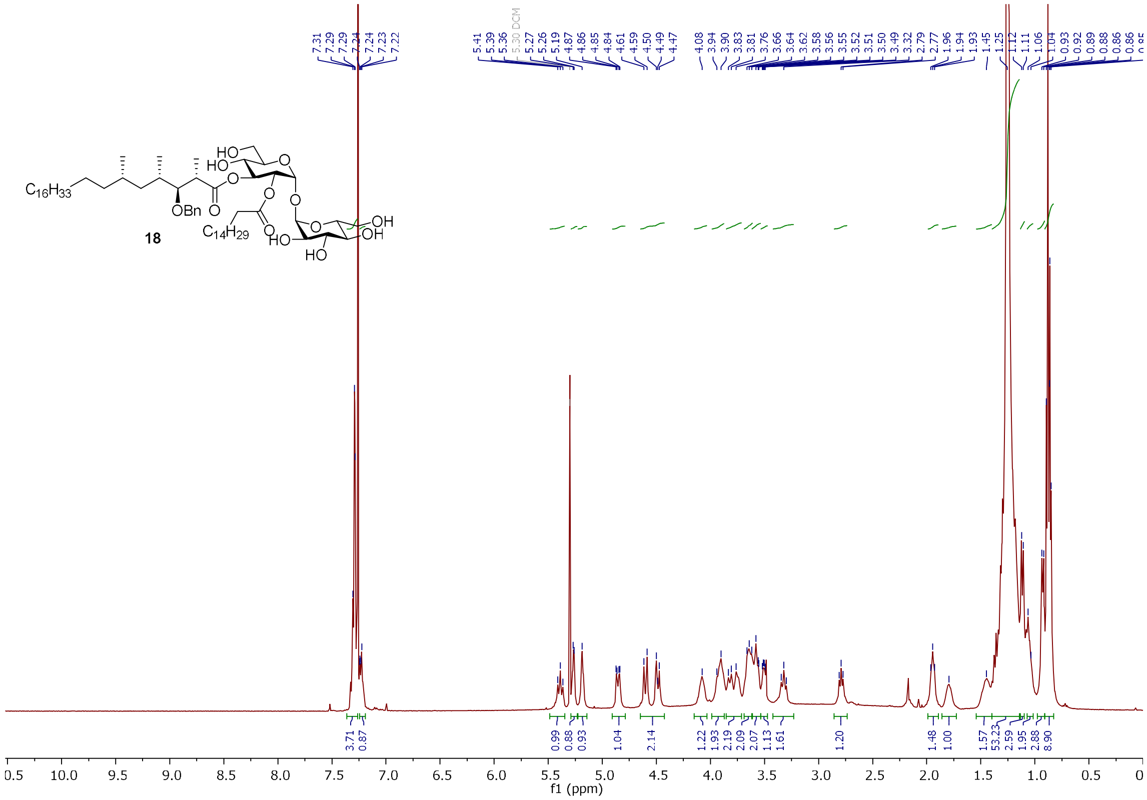
7.31 7.29 7.29 7.24 7.24 7.23 7.22
5.41 5.39 5.36 5.30 DCM 5.27 5.26 5.19 4.87 4.86 4.85 4.84 4.61 4.59 4.50 4.49 4.47
4.08 3.94 3.90 3.83 3.81 3.76 3.66 3.64 3.62 3.58 3.56 3.55 3.52 3.51 3.50 3.49 3.32 2.79 2.77 1.96 1.94 1.93
1.45 1.25 1.12 1.11 1.06 1.04 0.93 0.92 0.89 0.88 0.86 0.86
$C_{16}H_{33}$
OBn
$C_{14}H_{29}$
HO
OH
18
3.71 0.87 0.99 0.88 0.93 1.04 2.14 1.22 1.93 2.19 2.09 2.07 1.13 1.61 1.20 1.48 1.00 1.57 53.23 2.59 1.95 2.88 8.90
f1 (ppm)

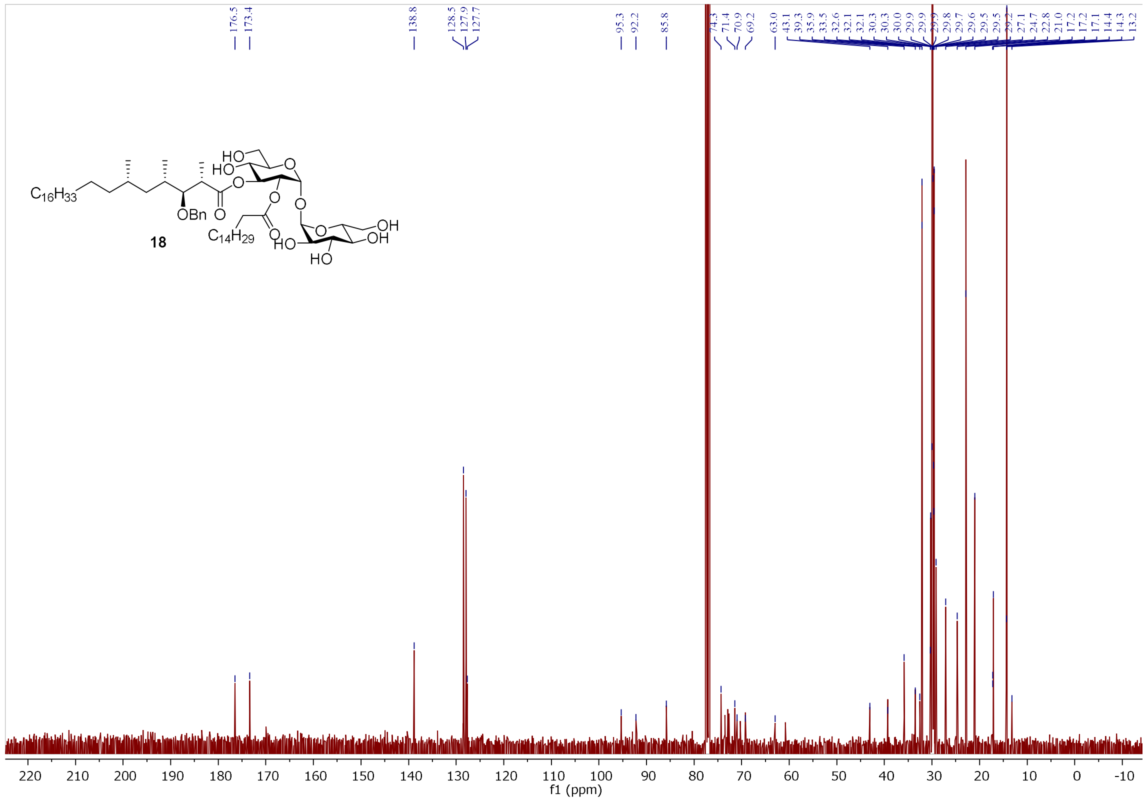
176.5 173.4 138.8 128.5 127.9 127.7 95.3 92.2 85.8
74.3 71.4 70.9 69.2 63.0 43.1 39.3 35.9 33.5 32.6 32.1 30.3 30.0 29.9 29.9 29.8 29.7 29.6 29.5 29.5 29.2 27.1 24.7 22.8 21.0 17.2 17.1 14.4 14.3 13.2
$C_{16}H_{33}$
OBn
$C_{14}H_{29}$
HO
OH
18
f1 (ppm)

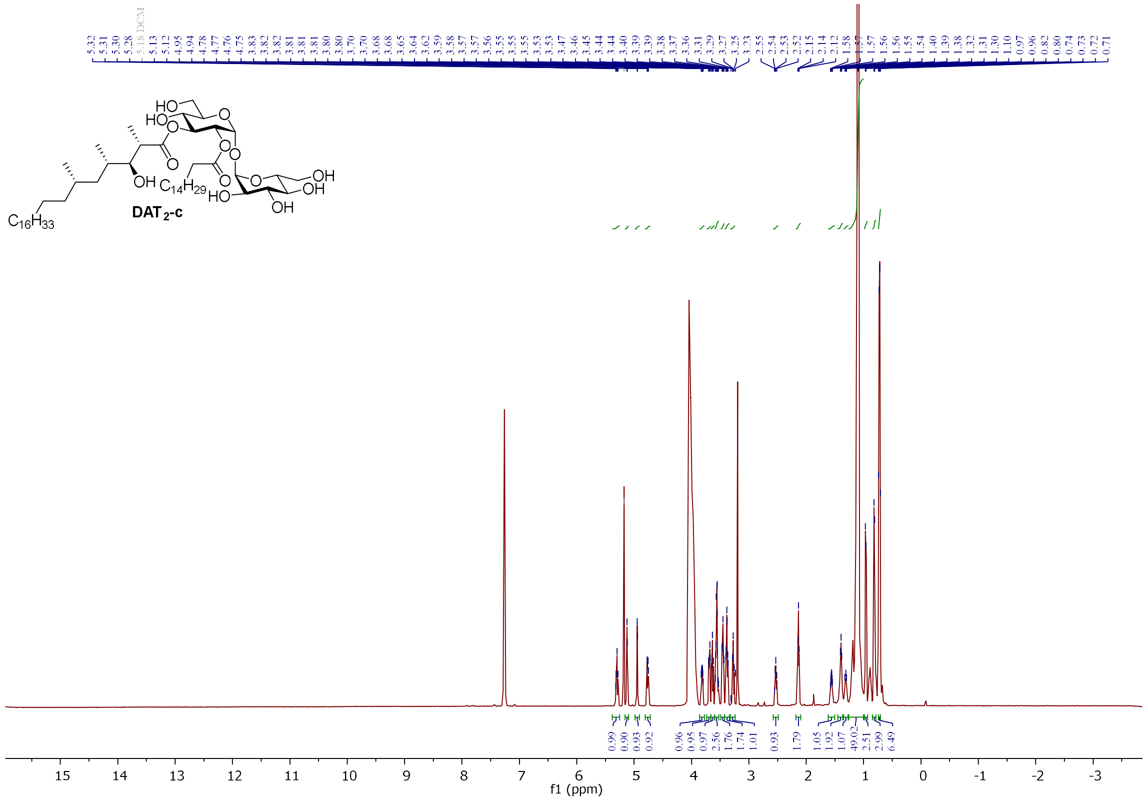
HO
HO
O
O
OH
C14H29
O
OH
OH
HO
OH
C16H33
DAT2-c
f1 (ppm)

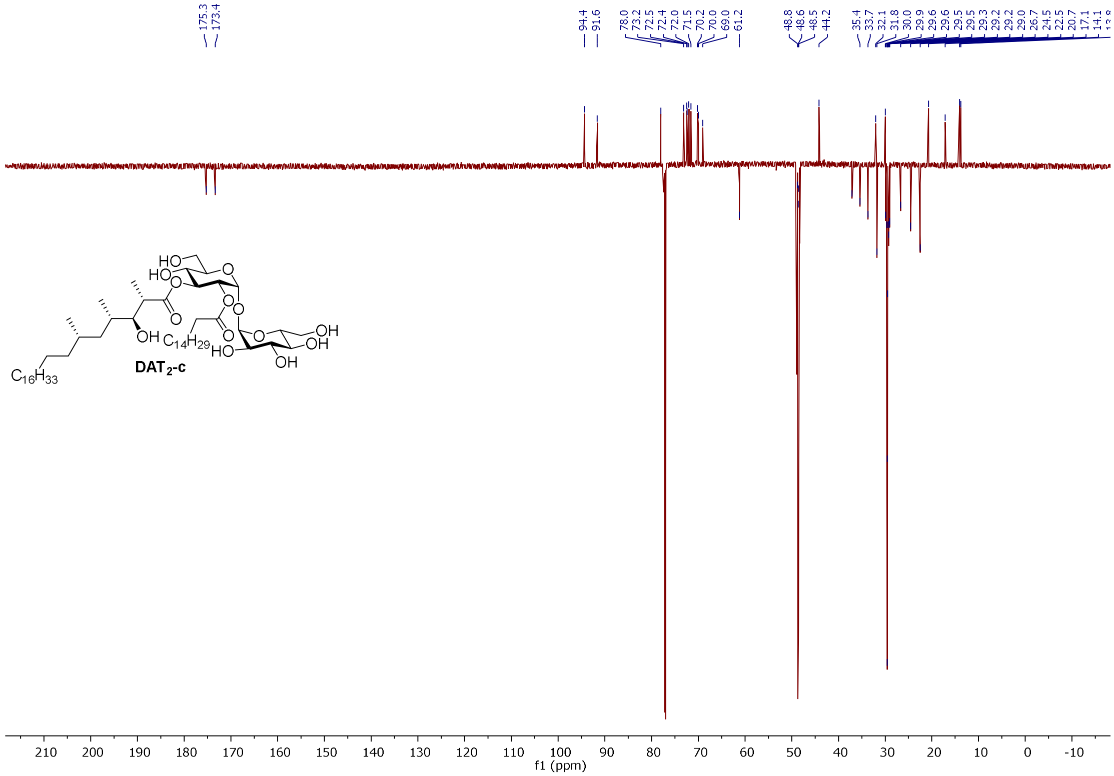
175.3
173.4
94.4
91.6
78.0
73.2
72.5
72.4
72.0
71.5
70.2
70.0
69.0
61.2
48.8
48.6
48.5
44.2
35.4
33.7
32.1
31.8
30.0
29.9
29.6
29.6
29.5
29.5
29.3
29.2
29.2
29.0
26.7
24.5
22.5
20.7
17.1
14.1
[illegible]
HO
HO
O
O
OH
C14H29
O
OH
OH
HO
OH
C16H33
DAT2-c
f1 (ppm)

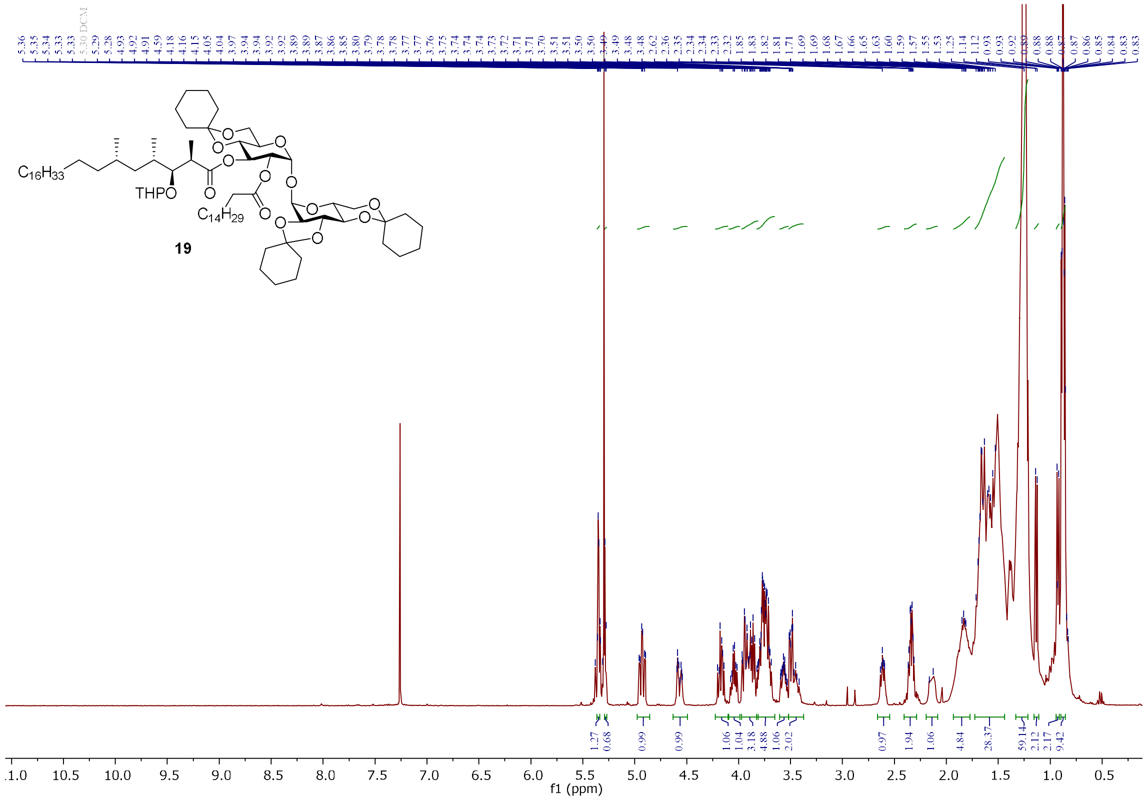

C16H33
THPO
C14H29
19
f1 (ppm)

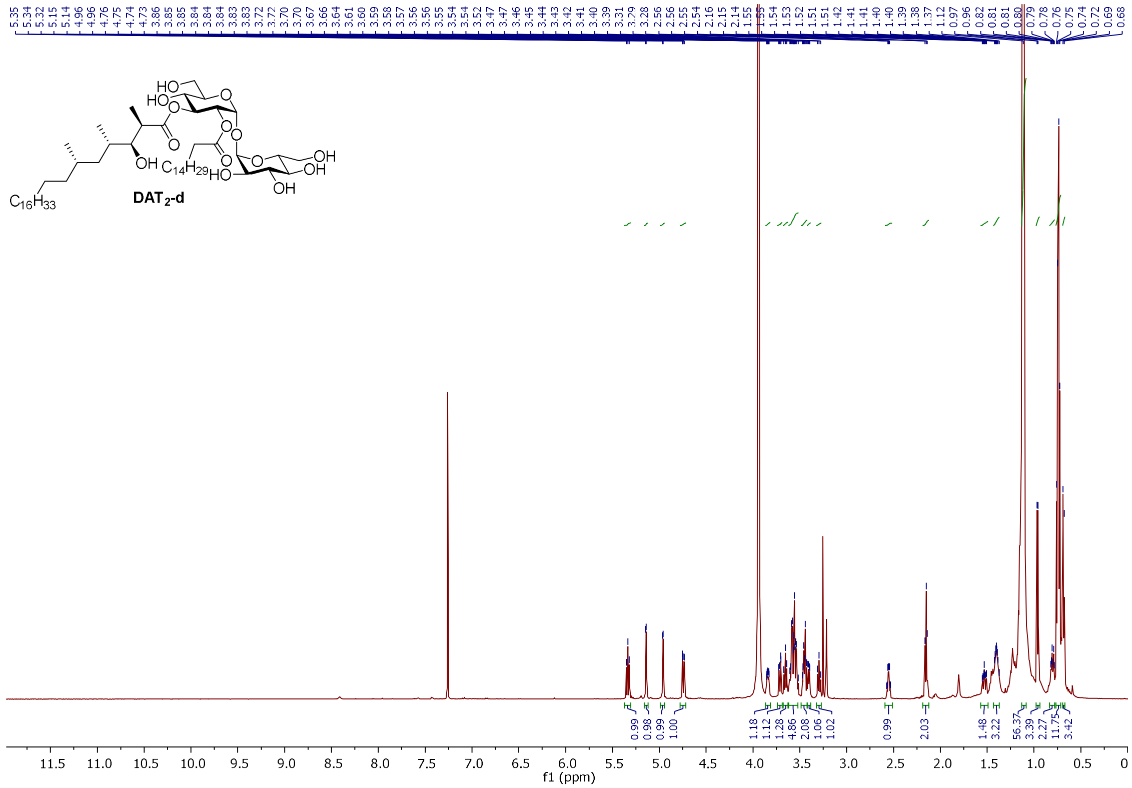
DAT2-d
f1 (ppm)

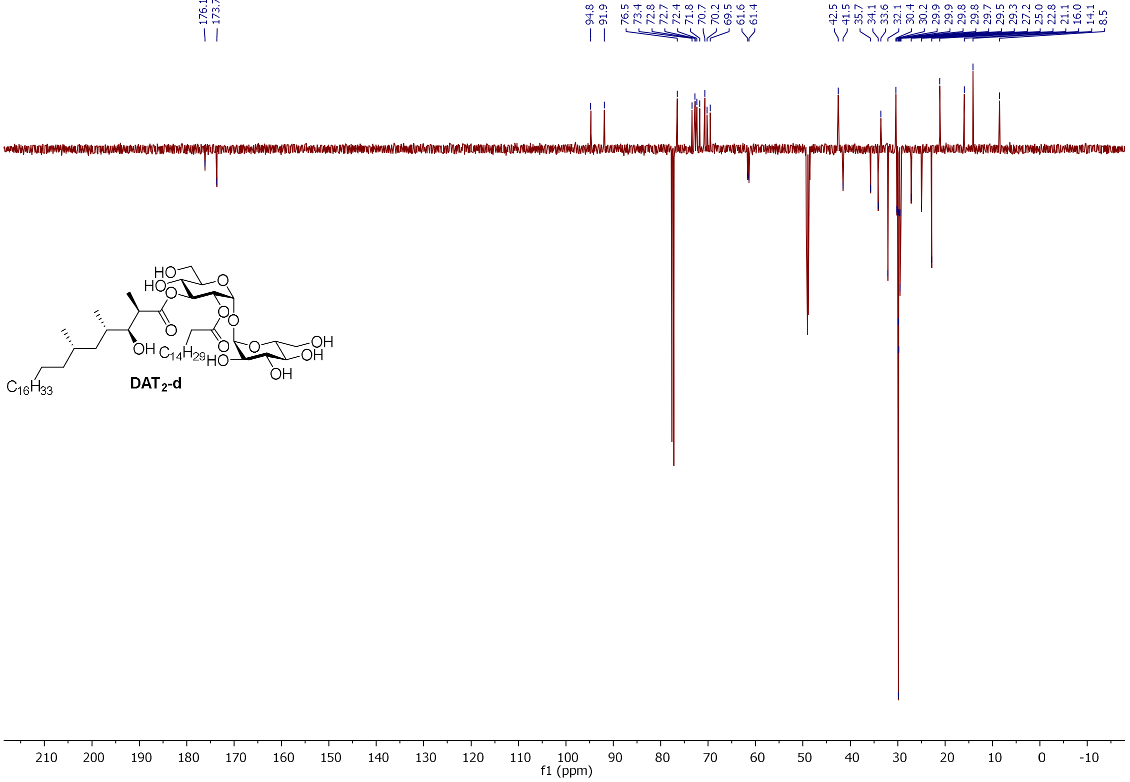
DAT2-d
f1 (ppm)

HRMS spectra

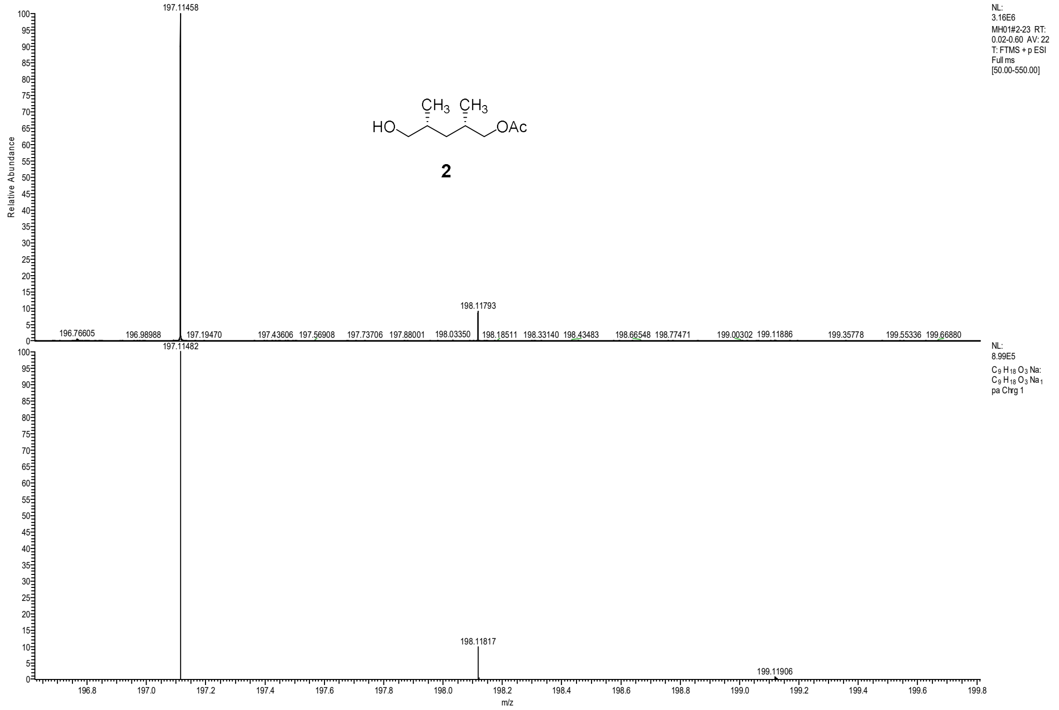

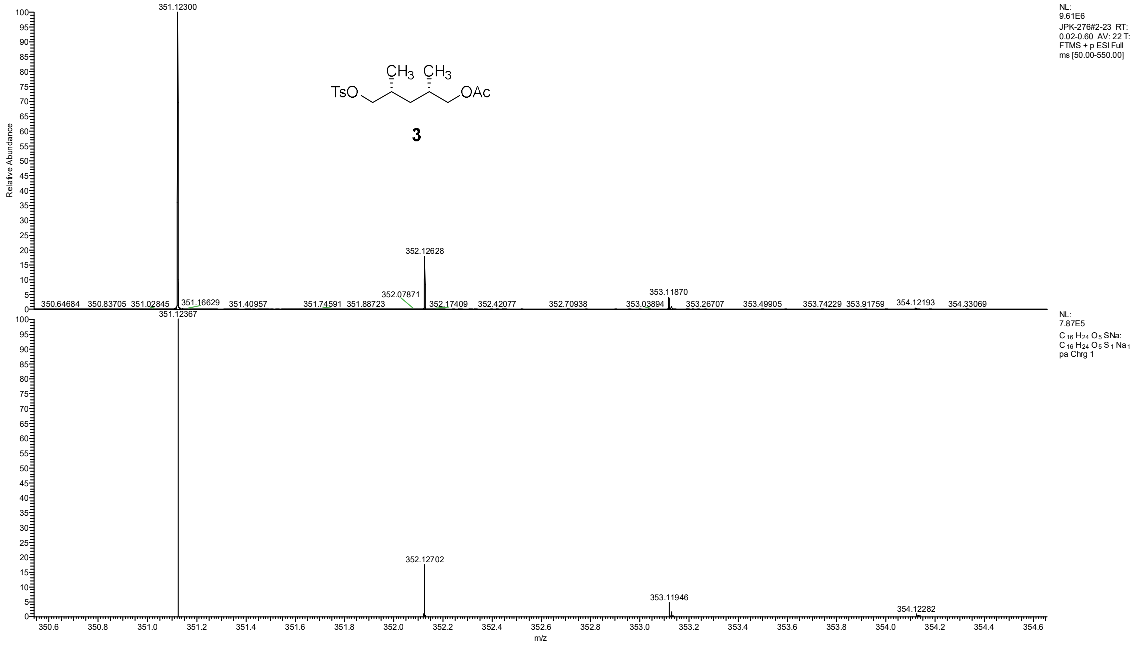

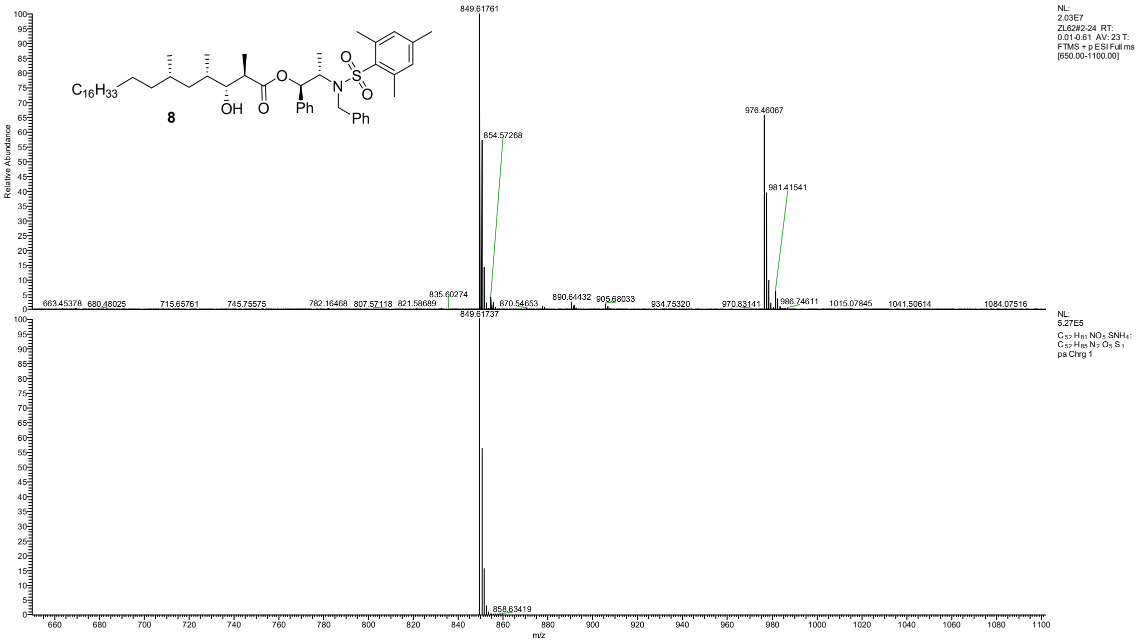
C16H33
OH
O
O
Ph
N
S
O
O
Ph
8
849.61761
854.57268
976.46067
981.41541
NL: 2.03E7 ZL62#2-24 RT: 0.01-0.61 AV: 23 T: FTMS + p ESI Full ms [650.00-1100.00]
849.61737
858.63419
NL: 5.27E5 C52 H81 NO5 SNH4: C52 H85 N2 O5 S1 pa Chrg 1
Relative Abundance
m/z

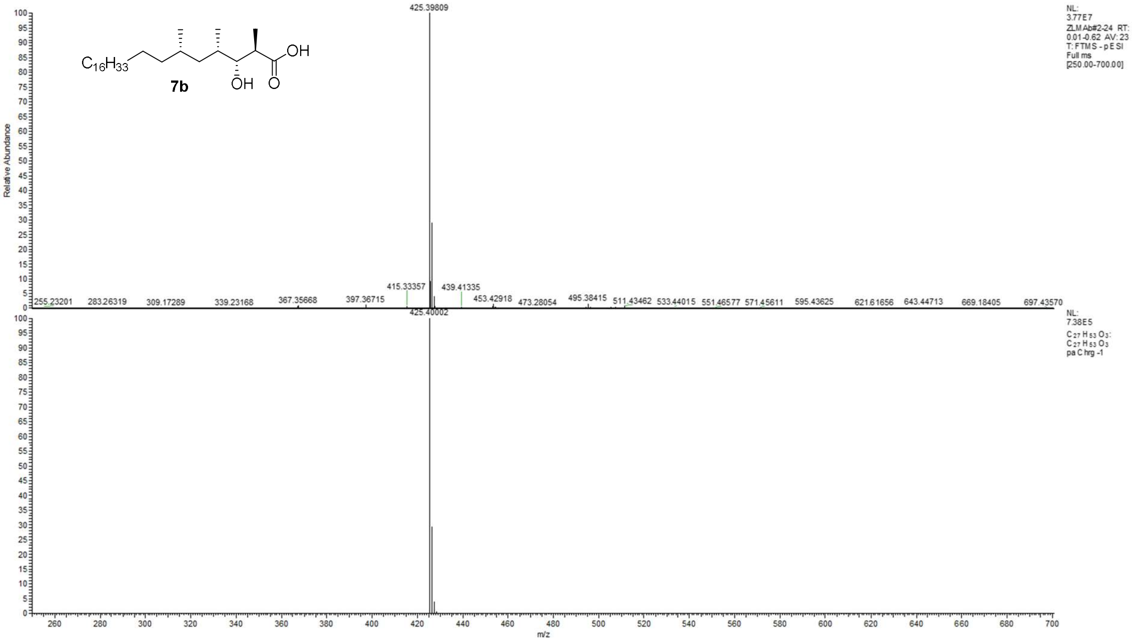
C16H33
OH
O
OH
7b
425.39809
415.33357
439.41335
NL: 3.77E7 ZLMAb#2-24 RT: 0.01-0.62 AV: 23 T: FTMS - p ESI Full ms [250.00-700.00]
425.40002
NL: 7.38E5 C27 H53 O3: C27 H53 O3 pa Chrg -1
Relative Abundance
m/z

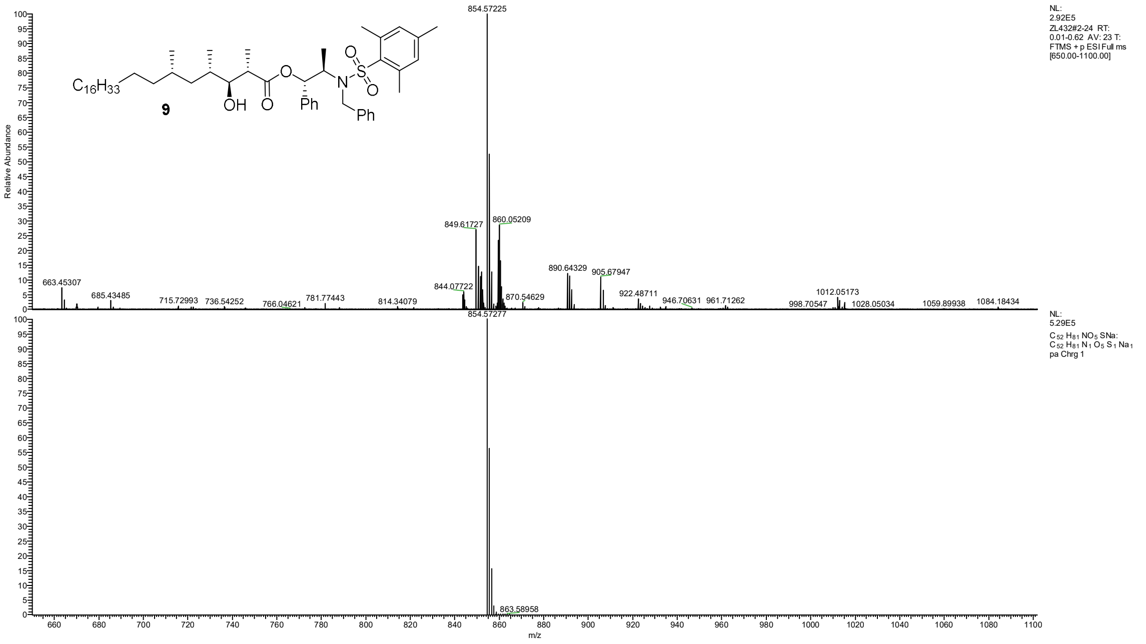
854.57225
849.61727
860.05209
663.45307
685.43485
715.72993
736.54252
766.04621
781.77443
814.34079
844.07722
870.54629
890.64329
905.67947
922.48711
946.70631
961.71262
998.70547
1012.05173
1028.05034
1059.89938
1084.18434
NL: 2.92E5
ZL432#2-24 RT: 0.01-0.62 AV: 23 T: FTMS + p ESI Full ms [650.00-1100.00]
854.57277
863.58958
NL: 5.29E5
C52 H81 NO5 SNa: C52 H81 N1 O5 S1 Na1 pa Chrg 1
Relative Abundance
m/z
C16H33
OH
O
Ph
N
S
Ph
9

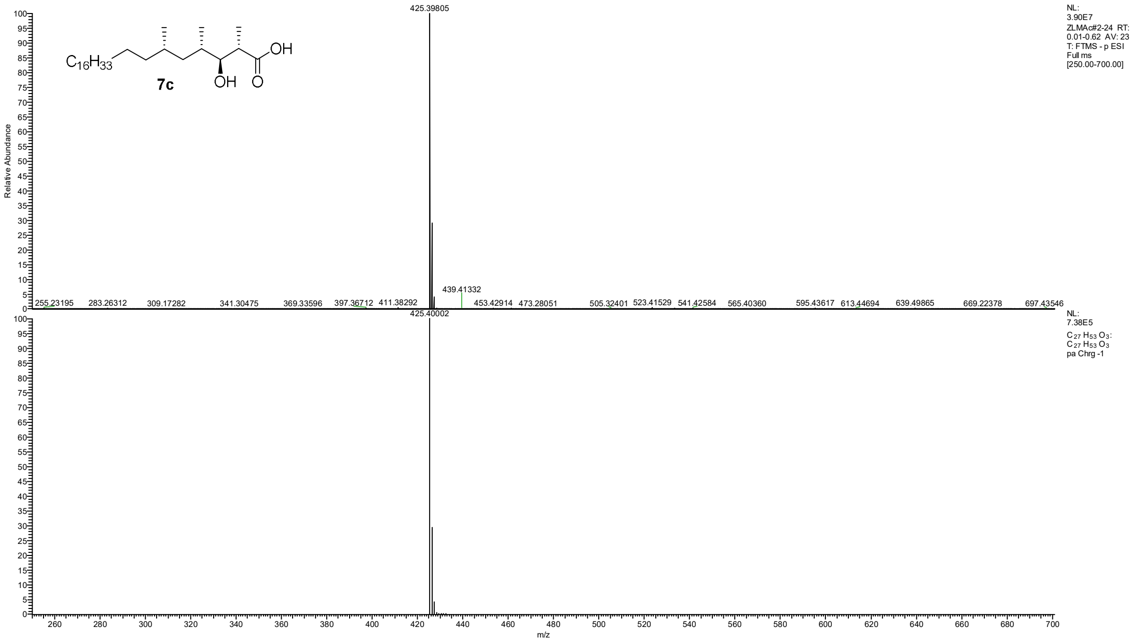
425.39805
255.23195
283.26312
309.17282
341.30475
369.33596
397.36712
411.38292
439.41332
453.42914
473.28051
505.32401
523.41529
541.42584
565.40360
595.43617
613.44694
639.49865
669.22378
697.43546
NL: 3.90E7
ZLMAci#2-24 RT: 0.01-0.62 AV: 23 T: FTMS - p ESI Full ms [250.00-700.00]
425.40002
NL: 7.38E5
C27 H53 O3: C27 H53 O3 pa Chrg -1
Relative Abundance
m/z
C16H33
OH
O
OH
7c

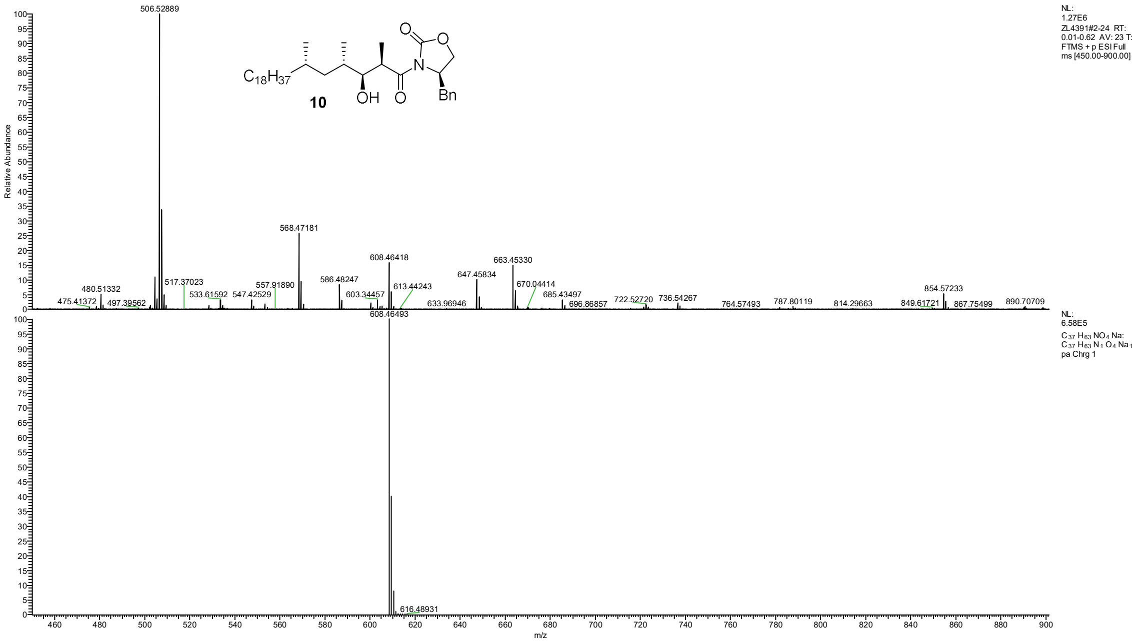
NL:
1.27E6
ZL4391#2-24 RT:
0.01-0.62 AV: 23 T:
FTMS + p ESI Full
ms [450.00-900.00]
506.52889
568.47181
608.46418
663.45330
647.45834
NL:
6.58E5
608.46493
616.48931
Relative Abundance
m/z
10
C18H37
OH
Bn

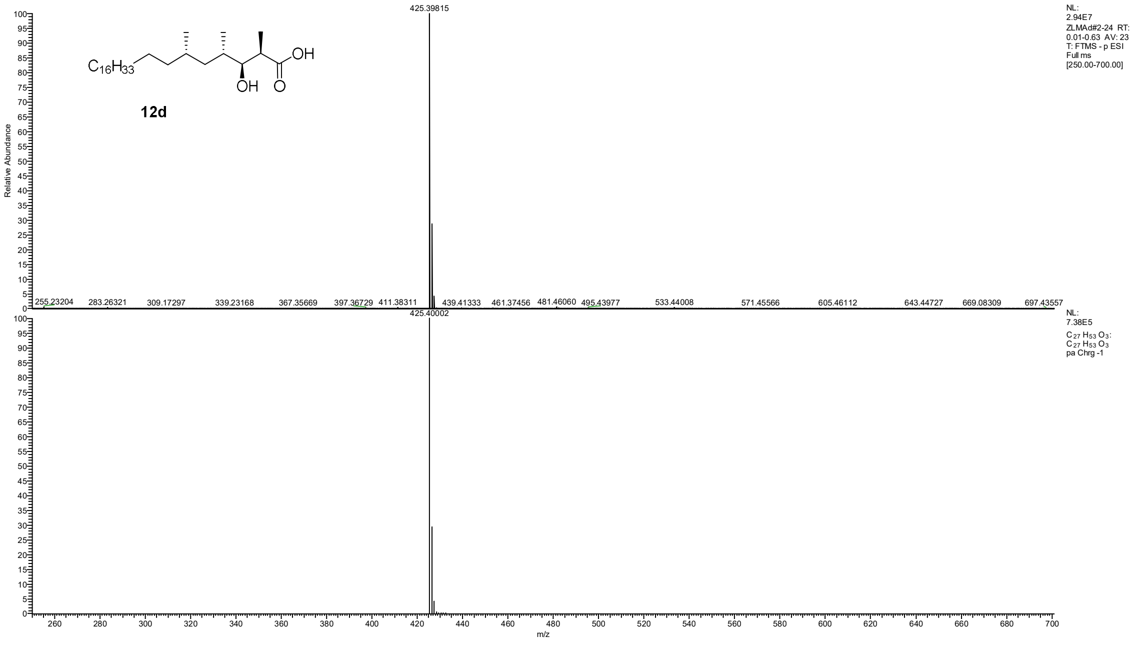
NL:
2.94E7
ZLMAd#2-24 RT:
0.01-0.63 AV: 23
T: FTMS - p ESI
Full ms
[250.00-700.00]
425.39815
NL:
7.38E5
425.40002
Relative Abundance
m/z
12d
C16H33
OH

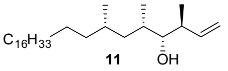
C16H33
11
OH

GC-MS

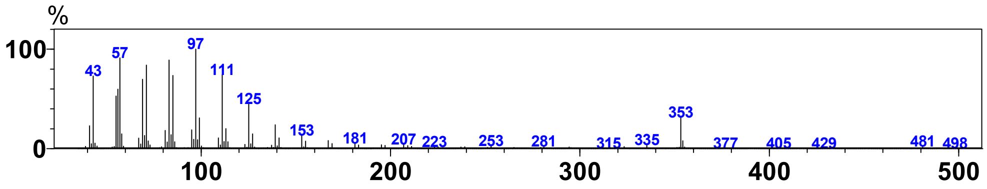
%
100
0
43
57
97
111
125
153
181
207
223
253
281
315
335
353
377
405
429
481
498
100
200
300
400
500

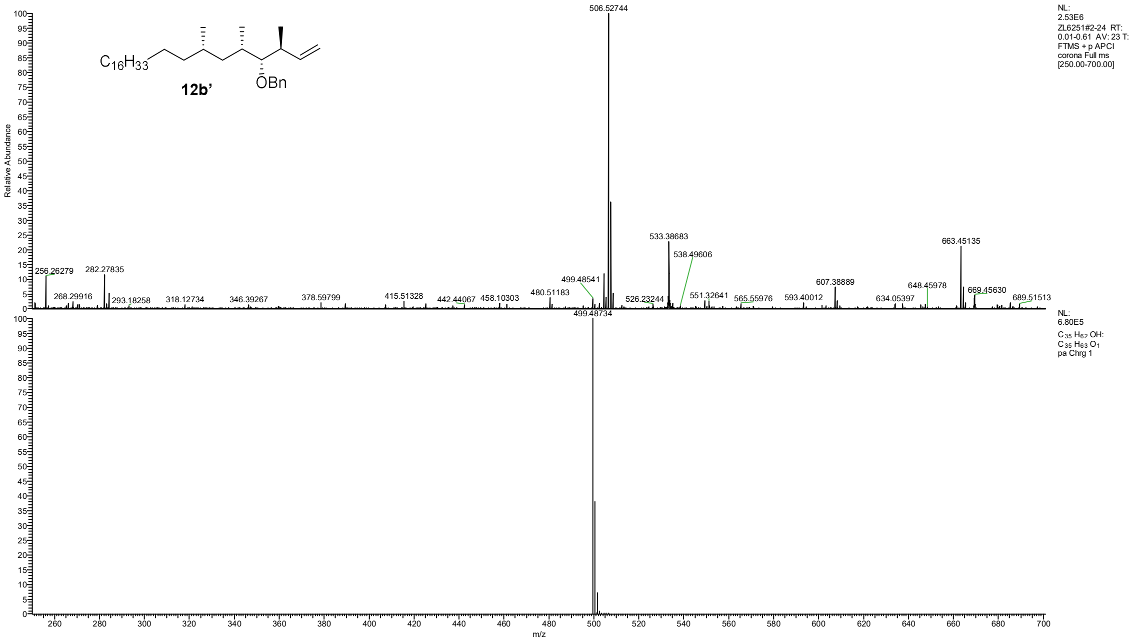
C16H33
12b'
OBn
Relative Abundance
506.52744
533.38683
538.49606
663.45135
256.26279
282.27835
268.29916
293.18258
318.12734
346.39267
378.59799
415.51328
442.44067
458.10303
480.51183
499.48541
526.23244
551.32641
565.55976
593.40012
607.38889
634.05397
648.45978
669.45630
689.51513
NL:
2.53E6
ZL6251#2-24 RT:
0.01-0.61 AV: 23 T:
FTMS + p APCI
corona Full ms
[250.00-700.00]
499.48734
NL:
6.80E5
C35 H62 OH:
C35 H63 O1
pa Chrg 1
m/z

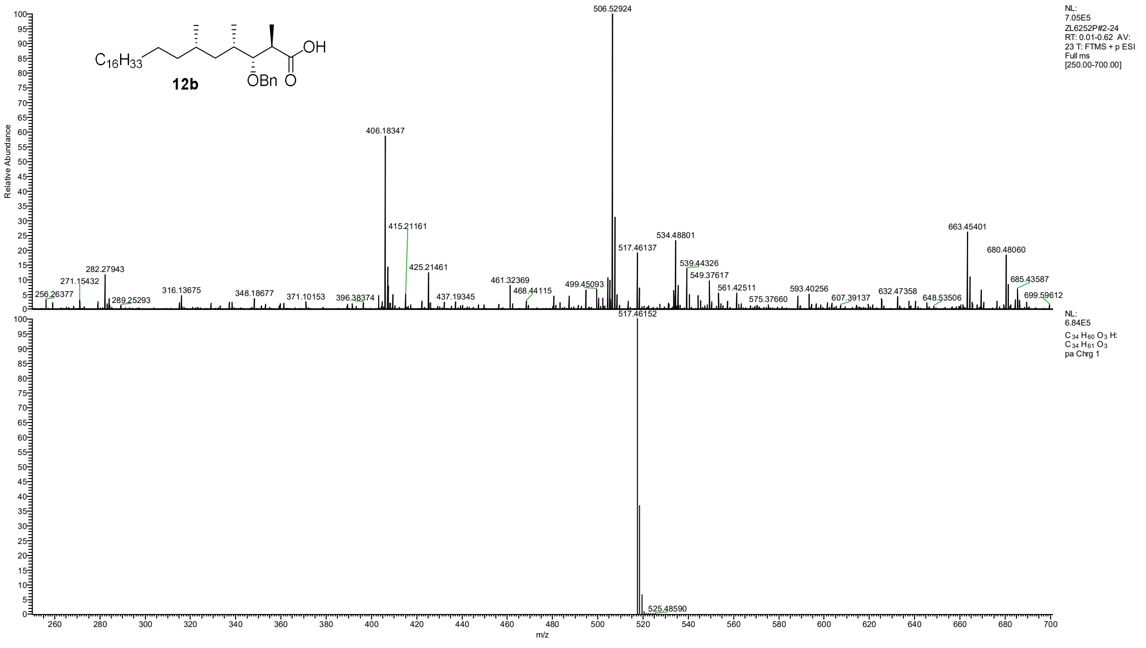

12b
Relative Abundance
m/z
NL: 7.05E5
ZL6252P#2-24
RT: 0.01-0.62 AV: 23 T: FTMS + p ESI Full ms [250.00-700.00]
NL: 6.84E5
C34 H60 O3 H: C34 H61 O3 pa Chrg 1
506.52924
406.18347
517.46137
534.48801
663.45401
680.48060
517.46152
525.48590

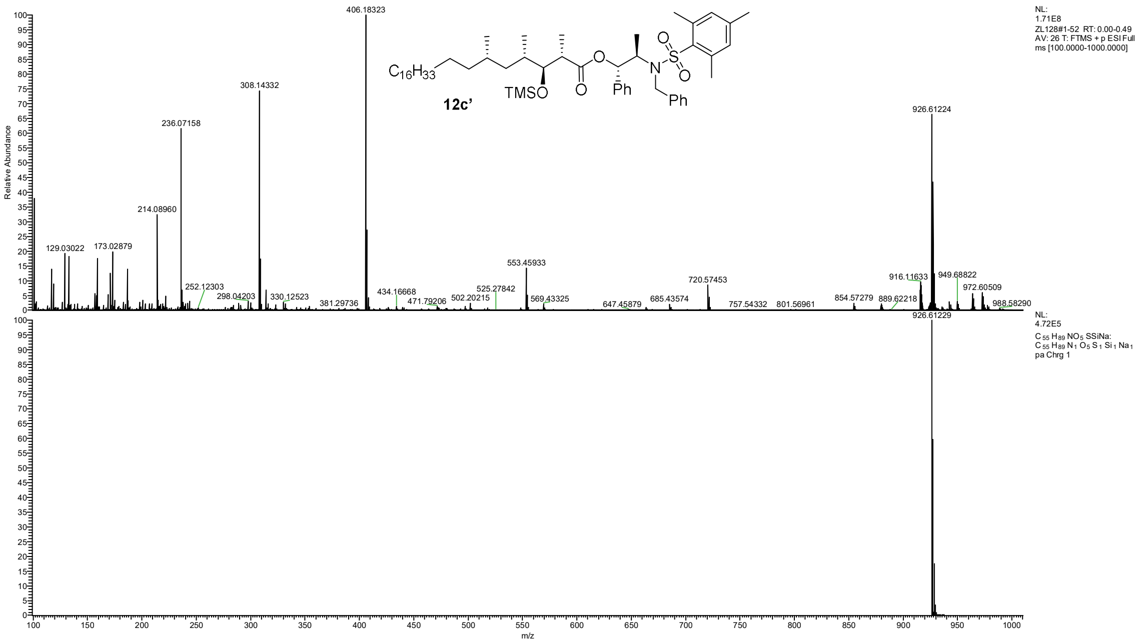

12c'
Relative Abundance
m/z
NL: 1.71E8
ZL128#1-52 RT: 0.00-0.49 AV: 26 T: FTMS + p ESI Full ms [100.0000-1000.0000]
NL: 4.72E5
C55 H89 NO5 SSiNa: C55 H89 N1 O5 S1 Si1 Na1 pa Chrg 1
406.18323
308.14332
236.07158
214.08960
926.61224
553.45933
720.57453
926.61229

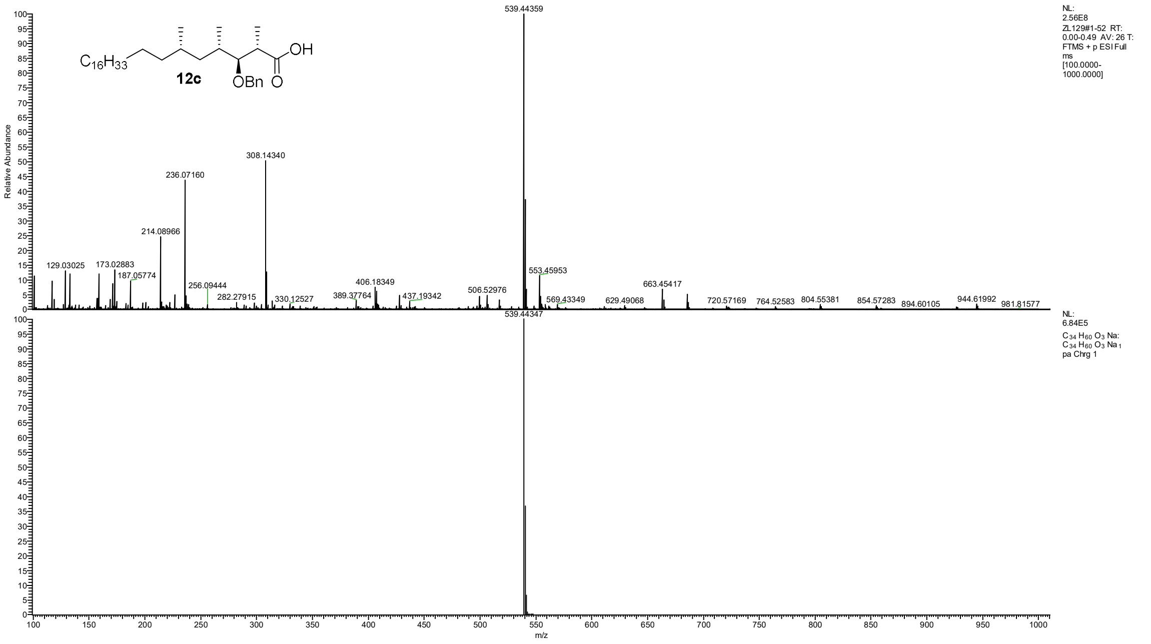

12c
OBn
OH
NL: 2.56E8
ZL129#1-52 RT: 0.00-0.49 AV: 26 T: FTMS + p ESI Full ms [100.0000-1000.0000]
NL: 6.84E5
Relative Abundance
m/z

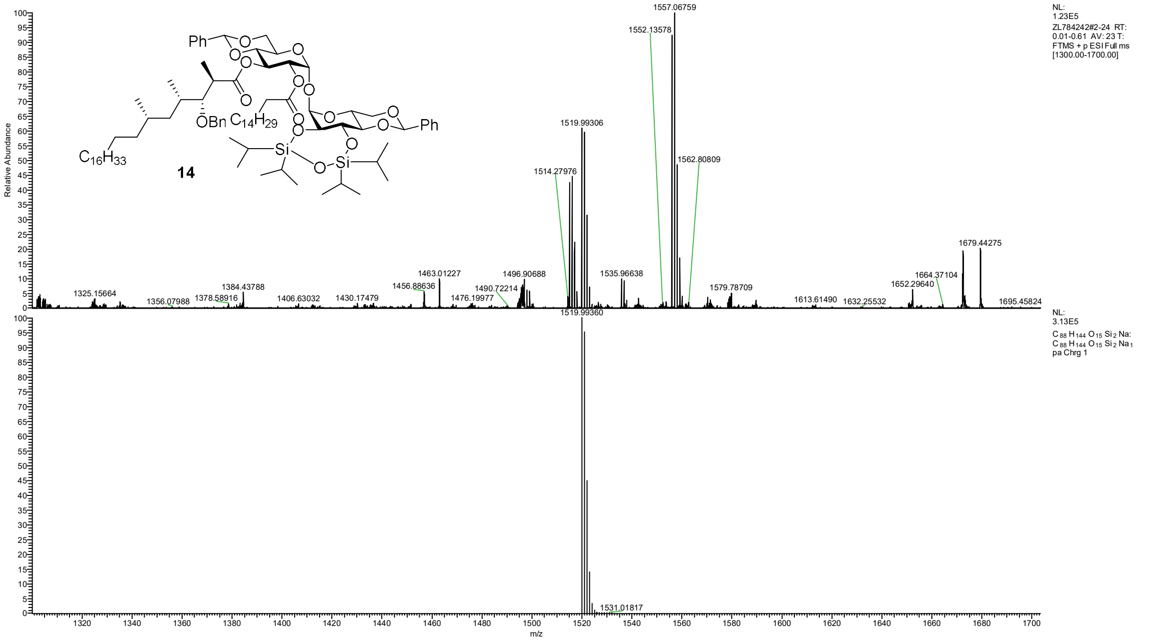

14
NL: 1.23E5
ZL784242#2-24 RT: 0.01-0.61 AV: 23 T: FTMS + p ESI Full ms [1300.00-1700.00]
NL: 3.13E5
Relative Abundance
m/z

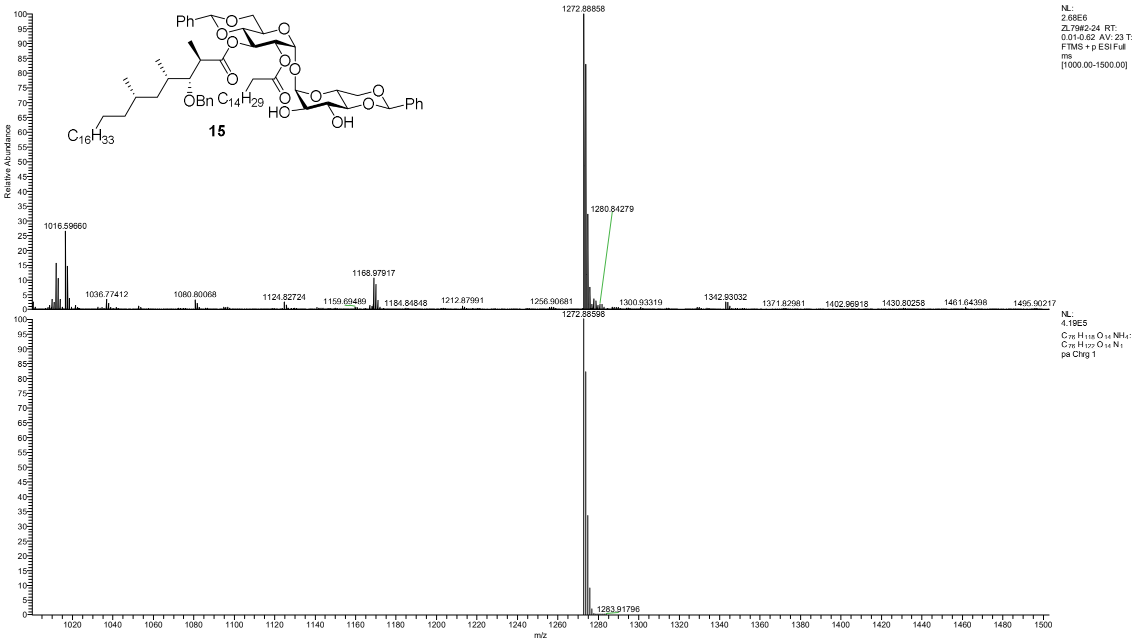

NL: 2.68E6
ZL79#2-24 RT: 0.01-0.62 AV: 23 T: FTMS + p ESI Full ms [1000.00-1500.00]
NL: 4.19E5
C 76 H 118 O 14 NH4: C 76 H 122 O 14 N 1 pa Chrg 1
Relative Abundance
m/z
15
C16H33
OBn C14H29
HO
OH
Ph
1272.88858
1280.84279
1016.59660
1168.97917
1036.77412
1080.80068
1124.82724
1159.69489
1184.84848
1212.87991
1256.90681
1300.93319
1342.93032
1371.82981
1402.96918
1430.80258
1461.64398
1495.90217
1272.88598
1283.91796

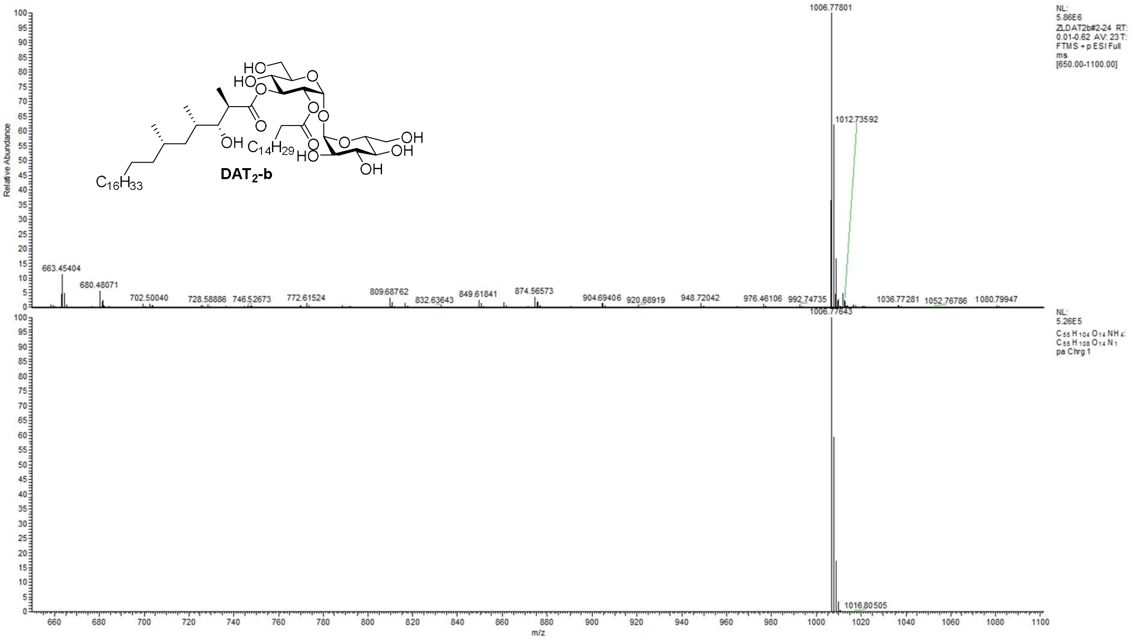

NL: 5.86E6
ZLDAT2b#2-24 RT: 0.01-0.62 AV: 23 T: FTMS + p ESI Full ms [650.00-1100.00]
NL: 5.26E5
C 55 H 104 O 14 NH4: C 55 H 108 O 14 N 1 pa Chrg 1
Relative Abundance
m/z
DAT2-b
C16H33
OH C14H29
HO
OH
1006.77801
1012.73592
663.45404
680.48071
702.50040
728.58886
746.52673
772.61524
809.68762
832.63643
849.61841
874.56573
904.69406
920.68919
948.72042
976.46106
992.74735
1036.77281
1052.76786
1080.79947
1006.77643
1016.80505

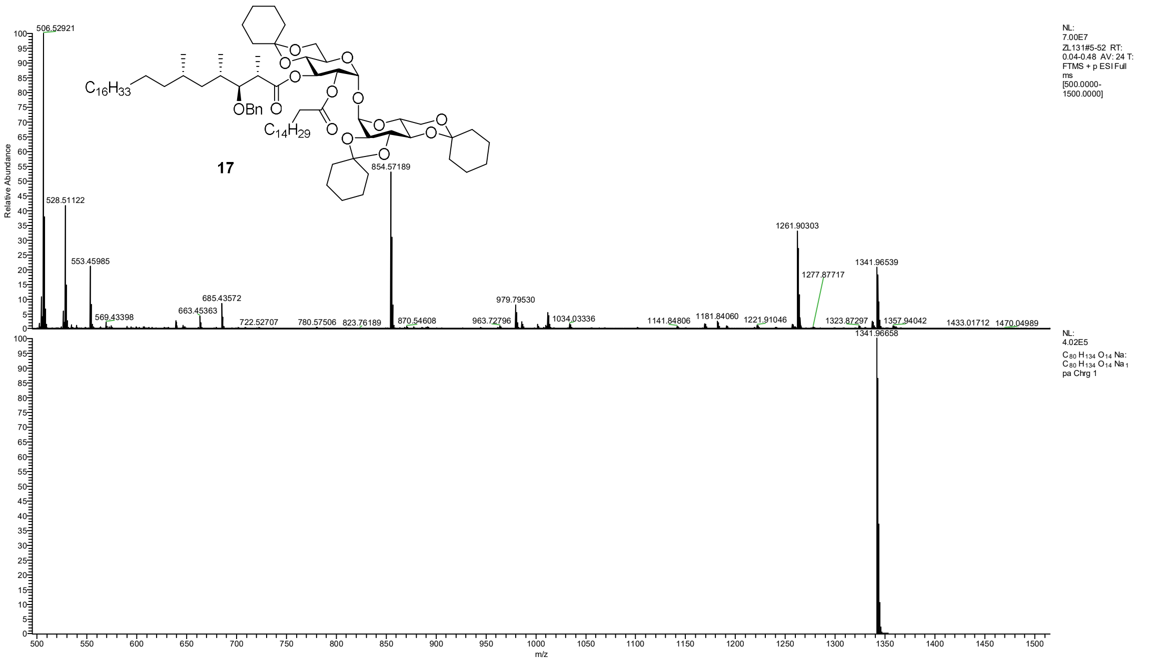

17
C16H33
OBn
C14H29
Relative Abundance
m/z
506.52921
528.51122
553.45985
569.43398
663.45363
685.43572
722.52707
780.57506
823.76189
854.57189
870.54608
963.72796
979.79530
1034.03336
1141.84806
1181.84060
1221.91046
1261.90303
1277.87717
1323.87297
1341.96539
1357.94042
1433.01712
1470.04989
1341.96658
NL:
7.00E7
ZL131#5-52 RT: 0.04-0.48 AV: 24 T: FTMS + p ESI Full ms [500.0000-1500.0000]
NL:
4.02E5
C80 H134 O14 Na: C80 H134 O14 Na1 pa Chrg 1

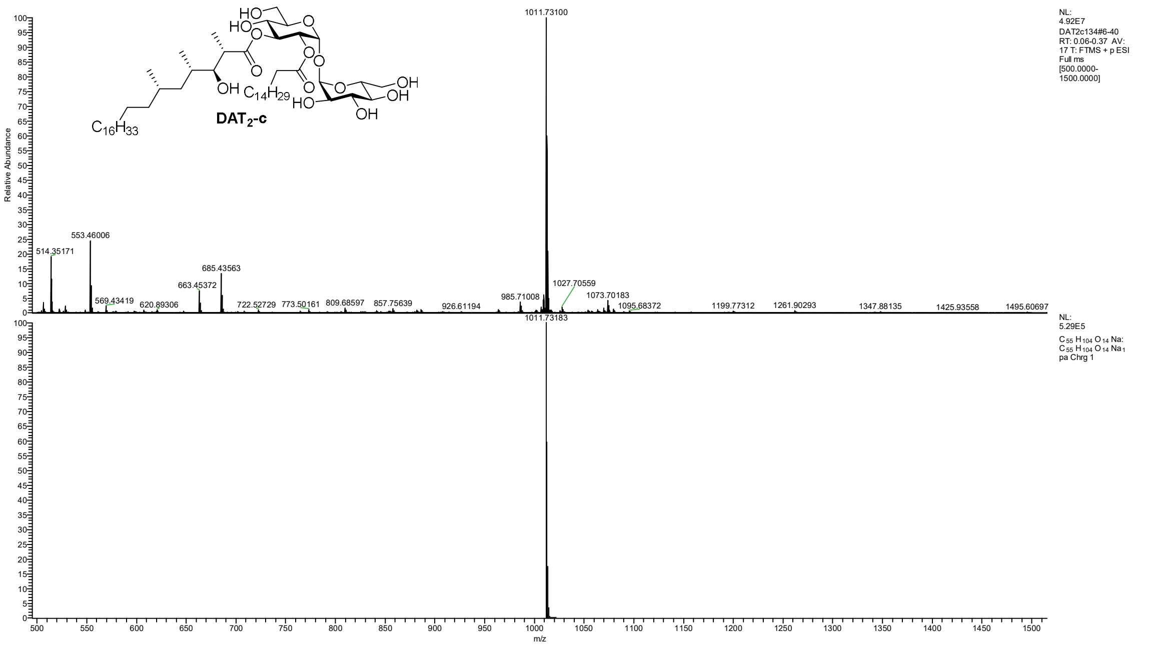

DAT2-c
C16H33
C14H29
Relative Abundance
m/z
1011.73100
514.35171
553.46006
569.43419
620.89306
663.45372
685.43563
722.52729
773.50161
809.68597
857.75639
926.61194
985.71008
1027.70559
1073.70183
1095.68372
1199.77312
1261.90293
1347.88135
1425.93558
1495.60697
1011.73183
NL:
4.92E7
DAT2c134#6-40 RT: 0.06-0.37 AV: 17 T: FTMS + p ESI Full ms [500.0000-1500.0000]
NL:
5.29E5
C55 H104 O14 Na: C55 H104 O14 Na1 pa Chrg 1

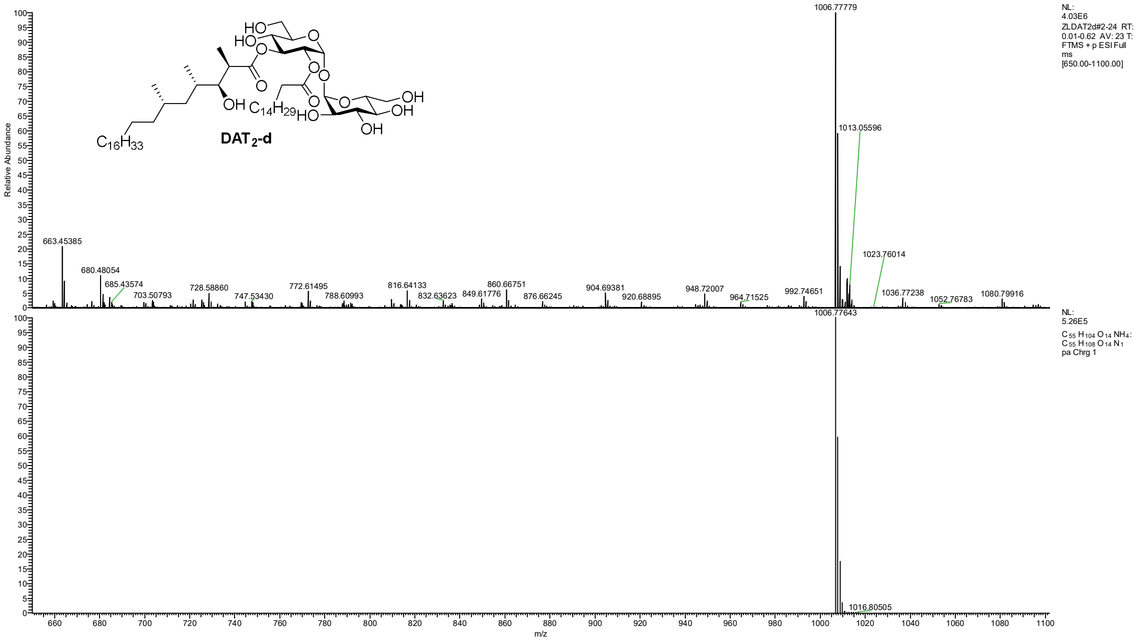

DAT2-d
NL: 4.03E6
ZLDAT2d#2-24 RT: 0.01-0.62 AV: 23 T: FTMS + p ESI Full ms [650.00-1100.00]
NL: 5.26E5
pa Chrg 1
Relative Abundance
m/z
